# Coordinated gas release among the physostomous fish sprat (*Sprattus sprattus*)

**DOI:** 10.1038/s41598-021-92585-2

**Published:** 2021-06-23

**Authors:** Stein Kaartvedt, Karl I. Ugland, Jan Heuschele, Ingrid Solberg

**Affiliations:** 1 Department of Biosciences, University of Oslo, Blindern, PO Box 1066, 0316 Oslo, Norway; 2 Norwegian Institute for Nature Research, Sluppen, PO Box 5685, 7485 Trondheim, Norway

**Keywords:** Behavioural ecology, Ecology

## Abstract

Previous experimental studies suggest that the production of sound associated with expelling gas from an open swimbladder may play a role in communication. This would suggest non-random gas release. We used deployed echosounders to study patterns of gas release among a fjord population of sprat (*Sprattus sprattus*). The echosounder records concurrently revealed individual fish and their release of gas. The gas release primarily occurred at night, partly following recurrent temporal patterns, but also varying between nights. In testing for non-randomness, we formulated a data-driven simulation approach. Non-random gas release scaled with the length of the analyzed time intervals from 1 min to 6 h, and above 30 min the release events in more than 50% of the intervals were significantly connected.

## Introduction

The swimbladder of fishes has various functions. It facilitates buoyancy^1^, can play part in respiration^2^, acts as a sense organ^3^, and serves for sound production^1,4,5^. Fishes are characterized as physoclists or physostomous based on swimbladder morphology. The physoclist swimbladder is closed, with gas regulation via the blood system. The physostome swimbladder is open, and the bladder typically fills by gulping air at the surface. The open swim bladder enables rapid release of gas, expelled through either an anal duct or the esophagus^1,6^.

Clupeids are physostomous fishes^7^. The swimbladder wall of herring has a barrier of guanine crystals hampering diffusion rates and allowing for prolonged retention of gas^3^. Yet, clupeids release gas in unspecified patterns and for uncertain and debated reasons^6,8,9^.

The release of gas from the swimbladder produces sound^4,10^ and appears to be a behavioral, and not a physical response^10^. The sound produced by gas release in both Pacific (*Clupea pallacii*) and Atlantic herring (*Clupea harengus*) is characterized by distinctive bursts of pulses at unusual frequencies compared to other fish sounds^4,10^. Sound records do not appear to have been made for sprat (*Sprattus sprattus*), but based on their open canal from the swimbladder to the anal duct, Wahlberg and Westerberg^10^ suggested that sprat produced similar chirps as herring during gas release. Wilson et al.^4^ observed that sounds were temporally associated with the appearance of fine bubble streams from the anus or anal duct of individual fishes, which compares with echosounder records of gas release by sprat^9^.

Clupeids have particularly well-developed sound reception, suggesting that hearing is important^11^. The frequencies of sound produced during gas release are within the hearing capacity of herring^10,12^ and individual herring can probably hear the bubbles from nearby fishes^10^. Denton and Gray^13^ found that the ear of sprat was largely similar to that of herring, being a very sensitive sound pressure detector. Also Hawkins and Popper^14^ underline that clupeid fishes, including the sprat, are especially sensitive to sounds, and that hearing in sprat is likely to be similar to that of herring.

The functions of these sounds are unknown. Although it is feasible that sound production is incidental, Wilson et al.^4^ suggested that sound associated with gas release might have communication functions. Their reasoning found support in experimental findings of complex, pulsed sound patterns and greater per capita rates of sound production at higher fish densities in experimental settings where alternative explanations like pressure changes, buoyancy adjustment and responses to predators^6,8,15^ did not apply. Production of sound was nocturnal, and Wilson et al.^4^ hypothesized acoustic communication might allow fishes to keep contact in the darkness of night. In modeling the sound levels produced by bubble production of individual herring, Hahn and Thomas^16^ concluded that acoustic emission of herring would allow for sophisticated behavioral modes, such as communication and active modes of predator escape and avoidance. In experiments with Pacific herring, a single chirp heard from a few fish swimming in loose aggregation was accompanied by a brief pause by all individuals^17^. The captive schooling herring showed, however, no behavioral response to playbacks of such sounds during daytime^17^.

Solberg and Kaartvedt^9^ applied submerged, upward-facing echosounders to study sprat during overwintering in Oslofjorden, Norway. Gas bubbles are excellent acoustic targets and gas release became clearly depicted in the echograms. The sprat carried out rapid and short excursions to the surface, apparently to fill the swim bladder. Each fish surfaced an estimated 3–4 times daily, with ~ 70 daily burst of gas release per fish. Both types of events were nocturnal. Here, we analyze data from the field campaign of Solberg and Kaartvedt^9^ in more detail, using gas release as a proxy for sound production. The fjord froze over from January to April and we analyze data from ice-free conditions in early winter. Specifically, we tested the hypothesis of connected gas release and associated sound production among fishes, expecting that gas release would appear in non-random patterns if related to communication. We compare the observed patterns with model results for the outcome in the case of random releases. For this, we formulate a model assessing connectivity among gas bubble releases allowing for a formal test of non-randomness.

## Results

### Patterns of gas release

**Figure 1:**
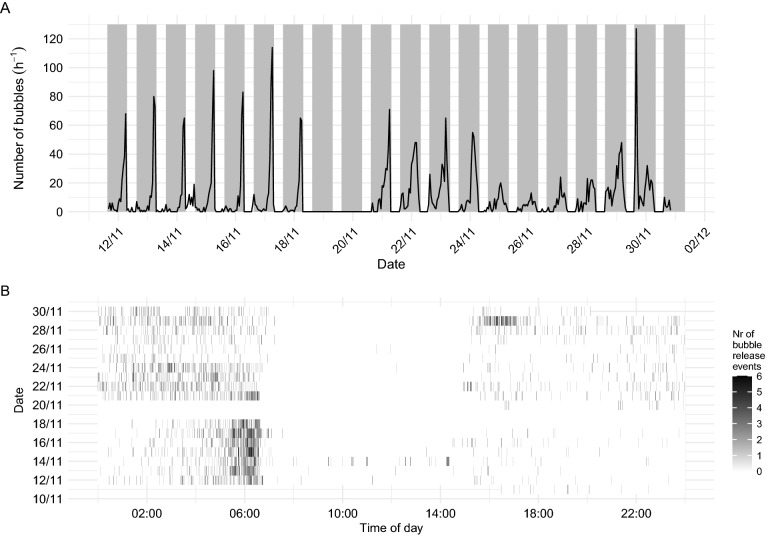
(**A**) Daily patterns of gas release, with shaded areas representing night. (**B**) Release events per minute over the course of a day. Data are not available on 19 and 20 November due to instrument failure.

**Figure 2:**
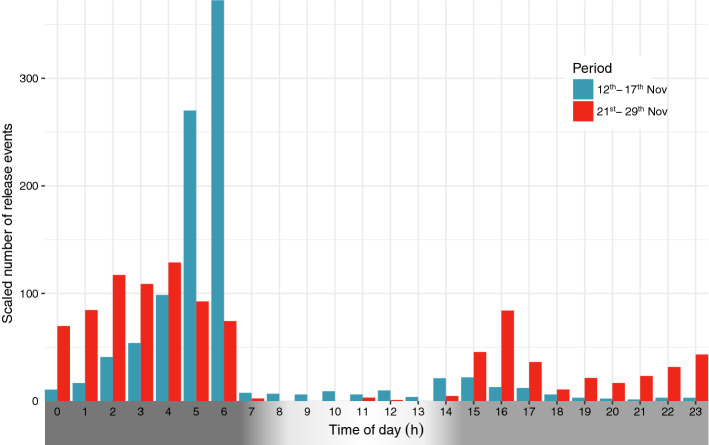
Diel patterns in gas release as represented by the hourly records averaged for two registration periods. Patterns of shading of x-axis refers to darkness and daylight.

The gas release had a strong non-random component with marked daily peaks, confined to the night (Fig. 1A,B). The nocturnal discharge of gas largely followed recurrent patterns, but also changed during the registration period. To illustrate the main patterns within days, we here combine results on hourly discharge from days 1–6 and 10–18, respectively (Fig. 2). During the first period, a minor initial pulse close to and after sunset (14:56–14:45 GMT) was followed by an exponential increase in gas discharge after mid-night, peaking 1–2 h prior to dawn (sunrise at 07:07–07:19 GMT during this first period). During the second part, the afternoon peak was larger, and the subsequent pattern more dome shaped, with an apparent slight decline in gas release towards dawn (Figs. 1, 2). However, such averaging between nights conceals some marked variations, including a strong peak just after sunset on 29 Nov, (day 18; ref Fig. 1), this day with particularly low release of gas bubbles at the end of the night.

**Figure 3:**
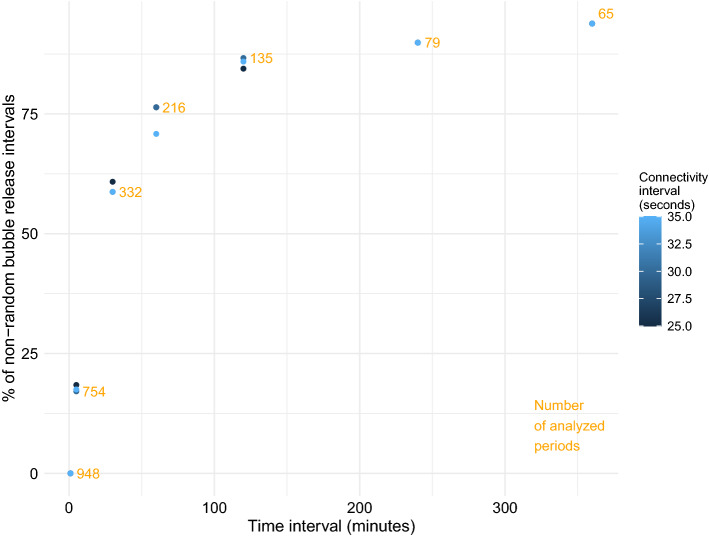
The percentage of the time intervals where the observed connectivity of the release events was determined to be non-random. Yellow numbers represent the number of analyzed time intervals with 2 or more release events for each interval length. The connectivity interval is the period within following gas releases were considered to be connected.

The test for connectivity among gas releases revealed increasing rejection of the null hypothesis of random release when increasing the time window from 1 min to 6 h (Fig. 3). With the S-CON test developed for this study (see “Methods”), the average rejection increased from 0% at 1-min intervals to 93.8% at 6-h intervals (assuming a connectivity interval of 30 s, Fig. 3). Furthermore, the connectivity was non-random at the 5% significance level in more than 50% of the windows equal to or longer than 30 min.

### Relationship between gas release and fish abundance

To exclude the possibility that apparent connectivity would be a mere result of fluctuating fish abundance, we tested whether the number of released bubbles is a function of fish biomass, as assessed by acoustic abundance estimates. The general least squares analysis revealed a significant relationship between gas release and fish abundance as measured by the surface integrated backscatter coefficient (F_1,420_ = 23.37, p < 0.001). However, fish abundance only explained approximately 12% of the variation in the release events.

## Discussion

The sprat primarily released gas at night, in accordance with previous experimental studies of Pacific herring^4^. Our records did not cover the lower part of the water column, yet nocturnal gas release was confirmed by simultaneous measurements encompassing the vertical range of the entire sprat population (see “Methods”). Most discharge events were significantly connected at any time scale exceeding 30 min (Fig. 3). We therefore reject the null hypothesis of random gas release and interpret the results as strong support of the alternative hypothesis of non-random release. Thus, this finding from this field study of sprat is in line with conclusions from the experimental studies of pacific herring^4^.

Rejection rates for the null hypothesis become less, the smaller the considered time window becomes. Within short time windows, even purely random points will tend to form peaks because of lack of space. Consequently, the statistical tests at this time scale will only be able to separate highly dependent short-sequenced release events from a random process. Nevertheless, even among the 754 5-min intervals which had more than one release event, 17% appeared with dependency between the gas bubble releases. This is 3.6 times more than expected at the 5% level if all the 5-min intervals contained purely random release.

There is predation risk associated with filling the swim bladder at the surface, manifested in that the sprat only surface for refills at night^9^. Continued gas release requires repeated access to surface air each night^4,9^, thus apparently increasing mortality risk. A particular urge to refill the swim bladder appeared when the study site froze over later in the same winter. The frequency of surfacing increased markedly after the fjord became ice covered, with apparent frantic, yet unsuccessful search for air under the ice as the records of gas release decreased sharply^9^. Evidently, gas is a valuable resource. This inference is underlined by the swim bladder of herring being equipped with guanine crystals, likely to hamper leakage of gas^3^.

Sprat is negatively buoyant at depth, manifested by a continuous sink-and-rise swimming^18^. Although fat plays a significant role for the buoyancy of clupeids^7^, the deliberate release of gas probably involves a metabolic cost for the negatively buoyant sprat. Other costs are conceivable, given the multitude of swimbladder functions^1–4^. Nevertheless, the sprat consistently released gas in spite of the costs, signifying there is a gain in gas release.

The pulsed release patterns suggest that release was either stimulated by common external factors, or that the gas release—considered here as a proxy for sound production of the fishes—stimulated each other. Solberg and Kaartvedt^9^ could not identify any external potential trigger for the gas release, but we cannot exclude the possibility of individuals reacting to an external stimulus that remains to be identified. The sprat in the Oslofjord exhibit diverse DVM patterns, including “mid-night sinking” and an intermittent dawn ascent prior to sunrise^18,19^. During the current study, dawn ascent was apparent the first weeks of November. The timing of gas release suggests some association with this event (cf. Fig. 1). However, the sprat also released gas at other times of night. The nocturnal distribution became shallower by the end of November^9^. The sprat then released gas in a nonrandom fashion throughout the night (Fig. 1). While fish abundance expectedly would affect the number of gas release events, it could only explain 12% of the variation in the data so that fluctuating fish abundance in the acoustic beam did not explain the non-random gas releases observed in our data.

Reciprocal stimulation of gas release would be in accordance with the experimental studies by Wilson et al.^4^. A filled swim bladder holds potential for producing sound, which Wilson et al.^4^ suggested herring might use for keeping contact in darkness. More than 800 fish species are known to produce sound^20^ and mounting evidence suggests that (intraspecific) acoustic communication can affect their success in various ways^21,22^. However, knowledge about the ecology of sound communication is still very limited^23^. The observed non-random connectivity of gas release—i.e. sound production—recorded here concords with the hypothesis of communication purposes. Further research is needed to provide first hand evidence of acoustic communication through gas release.

## Methods

### Study area

The study was carried out in Bunnefjorden, Norway. The fjord froze over from January to April and we here analyze data from ice-free conditions in early winter (12 Nov to 2 Dec 2009). Bunnefjorden is a 150 m deep inner branch of the Oslofjord, and a 57 m deep sill at the entrance restricts water exchange with the outer part of the fjord. Klevjer and Kaartvedt^24^ provide a map of the study area. The fjord branch normally becomes hypoxic in the lower part of the water column. During the current study, oxygen contents were 2–3 ml l^−1^ between 15 and 60 m, while waters below 70–80 m were severely hypoxic and devoid of fishes^9^.

Studies of overwintering sprat have been undertaken in Bunnefjorden during several winters, and the biology of sprat as well as the identity of the main acoustic targets in the fjord are well established^9,18,24^. In the winter of the current study, catches from 33 trawl samples were dominated by sprat; with ~ 40 times higher catches than the next most abundant species, herring (*Clupea harengus*)^9^.

### Study design

Solberg and Kaartvedt^9^ and Solberg et al.^18^ provide details on methods, and we here only give a summary of the acoustic setup. In short, upward-looking Simrad EK 60 echosounders kept in pressure-proof casings were deployed at the bottom (150 m) and in buoys (80 and 30 m) for enhanced resolution in shallower part of the water column. Cables for electricity and transfer of data to a PC on shore enabled continuous operation of the systems. We here use the data from the shallowest echosounder (200 kHz) that provided superior resolution in near-surface water, though did not cover the full depth range of the population distribution. Echograms from the deeper located echosounders covering the whole (inhabited) water column and showing the full diel population behavior are given in Solberg and Kaartvedt^9^ and Solberg et al.^18^.

### Records of gas release

**Figure 4:**
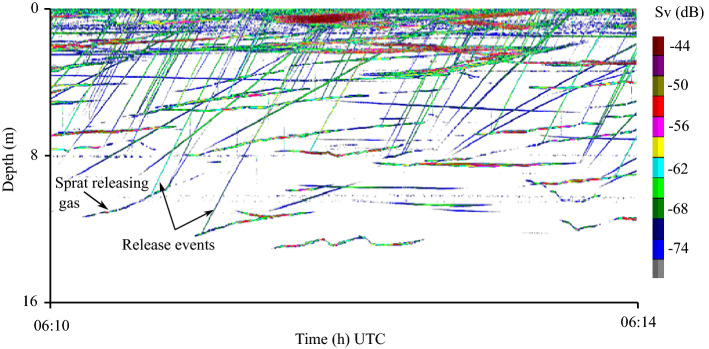
Echogram showing sprat releasing gas, with every oblique line representing one release event and lines with a different angle to the release events representing swimming sprat. Colors represent the volume-backscatter coefficient (Sv).

Released gas appeared as ascending lines in the echogram (Fig. 4). We quantified the release as explained by Solberg and Kaartvedt^9^. We only included ascending traces connected to the acoustic record of a fish, but without enumerating the release per individual fish. Since the same fish may release several bursts of bubbles within a short time interval, we here pooled any sequences of gas release within a 10-s period as one event. This procedure will also exclude cases with several different individuals releasing bubbles in the course of this short time interval, yet we chose this conservative approach not to generate an artificial high connection of gas releases between the fishes.

### Analyses of data

**Figure 5:**
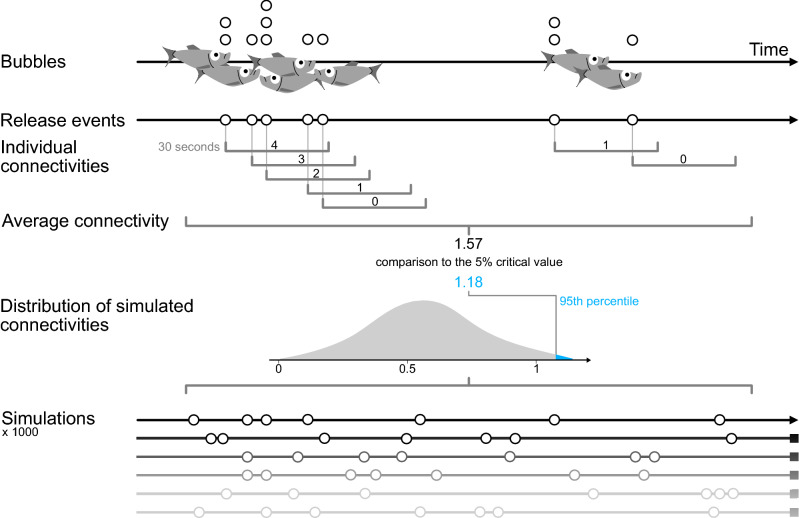
Illustration of the steps related to the simulated connectivity test (S-CON test). Bubbles occurring within 10 s are pooled into single release events. We then determine the connectivity of each release event—aka the number of release events within the following 30 s time window. From these, we calculate the average connectivity for a specified period (1, 5, 30 min, 1-, 2-, 4- and 6-h intervals). In a final step, we compare the observed average connectivity to the critical value which is defined as the 95th percentile of 1000 simulations of random placements of the same number of releases. If the observed connectivity is larger than the critical value, we reject the null hypothesis of random gas releases for the specified time interval.

The frequency of gas releases varied with time, both within a day and between the weeks. Such patterns compare to service systems like call centres and hospital emergency rooms^25^ that can be modelled as a Poisson process^26,27^. We therefore started our analysis with the statistical procedure suggested by Brown et al.^28^ in their influential analysis of the call dynamics in a banking call centre. The first step is to subdivide the day into time intervals, which are short enough to consider event rates as approximately constant. Here we chose to investigate alternative periods of respectively 1, 5, and 30 min, as well as 1, 2, 4, and 6 h. At the longest interval, the peaks in the gas release intensity are expected to be the result of a non-constant Poisson parameter, and therefore more likely to induce rejection of the null-hypothesis of a homogenous random process. In contrast, we expect to find higher concordance with a random process for the short intervals of 5 min. In assessing connectivity among gas bubble releases, we formulate a new model allowing for a formal test of non-randomness (summarized in Fig. 5). We name this approach the simulated connectivity test (S-CON test), which we implemented in R^29^, with the code being available in the Supplementary appendix.

If there is a common physiological reason or some form of communication among sprat, a burst of gas release is likely followed by subsequent releases. Thus, it is reasonable to assume that the total number of releases within a short time interval like 30 s would be effective in detecting dependencies between the releases. We therefore define the concept of connectivity as follows:

Let the gas be released at times T_1_, T_2_, …, T_n_ and define the connectivity at each event as the numbers of records within the following 30 s. The average connectivity in any considered window of the investigated time-period (for example a window of 1 h) is defined as the average connectivity of all cases of connectivity within the considered window (see also Fig. 5).

In order to test the null hypothesis of no dependency between gas bubble releases, we compare the measured average connectivity in the data set with the simulation of 1000 random placements of the total number of observations in a given time window. For example, if we consider a window of 30 min with 15 release events having an average connectivity of 2.1, we performed 1000 random placements of 15 points between 1 and 1800. In this way, we get 1000 simulated values of the average connectivity, from which we pick out the critical 95th percentile, following the common significance level of 0.05 in biology. If the observed average connectivity is larger than this critical value, we reject the null-hypothesis and conclude that the releases of gas bubbles are dependent random variables. Thus, if our example obtains a critical value of 1.7, the null-hypothesis of random arrival times of bubbles is rejected (because the observed value of 2.1 is larger than the critical value of 1.7).

Since a dependency between the fish will induce a higher concentration of release events than produced by random releases, we expect the average connectivity to be quite sensitive to the alternative hypothesis of dependent arrival times. Also, note that the concept of connectivity has a combinatory nature, so we need only require that the considered window contains at least two releases of gas bubbles. In contrast, alternative approaches using Kolmogorov–Smirnov tests^28^ are based on the cumulative distribution function and therefore require at least five observed bubble releases.

To test the dependency of the results on the chosen time interval, we also ran the analysis using connectivity intervals of 25 and 35 s, which revealed some variability to the estimates of non-random bubble release (Fig. 3) but did not influence the general pattern. We also tested whether the interval within which we consider subsequent bubbles to be part of one single release event influences our results. The more we consider sequential bubbles to be independent of each other, i.e. their own release event, the higher the proportion of non-random gas release and vice versa.

### Fish abundance

To exclude the possibility that apparent connectivity would be a mere result of fluctuating fish abundance, we tested whether the number of released bubbles is a function of fish biomass. For this, we first calculated the total number of gas release events within 30-min periods. We then compared these values to the summed surface integrated acoustic scattering coefficient (SA) for the same periods and for the same depth interval (upper 30 m), assuming that the integrated scattering coefficient (SA) serves as a proxy for the total fish biomass^9^. We filtered the scattering data to remove noise from non-biological sources prior to use. Both variables were log-transformed prior to analysis. We then fitted a linear model of the two variables using generalized least squares. To account for temporal autocorrelation in the data, we also included a correlation structure of type 1 (corAR1). The analysis was done in R^29^ using the nlme package^30^.

### Ethics declarations

Live animals (fish) were not used in this study.

## Supplementary Information

Supplementary Information.

## Data Availability

The scripts for the S-CON analysis is available in the Supplementary appendix. The raw echo sounder data are available from the corresponding author upon request.
