# Supplementary material for: Coordinated gas release among the physostomous fish sprat (Sprattus sprattus)
Source: Sci Rep. 2021 Jun 23;11:13145. doi: 10.1038/s41598-021-92585-2 (PMC8222301; doi:10.1038/s41598-021-92585-2)
Supplement: Supplementary file 1 — Supplementary Information. [file 41598_2021_92585_MOESM1_ESM.pdf]

Date (DDMMYYY),Clock (hh:mm:ss),Depth m (from transducer),Depth,DepthRank,nBubD

11/11/2009,16:29:58,24.61,5.39,5.4,1

11/11/2009,16:29:59,24.46,5.54,5.5,1

11/11/2009,16:30:03,25.75,4.25,4.3,1

11/11/2009,16:30:05,25.87,4.13,4.1,1

11/11/2009,16:30:07,25.45,4.55,4.6,1

11/11/2009,16:30:08,25.14,4.86,4.9,1

11/11/2009,17:00:59,21.18,8.82,8.8,1

11/11/2009,17:01:04,20.83,9.17,9.2,1

11/11/2009,17:01:07,20.85,9.15,9.2,1

11/11/2009,17:14:42,25.77,4.23,4.2,1

11/11/2009,17:40:48,25.92,4.08,4.1,1

11/11/2009,17:40:49,25.85,4.15,4.2,1

11/11/2009,17:41:11,25.2,4.8,4.8,1

11/11/2009,17:49:02,24.4,5.6,5.6,1

11/11/2009,18:40:53,25.19,4.81,4.8,1

11/11/2009,19:13:27,20.62,9.38,9.4,1

11/11/2009,19:13:34,20.58,9.42,9.4,1

11/11/2009,19:13:35,20.44,9.56,9.6,1

11/11/2009,19:13:46,20.44,9.56,9.6,1

11/11/2009,19:13:50,20.62,9.38,9.4,1

11/11/2009,19:14:57,22.47,7.53,7.5,1

11/11/2009,19:36:06,23.56,6.44,6.4,1

11/11/2009,19:36:07,23.14,6.86,6.9,1

11/11/2009,20:05:44,23.98,6.02,6.0,1

11/11/2009,20:05:45,23.7,6.3,6.3,1

11/11/2009,20:06:09,24.54,5.46,5.5,1

11/11/2009,21:25:09,16.87,13.13,13.1,1

11/11/2009,22:30:08,23.77,6.23,6.2,1

11/11/2009,22:30:09,23.49,6.51,6.5,1

12/11/2009,00:28:53,22.23,7.77,7.8,2

12/11/2009,00:28:53,21.77,8.23,8.2,1

12/11/2009,00:29:27,23.38,6.62,6.6,1

12/11/2009,00:29:28,23.17,6.83,6.8,1

12/11/2009,00:38:04,17.75,12.25,12.3,1

12/11/2009,00:38:06,17.43,12.57,12.6,1

12/11/2009,00:38:15,17.61,12.39,12.4,1

12/11/2009,00:38:22,16.87,13.13,13.1,1

12/11/2009,00:45:10,20.86,9.14,9.1,1

12/11/2009,01:05:14,25.45,4.55,4.6,1

12/11/2009,01:05:17,17.96,12.04,12.0,1

12/11/2009,01:05:20,17.85,12.15,12.2,2

12/11/2009,01:05:23,18.17,11.83,11.8,1

12/11/2009,01:05:23,18.1,11.9,11.9,1

12/11/2009,01:05:25,18.03,11.97,12.0,1

12/11/2009,01:18:29,16.91,13.09,13.1,1

12/11/2009,01:18:30,16.63,13.37,13.4,1

12/11/2009,01:35:36,24.68,5.32,5.3,1

12/11/2009,01:35:39,24.64,5.36,5.4,1

12/11/2009,01:35:46,25.1,4.9,4.9,1

12/11/2009,01:44:39,11.48,18.52,18.5,1

12/11/2009,01:45:26,24.47,5.53,5.5,1

12/11/2009,01:50:00,24.12,5.88,5.9,1

12/11/2009,01:50:05,24.22,5.78,5.8,1

12/11/2009,01:50:06,24.33,5.67,5.7,1

12/11/2009,02:01:24,18.62,11.38,11.4,1

12/11/2009,02:32:31,23.63,6.37,6.4,1

12/11/2009,02:41:42,22.89,7.11,7.1,1

12/11/2009,02:57:13,19.57,10.43,10.4,1

12/11/2009,02:57:20,19.78,10.22,10.2,1

12/11/2009,02:58:04,21.49,8.51,8.5,1

12/11/2009,02:58:08,21.53,8.47,8.5,1

12/11/2009,02:58:25,22.86,7.14,7.1,1

12/11/2009,02:58:27,22.79,7.21,7.2,1

12/11/2009,03:04:15,17.78,12.22,12.2,1

12/11/2009,03:22:25,17.43,12.57,12.3,1

12/11/2009,03:22:19,17.68,12.32,12.6,1

12/11/2009,03:26:31,15.05,14.95,5.3,1

12/11/2009,03:25:12,24.68,5.32,5.3,1

12/11/2009,03:25:14,24.75,5.25,15.0,1

12/11/2009,03:27:33,24.36,5.64,5.6,1

12/11/2009,03:27:46,16.94,13.06,13.1,1

12/11/2009,03:27:57,17.29,12.71,12.7,1

12/11/2009,03:28:02,17.29,12.71,12.7,1

12/11/2009,03:28:43,18.8,11.2,11.2,1

12/11/2009,03:29:46,20.02,9.98,10.0,1

12/11/2009,03:30:34,15.05,14.95,15.0,1

12/11/2009,03:30:36,15.16,14.84,14.8,1

12/11/2009,03:32:55,17.96,12.04,12.0,1

12/11/2009,03:42:02,21.18,8.82,8.8,1

12/11/2009,03:42:04,21.25,8.75,8.8,1

12/11/2009,03:49:09,24.43,5.57,5.7,2

12/11/2009,03:49:58,24.64,5.36,5.6,1

12/11/2009,03:50:02,24.64,5.36,5.2,1

12/11/2009,03:49:08,24.26,5.74,5.4,1

12/11/2009,03:49:58,24.82,5.18,5.4,1

12/11/2009,03:50:03,24.82,5.18,5.2,1

12/11/2009,03:50:48,24.36,5.64,5.6,1

12/11/2009,03:50:50,24.33,5.67,5.7,2

12/11/2009,03:52:04,18.2,11.8,11.8,1

12/11/2009,03:52:07,18.27,11.73,11.7,1

12/11/2009,03:52:16,21.91,8.09,8.1,1

12/11/2009,03:52:16,21.32,8.68,8.7,1

12/11/2009,03:55:20,22.54,7.46,7.5,1

12/11/2009,04:09:46,23.84,6.16,6.2,1

12/11/2009,04:13:40,23,7,7.0,1

12/11/2009,04:13:44,23.03,6.97,7.0,1

12/11/2009,04:16:03,21.14,8.86,8.9,1

12/11/2009,04:22:24,24.71,5.29,10.6,1

12/11/2009,04:22:26,24.78,5.22,5.3,1

12/11/2009,04:21:13,19.43,10.57,5.2,1

12/11/2009,04:24:23,21,9,9.0,1

12/11/2009,04:24:49,22.33,7.67,7.7,1

12/11/2009,04:24:53,22.26,7.74,7.7,1

12/11/2009,04:25:14,24.78,5.22,5.2,1

12/11/2009,04:28:38,20.34,9.66,9.7,1

12/11/2009,04:28:39,20.41,9.59,9.6,1

12/11/2009,04:29:21,13.83,16.17,16.2,1

12/11/2009,04:30:14,23.94,6.06,16.2,1

12/11/2009,04:30:16,23.91,6.09,16.0,1

12/11/2009,04:30:26,24.47,5.53,6.1,1

12/11/2009,04:32:28,23.35,6.65,6.1,1

12/11/2009,04:29:25,14.04,15.96,5.5,1

12/11/2009,04:29:22,13.76,16.24,6.7,1

12/11/2009,04:43:38,18.52,11.48,11.5,3

12/11/2009,04:43:42,18.76,11.24,11.2,1

12/11/2009,04:43:43,18.76,11.24,11.2,1

12/11/2009,04:43:48,19.18,10.82,10.8,1

12/11/2009,04:44:37,21.7,8.3,7.9,1

12/11/2009,04:44:37,21.88,8.12,8.1,1

12/11/2009,04:44:37,22.09,7.91,8.3,1

12/11/2009,04:44:46,22.16,7.84,7.8,1

12/11/2009,04:45:31,18.83,11.17,10.5,1

12/11/2009,04:45:25,19.5,10.5,11.2,1

12/11/2009,04:49:04,23.28,6.72,6.7,1

12/11/2009,04:49:05,23.1,6.9,6.9,1

12/11/2009,04:49:07,23.1,6.9,6.9,1

12/11/2009,04:49:25,25.76,4.24,4.2,1

12/11/2009,04:49:27,26.01,3.99,4.0,1

12/11/2009,04:49:30,26.01,3.99,4.0,1

12/11/2009,04:50:50,16.24,13.76,13.8,1

12/11/2009,04:51:01,16.84,13.16,13.2,1

12/11/2009,04:51:04,16.87,13.13,13.1,1

12/11/2009,04:53:16,23.38,6.62,6.6,1

12/11/2009,04:53:18,23.59,6.41,6.4,1

12/11/2009,04:55:35,18.45,11.55,11.6,1

12/11/2009,04:55:39,18.52,11.48,11.5,1

12/11/2009,04:56:37,23.03,6.97,7.0,1

12/11/2009,04:56:38,23.17,6.83,6.8,1

12/11/2009,04:56:42,23.45,6.55,6.6,1

12/11/2009,04:56:43,23.42,6.58,6.6,1

12/11/2009,04:56:46,23.49,6.51,6.5,1

12/11/2009,04:56:50,23.7,6.3,6.3,1

12/11/2009,04:56:52,23.8,6.2,6.2,1

12/11/2009,04:56:56,24.01,5.99,6.0,1

12/11/2009,04:59:30,16.1,13.9,13.9,1

12/11/2009,04:59:32,16.1,13.9,13.9,1

12/11/2009,05:01:39,22.09,7.91,7.9,1

12/11/2009,05:02:58,22.79,7.21,7.2,1

12/11/2009,05:03:00,22.4,7.6,7.6,1

12/11/2009,05:03:46,22.44,7.56,7.6,1

12/11/2009,05:05:05,21.35,8.65,8.7,1

12/11/2009,05:05:08,21.35,8.65,8.7,1

12/11/2009,05:08:33,18.62,11.38,11.4,1

12/11/2009,05:08:34,18.62,11.38,11.4,1

12/11/2009,05:10:42,11.83,18.17,18.2,1

12/11/2009,05:10:46,12.04,17.96,18.0,1

12/11/2009,05:10:48,12.18,17.82,17.8,1

12/11/2009,05:11:00,12.88,17.12,17.1,1

12/11/2009,05:14:24,21.28,8.72,8.7,1

12/11/2009,05:15:22,22.23,7.77,7.8,1

12/11/2009,05:15:24,22.16,7.84,7.8,1

12/11/2009,05:16:36,20.9,9.1,9.1,1

12/11/2009,05:19:32,24.29,5.71,5.7,1

12/11/2009,05:19:35,24.4,5.6,5.6,2

12/11/2009,05:24:56,21.07,8.93,8.9,1

12/11/2009,05:25:01,21.21,8.79,8.8,1

12/11/2009,05:25:09,21.53,8.47,8.5,1

12/11/2009,05:25:19,22.3,7.7,7.7,1

12/11/2009,05:25:20,22.16,7.84,7.8,2

12/11/2009,05:29:40,21.18,8.82,8.8,1

12/11/2009,05:29:40,20.79,9.21,9.2,1

12/11/2009,05:33:38,19.95,10.05,10.1,1

12/11/2009,05:33:46,20.58,9.42,9.4,1

12/11/2009,05:33:56,22.19,7.81,7.8,1

12/11/2009,05:33:57,22.16,7.84,7.8,1

12/11/2009,05:33:58,22.12,7.88,7.9,1

12/11/2009,05:33:59,21.81,8.19,8.2,1

12/11/2009,05:33:58,21.81,8.19,8.2,1

12/11/2009,05:36:19,16.17,13.83,13.8,1

12/11/2009,05:36:22,16.1,13.9,13.9,1

12/11/2009,05:43:53,13.13,16.87,16.9,1

12/11/2009,05:46:42,24.99,5.01,14.3,2

12/11/2009,05:46:53,25.03,4.97,5.0,1

12/11/2009,05:45:29,15.72,14.28,5.0,1

12/11/2009,05:48:25,21.14,8.86,5.0,1

12/11/2009,05:48:28,21.25,8.75,9.1,1

12/11/2009,05:48:10,20.86,9.14,8.9,1

12/11/2009,05:48:00,25.03,4.97,8.8,1

12/11/2009,05:50:17,15.68,14.32,14.3,1

12/11/2009,05:51:12,23.21,6.79,6.8,1

12/11/2009,05:51:12,22.96,7.04,7.0,2

12/11/2009,05:51:15,23.03,6.97,7.0,1

12/11/2009,05:52:29,11.73,18.27,18.3,1

12/11/2009,05:56:46,16.1,13.9,13.9,1

12/11/2009,05:57:23,17.08,12.92,12.9,1

12/11/2009,05:57:58,20.23,9.77,9.8,1

12/11/2009,05:58:09,20.51,9.49,9.5,1

12/11/2009,06:00:29,21.39,8.61,5.6,1

12/11/2009,06:00:42,23.07,6.93,5.3,1

12/11/2009,06:00:44,23.1,6.9,18.1,1

12/11/2009,06:00:47,23.14,6.86,18.3,1

12/11/2009,05:59:48,24.78,5.22,5.2,1

12/11/2009,05:59:13,24.75,5.25,8.6,1

12/11/2009,05:59:10,24.43,5.57,6.9,1

12/11/2009,05:59:17,11.87,18.13,6.9,1

12/11/2009,05:59:17,11.66,18.34,6.9,1

12/11/2009,06:05:48,16.87,13.13,13.1,1

12/11/2009,06:05:49,16.7,13.3,13.3,1

12/11/2009,06:05:51,16.84,13.16,13.2,2

12/11/2009,06:05:53,16.87,13.13,13.1,1

12/11/2009,06:05:56,17.01,12.99,13.0,1

12/11/2009,06:05:57,17.05,12.95,13.0,1

12/11/2009,06:06:01,17.36,12.64,12.6,1

12/11/2009,06:06:32,20.02,9.98,10.0,1

12/11/2009,06:07:14,21.91,8.09,8.1,1

12/11/2009,06:07:17,21.88,8.12,8.1,1

12/11/2009,06:08:52,23.52,6.48,6.5,1

12/11/2009,06:09:34,24.61,5.39,5.4,1

12/11/2009,06:10:13,22.05,7.95,8.0,1

12/11/2009,06:10:19,21.84,8.16,8.0,1

12/11/2009,06:10:35,19.18,10.82,8.2,1

12/11/2009,06:10:13,21.98,8.02,10.8,1

12/11/2009,06:11:41,22.86,7.14,12.7,1

12/11/2009,06:11:39,22.75,7.25,12.6,2

12/11/2009,06:11:38,22.75,7.25,7.3,1

12/11/2009,06:11:19,17.36,12.64,7.3,1

12/11/2009,06:11:18,17.26,12.74,7.1,1

12/11/2009,06:11:56,20.55,9.45,9.5,1

12/11/2009,06:11:58,20.65,9.35,12.0,1

12/11/2009,06:12:12,18.27,11.73,9.4,1

12/11/2009,06:12:32,20.06,9.94,11.7,1

12/11/2009,06:12:34,20.2,9.8,9.9,1

12/11/2009,06:12:49,14.98,15.02,9.8,1

12/11/2009,06:12:56,15.09,14.91,15.0,1

12/11/2009,06:11:57,17.99,12.01,14.9,1

12/11/2009,06:12:56,14.91,15.09,15.1,1

12/11/2009,06:14:13,23.35,6.65,6.7,1

12/11/2009,06:14:19,23.49,6.51,6.5,1

12/11/2009,06:14:23,23.84,6.16,6.2,1

12/11/2009,06:15:36,20.02,9.98,6.1,1

12/11/2009,06:15:38,19.85,10.15,6.2,1

12/11/2009,06:15:33,22.82,7.18,6.1,1

12/11/2009,06:15:13,24.01,5.99,6.0,1

12/11/2009,06:15:12,23.98,6.02,6.0,1

12/11/2009,06:15:02,23.91,6.09,7.2,1

12/11/2009,06:15:01,23.8,6.2,10.0,1

12/11/2009,06:14:57,23.91,6.09,10.2,1

12/11/2009,06:15:56,23.35,6.65,6.7,1

12/11/2009,06:16:33,21.7,8.3,8.3,1

12/11/2009,06:16:35,21.7,8.3,8.3,1

12/11/2009,06:16:38,22.19,7.81,7.8,1

12/11/2009,06:16:42,22.75,7.25,7.3,1

12/11/2009,06:17:19,23.14,6.86,6.9,1

12/11/2009,06:17:46,24.89,5.11,15.3,1

12/11/2009,06:17:47,24.64,5.36,5.1,1

12/11/2009,06:17:48,24.5,5.5,5.4,1

12/11/2009,06:17:39,14.74,15.26,5.5,1

12/11/2009,06:18:18,21,9,9.0,1

12/11/2009,06:18:22,21.11,8.89,8.9,1

12/11/2009,06:18:44,22.65,7.35,7.4,1

12/11/2009,06:18:48,22.54,7.46,7.5,1

12/11/2009,06:21:58,24.82,5.18,5.2,1

12/11/2009,06:22:13,26.36,3.64,3.6,1

12/11/2009,06:22:56,18.2,11.8,11.8,1

12/11/2009,06:23:22,20.27,9.73,9.7,1

12/11/2009,06:23:26,20.48,9.52,9.5,1

12/11/2009,06:23:27,21.18,8.82,8.8,1

12/11/2009,06:24:11,22.54,7.46,7.5,1

12/11/2009,06:24:12,25.55,4.45,4.5,1

12/11/2009,06:25:09,18.62,11.38,11.4,1

12/11/2009,06:25:13,18.97,11.03,11.0,1

12/11/2009,06:26:45,20.79,9.21,9.2,1

12/11/2009,06:26:47,20.79,9.21,9.2,1

12/11/2009,06:26:50,20.83,9.17,9.2,1

12/11/2009,06:26:58,18.69,11.31,11.3,1

12/11/2009,06:27:22,22.37,7.63,7.6,1

12/11/2009,06:27:24,22.12,7.88,7.9,1

12/11/2009,06:27:49,20.27,9.73,9.7,1

12/11/2009,06:28:16,20.93,9.07,9.1,1

12/11/2009,06:29:26,17.15,12.85,12.9,1

12/11/2009,06:30:30,18.52,11.48,11.5,1

12/11/2009,06:30:33,18.69,11.31,11.3,1

12/11/2009,06:30:55,22.61,7.39,7.3,1

12/11/2009,06:30:54,22.75,7.25,7.4,1

12/11/2009,06:31:03,24.54,5.46,5.5,1

12/11/2009,06:31:12,19.88,10.12,10.1,1

12/11/2009,06:31:10,19.92,10.08,10.1,1

12/11/2009,06:31:31,21.6,8.4,8.4,1

12/11/2009,06:31:32,21.6,8.4,8.4,1

12/11/2009,06:31:35,21.6,8.4,8.4,1

12/11/2009,06:31:44,14.91,15.09,15.1,1

12/11/2009,06:31:45,14.91,15.09,15.1,2

12/11/2009,06:31:47,15.16,14.84,14.8,1

12/11/2009,06:32:45,19.32,10.68,10.7,1

12/11/2009,06:32:50,19.15,10.85,10.9,1

12/11/2009,06:35:21,19.57,10.43,6.1,1

12/11/2009,06:35:22,20.51,9.49,10.4,1

12/11/2009,06:35:09,23.91,6.09,9.5,1

12/11/2009,06:40:20,19.5,10.5,10.5,1

12/11/2009,06:40:22,19.64,10.36,10.4,1

12/11/2009,06:40:25,19.71,10.29,10.3,1

12/11/2009,06:40:51,23.84,6.16,6.2,1

12/11/2009,06:40:57,24.26,5.74,5.7,1

12/11/2009,06:41:02,24.78,5.22,5.2,2

12/11/2009,06:41:02,24.61,5.39,5.4,1

12/11/2009,06:41:06,24.96,5.04,5.0,1

12/11/2009,06:41:09,25.69,4.31,4.3,1

12/11/2009,06:43:41,20.55,9.45,7.7,1

12/11/2009,06:43:38,20.76,9.24,8.0,1

12/11/2009,06:44:13,22.86,7.14,9.2,1

12/11/2009,06:44:17,23.17,6.83,9.5,1

12/11/2009,06:44:19,23.21,6.79,7.1,1

12/11/2009,06:44:21,23.31,6.69,6.8,1

12/11/2009,06:44:23,23.31,6.69,6.8,1

12/11/2009,06:44:54,16.52,13.48,6.7,1

12/11/2009,06:44:54,16.91,13.09,6.7,2

12/11/2009,06:45:16,17.92,12.08,13.1,1

12/11/2009,06:45:17,17.78,12.22,13.5,1

12/11/2009,06:45:19,17.64,12.36,12.1,1

12/11/2009,06:43:19,22.33,7.67,12.2,1

12/11/2009,06:43:21,22.05,7.95,12.4,1

12/11/2009,07:22:17,21.35,8.65,8.7,1

12/11/2009,07:22:18,20.97,9.03,9.0,1

12/11/2009,07:22:19,20.41,9.59,9.6,1

12/11/2009,08:18:40,23.45,6.55,6.6,1

12/11/2009,08:18:42,23.63,6.37,6.4,1

12/11/2009,08:18:43,23.73,6.27,6.3,1

12/11/2009,08:19:05,23.63,6.37,6.4,1

12/11/2009,11:13:51,20.34,9.66,9.0,1

12/11/2009,11:13:52,20.37,9.63,9.1,1

12/11/2009,11:13:47,20.83,9.17,9.2,1

12/11/2009,11:13:47,20.9,9.1,9.7,1

12/11/2009,11:13:46,21,9,9.6,1

12/11/2009,11:14:02,20.44,9.56,9.9,1

12/11/2009,11:14:00,20.41,9.59,9.6,2

12/11/2009,11:13:59,20.13,9.87,9.6,1

12/11/2009,11:14:07,20.23,9.77,9.8,1

12/11/2009,15:24:04,24.78,5.22,5.2,1

12/11/2009,15:24:06,24.4,5.6,5.6,1

12/11/2009,15:24:10,24.47,5.53,5.5,2

12/11/2009,15:24:14,24.5,5.5,5.5,1

12/11/2009,15:24:16,24.08,5.92,5.9,1

12/11/2009,15:24:19,23.87,6.13,6.1,1

12/11/2009,15:27:41,24.01,5.99,6.0,1

12/11/2009,15:27:44,23.28,6.72,6.7,1

12/11/2009,15:30:45,24.22,5.78,4.6,1

12/11/2009,15:30:35,25.41,4.59,5.8,1

12/11/2009,15:38:28,26.22,3.78,3.8,1

12/11/2009,15:38:30,26.15,3.85,3.9,2

12/11/2009,15:38:36,26.01,3.99,3.8,1

12/11/2009,15:38:36,26.25,3.75,4.0,1

12/11/2009,16:06:58,25.87,4.13,4.1,1

12/11/2009,16:10:00,22.61,7.39,7.4,1

12/11/2009,17:21:15,25.97,4.03,4.0,1

12/11/2009,17:21:18,25.86,4.14,4.1,1

12/11/2009,17:36:52,17.22,12.78,12.8,1

12/11/2009,17:36:52,17.12,12.88,12.9,1

12/11/2009,17:36:58,17.29,12.71,12.7,1

12/11/2009,17:56:37,16.8,13.2,12.5,1

12/11/2009,17:56:36,17.54,12.46,13.2,1

12/11/2009,19:31:55,25.62,4.38,4.4,1

12/11/2009,19:31:58,25.8,4.2,4.2,1

12/11/2009,21:00:42,26.85,3.15,3.2,1

12/11/2009,23:20:04,9.52,20.48,20.5,1

12/11/2009,23:20:09,11.2,18.8,18.8,2

13/11/2009,00:06:28,14.58,15.42,15.3,1

13/11/2009,00:06:28,14.72,15.28,15.4,1

13/11/2009,00:41:28,11.85,18.15,18.2,1

13/11/2009,00:53:08,21.86,8.14,8.2,1

13/11/2009,00:52:59,21.82,8.18,8.1,1

13/11/2009,01:50:31,16.21,13.79,13.8,1

13/11/2009,02:11:29,21.34,8.66,8.1,1

13/11/2009,02:11:27,21.86,8.14,8.7,1

13/11/2009,02:27:53,20.26,9.74,9.7,1

13/11/2009,02:28:04,20.26,9.74,9.7,1

13/11/2009,02:31:10,15.8,14.2,14.2,2

13/11/2009,02:31:12,15.86,14.14,14.1,1

13/11/2009,02:31:19,16.25,13.75,13.8,1

13/11/2009,02:31:22,16.38,13.62,13.6,2

13/11/2009,02:37:34,10.39,19.61,19.6,1

13/11/2009,02:39:32,24.32,5.68,5.7,1

13/11/2009,02:39:34,24.35,5.65,5.7,1

13/11/2009,02:43:50,24.8,5.2,4.9,1

13/11/2009,02:43:50,25.15,4.85,5.2,1

13/11/2009,02:57:32,11.29,18.71,16.0,1

13/11/2009,02:57:31,11.36,18.64,4.2,1

13/11/2009,02:57:23,25.81,4.19,4.2,1

13/11/2009,02:57:27,25.77,4.23,18.6,1

13/11/2009,02:57:19,14.03,15.97,18.7,1

13/11/2009,03:00:58,16,14,14.0,1

13/11/2009,03:04:05,25.95,4.05,4.1,1

13/11/2009,03:25:22,23.94,6.06,6.1,1

13/11/2009,03:39:46,12.99,17.01,16.8,1

13/11/2009,03:39:44,13.2,16.8,17.0,1

13/11/2009,03:40:33,17.46,12.54,12.3,1

13/11/2009,03:40:33,17.7,12.3,12.5,1

13/11/2009,03:40:34,17.87,12.13,12.1,1

13/11/2009,03:40:36,17.98,12.02,12.0,1

13/11/2009,03:45:43,17.25,12.75,11.8,1

13/11/2009,03:45:39,18.05,11.95,12.0,1

13/11/2009,03:45:39,18.22,11.78,12.8,1

13/11/2009,03:46:22,19.57,10.43,10.4,1

13/11/2009,03:46:24,19.81,10.19,10.2,1

13/11/2009,03:46:28,20.3,9.7,9.7,1

13/11/2009,03:46:32,20.37,9.63,9.6,1

13/11/2009,03:49:00,25.05,4.95,5.0,1

13/11/2009,04:02:08,20.16,9.84,9.8,1

13/11/2009,04:02:02,20.16,9.84,9.8,1

13/11/2009,04:02:00,20.23,9.77,9.8,2

13/11/2009,04:06:50,20.54,9.46,9.5,1

13/11/2009,04:10:30,19.68,10.32,10.3,1

13/11/2009,04:10:37,21.79,8.21,10.7,1

13/11/2009,04:10:35,19.16,10.84,10.8,1

13/11/2009,04:10:34,19.29,10.71,8.2,1

13/11/2009,04:23:11,24.87,5.13,5.7,2

13/11/2009,04:23:07,24.28,5.72,5.1,1

13/11/2009,04:23:26,25.01,4.99,5.0,1

13/11/2009,04:23:28,25.08,4.92,4.9,1

13/11/2009,04:26:02,24.66,5.34,5.3,1

13/11/2009,04:26:20,14.69,15.31,15.3,1

13/11/2009,04:26:23,14.72,15.28,15.3,1

13/11/2009,04:26:27,15.17,14.83,14.8,1

13/11/2009,04:26:31,16.04,13.96,14.0,1

13/11/2009,04:26:33,15.69,14.31,14.3,2

13/11/2009,04:26:36,15.76,14.24,14.4,1

13/11/2009,04:26:35,15.59,14.41,14.2,1

13/11/2009,04:27:09,18.88,11.12,11.1,1

13/11/2009,04:32:03,23.97,6.03,6.0,1

13/11/2009,04:31:56,24.01,5.99,6.0,1

13/11/2009,04:38:55,6.55,23.45,22.4,1

13/11/2009,04:38:53,6.86,23.14,23.1,1

13/11/2009,04:38:53,7.58,22.42,23.5,1

13/11/2009,04:40:05,24.18,5.82,5.8,1

13/11/2009,04:42:45,19.19,10.81,10.8,1

13/11/2009,04:46:36,24.35,5.65,5.7,1

13/11/2009,04:47:11,24.25,5.75,5.8,1

13/11/2009,04:52:58,24.11,5.89,5.9,1

13/11/2009,04:59:41,22.86,7.14,5.9,1

13/11/2009,04:59:39,23,7,7.0,1

13/11/2009,04:52:58,24.08,5.92,7.1,1

13/11/2009,05:01:21,17.77,12.23,12.2,1

13/11/2009,05:07:34,23.8,6.2,6.2,2

13/11/2009,05:07:41,22.41,7.59,7.3,1

13/11/2009,05:07:40,22.66,7.34,7.6,1

13/11/2009,05:08:12,20.78,9.22,9.2,1

13/11/2009,05:08:06,20.78,9.22,9.2,1

13/11/2009,05:10:05,23.14,6.86,6.8,1

13/11/2009,05:10:05,23.21,6.79,6.9,1

13/11/2009,05:13:19,20.13,9.87,9.7,1

13/11/2009,05:13:16,20.3,9.7,9.9,1

13/11/2009,05:14:09,24.11,5.89,7.8,1

13/11/2009,05:13:33,22.2,7.8,5.9,1

13/11/2009,05:17:04,21.62,8.38,8.6,1

13/11/2009,05:17:02,21.44,8.56,8.4,1

13/11/2009,05:17:59,23.38,6.62,6.6,1

13/11/2009,05:23:58,20.3,9.7,9.3,1

13/11/2009,05:23:36,20.72,9.28,9.7,1

13/11/2009,05:25:08,22.17,7.83,7.8,1

13/11/2009,05:25:18,22.55,7.45,7.5,1

13/11/2009,05:25:22,22.83,7.17,7.2,1

13/11/2009,05:25:33,23.45,6.55,6.6,1

13/11/2009,05:26:58,19.09,10.91,3.9,1

13/11/2009,05:26:35,26.15,3.85,10.9,1

13/11/2009,05:28:01,25.67,4.33,4.3,1

13/11/2009,05:28:07,25.95,4.05,4.1,1

13/11/2009,05:28:10,26.12,3.88,3.9,1

13/11/2009,05:28:11,26.05,3.95,3.9,1

13/11/2009,05:28:10,26.12,3.88,4.0,1

13/11/2009,05:29:12,26.15,3.85,3.9,1

13/11/2009,05:30:03,22.41,7.59,7.6,1

13/11/2009,05:30:59,24.11,5.89,5.8,1

13/11/2009,05:30:56,24.21,5.79,5.9,1

13/11/2009,05:31:17,19.26,10.74,10.5,1

13/11/2009,05:31:14,19.47,10.53,10.7,2

13/11/2009,05:31:47,19.92,10.08,10.1,1

13/11/2009,05:32:33,22.2,7.8,8.2,1

13/11/2009,05:32:31,21.89,8.11,8.1,1

13/11/2009,05:32:50,21.89,8.11,7.8,1

13/11/2009,05:32:48,22.17,7.83,7.8,1

13/11/2009,05:32:29,21.82,8.18,8.1,1

13/11/2009,05:33:51,19.33,10.67,10.7,1

13/11/2009,05:34:26,21.62,8.38,8.4,1

13/11/2009,05:34:31,21.51,8.49,8.5,1

13/11/2009,05:34:34,21.65,8.35,8.4,1

13/11/2009,05:34:39,21.72,8.28,8.3,1

13/11/2009,05:34:49,23.45,6.55,6.4,1

13/11/2009,05:34:48,23.56,6.44,6.6,1

13/11/2009,05:35:02,23.69,6.31,6.3,1

13/11/2009,05:35:10,22.52,7.48,7.5,2

13/11/2009,05:35:12,22.59,7.41,7.4,1

13/11/2009,05:35:19,22.2,7.8,7.8,1

13/11/2009,05:35:26,22.41,7.59,7.6,2

13/11/2009,05:36:12,24.04,5.96,6.0,1

13/11/2009,05:36:28,24.01,5.99,6.0,1

13/11/2009,05:36:59,21.2,8.8,8.8,1

13/11/2009,05:37:10,17.01,12.99,13.0,1

13/11/2009,05:37:14,16.73,13.27,13.3,1

13/11/2009,05:37:46,23.49,6.51,6.5,1

13/11/2009,05:37:53,23.42,6.58,6.4,1

13/11/2009,05:37:53,23.56,6.44,6.6,1

13/11/2009,05:38:06,23.28,6.72,6.7,1

13/11/2009,05:38:23,14.24,15.76,15.8,1

13/11/2009,05:38:50,18.36,11.64,11.6,1

13/11/2009,05:39:12,19.12,10.88,10.9,1

13/11/2009,05:39:13,19.09,10.91,10.9,1

13/11/2009,05:39:18,19.64,10.36,10.4,1

13/11/2009,05:39:22,16.32,13.68,13.7,1

13/11/2009,05:39:20,16.32,13.68,13.7,1

13/11/2009,05:39:38,13.47,16.53,16.5,1

13/11/2009,05:39:47,14.44,15.56,15.6,1

13/11/2009,05:39:50,14.44,15.56,15.6,1

13/11/2009,05:40:05,25.67,4.33,4.3,1

13/11/2009,05:41:38,23.49,6.51,8.7,1

13/11/2009,05:41:49,25.08,4.92,6.5,1

13/11/2009,05:41:49,25.7,4.3,4.3,1

13/11/2009,05:41:36,21.34,8.66,4.9,1

13/11/2009,05:42:57,22.86,7.14,7.1,1

13/11/2009,05:43:07,21.69,8.31,8.3,2

13/11/2009,05:43:21,22.45,7.55,6.9,1

13/11/2009,05:43:21,23.07,6.93,7.6,1

13/11/2009,05:44:11,25.05,4.95,5.0,1

13/11/2009,05:44:21,26.09,3.91,3.9,1

13/11/2009,05:46:00,19.19,10.81,10.8,1

13/11/2009,05:46:02,19.12,10.88,10.9,1

13/11/2009,05:47:01,24.39,5.61,6.2,1

13/11/2009,05:47:05,24.28,5.72,5.6,1

13/11/2009,05:47:19,23.73,6.27,5.7,1

13/11/2009,05:47:24,23.8,6.2,6.3,1

13/11/2009,05:47:28,23.97,6.03,6.2,1

13/11/2009,05:47:32,23.9,6.1,6.0,1

13/11/2009,05:46:59,23.76,6.24,6.1,1

13/11/2009,05:47:59,24.11,5.89,5.9,1

13/11/2009,05:48:14,24.04,5.96,6.0,1

13/11/2009,05:48:17,24.21,5.79,5.8,1

13/11/2009,05:52:22,21.69,8.31,8.3,1

13/11/2009,05:53:46,16.59,13.41,13.4,1

13/11/2009,05:53:50,16.8,13.2,13.2,1

13/11/2009,05:53:53,17.63,12.37,12.4,1

13/11/2009,05:55:51,24.18,5.82,5.8,2

13/11/2009,05:55:57,24.94,5.06,5.1,1

13/11/2009,05:56:13,23.87,6.13,6.1,1

13/11/2009,05:56:42,26.09,3.91,3.9,1

13/11/2009,05:56:45,22.66,7.34,7.3,1

13/11/2009,05:57:10,25.63,4.37,4.4,1

13/11/2009,05:57:12,25.32,4.68,4.7,1

13/11/2009,05:57:17,25.57,4.43,4.4,1

13/11/2009,05:57:19,24.35,5.65,5.7,1

13/11/2009,05:57:17,24.35,5.65,5.7,1

13/11/2009,05:57:50,25.29,4.71,4.7,1

13/11/2009,05:58:29,24.94,5.06,4.9,1

13/11/2009,05:58:28,25.15,4.85,5.1,1

13/11/2009,05:59:04,20.26,9.74,9.7,1

13/11/2009,05:59:03,20.33,9.67,9.7,1

13/11/2009,05:59:00,20.26,9.74,9.7,1

13/11/2009,05:59:26,21.72,8.28,8.3,1

13/11/2009,05:59:28,21.82,8.18,9.8,1

13/11/2009,05:59:29,21.89,8.11,8.2,1

13/11/2009,05:59:32,21.93,8.07,8.1,1

13/11/2009,05:59:27,20.23,9.77,8.1,1

13/11/2009,05:59:55,14.89,15.11,15.0,1

13/11/2009,05:59:53,14.96,15.04,15.1,1

13/11/2009,06:00:46,16.35,13.65,13.9,1

13/11/2009,06:00:43,16.14,13.86,13.7,1

13/11/2009,06:01:33,24.7,5.3,5.3,1

13/11/2009,06:01:31,24.66,5.34,5.3,1

13/11/2009,06:02:02,21.65,8.35,8.4,1

13/11/2009,06:02:04,25.7,4.3,4.3,1

13/11/2009,06:02:20,25.74,4.26,4.3,1

13/11/2009,06:02:24,25.74,4.26,9.7,1

13/11/2009,06:02:22,20.58,9.42,9.4,1

13/11/2009,06:02:20,20.33,9.67,4.3,1

13/11/2009,06:02:49,23.42,6.58,6.6,1

13/11/2009,06:02:54,21.23,8.77,8.8,1

13/11/2009,06:03:01,21.17,8.83,8.8,1

13/11/2009,06:03:04,21.17,8.83,8.8,1

13/11/2009,06:03:12,19.19,10.81,10.8,1

13/11/2009,06:04:07,25.12,4.88,4.9,1

13/11/2009,06:04:20,25.53,4.47,4.5,2

13/11/2009,06:04:22,25.63,4.37,4.4,1

13/11/2009,06:05:53,26.12,3.88,3.9,1

13/11/2009,06:05:56,26.12,3.88,3.9,1

13/11/2009,06:06:18,23.07,6.93,6.9,1

13/11/2009,06:06:16,23.07,6.93,6.9,1

13/11/2009,06:06:31,23.04,6.96,7.0,1

13/11/2009,06:06:34,22.86,7.14,7.1,1

13/11/2009,06:06:55,23.24,6.76,6.8,1

13/11/2009,06:06:57,23.28,6.72,6.7,1

13/11/2009,06:07:26,24.87,5.13,5.1,1

13/11/2009,06:07:32,25.22,4.78,4.8,1

13/11/2009,06:07:34,25.36,4.64,4.6,1

13/11/2009,06:07:37,25.5,4.5,4.5,1

13/11/2009,06:07:45,25.36,4.64,4.6,1

13/11/2009,06:07:47,25.36,4.64,4.6,1

13/11/2009,06:07:49,25.15,4.85,4.9,1

13/11/2009,06:07:53,25.29,4.71,4.7,1

13/11/2009,06:07:57,25.15,4.85,8.6,1

13/11/2009,06:07:55,21.37,8.63,4.9,1

13/11/2009,06:08:06,21.41,8.59,8.6,1

13/11/2009,06:09:13,25.08,4.92,4.9,1

13/11/2009,06:09:14,25.36,4.64,4.6,1

13/11/2009,06:09:49,18.26,11.74,11.7,1

13/11/2009,06:10:29,18.53,11.47,6.7,1

13/11/2009,06:10:24,23.35,6.65,11.5,1

13/11/2009,06:10:39,23.76,6.24,6.2,1

13/11/2009,06:10:58,19.81,10.19,10.2,1

13/11/2009,06:11:03,19.92,10.08,10.1,1

13/11/2009,06:11:11,21.69,8.31,8.3,1

13/11/2009,06:11:15,21.93,8.07,8.1,1

13/11/2009,06:11:19,22.07,7.93,20.9,1

13/11/2009,06:11:21,22.17,7.83,20.9,1

13/11/2009,06:11:45,23.04,6.96,20.9,2

13/11/2009,06:12:16,21.89,8.11,7.9,1

13/11/2009,06:12:21,22.62,7.38,7.8,2

13/11/2009,06:11:18,9.14,20.86,7.0,1

13/11/2009,06:11:16,9.07,20.93,8.1,1

13/11/2009,06:11:15,9.14,20.86,7.4,2

13/11/2009,06:13:30,14.24,15.76,15.8,1

13/11/2009,06:13:41,14.79,15.21,15.2,1

13/11/2009,06:13:44,15.31,14.69,14.7,1

13/11/2009,06:13:55,13.13,16.87,16.9,1

13/11/2009,06:14:44,15.38,14.62,14.6,1

13/11/2009,06:15:10,19.33,10.67,10.7,1

13/11/2009,06:15:15,19.54,10.46,10.5,1

13/11/2009,06:15:25,21.13,8.87,8.9,1

13/11/2009,06:15:29,21.3,8.7,8.7,1

13/11/2009,06:15:47,20.44,9.56,9.6,1

13/11/2009,06:16:00,20.65,9.35,9.4,1

13/11/2009,06:16:29,21.37,8.63,8.6,1

13/11/2009,06:16:40,21.27,8.73,8.7,1

13/11/2009,06:17:43,21.1,8.9,8.9,1

13/11/2009,06:17:46,21.06,8.94,8.9,1

13/11/2009,06:18:02,21.79,8.21,8.2,1

13/11/2009,06:18:14,22.52,7.48,7.6,1

13/11/2009,06:18:12,22.45,7.55,7.5,1

13/11/2009,06:18:45,23.31,6.69,6.7,1

13/11/2009,06:19:12,23.35,6.65,10.0,1

13/11/2009,06:19:10,20.02,9.98,6.7,1

13/11/2009,06:19:31,22.69,7.31,7.3,1

13/11/2009,06:19:35,22.62,7.38,19.2,2

13/11/2009,06:19:34,10.84,19.16,7.4,1

13/11/2009,06:19:50,19.78,10.22,10.1,1

13/11/2009,06:19:50,19.85,10.15,10.2,1

13/11/2009,06:19:51,20.06,9.94,10.2,1

13/11/2009,06:19:46,19.88,10.12,9.9,1

13/11/2009,06:20:53,19.75,10.25,10.3,1

13/11/2009,06:20:53,17.46,12.54,12.5,1

13/11/2009,06:21:03,17.46,12.54,12.5,1

13/11/2009,06:22:00,22.2,7.8,7.6,1

13/11/2009,06:21:58,22.38,7.62,4.7,1

13/11/2009,06:22:00,25.29,4.71,7.8,1

13/11/2009,06:22:33,21.27,8.73,8.7,2

13/11/2009,06:22:36,21.23,8.77,8.8,1

13/11/2009,06:22:39,21.23,8.77,8.8,1

13/11/2009,06:22:59,21.62,8.38,8.4,2

13/11/2009,06:23:01,21.58,8.42,8.4,1

13/11/2009,06:23:22,22.14,7.86,7.9,1

13/11/2009,06:23:26,22.34,7.66,7.7,1

13/11/2009,06:23:39,22.17,7.83,7.8,1

13/11/2009,06:23:41,22.31,7.69,7.7,1

13/11/2009,06:23:49,22.27,7.73,7.7,1

13/11/2009,06:24:11,24.63,5.37,5.4,2

13/11/2009,06:24:14,24.56,5.44,5.4,1

13/11/2009,06:24:18,24.56,5.44,5.4,1

13/11/2009,06:24:36,24.84,5.16,5.2,2

13/11/2009,06:24:40,23.73,6.27,19.0,1

13/11/2009,06:25:09,25.63,4.37,6.3,1

13/11/2009,06:24:42,11.01,18.99,19.0,1

13/11/2009,06:24:39,10.98,19.02,4.4,1

13/11/2009,06:28:14,21.51,8.49,8.5,2

13/11/2009,06:28:21,23.8,6.2,6.2,1

13/11/2009,06:28:47,21.69,8.31,8.3,1

13/11/2009,06:28:50,21.79,8.21,8.2,1

13/11/2009,06:29:12,14.62,15.38,15.2,1

13/11/2009,06:29:12,14.79,15.21,15.4,1

13/11/2009,06:32:39,25.01,4.99,5.0,1

13/11/2009,06:32:45,25.15,4.85,4.9,1

13/11/2009,06:32:50,22.93,7.07,7.1,1

13/11/2009,06:32:59,22.38,7.62,7.5,1

13/11/2009,06:32:57,22.52,7.48,7.6,1

13/11/2009,06:35:29,18.15,11.85,11.9,1

13/11/2009,06:35:26,18.08,11.92,11.9,1

13/11/2009,06:35:25,18.08,11.92,11.9,1

13/11/2009,06:35:42,19.92,10.08,10.1,1

13/11/2009,06:37:55,25.74,4.26,4.3,1

13/11/2009,08:37:46,23.45,6.55,6.6,1

13/11/2009,08:37:47,23.28,6.72,6.7,1

13/11/2009,08:37:47,22.97,7.03,7.0,1

13/11/2009,12:10:50,12.82,17.18,17.8,1

13/11/2009,12:10:47,12.23,17.77,17.2,1

13/11/2009,14:32:01,24.73,5.27,4.6,1

13/11/2009,14:32:00,25.12,4.88,4.9,1

13/11/2009,14:32:00,25.39,4.61,5.3,1

13/11/2009,15:22:54,26.02,3.98,4.0,1

13/11/2009,15:42:57,21.75,8.25,8.4,1

13/11/2009,15:42:54,21.55,8.45,8.5,1

13/11/2009,15:42:52,21.55,8.45,8.5,1

13/11/2009,15:42:51,21.58,8.42,8.3,1

13/11/2009,15:57:29,23.87,6.13,6.1,1

13/11/2009,15:57:29,23.9,6.1,6.1,1

13/11/2009,15:57:32,23.14,6.86,6.9,1

13/11/2009,15:57:43,24.32,5.68,5.7,1

13/11/2009,17:59:23,24.49,5.51,5.4,2

13/11/2009,17:59:21,24.56,5.44,5.4,1

13/11/2009,17:59:21,24.56,5.44,5.5,1

13/11/2009,18:00:34,23.97,6.03,5.7,1

13/11/2009,18:00:33,24.32,5.68,6.0,1

13/11/2009,18:54:53,23.17,6.83,6.8,1

14/11/2009,01:04:43,22.31,7.69,7.7,1

14/11/2009,01:04:43,22.34,7.66,7.7,1

14/11/2009,01:47:30,20.24,9.76,9.8,1

14/11/2009,01:47:37,20.59,9.41,9.4,1

14/11/2009,01:47:38,20.52,9.48,9.5,1

14/11/2009,01:47:39,20.59,9.41,9.4,1

14/11/2009,01:47:41,20.59,9.41,9.4,1

14/11/2009,01:47:43,20.45,9.55,9.6,1

14/11/2009,02:04:08,23.8,6.2,6.2,1

14/11/2009,02:04:14,24.19,5.81,5.8,1

14/11/2009,02:04:15,24.01,5.99,6.0,1

14/11/2009,02:33:09,22.34,7.66,7.7,1

14/11/2009,03:02:18,15.07,14.93,14.7,1

14/11/2009,03:02:17,15.28,14.72,14.9,1

14/11/2009,03:03:24,18.67,11.33,11.3,1

14/11/2009,03:03:26,19.1,10.9,10.9,1

14/11/2009,03:04:24,10.69,19.31,19.3,1

14/11/2009,03:04:28,10.83,19.17,19.2,1

14/11/2009,03:27:46,21.45,8.55,8.3,1

14/11/2009,03:27:45,21.56,8.44,8.4,1

14/11/2009,03:27:44,21.66,8.34,8.6,1

14/11/2009,03:27:55,22.52,7.48,7.5,1

14/11/2009,03:27:57,22.34,7.66,7.7,3

14/11/2009,03:29:50,17.88,12.12,12.1,1

14/11/2009,03:29:53,17.78,12.22,12.2,1

14/11/2009,03:29:56,18.03,11.97,12.0,1

14/11/2009,03:30:13,19.92,10.08,10.1,1

14/11/2009,03:30:15,19.95,10.05,10.1,1

14/11/2009,03:39:49,14.21,15.79,15.8,1

14/11/2009,03:39:58,16.82,13.18,13.2,1

14/11/2009,03:40:10,21.27,8.73,8.7,1

14/11/2009,03:49:45,19.31,10.69,10.7,1

14/11/2009,04:00:39,19.1,10.9,10.9,1

14/11/2009,04:00:38,19.1,10.9,10.9,1

14/11/2009,04:01:17,11.65,18.35,18.1,1

14/11/2009,04:01:17,11.93,18.07,18.4,1

14/11/2009,04:08:41,22.84,7.16,7.2,1

14/11/2009,04:08:44,22.73,7.27,7.3,1

14/11/2009,04:09:09,23.8,6.2,6.2,1

14/11/2009,04:09:14,24.94,5.06,5.1,1

14/11/2009,04:24:36,17.42,12.58,12.6,1

14/11/2009,04:33:29,19.77,10.23,10.2,1

14/11/2009,04:41:54,15.43,14.57,14.6,1

14/11/2009,04:47:12,20.63,9.37,9.3,2

14/11/2009,04:47:11,20.7,9.3,9.4,1

14/11/2009,04:52:43,12.9,17.1,17.2,1

14/11/2009,04:52:41,12.79,17.21,17.1,1

14/11/2009,04:55:47,16.74,13.26,13.1,1

14/11/2009,04:55:46,16.89,13.11,13.3,1

14/11/2009,04:57:06,21.16,8.84,8.8,1

14/11/2009,05:01:41,16.85,13.15,13.0,2

14/11/2009,05:01:37,16.96,13.04,13.0,1

14/11/2009,05:01:35,16.99,13.01,13.2,1

14/11/2009,05:02:04,16.25,13.75,13.8,1

14/11/2009,05:03:24,16.5,13.5,13.4,1

14/11/2009,05:03:23,16.57,13.43,13.5,1

14/11/2009,05:03:29,18.74,11.26,11.0,1

14/11/2009,05:03:28,18.99,11.01,11.3,1

14/11/2009,05:07:38,21.84,8.16,8.2,1

14/11/2009,05:07:50,22.16,7.84,7.8,1

14/11/2009,05:07:51,22.27,7.73,7.7,1

14/11/2009,05:07:57,23.55,6.45,6.5,1

14/11/2009,05:08:01,15.39,14.61,16.9,1

14/11/2009,05:08:01,15.53,14.47,14.5,1

14/11/2009,05:08:00,13.15,16.85,14.6,2

14/11/2009,05:08:01,12.61,17.39,17.4,1

14/11/2009,05:09:23,18.88,11.12,11.1,1

14/11/2009,05:09:25,18.88,11.12,11.1,1

14/11/2009,05:10:28,21.7,8.3,8.3,2

14/11/2009,05:10:29,21.52,8.48,8.5,1

14/11/2009,05:10:42,20.41,9.59,9.6,1

14/11/2009,05:13:38,19.95,10.05,10.1,1

14/11/2009,05:13:43,16.07,13.93,13.9,1

14/11/2009,05:14:28,16.39,13.61,13.6,1

14/11/2009,05:18:36,24.51,5.49,5.5,1

14/11/2009,05:19:19,24.3,5.7,5.7,1

14/11/2009,05:19:24,24.16,5.84,5.8,2

14/11/2009,05:19:30,24.3,5.7,5.7,1

14/11/2009,05:19:33,24.41,5.59,5.6,1

14/11/2009,05:26:30,21.73,8.27,8.3,1

14/11/2009,05:26:32,23.62,6.38,6.4,1

14/11/2009,05:28:27,21.48,8.52,8.5,1

14/11/2009,05:28:38,22.2,7.8,7.8,1

14/11/2009,05:28:55,19.6,10.4,10.4,1

14/11/2009,05:30:50,21.45,8.55,8.6,1

14/11/2009,05:31:03,21.2,8.8,8.8,1

14/11/2009,05:35:28,21.95,8.05,7.8,1

14/11/2009,05:35:28,22.2,7.8,8.1,1

14/11/2009,05:35:55,21.16,8.84,8.8,1

14/11/2009,05:35:58,21.23,8.77,8.8,2

14/11/2009,05:36:01,21.23,8.77,8.8,1

14/11/2009,05:37:54,23.02,6.98,7.0,1

14/11/2009,05:38:47,22.48,7.52,7.5,1

14/11/2009,05:38:50,22.37,7.63,7.6,1

14/11/2009,05:39:28,21.31,8.69,8.6,1

14/11/2009,05:39:28,21.41,8.59,8.7,1

14/11/2009,05:39:36,21.41,8.59,8.6,1

14/11/2009,05:39:39,21.73,8.27,8.3,1

14/11/2009,05:39:47,21.73,8.27,8.3,1

14/11/2009,05:40:00,22.41,7.59,7.1,1

14/11/2009,05:39:59,22.87,7.13,7.6,1

14/11/2009,05:40:15,21.8,8.2,8.2,1

14/11/2009,05:40:50,23.66,6.34,6.3,1

14/11/2009,05:40:53,23.55,6.45,6.5,1

14/11/2009,05:41:20,23.55,6.45,6.5,1

14/11/2009,05:44:07,23.41,6.59,6.6,1

14/11/2009,05:44:12,23.48,6.52,6.5,1

14/11/2009,05:44:38,23.73,6.27,6.3,2

14/11/2009,05:44:45,23.87,6.13,7.1,1

14/11/2009,05:44:43,22.87,7.13,6.1,1

14/11/2009,05:44:45,22.87,7.13,7.1,1

14/11/2009,05:44:59,25.44,4.56,4.6,1

14/11/2009,05:44:57,25.37,4.63,4.7,1

14/11/2009,05:44:53,25.3,4.7,4.6,1

14/11/2009,05:44:52,25.44,4.56,4.6,1

14/11/2009,05:44:59,21.23,8.77,8.8,1

14/11/2009,05:45:06,21.84,8.16,8.2,1

14/11/2009,05:45:13,24.66,5.34,5.3,1

14/11/2009,05:46:36,25.72,4.28,4.3,1

14/11/2009,05:48:19,19.6,10.4,10.4,1

14/11/2009,05:48:21,18.17,11.83,11.8,2

14/11/2009,05:48:24,18.17,11.83,11.8,1

14/11/2009,05:51:43,19.42,10.58,10.6,1

14/11/2009,05:51:45,19.03,10.97,11.0,1

14/11/2009,05:51:45,18.7,11.3,11.3,1

14/11/2009,05:52:42,15.57,14.43,14.4,1

14/11/2009,05:53:19,19.7,10.3,10.3,1

14/11/2009,05:53:20,20.59,9.41,9.4,1

14/11/2009,05:54:32,18.6,11.4,11.4,1

14/11/2009,05:54:33,18.46,11.54,11.5,1

14/11/2009,05:54:34,18.46,11.54,11.5,1

14/11/2009,05:54:46,18.6,11.4,11.4,1

14/11/2009,05:54:47,18.67,11.33,11.3,1

14/11/2009,05:54:49,18.6,11.4,11.4,1

14/11/2009,05:56:17,18.63,11.37,11.4,1

14/11/2009,05:56:22,17.35,12.65,12.7,1

14/11/2009,05:57:10,24.9,5.1,5.2,1

14/11/2009,05:57:10,24.66,5.34,5.1,1

14/11/2009,05:57:12,24.37,5.63,5.3,1

14/11/2009,05:57:05,24.8,5.2,5.6,1

14/11/2009,05:57:51,19.13,10.87,10.9,1

14/11/2009,05:57:48,19.06,10.94,10.9,1

14/11/2009,06:00:27,19.7,10.3,5.3,1

14/11/2009,06:00:25,19.63,10.37,6.6,1

14/11/2009,06:00:23,19.67,10.33,6.5,1

14/11/2009,06:00:18,23.51,6.49,10.3,1

14/11/2009,06:00:14,23.44,6.56,10.4,1

14/11/2009,06:00:03,24.66,5.34,10.3,1

14/11/2009,06:00:44,21.56,8.44,8.4,1

14/11/2009,06:00:48,21.7,8.3,8.3,1

14/11/2009,06:00:50,21.8,8.2,8.2,1

14/11/2009,06:00:51,21.88,8.12,8.1,1

14/11/2009,06:00:59,24.16,5.84,5.8,1

14/11/2009,06:01:46,24.37,5.63,15.1,1

14/11/2009,06:01:14,14.93,15.07,5.6,1

14/11/2009,06:02:05,22.62,7.38,7.4,1

14/11/2009,06:02:08,22.55,7.45,7.4,1

14/11/2009,06:02:08,22.62,7.38,7.5,1

14/11/2009,06:02:11,22.59,7.41,7.4,1

14/11/2009,06:02:12,21.41,8.59,8.6,1

14/11/2009,06:02:47,23.05,6.95,7.0,1

14/11/2009,06:03:14,22.59,7.41,7.4,1

14/11/2009,06:03:36,22.45,7.55,7.6,1

14/11/2009,06:04:20,23.02,6.98,6.8,1

14/11/2009,06:04:19,23.16,6.84,7.0,1

14/11/2009,06:04:21,22.59,7.41,7.4,1

14/11/2009,06:04:23,22.55,7.45,7.5,1

14/11/2009,06:04:24,22.52,7.48,7.5,2

14/11/2009,06:04:35,22.41,7.59,7.6,1

14/11/2009,06:06:52,19.24,10.76,10.8,1

14/11/2009,06:06:53,17.67,12.33,12.1,3

14/11/2009,06:06:53,17.88,12.12,12.3,1

14/11/2009,06:07:13,22.16,7.84,7.9,2

14/11/2009,06:07:11,22.13,7.87,7.8,1

14/11/2009,06:07:26,20.06,9.94,9.9,1

14/11/2009,06:08:03,21.88,8.12,8.1,1

14/11/2009,06:08:44,19.63,10.37,10.4,1

14/11/2009,06:08:45,19.77,10.23,10.2,2

14/11/2009,06:09:24,24.26,5.74,5.7,2

14/11/2009,06:10:48,24.01,5.99,6.0,1

14/11/2009,06:12:01,21.73,8.27,8.3,1

14/11/2009,06:12:09,24.05,5.95,6.0,1

14/11/2009,06:13:27,24.58,5.42,5.4,1

14/11/2009,06:14:25,19.27,10.73,10.7,1

14/11/2009,06:14:46,21.48,8.52,8.5,2

14/11/2009,06:15:51,22.84,7.16,7.2,1

14/11/2009,06:16:04,21.27,8.73,8.7,1

14/11/2009,06:16:05,20.95,9.05,9.1,1

14/11/2009,06:16:17,24.26,5.74,5.7,1

14/11/2009,06:17:53,24.26,5.74,5.6,1

14/11/2009,06:17:52,24.44,5.56,5.7,1

14/11/2009,06:18:00,24.55,5.45,5.5,1

14/11/2009,06:18:13,24.58,5.42,5.4,2

14/11/2009,06:19:05,23.8,6.2,6.2,1

14/11/2009,06:20:59,22.16,7.84,7.8,1

14/11/2009,06:21:05,22.98,7.02,7.0,2

14/11/2009,06:22:45,19.84,10.16,9.9,1

14/11/2009,06:22:44,20.13,9.87,10.2,1

14/11/2009,06:23:40,18.46,11.54,11.5,1

14/11/2009,06:23:52,24.19,5.81,5.8,1

14/11/2009,06:24:17,24.55,5.45,15.6,2

14/11/2009,06:24:20,24.48,5.52,5.5,1

14/11/2009,06:24:28,25.15,4.85,5.5,2

14/11/2009,06:24:00,14.39,15.61,9.6,1

14/11/2009,06:24:21,20.45,9.55,4.9,1

14/11/2009,06:25:00,25.15,4.85,7.6,1

14/11/2009,06:24:58,22.45,7.55,7.5,2

14/11/2009,06:24:59,22.55,7.45,4.9,1

14/11/2009,06:25:03,22.73,7.27,7.3,2

14/11/2009,06:25:04,22.55,7.45,7.5,1

14/11/2009,06:25:41,24.76,5.24,13.5,1

14/11/2009,06:25:43,24.44,5.56,13.7,1

14/11/2009,06:25:27,16.35,13.65,5.2,1

14/11/2009,06:25:26,16.5,13.5,5.6,1

14/11/2009,06:25:56,23.3,6.7,6.7,1

14/11/2009,06:26:00,23.3,6.7,6.7,1

14/11/2009,06:26:11,24.8,5.2,5.2,1

14/11/2009,06:26:16,24.73,5.27,5.3,2

14/11/2009,06:26:19,24.9,5.1,5.1,1

14/11/2009,06:26:20,25.05,4.95,5.4,1

14/11/2009,06:26:21,24.83,5.17,5.1,1

14/11/2009,06:26:22,24.69,5.31,5.0,1

14/11/2009,06:26:25,24.83,5.17,5.2,1

14/11/2009,06:26:18,24.62,5.38,5.2,1

14/11/2009,06:26:18,24.87,5.13,5.5,1

14/11/2009,06:26:21,24.83,5.17,5.3,1

14/11/2009,06:26:21,24.55,5.45,5.2,1

14/11/2009,06:26:25,24.83,5.17,5.2,1

14/11/2009,06:27:18,24.05,5.95,6.0,1

14/11/2009,06:28:07,23.59,6.41,8.8,2

14/11/2009,06:28:05,21.38,8.62,8.6,1

14/11/2009,06:28:03,21.2,8.8,6.4,1

14/11/2009,06:28:13,23.76,6.24,5.8,1

14/11/2009,06:28:13,24.23,5.77,6.2,1

14/11/2009,06:28:36,22.62,7.38,7.4,1

14/11/2009,06:28:36,22.52,7.48,7.5,1

14/11/2009,06:28:40,22.73,7.27,7.3,2

14/11/2009,06:28:50,24.87,5.13,5.1,1

14/11/2009,06:28:55,25.69,4.31,4.3,1

14/11/2009,06:28:56,25.47,4.53,4.5,1

14/11/2009,06:28:59,25.33,4.67,4.7,1

14/11/2009,06:29:07,25.23,4.77,4.8,1

14/11/2009,06:29:07,25.12,4.88,4.9,1

14/11/2009,06:31:19,18.17,11.83,11.8,1

14/11/2009,06:31:23,20.06,9.94,9.9,1

14/11/2009,06:33:44,19.84,10.16,10.2,1

14/11/2009,06:34:11,21.06,8.94,8.9,1

14/11/2009,06:34:19,22.7,7.3,7.3,1

14/11/2009,06:34:22,22.48,7.52,7.5,1

14/11/2009,06:35:43,17.67,12.33,12.3,1

14/11/2009,06:39:49,20.63,9.37,8.9,1

14/11/2009,06:39:49,21.06,8.94,9.4,1

14/11/2009,06:40:26,22.84,7.16,7.2,1

14/11/2009,06:54:18,18.53,11.47,11.5,1

14/11/2009,07:01:27,22.59,7.41,6.7,2

14/11/2009,07:01:25,22.98,7.02,7.0,1

14/11/2009,07:01:25,23.3,6.7,7.4,1

14/11/2009,07:08:02,20.66,9.34,9.3,1

14/11/2009,07:08:05,21.77,8.23,8.2,1

14/11/2009,08:01:17,22.05,7.95,8.0,2

14/11/2009,08:01:18,21.7,8.3,8.3,3

14/11/2009,08:01:18,21.38,8.62,8.6,1

14/11/2009,08:01:26,20.56,9.44,9.4,1

14/11/2009,08:07:54,25.33,4.67,4.9,1

14/11/2009,08:07:53,25.12,4.88,5.1,2

14/11/2009,08:07:53,24.87,5.13,4.7,1

14/11/2009,08:07:56,24.55,5.45,5.5,1

14/11/2009,08:07:59,24.01,5.99,5.7,1

14/11/2009,08:07:59,24.16,5.84,5.8,1

14/11/2009,08:07:58,24.33,5.67,6.0,1

14/11/2009,09:25:04,19.6,10.4,10.4,1

14/11/2009,09:25:18,19.45,10.55,10.6,1

14/11/2009,09:25:18,19.31,10.69,10.7,1

14/11/2009,09:25:24,18.03,11.97,12.0,1

14/11/2009,09:25:27,18.17,11.83,11.8,1

14/11/2009,09:25:32,18.49,11.51,11.5,1

14/11/2009,09:25:33,18.38,11.62,11.6,1

14/11/2009,09:25:35,18.24,11.76,11.8,1

14/11/2009,09:25:37,18.63,11.37,11.4,1

14/11/2009,09:25:40,18.88,11.12,11.1,1

14/11/2009,09:25:43,19.17,10.83,10.8,1

14/11/2009,09:25:44,18.53,11.47,11.5,1

14/11/2009,09:25:44,18.1,11.9,11.9,1

14/11/2009,09:25:47,17.85,12.15,12.2,1

14/11/2009,09:25:49,17.24,12.76,12.8,1

14/11/2009,09:32:17,17.64,12.36,12.0,1

14/11/2009,09:32:15,18.03,11.97,12.0,1

14/11/2009,09:32:14,18.03,11.97,12.4,1

14/11/2009,10:00:39,23.41,6.59,6.5,1

14/11/2009,10:00:37,23.55,6.45,6.6,2

14/11/2009,10:23:30,18.81,11.19,11.2,1

14/11/2009,10:23:47,19.42,10.58,10.6,1

14/11/2009,10:23:48,19.38,10.62,10.6,1

14/11/2009,10:23:51,18.92,11.08,11.1,1

14/11/2009,10:23:52,18.88,11.12,11.1,1

14/11/2009,10:23:52,18.67,11.33,11.3,1

14/11/2009,10:25:35,19.74,10.26,10.3,1

14/11/2009,10:26:29,19.31,10.69,10.7,1

14/11/2009,10:26:32,19.24,10.76,10.8,1

14/11/2009,10:26:34,19.17,10.83,10.8,1

14/11/2009,10:26:35,19.17,10.83,10.8,1

14/11/2009,10:26:40,18.56,11.44,11.4,1

14/11/2009,10:26:41,18.31,11.69,11.7,1

14/11/2009,10:26:41,18.24,11.76,11.8,1

14/11/2009,10:26:42,18.1,11.9,11.9,1

14/11/2009,10:26:45,18.35,11.65,11.7,1

14/11/2009,10:26:55,18.06,11.94,11.9,1

14/11/2009,10:26:56,17.96,12.04,12.0,1

14/11/2009,10:59:34,22.34,7.66,7.7,1

14/11/2009,10:59:40,21.84,8.16,8.2,1

14/11/2009,10:59:49,21.98,8.02,8.0,1

14/11/2009,10:59:50,21.91,8.09,8.1,1

14/11/2009,11:00:12,23.34,6.66,6.7,1

14/11/2009,11:00:13,23.05,6.95,7.0,1

14/11/2009,11:00:34,22.66,7.34,7.3,1

14/11/2009,11:01:26,22.66,7.34,7.3,1

14/11/2009,11:01:29,22.84,7.16,7.2,1

14/11/2009,11:01:49,23.34,6.66,6.7,1

14/11/2009,11:01:52,23.37,6.63,6.6,1

14/11/2009,12:20:17,23.87,6.13,6.0,1

14/11/2009,12:20:16,23.98,6.02,6.1,1

14/11/2009,12:34:50,23.48,6.52,3.7,1

14/11/2009,12:34:48,23.69,6.31,3.9,1

14/11/2009,12:34:47,23.94,6.06,4.1,1

14/11/2009,12:34:38,23.87,6.13,3.9,1

14/11/2009,12:34:38,23.98,6.02,4.2,1

14/11/2009,12:34:37,24.26,5.74,4.3,1

14/11/2009,12:34:39,24.3,5.7,4.7,1

14/11/2009,12:34:36,24.48,5.52,4.8,1

14/11/2009,12:34:34,24.51,5.49,5.1,1

14/11/2009,12:34:33,24.62,5.38,5.3,1

14/11/2009,12:34:33,24.66,5.34,5.4,1

14/11/2009,12:34:33,24.9,5.1,5.5,1

14/11/2009,12:34:30,25.23,4.77,5.5,1

14/11/2009,12:34:30,25.3,4.7,5.7,1

14/11/2009,12:34:29,25.69,4.31,6.0,1

14/11/2009,12:34:28,25.83,4.17,6.1,1

14/11/2009,12:34:25,26.15,3.85,5.7,1

14/11/2009,12:34:13,26.15,3.85,6.1,1

14/11/2009,12:34:13,26.26,3.74,6.3,1

14/11/2009,12:34:16,25.94,4.06,6.5,1

14/11/2009,12:46:45,25.83,4.17,4.0,1

14/11/2009,12:46:43,25.97,4.03,4.2,1

14/11/2009,12:47:18,26.19,3.81,3.8,1

14/11/2009,12:47:23,26.19,3.81,3.8,2

14/11/2009,12:47:32,26.15,3.85,3.9,1

14/11/2009,13:34:00,21.98,8.02,8.0,1

14/11/2009,13:34:08,21.91,8.09,8.1,2

14/11/2009,13:34:20,21.66,8.34,8.3,1

14/11/2009,13:38:01,22.41,7.59,7.6,1

14/11/2009,13:38:04,22.37,7.63,7.6,1

14/11/2009,13:49:50,22.8,7.2,7.2,1

14/11/2009,14:15:56,25.58,4.42,4.4,1

14/11/2009,14:15:58,25.65,4.35,4.4,1

14/11/2009,14:16:05,26.04,3.96,4.0,1

14/11/2009,14:16:07,26.08,3.92,3.9,1

14/11/2009,14:16:10,26.04,3.96,4.0,1

14/11/2009,14:16:25,26.61,3.39,3.4,1

14/11/2009,14:16:26,26.44,3.56,3.6,1

14/11/2009,14:16:27,26.22,3.78,3.8,1

14/11/2009,14:16:28,25.9,4.1,4.1,1

14/11/2009,14:16:29,25.69,4.31,4.3,1

14/11/2009,14:16:29,25.47,4.53,4.5,1

14/11/2009,14:16:33,24.9,5.1,5.1,1

14/11/2009,14:17:19,23.37,6.63,6.6,1

14/11/2009,14:17:47,23.44,6.56,6.6,1

14/11/2009,14:18:34,24.48,5.52,5.5,1

14/11/2009,14:18:35,24.51,5.49,5.5,1

14/11/2009,14:18:37,24.8,5.2,5.2,1

14/11/2009,14:18:38,24.8,5.2,5.2,1

14/11/2009,14:18:40,24.83,5.17,5.2,1

14/11/2009,14:18:43,24.83,5.17,5.2,1

14/11/2009,14:18:45,24.87,5.13,5.1,3

14/11/2009,14:18:48,24.9,5.1,5.1,1

14/11/2009,14:18:51,24.87,5.13,5.1,1

14/11/2009,14:18:52,24.87,5.13,5.1,2

14/11/2009,14:18:53,24.94,5.06,5.1,1

14/11/2009,14:18:54,24.87,5.13,5.1,1

14/11/2009,14:18:58,24.48,5.52,5.5,1

14/11/2009,14:18:59,24.09,5.91,5.9,1

14/11/2009,14:19:02,24.19,5.81,5.8,1

14/11/2009,14:20:22,23.8,6.2,6.1,2

14/11/2009,14:20:21,23.94,6.06,6.2,1

14/11/2009,14:20:34,24.69,5.31,5.3,1

14/11/2009,14:20:35,24.62,5.38,5.4,1

14/11/2009,14:20:36,24.69,5.31,5.3,1

14/11/2009,14:20:38,24.73,5.27,5.3,1

14/11/2009,14:20:41,24.8,5.2,5.2,1

14/11/2009,14:20:42,24.8,5.2,5.2,1

14/11/2009,14:20:43,24.73,5.27,5.3,1

14/11/2009,14:20:45,24.8,5.2,5.2,1

14/11/2009,14:20:47,24.76,5.24,5.2,1

14/11/2009,14:20:50,24.66,5.34,5.3,1

14/11/2009,14:20:52,24.94,5.06,5.1,1

14/11/2009,14:20:53,25.05,4.95,5.0,1

14/11/2009,14:21:03,24.98,5.02,5.0,1

14/11/2009,14:21:06,25.05,4.95,5.0,1

14/11/2009,14:21:09,25.08,4.92,4.9,1

14/11/2009,14:21:10,25.12,4.88,4.9,1

14/11/2009,14:21:12,25.19,4.81,4.8,1

14/11/2009,14:21:18,25.37,4.63,4.6,1

14/11/2009,14:21:20,25.37,4.63,4.6,2

14/11/2009,14:21:22,25.3,4.7,4.7,1

14/11/2009,14:21:23,25.33,4.67,4.7,1

14/11/2009,14:21:28,25.55,4.45,4.5,1

14/11/2009,14:21:30,25.51,4.49,4.5,2

14/11/2009,14:21:32,25.76,4.24,4.2,1

14/11/2009,14:21:34,25.62,4.38,4.4,1

14/11/2009,14:21:35,25.4,4.6,4.6,1

14/11/2009,14:21:36,24.98,5.02,5.0,1

14/11/2009,15:47:28,26.86,3.14,3.1,1

14/11/2009,15:47:29,26.76,3.24,3.2,1

14/11/2009,15:55:32,23.55,6.45,6.5,1

14/11/2009,15:55:34,22.98,7.02,7.0,1

14/11/2009,15:56:10,24.01,5.99,6.0,1

14/11/2009,15:56:13,24.01,5.99,6.0,1

14/11/2009,16:30:49,24.94,5.06,5.1,1

14/11/2009,16:42:55,19.2,10.8,10.7,1

14/11/2009,16:42:55,19.31,10.69,10.8,1

14/11/2009,16:58:00,25.19,4.81,4.8,1

14/11/2009,17:54:54,23.91,6.09,6.1,1

14/11/2009,17:54:58,23.76,6.24,6.2,1

14/11/2009,17:54:58,23.55,6.45,6.5,1

14/11/2009,18:07:28,26.15,3.85,3.9,1

14/11/2009,18:07:30,26.08,3.92,3.9,1

14/11/2009,22:25:21,23.48,6.52,6.7,1

14/11/2009,22:25:19,23.27,6.73,6.5,1

14/11/2009,22:31:29,23.41,6.59,6.2,1

14/11/2009,22:31:25,23.8,6.2,6.6,1

15/11/2009,00:26:35,25.72,4.28,4.3,1

15/11/2009,00:26:36,25.55,4.45,4.5,1

15/11/2009,00:26:41,25.97,4.03,4.0,1

15/11/2009,00:33:26,26.08,3.92,3.9,1

15/11/2009,00:33:29,26.08,3.92,3.9,1

15/11/2009,01:25:36,18.63,11.37,11.4,1

15/11/2009,01:27:50,18.03,11.97,12.0,1

15/11/2009,01:27:49,17.96,12.04,12.0,1

15/11/2009,01:56:09,24.8,5.2,5.2,1

15/11/2009,01:56:11,24.62,5.38,5.4,1

15/11/2009,01:56:12,24.94,5.06,5.1,1

15/11/2009,01:56:13,24.9,5.1,5.1,1

15/11/2009,01:56:15,25.05,4.95,5.0,1

15/11/2009,01:57:23,25.55,4.45,4.5,1

15/11/2009,02:00:00,16.92,13.08,13.1,1

15/11/2009,02:00:02,17.07,12.93,12.9,1

15/11/2009,02:13:29,24.05,5.95,6.0,1

15/11/2009,02:13:37,25.12,4.88,4.9,1

15/11/2009,02:13:42,25.01,4.99,5.0,1

15/11/2009,02:13:42,24.94,5.06,5.1,1

15/11/2009,02:13:48,24.73,5.27,5.4,1

15/11/2009,02:13:50,24.98,5.02,5.3,2

15/11/2009,02:13:42,24.58,5.42,5.0,1

15/11/2009,02:22:45,16.42,13.58,13.6,1

15/11/2009,02:22:45,16.03,13.97,14.0,1

15/11/2009,02:29:59,24.12,5.88,6.2,1

15/11/2009,02:29:52,23.8,6.2,5.9,1

15/11/2009,02:37:58,25.23,4.77,4.8,1

15/11/2009,02:38:00,25.23,4.77,4.8,1

15/11/2009,02:38:01,25.3,4.7,4.7,1

15/11/2009,02:38:03,25.12,4.88,4.9,1

15/11/2009,02:38:03,20.34,9.66,9.7,1

15/11/2009,02:43:47,23.8,6.2,6.2,1

15/11/2009,02:43:51,23.76,6.24,6.2,1

15/11/2009,02:43:52,23.91,6.09,6.1,1

15/11/2009,02:43:54,23.87,6.13,6.1,1

15/11/2009,02:51:24,19.6,10.4,10.4,1

15/11/2009,03:16:26,20.13,9.87,9.9,1

15/11/2009,03:16:29,20.17,9.83,9.8,1

15/11/2009,03:16:32,20.24,9.76,9.8,1

15/11/2009,03:16:35,20.34,9.66,9.7,1

15/11/2009,03:19:05,24.9,5.1,5.1,1

15/11/2009,03:19:07,24.9,5.1,5.1,1

15/11/2009,03:28:23,23.16,6.84,6.8,1

15/11/2009,03:28:25,23.27,6.73,6.7,1

15/11/2009,03:28:35,23.34,6.66,6.7,1

15/11/2009,03:28:50,24.01,5.99,6.0,1

15/11/2009,03:30:23,21.59,8.41,8.4,1

15/11/2009,03:30:24,21.27,8.73,8.7,1

15/11/2009,03:30:30,21.31,8.69,8.7,1

15/11/2009,03:30:35,21.8,8.2,8.2,1

15/11/2009,03:30:36,22.16,7.84,7.8,1

15/11/2009,03:30:36,21.88,8.12,8.1,1

15/11/2009,03:30:37,21.73,8.27,8.3,1

15/11/2009,03:33:38,21.77,8.23,8.2,1

15/11/2009,03:33:42,22.52,7.48,7.5,1

15/11/2009,03:37:39,26.01,3.99,3.9,1

15/11/2009,03:37:39,26.15,3.85,4.0,1

15/11/2009,03:49:19,18.42,11.58,11.6,1

15/11/2009,03:49:21,18.46,11.54,11.5,1

15/11/2009,03:53:37,17.74,12.26,12.3,2

15/11/2009,03:53:42,18.38,11.62,11.6,1

15/11/2009,03:59:19,5.59,24.41,24.4,1

15/11/2009,04:02:54,21.06,8.94,8.9,1

15/11/2009,04:11:45,13.61,16.39,16.4,1

15/11/2009,04:11:46,13.5,16.5,16.5,1

15/11/2009,04:11:47,13.54,16.46,16.5,1

15/11/2009,04:13:28,22.94,7.06,7.1,1

15/11/2009,04:13:42,25.08,4.92,4.9,1

15/11/2009,04:16:07,19.84,10.16,10.2,1

15/11/2009,04:16:10,19.95,10.05,10.1,1

15/11/2009,04:17:01,21.45,8.55,8.6,1

15/11/2009,04:17:05,21.45,8.55,8.6,1

15/11/2009,04:28:49,19.13,10.87,10.9,1

15/11/2009,04:28:54,19.27,10.73,10.7,1

15/11/2009,04:28:57,19.38,10.62,10.6,1

15/11/2009,04:33:12,19.03,10.97,11.0,1

15/11/2009,04:33:13,18.63,11.37,11.4,1

15/11/2009,04:33:40,16.67,13.33,13.3,1

15/11/2009,04:33:41,16.64,13.36,13.4,1

15/11/2009,04:33:44,16.64,13.36,13.4,1

15/11/2009,04:36:23,23.69,6.31,6.3,1

15/11/2009,04:39:38,17.71,12.29,12.3,1

15/11/2009,04:39:39,17.49,12.51,12.5,1

15/11/2009,04:48:01,18.06,11.94,11.9,1

15/11/2009,04:49:08,24.76,5.24,5.2,1

15/11/2009,04:49:09,24.76,5.24,5.2,1

15/11/2009,04:50:32,7.77,22.23,22.2,1

15/11/2009,04:50:38,8.01,21.99,22.0,1

15/11/2009,04:55:22,21.27,8.73,8.7,1

15/11/2009,04:56:40,22.66,7.34,7.3,1

15/11/2009,04:56:47,22.8,7.2,7.2,2

15/11/2009,04:57:07,23.19,6.81,6.8,1

15/11/2009,04:58:16,17.56,12.44,12.4,1

15/11/2009,05:03:05,25.4,4.6,4.6,1

15/11/2009,05:03:07,25.19,4.81,4.8,1

15/11/2009,05:10:11,23.69,6.31,6.3,1

15/11/2009,05:10:12,23.05,6.95,7.0,1

15/11/2009,05:11:39,22.41,7.59,7.6,1

15/11/2009,05:11:40,22.37,7.63,7.6,1

15/11/2009,05:12:47,25.62,4.38,4.4,1

15/11/2009,05:12:49,25.26,4.74,4.7,1

15/11/2009,05:12:53,19.84,10.16,10.2,1

15/11/2009,05:12:53,18.56,11.44,11.4,2

15/11/2009,05:12:55,18.67,11.33,11.3,1

15/11/2009,05:13:32,25.72,4.28,4.3,1

15/11/2009,05:13:33,25.62,4.38,4.4,1

15/11/2009,05:20:20,16.28,13.72,13.7,1

15/11/2009,05:20:21,16.57,13.43,13.4,1

15/11/2009,05:21:03,21.63,8.37,8.4,1

15/11/2009,05:21:08,21.8,8.2,8.2,1

15/11/2009,05:21:11,21.77,8.23,8.2,1

15/11/2009,05:21:13,21.88,8.12,8.1,1

15/11/2009,05:22:12,26.29,3.71,3.7,1

15/11/2009,05:22:22,24.83,5.17,5.2,1

15/11/2009,05:23:13,24.37,5.63,5.6,1

15/11/2009,05:24:10,19.67,10.33,10.3,1

15/11/2009,05:24:19,19.49,10.51,10.5,1

15/11/2009,05:24:21,19.84,10.16,10.2,1

15/11/2009,05:25:18,24.01,5.99,6.0,1

15/11/2009,05:25:36,24.05,5.95,5.7,1

15/11/2009,05:25:34,24.3,5.7,6.0,1

15/11/2009,05:26:02,24.66,5.34,5.3,1

15/11/2009,05:26:14,24.44,5.56,5.6,1

15/11/2009,05:27:37,24.58,5.42,5.4,1

15/11/2009,05:27:51,25.01,4.99,5.0,1

15/11/2009,05:28:05,25.05,4.95,5.0,1

15/11/2009,05:29:05,23.23,6.77,6.8,1

15/11/2009,05:29:08,22.94,7.06,7.1,1

15/11/2009,05:29:21,20.56,9.44,9.4,1

15/11/2009,05:31:07,24.26,5.74,5.7,1

15/11/2009,05:31:35,21.7,8.3,8.3,1

15/11/2009,05:32:05,25.05,4.95,5.0,1

15/11/2009,05:34:48,24.16,5.84,5.8,1

15/11/2009,05:34:51,24.16,5.84,5.8,1

15/11/2009,05:37:21,25.44,4.56,4.6,1

15/11/2009,05:40:37,15.07,14.93,14.9,1

15/11/2009,05:40:39,15.32,14.68,14.7,1

15/11/2009,05:40:40,15.03,14.97,15.0,1

15/11/2009,05:44:17,19.95,10.05,10.1,1

15/11/2009,05:44:18,19.7,10.3,10.3,1

15/11/2009,05:44:19,19.63,10.37,10.4,1

15/11/2009,05:44:20,19.6,10.4,10.4,1

15/11/2009,05:44:23,19.84,10.16,11.7,1

15/11/2009,05:44:25,19.99,10.01,10.2,1

15/11/2009,05:44:27,20.09,9.91,10.0,1

15/11/2009,05:44:20,18.31,11.69,9.9,2

15/11/2009,05:44:43,21.59,8.41,8.4,2

15/11/2009,05:44:44,21.66,8.34,8.3,1

15/11/2009,05:44:46,21.73,8.27,8.3,2

15/11/2009,05:45:01,23.23,6.77,6.8,2

15/11/2009,05:45:04,23.19,6.81,6.8,1

15/11/2009,05:45:08,23.51,6.49,6.5,1

15/11/2009,05:45:17,23.91,6.09,6.1,1

15/11/2009,05:45:21,23.94,6.06,6.1,1

15/11/2009,05:45:27,20.66,9.34,9.3,1

15/11/2009,05:45:37,25.58,4.42,4.4,1

15/11/2009,05:45:43,24.01,5.99,6.0,2

15/11/2009,05:45:44,23.73,6.27,6.3,1

15/11/2009,05:45:47,21.8,8.2,8.2,1

15/11/2009,05:45:56,23.27,6.73,6.7,1

15/11/2009,05:45:57,23.23,6.77,6.8,1

15/11/2009,05:46:12,23.69,6.31,6.3,1

15/11/2009,05:46:18,24.94,5.06,5.1,1

15/11/2009,05:46:29,25.58,4.42,4.4,1

15/11/2009,05:46:30,25.33,4.67,4.7,1

15/11/2009,05:49:06,12.54,17.46,17.5,1

15/11/2009,05:49:08,12.9,17.1,17.1,2

15/11/2009,05:49:10,12.93,17.07,17.1,1

15/11/2009,05:49:53,22.48,7.52,7.5,1

15/11/2009,05:49:55,22.84,7.16,7.2,1

15/11/2009,05:50:46,23.62,6.38,6.4,1

15/11/2009,05:50:49,23.66,6.34,6.3,1

15/11/2009,05:52:45,23.59,6.41,6.4,1

15/11/2009,05:52:47,23.44,6.56,7.4,1

15/11/2009,05:52:46,22.41,7.59,7.6,1

15/11/2009,05:52:46,22.59,7.41,6.6,1

15/11/2009,05:52:48,21.59,8.41,8.4,1

15/11/2009,05:52:50,20.38,9.62,9.6,1

15/11/2009,05:52:51,20.13,9.87,9.9,1

15/11/2009,05:53:16,24.98,5.02,5.0,1

15/11/2009,05:53:58,18.78,11.22,10.9,1

15/11/2009,05:53:57,19.06,10.94,11.2,1

15/11/2009,05:54:43,19.77,10.23,10.2,1

15/11/2009,05:54:45,19.63,10.37,10.4,1

15/11/2009,05:54:47,19.77,10.23,10.2,1

15/11/2009,05:56:47,24.05,5.95,6.0,1

15/11/2009,05:56:54,22.91,7.09,7.1,1

15/11/2009,05:56:54,22.77,7.23,7.2,1

15/11/2009,05:56:57,22.91,7.09,7.1,1

15/11/2009,05:57:42,21.23,8.77,8.8,1

15/11/2009,05:57:40,21.23,8.77,8.8,1

15/11/2009,05:57:49,20.84,9.16,9.2,1

15/11/2009,05:57:50,20.77,9.23,9.2,1

15/11/2009,05:58:00,24.76,5.24,5.2,1

15/11/2009,05:59:00,24.62,5.38,8.1,1

15/11/2009,05:59:02,24.37,5.63,5.4,1

15/11/2009,05:58:51,21.88,8.12,5.6,1

15/11/2009,05:59:23,23.84,6.16,6.2,1

15/11/2009,05:59:26,24.05,5.95,6.0,1

15/11/2009,05:59:29,23.69,6.31,6.3,1

15/11/2009,05:59:30,23.69,6.31,6.3,1

15/11/2009,05:59:31,23.73,6.27,6.3,1

15/11/2009,05:59:38,25.01,4.99,5.0,1

15/11/2009,05:59:40,19.63,10.37,14.3,1

15/11/2009,05:59:44,19.24,10.76,10.4,1

15/11/2009,05:59:42,15.75,14.25,14.3,1

15/11/2009,05:59:39,15.68,14.32,10.8,1

15/11/2009,06:01:45,24.01,5.99,6.0,1

15/11/2009,06:02:40,22.98,7.02,7.0,2

15/11/2009,06:02:43,23.05,6.95,7.0,1

15/11/2009,06:02:44,23.05,6.95,7.0,1

15/11/2009,06:03:51,21.88,8.12,8.1,1

15/11/2009,06:03:53,21.98,8.02,8.0,1

15/11/2009,06:03:57,22.13,7.87,7.9,1

15/11/2009,06:04:10,20.06,9.94,9.9,1

15/11/2009,06:04:17,24.48,5.52,5.5,1

15/11/2009,06:04:24,24.73,5.27,5.3,2

15/11/2009,06:04:41,23.37,6.63,6.6,1

15/11/2009,06:04:47,23.98,6.02,6.0,1

15/11/2009,06:04:51,22.16,7.84,8.5,1

15/11/2009,06:04:52,21.66,8.34,7.8,1

15/11/2009,06:04:50,21.48,8.52,8.3,1

15/11/2009,06:05:04,21.38,8.62,8.6,2

15/11/2009,06:06:03,20.98,9.02,9.0,1

15/11/2009,06:06:04,21.13,8.87,8.9,1

15/11/2009,06:06:11,21.56,8.44,8.3,1

15/11/2009,06:06:10,21.7,8.3,8.4,1

15/11/2009,06:06:12,21.16,8.84,8.8,1

15/11/2009,06:06:13,21.06,8.94,8.9,1

15/11/2009,06:06:15,20.7,9.3,9.3,1

15/11/2009,06:06:17,20.66,9.34,9.3,1

15/11/2009,06:06:17,20.24,9.76,9.8,1

15/11/2009,06:06:19,19.95,10.05,10.1,1

15/11/2009,06:06:19,19.7,10.3,10.3,1

15/11/2009,06:06:39,22.45,7.55,7.6,2

15/11/2009,06:06:59,21.27,8.73,8.7,1

15/11/2009,06:07:00,21.09,8.91,9.3,1

15/11/2009,06:07:00,21.02,8.98,8.9,1

15/11/2009,06:07:01,20.91,9.09,9.0,1

15/11/2009,06:06:59,20.66,9.34,9.1,1

15/11/2009,06:07:08,19.35,10.65,10.7,1

15/11/2009,06:07:10,19.2,10.8,10.8,1

15/11/2009,06:08:46,25.05,4.95,5.0,1

15/11/2009,06:08:49,25.12,4.88,4.9,1

15/11/2009,06:08:54,17.78,12.22,12.2,1

15/11/2009,06:08:58,10.62,19.38,19.4,1

15/11/2009,06:08:58,9.69,20.31,20.3,1

15/11/2009,06:09:27,22.16,7.84,7.8,1

15/11/2009,06:09:36,23.76,6.24,6.2,1

15/11/2009,06:10:12,25.33,4.67,4.7,1

15/11/2009,06:10:39,25.47,4.53,4.5,1

15/11/2009,06:10:44,23.12,6.88,6.9,1

15/11/2009,06:10:55,24.48,5.52,5.5,1

15/11/2009,06:10:56,22.91,7.09,7.1,1

15/11/2009,06:11:13,23.16,6.84,6.8,1

15/11/2009,06:11:16,22.62,7.38,7.4,1

15/11/2009,06:11:17,22.52,7.48,7.5,1

15/11/2009,06:11:17,22.34,7.66,7.7,2

15/11/2009,06:11:18,22.05,7.95,8.0,1

15/11/2009,06:11:29,22.77,7.23,7.2,1

15/11/2009,06:11:35,24.01,5.99,6.0,1

15/11/2009,06:11:38,23.37,6.63,6.6,1

15/11/2009,06:11:42,23.66,6.34,6.3,1

15/11/2009,06:11:43,23.48,6.52,6.5,1

15/11/2009,06:11:49,23.69,6.31,6.3,1

15/11/2009,06:11:50,23.76,6.24,6.2,1

15/11/2009,06:11:53,23.84,6.16,6.2,1

15/11/2009,06:12:00,21.52,8.48,8.5,1

15/11/2009,06:12:06,22.98,7.02,7.0,1

15/11/2009,06:12:08,22.98,7.02,7.0,1

15/11/2009,06:12:14,23.41,6.59,6.6,1

15/11/2009,06:12:20,22.59,7.41,7.4,1

15/11/2009,06:12:22,22.66,7.34,7.3,1

15/11/2009,06:12:23,21.59,8.41,8.4,1

15/11/2009,06:12:45,22.27,7.73,7.7,1

15/11/2009,06:12:48,21.23,8.77,8.8,1

15/11/2009,06:12:49,21.31,8.69,8.7,1

15/11/2009,06:12:51,20.31,9.69,9.7,1

15/11/2009,06:12:52,20.31,9.69,9.7,1

15/11/2009,06:13:22,25.33,4.67,4.7,2

15/11/2009,06:13:25,25.4,4.6,4.6,1

15/11/2009,06:13:31,25.37,4.63,7.5,1

15/11/2009,06:13:33,25.44,4.56,4.6,1

15/11/2009,06:13:35,25.37,4.63,4.6,1

15/11/2009,06:13:38,25.37,4.63,4.6,1

15/11/2009,06:13:40,25.4,4.6,4.6,1

15/11/2009,06:13:27,22.48,7.52,4.6,1

15/11/2009,06:13:43,21.16,8.84,8.8,3

15/11/2009,06:13:54,21.23,8.77,8.8,1

15/11/2009,06:14:08,19.03,10.97,11.0,1

15/11/2009,06:14:31,22.02,7.98,8.0,1

15/11/2009,06:14:46,24.76,5.24,5.2,1

15/11/2009,06:14:47,24.16,5.84,5.8,1

15/11/2009,06:14:52,22.91,7.09,7.9,1

15/11/2009,06:14:50,22.13,7.87,7.1,1

15/11/2009,06:15:10,22.77,7.23,7.2,1

15/11/2009,06:15:12,22.84,7.16,7.2,1

15/11/2009,06:15:13,22.87,7.13,7.1,1

15/11/2009,06:15:14,22.77,7.23,7.2,1

15/11/2009,06:15:15,22.59,7.41,7.4,1

15/11/2009,06:15:15,22.37,7.63,7.6,1

15/11/2009,06:15:16,22.09,7.91,7.9,2

15/11/2009,06:15:37,24.26,5.74,5.7,1

15/11/2009,06:15:42,23.91,6.09,6.1,1

15/11/2009,06:15:44,23.87,6.13,6.1,1

15/11/2009,06:15:55,24.19,5.81,5.8,1

15/11/2009,06:16:07,24.05,5.95,6.0,1

15/11/2009,06:16:10,24.23,5.77,5.8,1

15/11/2009,06:16:23,24.66,5.34,5.3,1

15/11/2009,06:16:23,24.51,5.49,5.5,1

15/11/2009,06:16:25,24.51,5.49,5.5,1

15/11/2009,06:16:27,24.58,5.42,5.4,1

15/11/2009,06:16:29,24.09,5.91,5.9,1

15/11/2009,06:16:31,24.23,5.77,5.8,2

15/11/2009,06:16:32,24.26,5.74,5.7,1

15/11/2009,06:16:46,16.6,13.4,13.4,1

15/11/2009,06:16:46,16.17,13.83,13.8,1

15/11/2009,06:17:22,24.69,5.31,16.8,2

15/11/2009,06:17:28,24.73,5.27,16.6,1

15/11/2009,06:17:11,13.36,16.64,5.3,1

15/11/2009,06:17:09,13.25,16.75,5.3,1

15/11/2009,06:17:38,20.59,9.41,9.4,1

15/11/2009,06:17:43,20.91,9.09,9.1,1

15/11/2009,06:17:45,20.91,9.09,9.1,1

15/11/2009,06:17:52,21.02,8.98,9.0,1

15/11/2009,06:17:55,21.16,8.84,8.8,1

15/11/2009,06:18:03,21.23,8.77,8.8,1

15/11/2009,06:18:04,21.38,8.62,8.6,1

15/11/2009,06:18:10,24.26,5.74,5.7,1

15/11/2009,06:18:10,24.26,5.74,5.7,1

15/11/2009,06:18:12,22.7,7.3,7.3,2

15/11/2009,06:18:18,22.05,7.95,8.0,1

15/11/2009,06:18:19,22.41,7.59,7.6,1

15/11/2009,06:18:28,22.16,7.84,7.8,1

15/11/2009,06:18:31,22.16,7.84,7.8,1

15/11/2009,06:18:35,19.03,10.97,11.0,1

15/11/2009,06:18:36,18.92,11.08,11.1,1

15/11/2009,06:19:05,23.12,6.88,6.9,1

15/11/2009,06:19:08,22.84,7.16,7.2,1

15/11/2009,06:19:13,22.94,7.06,7.1,1

15/11/2009,06:19:16,23.02,6.98,7.0,1

15/11/2009,06:19:39,23.66,6.34,6.3,2

15/11/2009,06:19:57,24.9,5.1,5.1,1

15/11/2009,06:19:59,24.66,5.34,5.3,1

15/11/2009,06:20:02,24.73,5.27,5.3,1

15/11/2009,06:20:29,24.73,5.27,12.5,1

15/11/2009,06:20:24,22.16,7.84,7.8,1

15/11/2009,06:20:11,17.49,12.51,5.3,1

15/11/2009,06:21:15,26.04,3.96,4.0,2

15/11/2009,06:21:13,25.97,4.03,4.0,1

15/11/2009,06:21:36,26.51,3.49,3.5,1

15/11/2009,06:21:38,26.61,3.39,3.4,1

15/11/2009,06:22:17,20.13,9.87,9.9,1

15/11/2009,06:22:17,19.92,10.08,10.1,1

15/11/2009,06:22:21,19.88,10.12,10.1,1

15/11/2009,06:22:22,19.56,10.44,10.4,1

15/11/2009,06:22:30,23.12,6.88,6.9,2

15/11/2009,06:22:49,24.87,5.13,5.1,1

15/11/2009,06:22:58,25.26,4.74,4.7,1

15/11/2009,06:23:00,25.19,4.81,4.8,1

15/11/2009,06:23:02,24.09,5.91,5.9,1

15/11/2009,06:23:03,23.87,6.13,6.1,1

15/11/2009,06:23:17,23.91,6.09,6.1,1

15/11/2009,06:23:20,23.87,6.13,6.1,1

15/11/2009,06:23:29,22.41,7.59,7.6,1

15/11/2009,06:24:52,22.73,7.27,5.3,1

15/11/2009,06:24:48,22.62,7.38,7.4,1

15/11/2009,06:23:55,24.73,5.27,7.3,1

15/11/2009,06:25:55,23.76,6.24,6.2,1

15/11/2009,06:26:07,20.7,9.3,9.3,1

15/11/2009,06:26:28,24.62,5.38,5.4,1

15/11/2009,06:27:19,21.63,8.37,8.4,1

15/11/2009,06:27:21,21.8,8.2,8.2,1

15/11/2009,06:28:11,22.34,7.66,7.7,1

15/11/2009,06:28:12,22.16,7.84,7.8,1

15/11/2009,06:29:42,23.41,6.59,15.2,1

15/11/2009,06:29:39,14.82,15.18,15.4,1

15/11/2009,06:29:39,14.57,15.43,15.7,2

15/11/2009,06:29:41,14.32,15.68,6.6,1

15/11/2009,06:32:11,23.02,6.98,7.0,1

15/11/2009,06:32:13,22.84,7.16,7.2,1

15/11/2009,06:32:48,22.84,7.16,7.2,1

15/11/2009,06:33:20,25.55,4.45,6.8,1

15/11/2009,06:33:21,25.12,4.88,4.5,1

15/11/2009,06:32:49,23.16,6.84,4.4,1

15/11/2009,06:33:21,25.65,4.35,4.7,1

15/11/2009,06:33:21,25.3,4.7,4.9,1

15/11/2009,06:33:44,25.65,4.35,4.4,1

15/11/2009,06:33:48,25.62,4.38,4.4,1

15/11/2009,06:33:58,25.83,4.17,4.2,1

15/11/2009,06:34:10,25.9,4.1,4.1,1

15/11/2009,06:34:13,26.04,3.96,4.0,1

15/11/2009,06:34:15,26.12,3.88,3.9,1

15/11/2009,06:34:20,26.22,3.78,3.8,1

15/11/2009,06:34:25,26.26,3.74,3.7,2

15/11/2009,06:34:26,26.33,3.67,3.7,1

15/11/2009,06:34:27,26.29,3.71,3.7,1

15/11/2009,06:34:37,26.4,3.6,3.6,1

15/11/2009,06:34:41,26.44,3.56,3.6,1

15/11/2009,06:34:46,26.69,3.31,3.3,1

15/11/2009,06:34:51,26.61,3.39,3.4,1

15/11/2009,06:34:51,26.65,3.35,3.4,1

15/11/2009,06:34:53,26.58,3.42,3.4,1

15/11/2009,06:43:58,20.77,9.23,9.2,1

15/11/2009,07:00:43,19.2,10.8,10.8,1

15/11/2009,07:00:46,19.2,10.8,10.8,1

15/11/2009,07:00:49,19.38,10.62,10.6,1

15/11/2009,07:05:02,17.39,12.61,12.6,1

15/11/2009,13:26:19,16.78,13.22,13.2,1

15/11/2009,13:26:20,16.6,13.4,13.4,1

15/11/2009,13:26:21,16.25,13.75,13.8,1

15/11/2009,15:21:14,26.44,3.56,3.6,1

15/11/2009,15:21:24,26.22,3.78,3.8,1

15/11/2009,16:23:32,22.98,7.02,7.0,1

15/11/2009,16:23:32,22.73,7.27,7.3,1

15/11/2009,16:23:34,22.87,7.13,7.1,1

15/11/2009,16:24:12,24.09,5.91,5.9,1

15/11/2009,16:30:02,23.02,6.98,7.0,1

15/11/2009,16:44:46,24.87,5.13,5.1,1

15/11/2009,16:44:46,24.55,5.45,5.5,1

15/11/2009,17:44:03,23.48,6.52,3.9,1

15/11/2009,17:44:04,23.19,6.81,6.5,1

15/11/2009,17:43:05,26.12,3.88,6.8,1

15/11/2009,19:09:48,26.29,3.71,3.7,1

15/11/2009,19:14:47,13.43,16.57,17.9,1

15/11/2009,19:14:43,12.15,17.85,16.6,1

15/11/2009,20:31:49,26.29,3.71,3.7,1

15/11/2009,20:31:52,26.4,3.6,3.6,1

15/11/2009,20:31:54,26.58,3.42,3.4,1

16/11/2009,00:54:09,23.42,6.58,6.0,1

16/11/2009,00:54:06,24.08,5.92,5.9,1

16/11/2009,00:54:03,23.98,6.02,6.6,1

16/11/2009,01:23:36,16.17,13.83,13.0,1

16/11/2009,01:23:34,16.14,13.86,13.3,1

16/11/2009,01:23:31,16.73,13.27,13.9,1

16/11/2009,01:23:31,17.05,12.95,13.8,1

16/11/2009,02:08:15,23.38,6.62,6.6,1

16/11/2009,02:08:33,24.5,5.5,5.5,1

16/11/2009,02:08:37,24.15,5.85,5.9,1

16/11/2009,02:09:18,25.69,4.31,4.5,1

16/11/2009,02:09:11,25.41,4.59,4.6,1

16/11/2009,02:09:09,25.55,4.45,4.3,1

16/11/2009,02:15:15,24.68,5.32,5.5,1

16/11/2009,02:15:19,25.06,4.94,5.3,1

16/11/2009,02:15:19,24.96,5.04,4.9,1

16/11/2009,02:15:12,24.5,5.5,5.0,1

16/11/2009,02:17:55,25.1,4.9,4.9,1

16/11/2009,02:17:56,25.06,4.94,4.9,1

16/11/2009,02:17:59,25.27,4.73,4.7,1

16/11/2009,02:18:02,25.59,4.41,4.4,1

16/11/2009,02:18:10,26.39,3.61,3.6,1

16/11/2009,02:18:13,26.53,3.47,3.5,1

16/11/2009,02:45:14,26.36,3.64,3.6,1

16/11/2009,02:45:14,26.04,3.96,4.0,1

16/11/2009,02:45:16,25.52,4.48,4.5,1

16/11/2009,02:45:18,24.68,5.32,5.3,1

16/11/2009,02:45:23,25.31,4.69,5.6,1

16/11/2009,02:45:24,25.2,4.8,4.7,1

16/11/2009,02:45:22,24.36,5.64,4.8,1

16/11/2009,02:45:25,23.7,6.3,6.3,1

16/11/2009,02:58:33,25.69,4.31,4.3,1

16/11/2009,03:00:45,25.38,4.62,4.6,1

16/11/2009,03:00:45,24.96,5.04,5.0,1

16/11/2009,03:03:50,26.6,3.4,3.4,1

16/11/2009,03:27:47,14.88,15.12,15.1,1

16/11/2009,03:27:52,16.56,13.44,13.4,1

16/11/2009,04:16:37,20.34,9.66,9.7,1

16/11/2009,04:16:52,21,9,9.0,1

16/11/2009,04:16:57,21.39,8.61,8.6,1

16/11/2009,04:17:24,23.24,6.76,6.8,1

16/11/2009,04:17:26,23.17,6.83,6.8,1

16/11/2009,04:23:22,17.43,12.57,12.6,1

16/11/2009,04:27:35,24.29,5.71,5.7,1

16/11/2009,04:27:38,24.47,5.53,5.5,1

16/11/2009,04:27:44,24.71,5.29,5.3,1

16/11/2009,04:27:47,24.78,5.22,5.2,1

16/11/2009,04:28:11,25.73,4.27,4.3,1

16/11/2009,04:28:09,25.69,4.31,4.3,1

16/11/2009,04:38:20,23.63,6.37,6.4,1

16/11/2009,04:39:21,25.03,4.97,5.2,1

16/11/2009,04:39:16,24.78,5.22,5.0,1

16/11/2009,04:40:08,19.32,10.68,10.7,1

16/11/2009,04:40:09,19.22,10.78,10.8,2

16/11/2009,04:40:14,19.92,10.08,10.1,1

16/11/2009,04:40:14,19.78,10.22,10.2,1

16/11/2009,04:40:15,19.85,10.15,10.2,1

16/11/2009,04:40:19,19.88,10.12,10.1,1

16/11/2009,04:40:56,21.25,8.75,8.8,1

16/11/2009,04:40:57,21.32,8.68,8.7,1

16/11/2009,04:40:59,21.39,8.61,8.6,1

16/11/2009,04:41:00,21.46,8.54,8.5,2

16/11/2009,04:41:03,21.67,8.33,8.3,2

16/11/2009,04:41:04,21.6,8.4,8.4,1

16/11/2009,04:41:56,23.17,6.83,6.8,1

16/11/2009,04:41:59,23.28,6.72,6.7,1

16/11/2009,04:42:01,23.31,6.69,6.7,1

16/11/2009,04:58:56,18.97,11.03,11.2,1

16/11/2009,04:58:54,18.8,11.2,11.0,1

16/11/2009,05:00:10,14.74,15.26,15.3,1

16/11/2009,05:00:13,14.88,15.12,15.1,1

16/11/2009,05:03:07,17.92,12.08,12.1,1

16/11/2009,05:03:34,20.86,9.14,9.1,1

16/11/2009,05:03:34,20.3,9.7,9.7,1

16/11/2009,05:05:41,26.67,3.33,3.3,1

16/11/2009,05:06:01,5.7,24.3,24.3,1

16/11/2009,05:06:05,5.88,24.12,24.1,1

16/11/2009,05:06:38,7,23,23.0,1

16/11/2009,05:08:58,20.86,9.14,9.1,1

16/11/2009,05:13:23,16.56,13.44,13.4,1

16/11/2009,05:14:06,19.22,10.78,10.8,1

16/11/2009,05:14:09,19.46,10.54,10.5,1

16/11/2009,05:14:13,19.46,10.54,10.5,1

16/11/2009,05:14:15,19.53,10.47,10.5,1

16/11/2009,05:15:19,24.89,5.11,5.1,1

16/11/2009,05:16:29,24.5,5.5,5.5,1

16/11/2009,05:16:51,24.85,5.15,5.2,1

16/11/2009,05:17:18,19.46,10.54,10.5,2

16/11/2009,05:20:12,21.7,8.3,3.3,1

16/11/2009,05:20:13,21.56,8.44,3.2,1

16/11/2009,05:20:19,21.77,8.23,8.3,1

16/11/2009,05:19:35,26.78,3.22,8.4,1

16/11/2009,05:19:34,26.74,3.26,8.2,1

16/11/2009,05:21:37,19.46,10.54,21.2,2

16/11/2009,05:21:35,19.46,10.54,10.5,1

16/11/2009,05:21:19,8.82,21.18,10.5,1

16/11/2009,05:22:16,12.67,17.33,17.3,1

16/11/2009,05:22:37,13.97,16.03,16.0,1

16/11/2009,05:26:38,21.6,8.4,8.4,1

16/11/2009,05:26:43,21.04,8.96,9.0,1

16/11/2009,05:27:46,20.37,9.63,9.6,1

16/11/2009,05:27:51,20.34,9.66,9.7,1

16/11/2009,05:27:53,20.41,9.59,9.6,1

16/11/2009,05:28:40,22.3,7.7,7.7,1

16/11/2009,05:30:10,22.09,7.91,7.9,1

16/11/2009,05:30:24,22.4,7.6,7.6,2

16/11/2009,05:30:26,22.23,7.77,7.8,1

16/11/2009,05:30:37,22.44,7.56,7.6,1

16/11/2009,05:31:06,22.05,7.95,8.0,2

16/11/2009,05:31:34,13.3,16.7,16.7,1

16/11/2009,05:31:39,12.71,17.29,17.3,1

16/11/2009,05:32:41,19.85,10.15,10.2,1

16/11/2009,05:32:45,24.19,5.81,5.8,1

16/11/2009,05:32:47,24.22,5.78,5.8,1

16/11/2009,05:33:56,21.46,8.54,8.5,1

16/11/2009,05:34:00,21.56,8.44,8.4,1

16/11/2009,05:34:02,21.7,8.3,8.3,1

16/11/2009,05:34:28,23.52,6.48,6.5,1

16/11/2009,05:34:31,23.28,6.72,6.7,1

16/11/2009,05:34:36,23.59,6.41,6.4,1

16/11/2009,05:34:54,24.15,5.85,5.9,1

16/11/2009,05:34:56,24.19,5.81,5.8,1

16/11/2009,05:35:07,24.43,5.57,5.6,2

16/11/2009,05:35:15,10.92,19.08,19.1,1

16/11/2009,05:36:01,16.38,13.62,13.6,1

16/11/2009,05:36:58,17.36,12.64,12.6,1

16/11/2009,05:37:16,25.48,4.52,4.5,1

16/11/2009,05:37:42,23.8,6.2,6.2,1

16/11/2009,05:37:46,24.5,5.5,5.5,1

16/11/2009,05:37:51,25.31,4.69,4.7,1

16/11/2009,05:40:50,20.83,9.17,9.2,1

16/11/2009,05:41:16,22.58,7.42,7.4,1

16/11/2009,05:42:30,22.61,7.39,7.4,1

16/11/2009,05:42:49,23.49,6.51,6.5,1

16/11/2009,05:43:46,18.41,11.59,11.6,1

16/11/2009,05:43:48,18.52,11.48,11.5,1

16/11/2009,05:45:28,26.01,3.99,4.0,1

16/11/2009,05:45:25,25.97,4.03,4.0,1

16/11/2009,05:45:39,26.01,3.99,4.0,1

16/11/2009,05:45:41,25.97,4.03,4.0,1

16/11/2009,05:45:54,17.12,12.88,12.9,1

16/11/2009,05:49:37,20.79,9.21,9.2,2

16/11/2009,05:49:41,20.93,9.07,9.1,1

16/11/2009,05:51:30,19.36,10.64,10.6,1

16/11/2009,05:51:46,20.44,9.56,9.6,1

16/11/2009,05:51:53,21.14,8.86,8.9,1

16/11/2009,05:52:35,19.29,10.71,10.7,1

16/11/2009,05:53:25,19.32,10.68,10.7,1

16/11/2009,05:54:07,24.29,5.71,5.7,1

16/11/2009,05:56:03,23.77,6.23,6.2,2

16/11/2009,05:56:12,23.31,6.69,6.7,1

16/11/2009,05:56:56,21.6,8.4,8.4,1

16/11/2009,05:56:57,21.35,8.65,8.7,1

16/11/2009,05:57:00,21.32,8.68,8.7,1

16/11/2009,05:57:02,21.63,8.37,8.4,1

16/11/2009,05:57:46,14.21,15.79,15.8,1

16/11/2009,05:57:53,15.61,14.39,14.4,3

16/11/2009,05:58:51,16.63,13.37,13.4,1

16/11/2009,05:58:54,16.77,13.23,13.2,3

16/11/2009,06:00:45,19.71,10.29,10.3,1

16/11/2009,06:01:01,23.98,6.02,5.5,1

16/11/2009,06:01:00,24.78,5.22,5.1,1

16/11/2009,06:01:00,24.89,5.11,5.2,1

16/11/2009,06:00:56,24.47,5.53,6.0,1

16/11/2009,06:02:45,26.04,3.96,4.0,1

16/11/2009,06:03:00,21.98,8.02,8.0,1

16/11/2009,06:03:06,20.37,9.63,9.6,1

16/11/2009,06:03:14,21.95,8.05,8.1,1

16/11/2009,06:03:27,20.51,9.49,9.5,1

16/11/2009,06:03:29,20.16,9.84,9.8,1

16/11/2009,06:03:30,20.02,9.98,10.0,1

16/11/2009,06:03:30,19.85,10.15,10.2,1

16/11/2009,06:04:54,22.16,7.84,7.8,1

16/11/2009,06:04:54,18.83,11.17,11.2,1

16/11/2009,06:05:08,22.4,7.6,7.6,1

16/11/2009,06:05:11,22.61,7.39,7.4,1

16/11/2009,06:05:22,24.29,5.71,5.7,1

16/11/2009,06:05:25,26.53,3.47,3.5,1

16/11/2009,06:06:01,17.96,12.04,12.0,1

16/11/2009,06:06:03,18.2,11.8,11.8,1

16/11/2009,06:06:04,15.93,14.07,14.1,1

16/11/2009,06:06:09,16.45,13.55,13.6,1

16/11/2009,06:06:10,16.1,13.9,13.9,1

16/11/2009,06:06:38,18.45,11.55,11.6,1

16/11/2009,06:06:45,18.34,11.66,11.7,1

16/11/2009,06:06:53,13.65,16.35,16.4,1

16/11/2009,06:07:04,16.17,13.83,13.8,1

16/11/2009,06:07:33,17.36,12.64,12.6,2

16/11/2009,06:07:36,19.32,10.68,10.7,1

16/11/2009,06:07:38,18.31,11.69,11.7,1

16/11/2009,06:07:50,15.79,14.21,14.2,1

16/11/2009,06:08:00,15.19,14.81,14.8,1

16/11/2009,06:08:17,19.5,10.5,10.5,2

16/11/2009,06:08:21,20.27,9.73,9.7,1

16/11/2009,06:08:32,20.37,9.63,9.6,1

16/11/2009,06:08:36,20.9,9.1,13.5,1

16/11/2009,06:08:38,20.76,9.24,9.1,1

16/11/2009,06:08:37,20.83,9.17,9.2,1

16/11/2009,06:08:34,16.49,13.51,9.2,1

16/11/2009,06:08:38,16.63,13.37,13.4,1

16/11/2009,06:08:53,14.98,15.02,15.0,1

16/11/2009,06:08:58,15.19,14.81,14.8,1

16/11/2009,06:09:01,14.46,15.54,15.5,1

16/11/2009,06:09:36,24.61,5.39,5.4,1

16/11/2009,06:09:39,24.89,5.11,5.1,1

16/11/2009,06:10:50,16.77,13.23,5.4,2

16/11/2009,06:10:54,24.12,5.88,5.9,1

16/11/2009,06:10:48,24.15,5.85,13.2,1

16/11/2009,06:10:48,24.64,5.36,5.9,1

16/11/2009,06:12:27,21.74,8.26,7.9,1

16/11/2009,06:12:24,22.09,7.91,8.3,1

16/11/2009,06:12:44,25.38,4.62,9.2,2

16/11/2009,06:12:31,20.76,9.24,4.6,1

16/11/2009,06:13:01,26.39,3.61,3.6,1

16/11/2009,06:13:04,26.32,3.68,6.4,1

16/11/2009,06:13:02,23.56,6.44,3.7,1

16/11/2009,06:14:27,18.34,11.66,11.7,2

16/11/2009,06:15:18,24.29,5.71,5.7,1

16/11/2009,06:15:23,21.56,8.44,8.4,1

16/11/2009,06:15:23,20.86,9.14,9.1,1

16/11/2009,06:15:26,18.1,11.9,11.9,1

16/11/2009,06:15:27,17.75,12.25,12.3,1

16/11/2009,06:15:28,17.61,12.39,12.4,1

16/11/2009,06:15:28,17.43,12.57,12.6,1

16/11/2009,06:15:35,18.34,11.66,11.7,1

16/11/2009,06:16:58,16.87,13.13,3.3,1

16/11/2009,06:17:01,17.05,12.95,4.9,1

16/11/2009,06:16:53,25.13,4.87,4.9,1

16/11/2009,06:16:49,25.13,4.87,13.1,1

16/11/2009,06:16:43,26.71,3.29,13.0,1

16/11/2009,06:17:39,17.12,12.88,12.9,1

16/11/2009,06:17:43,17.75,12.25,12.3,1

16/11/2009,06:17:48,18.2,11.8,11.8,1

16/11/2009,06:17:52,19.74,10.26,10.3,1

16/11/2009,06:18:15,22.33,7.67,11.0,1

16/11/2009,06:18:16,26.08,3.92,11.2,1

16/11/2009,06:18:16,26.36,3.64,7.7,1

16/11/2009,06:18:14,18.76,11.24,3.6,2

16/11/2009,06:18:12,19.01,10.99,3.9,1

16/11/2009,06:18:57,23.24,6.76,6.8,1

16/11/2009,06:19:26,23.07,6.93,6.5,1

16/11/2009,06:19:24,23.49,6.51,6.9,1

16/11/2009,06:19:49,24.19,5.81,5.3,1

16/11/2009,06:19:48,24.68,5.32,5.8,1

16/11/2009,06:20:07,24.96,5.04,5.0,1

16/11/2009,06:21:18,20.86,9.14,9.1,1

16/11/2009,06:21:20,20.83,9.17,9.2,1

16/11/2009,06:21:33,21.28,8.72,8.7,1

16/11/2009,06:21:34,21.28,8.72,8.7,1

16/11/2009,06:21:48,24.22,5.78,5.8,1

16/11/2009,06:22:26,20.55,9.45,9.5,1

16/11/2009,06:22:30,23.8,6.2,6.2,1

16/11/2009,06:22:27,23.8,6.2,6.2,1

16/11/2009,06:22:57,25.24,4.76,4.8,2

16/11/2009,06:22:58,24.96,5.04,5.0,1

16/11/2009,06:23:09,23.8,6.2,6.2,1

16/11/2009,06:23:12,23.91,6.09,6.1,1

16/11/2009,06:24:41,20.41,9.59,9.6,1

16/11/2009,06:24:41,18.55,11.45,11.5,1

16/11/2009,06:24:49,19.99,10.01,10.0,1

16/11/2009,06:24:52,19.81,10.19,10.2,1

16/11/2009,06:24:57,20.27,9.73,9.7,1

16/11/2009,06:25:07,20.51,9.49,9.5,1

16/11/2009,06:25:41,17.15,12.85,12.9,1

16/11/2009,06:25:42,16.73,13.27,13.3,1

16/11/2009,06:26:24,25.13,4.87,7.8,1

16/11/2009,06:26:24,24.61,5.39,4.9,1

16/11/2009,06:26:18,22.16,7.84,5.4,1

16/11/2009,06:26:31,23.31,6.69,6.7,1

16/11/2009,06:26:47,26.22,3.78,14.5,1

16/11/2009,06:26:47,26.04,3.96,14.1,1

16/11/2009,06:26:50,26.22,3.78,13.6,1

16/11/2009,06:26:50,26.04,3.96,13.1,1

16/11/2009,06:26:32,15.51,14.49,4.1,1

16/11/2009,06:26:35,15.89,14.11,3.8,1

16/11/2009,06:26:40,16.45,13.55,3.8,1

16/11/2009,06:26:45,16.91,13.09,4.0,1

16/11/2009,06:26:48,17.22,12.78,12.8,1

16/11/2009,06:26:52,17.64,12.36,3.6,1

16/11/2009,06:26:58,17.89,12.11,3.8,1

16/11/2009,06:27:04,18.24,11.76,4.0,1

16/11/2009,06:26:46,25.9,4.1,12.4,1

16/11/2009,06:26:47,26.18,3.82,12.1,1

16/11/2009,06:26:50,26.39,3.61,11.8,1

16/11/2009,06:28:24,23.66,6.34,6.3,1

16/11/2009,06:28:27,22.72,7.28,7.3,1

16/11/2009,06:30:12,15.61,14.39,14.4,1

16/11/2009,06:30:14,16.42,13.58,13.6,1

16/11/2009,06:30:15,15.75,14.25,14.3,1

16/11/2009,06:30:18,15.89,14.11,14.1,1

16/11/2009,06:30:19,15.89,14.11,14.1,1

16/11/2009,06:30:56,25.24,4.76,4.8,1

16/11/2009,06:30:57,25.27,4.73,4.7,1

16/11/2009,06:30:59,25.48,4.52,4.5,1

16/11/2009,06:31:02,25.55,4.45,4.5,1

16/11/2009,06:31:03,25.83,4.17,4.2,2

16/11/2009,06:31:12,26.71,3.29,3.3,1

16/11/2009,06:31:14,26.6,3.4,3.4,1

16/11/2009,06:31:16,26.43,3.57,3.6,1

16/11/2009,06:31:47,21.11,8.89,8.9,1

16/11/2009,06:31:49,20.55,9.45,9.5,1

16/11/2009,06:31:50,20.23,9.77,9.8,1

16/11/2009,06:33:36,20.37,9.63,9.6,1

16/11/2009,06:33:48,22.54,7.46,7.3,1

16/11/2009,06:33:44,22.72,7.28,7.5,1

16/11/2009,06:34:30,24.99,5.01,5.0,1

16/11/2009,06:34:33,24.96,5.04,5.0,1

16/11/2009,06:34:36,25.17,4.83,4.8,1

16/11/2009,06:34:39,25.34,4.66,4.7,1

16/11/2009,06:34:40,25.38,4.62,4.6,1

16/11/2009,06:34:40,25.38,4.62,4.6,1

16/11/2009,06:34:52,25.45,4.55,4.6,1

16/11/2009,06:34:54,25.06,4.94,4.9,1

16/11/2009,06:39:16,26.64,3.36,3.6,1

16/11/2009,06:39:10,26.43,3.57,3.4,1

16/11/2009,06:39:25,26.46,3.54,3.5,1

16/11/2009,06:39:25,26.32,3.68,3.7,1

16/11/2009,06:39:47,26.43,3.57,3.6,1

16/11/2009,06:50:31,25.38,4.62,4.6,1

16/11/2009,14:31:23,25.9,4.1,3.6,1

16/11/2009,14:31:22,25.9,4.1,3.8,1

16/11/2009,14:31:13,26.25,3.75,4.1,1

16/11/2009,14:31:12,26.39,3.61,4.1,1

16/11/2009,14:35:55,26.74,3.26,3.2,1

16/11/2009,14:35:54,26.78,3.22,3.3,1

16/11/2009,14:54:38,24.89,5.11,5.1,1

16/11/2009,14:54:41,25.06,4.94,4.9,1

16/11/2009,15:06:23,26.39,3.61,3.6,1

16/11/2009,15:06:30,26.18,3.82,3.8,1

16/11/2009,15:17:01,26.71,3.29,2.9,1

16/11/2009,15:16:59,26.74,3.26,3.1,1

16/11/2009,15:16:55,26.92,3.08,3.3,1

16/11/2009,15:16:55,27.13,2.87,3.3,1

16/11/2009,15:17:33,26.43,3.57,3.6,1

16/11/2009,15:17:35,26.22,3.78,3.8,1

16/11/2009,15:17:39,26.64,3.36,3.4,1

16/11/2009,15:18:32,25.73,4.27,3.9,1

16/11/2009,15:18:25,26.08,3.92,6.9,2

16/11/2009,15:18:25,23.1,6.9,4.3,1

16/11/2009,15:18:41,25.24,4.76,4.8,1

16/11/2009,15:25:56,26.22,3.78,3.8,1

16/11/2009,15:26:06,26.15,3.85,3.9,1

16/11/2009,15:26:08,26.15,3.85,3.8,1

16/11/2009,15:26:09,26.04,3.96,3.9,1

16/11/2009,15:26:08,26.25,3.75,4.0,1

16/11/2009,15:56:10,23.21,6.79,6.8,1

16/11/2009,15:56:23,21.46,8.54,8.5,1

16/11/2009,16:31:09,25.9,4.1,4.1,1

16/11/2009,16:31:12,25.9,4.1,4.1,1

16/11/2009,16:31:37,26.43,3.57,3.6,1

16/11/2009,16:31:39,26.36,3.64,3.6,1

16/11/2009,16:31:43,26.43,3.57,3.6,1

16/11/2009,16:31:46,26.5,3.5,3.5,1

16/11/2009,16:58:17,21.25,8.75,8.8,1

16/11/2009,16:58:19,21.46,8.54,8.5,2

16/11/2009,17:01:39,27.09,2.91,2.9,1

16/11/2009,17:10:00,23.35,6.65,7.3,2

16/11/2009,17:09:52,22.68,7.32,7.3,1

16/11/2009,17:09:51,22.72,7.28,6.7,1

16/11/2009,17:18:50,21,9,9.0,1

16/11/2009,17:18:56,19.71,10.29,10.3,1

16/11/2009,18:14:55,26.6,3.4,3.4,1

16/11/2009,18:25:50,26.92,3.08,3.1,1

16/11/2009,19:36:38,26.71,3.29,3.3,1

16/11/2009,20:53:46,4.27,25.73,25.7,1

16/11/2009,20:53:46,4.13,25.87,25.9,1

16/11/2009,20:53:47,4.2,25.8,25.8,1

16/11/2009,22:06:21,26.11,3.89,3.9,1

16/11/2009,23:05:50,11.94,18.06,18.1,1

16/11/2009,23:16:25,11.83,18.17,18.2,1

16/11/2009,23:16:22,11.83,18.17,18.2,1

17/11/2009,00:43:37,9.13,20.87,20.8,1

17/11/2009,00:43:36,9.24,20.76,20.9,1

17/11/2009,01:36:32,18.31,11.69,11.7,1

17/11/2009,02:21:27,16.73,13.27,13.3,1

17/11/2009,02:31:32,24.22,5.78,5.8,1

17/11/2009,02:31:37,23.87,6.13,6.1,1

17/11/2009,02:31:39,23.84,6.16,6.2,1

17/11/2009,02:31:41,23.91,6.09,6.1,1

17/11/2009,02:31:44,23.94,6.06,6.1,1

17/11/2009,02:32:01,26.22,3.78,3.2,1

17/11/2009,02:32:01,26.36,3.64,3.6,1

17/11/2009,02:31:58,26.78,3.22,3.8,1

17/11/2009,02:49:50,7.42,22.58,22.6,1

17/11/2009,02:50:59,10.71,19.29,19.3,1

17/11/2009,02:52:49,26.95,3.05,3.4,1

17/11/2009,02:52:47,26.78,3.22,3.4,1

17/11/2009,02:52:44,26.67,3.33,3.3,1

17/11/2009,02:52:43,26.64,3.36,3.2,1

17/11/2009,02:52:42,26.64,3.36,3.1,1

17/11/2009,02:53:15,24.61,5.39,4.9,1

17/11/2009,02:53:14,25.06,4.94,5.4,1

17/11/2009,03:02:10,19.95,10.05,10.2,1

17/11/2009,03:02:03,19.81,10.19,10.1,1

17/11/2009,03:09:16,16.14,13.86,13.9,1

17/11/2009,03:09:14,16.1,13.9,13.9,1

17/11/2009,03:12:55,16.98,13.02,13.0,2

17/11/2009,03:12:54,17.05,12.95,13.0,1

17/11/2009,03:26:30,26.15,3.85,3.9,1

17/11/2009,03:26:38,26.18,3.82,3.8,2

17/11/2009,03:29:27,16.24,13.76,13.8,1

17/11/2009,03:29:31,16.42,13.58,13.6,1

17/11/2009,03:29:32,16.38,13.62,13.6,1

17/11/2009,03:29:34,16.35,13.65,13.7,1

17/11/2009,03:29:35,16.28,13.72,13.7,1

17/11/2009,03:41:08,19.32,10.68,10.3,1

17/11/2009,03:41:07,19.74,10.26,10.7,1

17/11/2009,03:45:28,19.74,10.26,10.2,1

17/11/2009,03:45:25,19.81,10.19,10.3,1

17/11/2009,03:52:32,26.01,3.99,4.0,1

17/11/2009,03:52:34,18.62,11.38,11.4,1

17/11/2009,03:57:29,13.27,16.73,16.7,1

17/11/2009,03:57:49,15.16,14.84,14.8,1

17/11/2009,03:57:52,15.4,14.6,14.6,1

17/11/2009,04:04:23,19.95,10.05,10.1,1

17/11/2009,04:04:59,19.71,10.29,10.4,1

17/11/2009,04:04:58,19.57,10.43,10.3,1

17/11/2009,04:05:58,17.85,12.15,12.2,1

17/11/2009,04:08:07,7.98,22.02,21.9,1

17/11/2009,04:08:02,8.15,21.85,22.0,2

17/11/2009,04:09:05,10.08,19.92,20.0,1

17/11/2009,04:09:00,10.04,19.96,19.9,1

17/11/2009,04:09:24,22.26,7.74,8.0,1

17/11/2009,04:09:20,21.98,8.02,7.7,1

17/11/2009,04:10:37,7.7,22.3,22.0,1

17/11/2009,04:10:37,7.98,22.02,22.3,2

17/11/2009,04:14:11,22.4,7.6,7.6,1

17/11/2009,04:14:10,22.44,7.56,7.6,1

17/11/2009,04:15:03,15.47,14.53,14.5,1

17/11/2009,04:18:17,12.25,17.75,17.8,1

17/11/2009,04:18:34,16.07,13.93,13.9,1

17/11/2009,04:18:33,16.14,13.86,13.9,1

17/11/2009,04:18:55,16.87,13.13,13.1,1

17/11/2009,04:18:56,16.73,13.27,13.3,1

17/11/2009,04:18:57,16.77,13.23,13.2,2

17/11/2009,04:19:01,17.12,12.88,12.9,1

17/11/2009,04:19:05,16.84,13.16,13.2,1

17/11/2009,04:19:30,14.39,15.61,15.6,1

17/11/2009,04:19:42,17.5,12.5,12.5,1

17/11/2009,04:19:57,20.9,9.1,9.1,1

17/11/2009,04:32:33,12.25,17.75,17.8,1

17/11/2009,04:34:29,26.36,3.64,22.2,1

17/11/2009,04:33:28,7.77,22.23,22.2,1

17/11/2009,04:33:32,7.77,22.23,22.6,1

17/11/2009,04:33:33,7.42,22.58,3.6,1

17/11/2009,04:36:22,18.06,11.94,11.9,1

17/11/2009,04:36:26,18.24,11.76,11.8,1

17/11/2009,04:36:30,18.66,11.34,11.3,1

17/11/2009,04:44:08,14.95,15.05,15.3,1

17/11/2009,04:44:05,14.74,15.26,15.1,1

17/11/2009,04:44:12,17.33,12.67,12.7,1

17/11/2009,04:51:03,18.34,11.66,11.7,1

17/11/2009,04:51:46,18.52,11.48,11.6,1

17/11/2009,04:51:45,18.45,11.55,11.5,1

17/11/2009,04:52:08,18.69,11.31,11.3,1

17/11/2009,04:52:25,11.97,18.03,18.0,1

17/11/2009,04:53:04,21.74,8.26,8.3,1

17/11/2009,04:53:29,16.49,13.51,13.5,1

17/11/2009,04:55:54,20.51,9.49,9.6,1

17/11/2009,04:55:52,20.44,9.56,9.5,1

17/11/2009,04:56:14,20.69,9.31,9.3,1

17/11/2009,04:58:13,17.4,12.6,12.6,1

17/11/2009,04:58:16,17.36,12.64,12.6,1

17/11/2009,04:58:23,19.32,10.68,10.7,1

17/11/2009,04:58:28,19.64,10.36,10.4,1

17/11/2009,04:58:45,17.43,12.57,12.3,1

17/11/2009,04:58:43,17.68,12.32,12.6,1

17/11/2009,04:59:30,18.55,11.45,11.5,1

17/11/2009,04:59:30,18.27,11.73,11.7,1

17/11/2009,04:59:34,18.31,11.69,11.7,1

17/11/2009,04:59:36,18.2,11.8,11.8,1

17/11/2009,04:59:37,18.24,11.76,11.8,1

17/11/2009,04:59:39,18.27,11.73,11.7,1

17/11/2009,05:00:34,18.8,11.2,11.2,1

17/11/2009,05:00:36,18.8,11.2,11.2,1

17/11/2009,05:00:51,12.39,17.61,17.6,1

17/11/2009,05:00:52,12.11,17.89,17.9,1

17/11/2009,05:01:17,20.44,9.56,9.6,1

17/11/2009,05:01:19,20.55,9.45,9.5,1

17/11/2009,05:02:39,22.65,7.35,7.4,1

17/11/2009,05:02:38,22.61,7.39,7.4,1

17/11/2009,05:03:32,20.72,9.28,9.3,1

17/11/2009,05:05:02,24.75,5.25,5.3,1

17/11/2009,05:05:04,24.85,5.15,5.2,1

17/11/2009,05:05:12,26.04,3.96,4.0,2

17/11/2009,05:06:02,24.33,5.67,4.8,1

17/11/2009,05:06:02,24.54,5.46,4.9,2

17/11/2009,05:06:01,24.82,5.18,5.2,1

17/11/2009,05:06:00,25.06,4.94,5.5,1

17/11/2009,05:06:00,25.17,4.83,5.7,1

17/11/2009,05:09:43,19.64,10.36,10.4,1

17/11/2009,05:09:45,20.48,9.52,9.5,1

17/11/2009,05:10:08,26.43,3.57,3.6,1

17/11/2009,05:16:11,11.38,18.62,18.6,1

17/11/2009,05:16:15,12.01,17.99,18.0,2

17/11/2009,05:21:06,18.66,11.34,11.3,1

17/11/2009,05:21:12,20.3,9.7,9.7,1

17/11/2009,05:23:02,26.64,3.36,3.4,2

17/11/2009,05:24:50,21.67,8.33,8.3,1

17/11/2009,05:25:08,22.96,7.04,13.3,1

17/11/2009,05:25:10,22.79,7.21,7.0,1

17/11/2009,05:25:06,16.7,13.3,7.2,2

17/11/2009,05:27:52,23.17,6.83,6.8,2

17/11/2009,05:27:55,23,7,7.0,1

17/11/2009,05:28:22,23.91,6.09,10.2,1

17/11/2009,05:28:21,19.78,10.22,10.2,1

17/11/2009,05:28:19,19.78,10.22,6.1,1

17/11/2009,05:29:50,19.57,10.43,10.4,1

17/11/2009,05:29:56,20.06,9.94,9.9,1

17/11/2009,05:29:58,20.02,9.98,10.0,1

17/11/2009,05:29:59,20.16,9.84,9.8,1

17/11/2009,05:30:00,20.9,9.1,9.1,1

17/11/2009,05:30:08,19.18,10.82,10.8,2

17/11/2009,05:30:14,19.25,10.75,10.8,4

17/11/2009,05:31:11,24.5,5.5,5.5,1

17/11/2009,05:32:00,19.64,10.36,10.7,3

17/11/2009,05:31:54,19.29,10.71,9.9,1

17/11/2009,05:31:55,20.06,9.94,10.4,1

17/11/2009,05:32:13,17.99,12.01,12.0,1

17/11/2009,05:32:23,20.09,9.91,9.9,1

17/11/2009,05:32:26,20.09,9.91,9.9,1

17/11/2009,05:32:29,20.62,9.38,9.4,1

17/11/2009,05:32:57,24.61,5.39,5.4,1

17/11/2009,05:33:03,22.4,7.6,7.6,1

17/11/2009,05:33:06,22.58,7.42,7.4,1

17/11/2009,05:33:17,22.75,7.25,7.3,1

17/11/2009,05:33:22,22.96,7.04,7.0,1

17/11/2009,05:33:31,24.75,5.25,5.3,1

17/11/2009,05:33:44,25.24,4.76,12.6,1

17/11/2009,05:33:44,24.01,5.99,4.8,1

17/11/2009,05:33:42,17.4,12.6,6.0,1

17/11/2009,05:33:58,16.03,13.97,14.0,2

17/11/2009,05:34:20,16.94,13.06,13.1,1

17/11/2009,05:34:42,12.01,17.99,17.9,3

17/11/2009,05:34:42,12.11,17.89,18.0,1

17/11/2009,05:35:25,23.28,6.72,21.0,2

17/11/2009,05:35:27,23.28,6.72,6.7,1

17/11/2009,05:35:16,8.96,21.04,6.7,1

17/11/2009,05:35:49,17.61,12.39,17.8,1

17/11/2009,05:35:36,12.18,17.82,12.4,1

17/11/2009,05:35:50,15.68,14.32,14.3,1

17/11/2009,05:35:54,16.14,13.86,13.9,1

17/11/2009,05:36:05,19.32,10.68,10.7,1

17/11/2009,05:36:07,19.29,10.71,10.7,1

17/11/2009,05:36:18,19.88,10.12,8.0,1

17/11/2009,05:36:19,19.92,10.08,15.0,1

17/11/2009,05:36:23,19.99,10.01,10.1,1

17/11/2009,05:36:26,19.99,10.01,10.1,1

17/11/2009,05:36:30,20.34,9.66,10.0,1

17/11/2009,05:36:16,14.98,15.02,10.0,2

17/11/2009,05:36:15,22.05,7.95,9.7,1

17/11/2009,05:37:22,16.49,13.51,13.5,1

17/11/2009,05:38:33,24.54,5.46,5.5,1

17/11/2009,05:38:57,25.94,4.06,4.1,1

17/11/2009,05:39:12,21.42,8.58,8.6,1

17/11/2009,05:39:14,18.83,11.17,11.2,2

17/11/2009,05:39:12,18.76,11.24,11.2,1

17/11/2009,05:40:10,17.29,12.71,12.8,1

17/11/2009,05:40:08,17.19,12.81,12.7,1

17/11/2009,05:40:31,24.19,5.81,5.8,1

17/11/2009,05:40:35,19.74,10.26,10.3,1

17/11/2009,05:40:37,19.46,10.54,10.5,1

17/11/2009,05:40:37,18.24,11.76,11.8,1

17/11/2009,05:41:35,14,16,16.0,1

17/11/2009,05:42:13,15.44,14.56,14.6,1

17/11/2009,05:42:12,15.37,14.63,14.6,1

17/11/2009,05:43:55,18.34,11.66,11.7,1

17/11/2009,05:43:54,18.31,11.69,11.7,1

17/11/2009,05:44:25,26.08,3.92,3.9,1

17/11/2009,05:44:28,22.09,7.91,7.9,1

17/11/2009,05:44:29,22.33,7.67,7.7,1

17/11/2009,05:44:31,22.58,7.42,7.4,1

17/11/2009,05:44:31,22.26,7.74,7.7,1

17/11/2009,05:44:34,22.44,7.56,7.6,1

17/11/2009,05:44:39,22.61,7.39,7.4,1

17/11/2009,05:44:40,22.68,7.32,7.3,1

17/11/2009,05:45:08,18.1,11.9,11.9,1

17/11/2009,05:45:11,18.31,11.69,14.3,1

17/11/2009,05:45:10,15.23,14.77,14.8,1

17/11/2009,05:45:09,15.72,14.28,11.7,1

17/11/2009,05:45:41,12.15,17.85,17.7,1

17/11/2009,05:45:38,12.29,17.71,17.9,1

17/11/2009,05:46:02,23.31,6.69,6.7,1

17/11/2009,05:46:42,24.57,5.43,5.4,1

17/11/2009,05:47:29,23.42,6.58,6.6,1

17/11/2009,05:47:33,23.45,6.55,6.6,1

17/11/2009,05:47:39,24.57,5.43,5.4,1

17/11/2009,05:48:27,19.6,10.4,10.4,1

17/11/2009,05:49:32,23.38,6.62,6.6,2

17/11/2009,05:49:40,22.65,7.35,7.4,1

17/11/2009,05:50:04,24.61,5.39,5.4,1

17/11/2009,05:50:05,24.33,5.67,5.7,1

17/11/2009,05:50:07,21.39,8.61,8.6,1

17/11/2009,05:50:09,21.53,8.47,8.5,1

17/11/2009,05:50:19,24.43,5.57,8.1,1

17/11/2009,05:50:15,21.88,8.12,5.6,1

17/11/2009,05:50:22,21.95,8.05,8.1,1

17/11/2009,05:50:42,24.57,5.43,11.7,1

17/11/2009,05:50:45,23.52,6.48,5.4,1

17/11/2009,05:50:44,21.95,8.05,8.1,1

17/11/2009,05:50:40,18.27,11.73,6.5,1

17/11/2009,05:50:57,22.86,7.14,7.0,1

17/11/2009,05:50:56,23.03,6.97,7.1,1

17/11/2009,05:51:00,22.89,7.11,7.1,1

17/11/2009,05:51:02,17.99,12.01,12.0,1

17/11/2009,05:51:23,14.46,15.54,15.5,1

17/11/2009,05:51:42,19.15,10.85,10.9,1

17/11/2009,05:51:47,18.55,11.45,11.5,1

17/11/2009,05:51:59,20.83,9.17,9.2,1

17/11/2009,05:52:00,21.56,8.44,8.4,1

17/11/2009,05:52:01,21.84,8.16,8.2,1

17/11/2009,05:52:30,21.25,8.75,8.8,1

17/11/2009,05:52:37,22.09,7.91,7.9,1

17/11/2009,05:52:40,21.53,8.47,8.5,1

17/11/2009,05:56:30,22.44,7.56,7.5,1

17/11/2009,05:56:28,22.47,7.53,7.6,2

17/11/2009,05:56:41,19.81,10.19,10.1,1

17/11/2009,05:56:40,19.95,10.05,10.2,1

17/11/2009,05:56:46,19.99,10.01,10.0,1

17/11/2009,05:56:49,20.27,9.73,9.7,1

17/11/2009,05:57:30,26.5,3.5,3.5,1

17/11/2009,05:57:42,19.64,10.36,12.9,1

17/11/2009,05:57:40,17.08,12.92,10.4,1

17/11/2009,05:57:42,17.29,12.71,12.7,1

17/11/2009,05:58:01,18.31,11.69,11.7,1

17/11/2009,05:58:48,18.94,11.06,10.8,1

17/11/2009,05:58:48,19.22,10.78,11.1,1

17/11/2009,06:00:02,22.68,7.32,7.3,1

17/11/2009,06:00:04,22.82,7.18,7.2,1

17/11/2009,06:00:36,20.3,9.7,9.7,1

17/11/2009,06:00:38,20.3,9.7,9.7,1

17/11/2009,06:00:40,20.44,9.56,9.6,1

17/11/2009,06:00:48,21.81,8.19,8.2,1

17/11/2009,06:00:50,21.88,8.12,8.1,1

17/11/2009,06:00:51,21.84,8.16,8.2,1

17/11/2009,06:00:51,21.42,8.58,8.6,1

17/11/2009,06:01:17,25.17,4.83,4.8,1

17/11/2009,06:01:19,25.34,4.66,4.7,1

17/11/2009,06:03:26,22.82,7.18,7.2,1

17/11/2009,06:03:26,22.37,7.63,7.6,1

17/11/2009,06:03:27,20.58,9.42,9.4,1

17/11/2009,06:04:12,19.85,10.15,10.2,2

17/11/2009,06:05:10,25.87,4.13,8.1,1

17/11/2009,06:05:05,21.95,8.05,4.1,3

17/11/2009,06:05:10,22.75,7.25,7.3,1

17/11/2009,06:06:00,25.59,4.41,4.4,1

17/11/2009,06:06:00,24.64,5.36,5.4,1

17/11/2009,06:06:05,24.64,5.36,5.4,1

17/11/2009,06:06:10,22.58,7.42,7.4,1

17/11/2009,06:06:17,23.1,6.9,6.9,1

17/11/2009,06:07:00,24.5,5.5,5.5,1

17/11/2009,06:07:03,24.4,5.6,5.6,2

17/11/2009,06:07:29,23.8,6.2,6.2,1

17/11/2009,06:07:33,23.66,6.34,6.3,1

17/11/2009,06:07:56,21.74,8.26,8.3,1

17/11/2009,06:08:03,22.05,7.95,8.0,1

17/11/2009,06:08:08,22.47,7.53,7.5,1

17/11/2009,06:08:10,22.47,7.53,16.9,1

17/11/2009,06:08:10,12.11,17.89,7.5,1

17/11/2009,06:08:10,12.88,17.12,17.0,1

17/11/2009,06:08:10,13.02,16.98,17.1,1

17/11/2009,06:08:08,13.09,16.91,17.9,1

17/11/2009,06:08:39,21.6,8.4,7.8,1

17/11/2009,06:08:39,21.74,8.26,8.1,2

17/11/2009,06:08:39,21.95,8.05,8.3,1

17/11/2009,06:08:38,22.19,7.81,8.4,1

17/11/2009,06:09:17,19.67,10.33,10.3,1

17/11/2009,06:09:30,25.62,4.38,4.3,1

17/11/2009,06:09:28,25.69,4.31,4.4,1

17/11/2009,06:09:40,21.25,8.75,8.8,1

17/11/2009,06:09:44,21.35,8.65,8.7,1

17/11/2009,06:09:45,21.81,8.19,8.2,1

17/11/2009,06:10:24,24.19,5.81,5.8,1

17/11/2009,06:10:27,24.43,5.57,5.6,1

17/11/2009,06:10:45,24.78,5.22,5.2,1

17/11/2009,06:11:18,24.43,5.57,5.6,1

17/11/2009,06:11:24,24.33,5.67,5.7,1

17/11/2009,06:11:25,24.26,5.74,5.7,1

17/11/2009,06:11:28,24.26,5.74,5.7,1

17/11/2009,06:11:30,24.36,5.64,5.6,1

17/11/2009,06:12:00,25.06,4.94,4.9,1

17/11/2009,06:12:19,21.04,8.96,8.8,1

17/11/2009,06:12:19,21.21,8.79,9.0,1

17/11/2009,06:12:33,24.78,5.22,5.2,1

17/11/2009,06:12:47,22.68,7.32,7.3,1

17/11/2009,06:12:49,22.79,7.21,10.3,1

17/11/2009,06:12:47,19.01,10.99,11.0,1

17/11/2009,06:12:47,19.67,10.33,7.2,1

17/11/2009,06:12:52,17.96,12.04,12.0,1

17/11/2009,06:12:52,17.29,12.71,12.7,1

17/11/2009,06:13:38,24.57,5.43,7.0,1

17/11/2009,06:13:39,23.03,6.97,5.4,1

17/11/2009,06:13:37,23.03,6.97,7.0,1

17/11/2009,06:13:41,22.26,7.74,7.7,1

17/11/2009,06:13:45,23.07,6.93,6.9,1

17/11/2009,06:14:27,25.59,4.41,4.4,1

17/11/2009,06:14:29,22.47,7.53,7.5,1

17/11/2009,06:14:45,22.4,7.6,7.3,1

17/11/2009,06:14:44,22.75,7.25,7.6,1

17/11/2009,06:14:51,21.95,8.05,9.9,1

17/11/2009,06:14:52,19.6,10.4,9.8,1

17/11/2009,06:14:53,20.41,9.59,8.1,1

17/11/2009,06:14:50,20.23,9.77,10.4,1

17/11/2009,06:14:49,20.13,9.87,9.6,1

17/11/2009,06:14:56,19.22,10.78,11.0,1

17/11/2009,06:14:53,19.01,10.99,10.8,2

17/11/2009,06:15:28,23.21,6.79,13.0,1

17/11/2009,06:15:30,23.45,6.55,12.7,1

17/11/2009,06:15:18,17.26,12.74,6.8,1

17/11/2009,06:15:15,17.05,12.95,6.6,1

17/11/2009,06:15:50,22.54,7.46,7.5,1

17/11/2009,06:15:50,22.4,7.6,7.6,1

17/11/2009,06:15:57,22.26,7.74,7.7,1

17/11/2009,06:16:00,19.67,10.33,10.3,1

17/11/2009,06:16:20,17.64,12.36,12.4,1

17/11/2009,06:16:23,17.4,12.6,12.6,1

17/11/2009,06:16:25,17.43,12.57,12.6,1

17/11/2009,06:16:49,21.67,8.33,8.3,1

17/11/2009,06:16:53,21.84,8.16,8.2,1

17/11/2009,06:16:54,22.02,7.98,8.0,1

17/11/2009,06:16:56,22.02,7.98,8.0,1

17/11/2009,06:16:58,21.84,8.16,10.7,1

17/11/2009,06:16:57,19.32,10.68,8.2,1

17/11/2009,06:16:59,18.69,11.31,11.3,1

17/11/2009,06:17:02,19.11,10.89,10.9,1

17/11/2009,06:17:04,19.36,10.64,10.6,1

17/11/2009,06:17:17,21.28,8.72,16.0,1

17/11/2009,06:17:14,21.11,8.89,8.9,1

17/11/2009,06:17:15,21.77,8.23,8.2,1

17/11/2009,06:17:09,14,16,8.7,1

17/11/2009,06:17:37,20.51,9.49,9.5,1

17/11/2009,06:17:41,20.79,9.21,9.2,1

17/11/2009,06:17:45,21,9,9.0,1

17/11/2009,06:18:07,23.66,6.34,9.6,1

17/11/2009,06:18:09,23.84,6.16,9.9,1

17/11/2009,06:18:05,20.09,9.91,6.3,1

17/11/2009,06:18:03,20.41,9.59,6.2,1

17/11/2009,06:18:20,20.76,9.24,9.2,1

17/11/2009,06:18:21,23.84,6.16,6.2,1

17/11/2009,06:18:32,23.98,6.02,6.0,1

17/11/2009,06:18:37,24.05,5.95,6.0,1

17/11/2009,06:18:47,21.04,8.96,9.0,1

17/11/2009,06:19:12,21.28,8.72,8.5,1

17/11/2009,06:19:10,21.49,8.51,8.7,1

17/11/2009,06:19:29,24.68,5.32,14.8,1

17/11/2009,06:19:28,24.68,5.32,15.3,1

17/11/2009,06:19:32,24.85,5.15,15.4,1

17/11/2009,06:19:21,14.35,15.65,15.7,1

17/11/2009,06:19:19,14.35,15.65,15.7,1

17/11/2009,06:19:15,14.56,15.44,5.3,1

17/11/2009,06:19:14,14.74,15.26,5.3,1

17/11/2009,06:19:14,15.19,14.81,5.2,1

17/11/2009,06:19:57,18.66,11.34,11.5,1

17/11/2009,06:19:54,18.48,11.52,11.3,1

17/11/2009,06:20:05,19.01,10.99,11.0,1

17/11/2009,06:20:12,19.04,10.96,11.0,1

17/11/2009,06:22:04,21.81,8.19,8.2,1

17/11/2009,06:22:30,25.17,4.83,7.2,1

17/11/2009,06:22:28,22.79,7.21,4.8,1

17/11/2009,06:22:56,23.42,6.58,6.6,1

17/11/2009,06:23:02,24.01,5.99,6.0,1

17/11/2009,06:23:16,24.71,5.29,5.3,1

17/11/2009,06:23:26,22.86,7.14,7.1,1

17/11/2009,06:23:33,23.31,6.69,6.7,1

17/11/2009,06:23:59,21.56,8.44,8.4,1

17/11/2009,06:24:33,20.83,9.17,9.2,2

17/11/2009,06:24:27,20.76,9.24,9.2,1

17/11/2009,06:25:00,21.77,8.23,8.2,1

17/11/2009,06:25:08,21.46,8.54,8.5,1

17/11/2009,06:25:11,21.39,8.61,8.6,1

17/11/2009,06:25:16,21.46,8.54,8.5,1

17/11/2009,06:25:28,21.67,8.33,8.3,1

17/11/2009,06:27:08,22.19,7.81,7.8,2

17/11/2009,06:27:08,19.08,10.92,10.9,1

17/11/2009,06:29:08,20.41,9.59,9.6,1

17/11/2009,06:29:11,20.34,9.66,9.7,1

17/11/2009,06:29:13,20.09,9.91,9.9,2

17/11/2009,06:29:15,19.85,10.15,10.2,1

17/11/2009,06:29:37,19.43,10.57,10.6,1

17/11/2009,06:31:41,20.97,9.03,9.0,1

17/11/2009,06:31:59,24.05,5.95,6.0,1

17/11/2009,06:32:01,23.8,6.2,6.2,1

17/11/2009,06:32:11,24.05,5.95,6.0,1

17/11/2009,06:32:21,24.08,5.92,5.9,1

17/11/2009,06:33:15,24.47,5.53,5.5,1

17/11/2009,06:33:44,21.14,8.86,8.9,1

17/11/2009,06:33:49,18.41,11.59,3.4,1

17/11/2009,06:34:05,17.29,12.71,11.6,2

17/11/2009,06:33:45,26.6,3.4,12.7,1

17/11/2009,06:34:52,24.57,5.43,7.0,1

17/11/2009,06:34:36,23,7,5.4,1

17/11/2009,06:35:02,21.11,8.89,8.9,1

17/11/2009,06:35:10,21,9,9.0,1

17/11/2009,06:36:55,23.87,6.13,6.1,1

17/11/2009,06:36:59,24.01,5.99,6.0,1

17/11/2009,06:37:00,22.3,7.7,7.7,1

17/11/2009,06:37:07,21.74,8.26,8.3,1

17/11/2009,06:37:09,21.46,8.54,8.5,1

17/11/2009,06:37:20,21.56,8.44,8.4,1

17/11/2009,06:37:32,24.15,5.85,5.6,1

17/11/2009,06:37:32,24.43,5.57,5.9,1

17/11/2009,06:37:52,22.93,7.07,6.8,1

17/11/2009,06:37:51,23.1,6.9,6.7,1

17/11/2009,06:37:51,23.24,6.76,6.8,1

17/11/2009,06:37:51,23.28,6.72,6.9,1

17/11/2009,06:37:50,23.24,6.76,7.1,2

17/11/2009,06:38:06,24.68,5.32,5.3,1

17/11/2009,06:38:31,24.57,5.43,5.4,1

17/11/2009,06:39:16,23.8,6.2,6.2,1

17/11/2009,06:39:19,23.73,6.27,6.3,1

17/11/2009,06:39:31,24.22,5.78,5.8,1

17/11/2009,06:39:34,21.63,8.37,8.4,1

17/11/2009,06:39:37,21.84,8.16,8.0,1

17/11/2009,06:39:37,22.02,7.98,8.2,2

17/11/2009,06:40:27,22.89,7.11,7.1,1

17/11/2009,06:40:33,23.03,6.97,7.0,1

17/11/2009,06:40:51,26.11,3.89,3.7,1

17/11/2009,06:40:50,26.29,3.71,3.9,1

17/11/2009,06:42:30,23.42,6.58,6.6,1

17/11/2009,06:42:29,23.42,6.58,6.6,1

17/11/2009,06:45:02,22.02,7.98,7.6,1

17/11/2009,06:45:01,22.3,7.7,7.7,1

17/11/2009,06:45:00,22.44,7.56,8.0,1

17/11/2009,06:54:45,22.51,7.49,7.3,1

17/11/2009,06:54:44,22.75,7.25,7.5,1

17/11/2009,06:56:12,25.59,4.41,4.4,2

17/11/2009,06:56:12,25.62,4.38,4.4,1

17/11/2009,06:56:18,25.83,4.17,4.2,1

17/11/2009,06:56:23,26.01,3.99,4.0,1

17/11/2009,06:56:26,25.94,4.06,4.1,1

17/11/2009,06:56:33,26.57,3.43,3.4,1

17/11/2009,06:56:34,26.71,3.29,3.3,1

17/11/2009,06:56:35,26.71,3.29,3.3,1

17/11/2009,06:58:15,24.19,5.81,5.8,1

17/11/2009,06:58:17,24.22,5.78,5.8,1

17/11/2009,06:58:18,24.12,5.88,5.9,2

17/11/2009,06:58:25,24.4,5.6,5.6,1

17/11/2009,06:58:29,24.4,5.6,5.6,1

17/11/2009,06:58:33,24.47,5.53,5.5,1

17/11/2009,06:58:35,24.43,5.57,5.6,1

17/11/2009,06:58:45,24.82,5.18,5.2,1

17/11/2009,06:58:48,24.85,5.15,5.2,1

17/11/2009,06:58:49,24.99,5.01,5.0,1

17/11/2009,06:58:52,24.96,5.04,5.0,1

17/11/2009,06:58:56,25.03,4.97,5.0,1

17/11/2009,06:59:03,25.1,4.9,4.9,1

17/11/2009,06:59:04,24.75,5.25,5.3,1

17/11/2009,07:06:38,24.43,5.57,5.6,1

17/11/2009,07:06:44,24.82,5.18,5.2,1

17/11/2009,07:06:47,25.17,4.83,4.8,2

17/11/2009,07:06:49,25.06,4.94,4.9,1

17/11/2009,07:06:51,25.13,4.87,4.9,1

17/11/2009,07:06:52,25.03,4.97,5.0,2

17/11/2009,07:32:43,24.78,5.22,5.0,1

17/11/2009,07:32:42,24.99,5.01,5.2,1

17/11/2009,07:32:59,24.96,5.04,5.0,1

17/11/2009,15:54:36,24.05,5.95,6.0,1

17/11/2009,15:54:39,24.01,5.99,6.0,1

17/11/2009,16:56:41,24.15,5.85,5.6,1

17/11/2009,16:56:40,24.22,5.78,5.8,1

17/11/2009,16:56:39,24.4,5.6,5.9,1

17/11/2009,17:35:06,24.61,5.39,5.4,1

17/11/2009,17:35:07,24.61,5.39,5.4,1

17/11/2009,17:35:11,24.82,5.18,5.2,1

17/11/2009,17:35:19,25.34,4.66,4.7,1

17/11/2009,17:35:29,26.64,3.36,3.4,1

17/11/2009,17:35:32,26.53,3.47,3.5,1

17/11/2009,17:35:34,26.39,3.61,3.6,1

17/11/2009,18:37:00,23.63,6.37,6.2,2

17/11/2009,18:37:00,23.84,6.16,6.4,1

17/11/2009,18:37:05,23.42,6.58,6.6,1

17/11/2009,18:37:08,23.42,6.58,6.6,1

17/11/2009,18:41:56,24.05,5.95,6.0,1

17/11/2009,21:21:31,22.3,7.7,7.7,1

17/11/2009,21:21:33,21.91,8.09,8.1,1

17/11/2009,22:16:32,21.35,8.65,8.7,1

17/11/2009,23:45:32,14.6,15.4,15.4,1

18/11/2009,01:23:00,21.67,8.33,8.3,1

18/11/2009,01:25:31,23.56,6.44,6.2,1

18/11/2009,01:25:30,23.77,6.23,6.4,2

18/11/2009,01:26:02,24.99,5.01,5.0,1

18/11/2009,02:22:43,18.94,11.06,11.1,1

18/11/2009,02:24:06,19.71,10.29,10.3,1

18/11/2009,02:39:59,18.48,11.52,11.5,1

18/11/2009,02:52:34,19.74,10.26,10.3,1

18/11/2009,03:03:32,18.62,11.38,11.4,1

18/11/2009,03:04:23,20.86,9.14,9.1,1

18/11/2009,03:04:26,21,9,9.0,1

18/11/2009,03:04:52,22.93,7.07,7.1,1

18/11/2009,03:04:55,22.86,7.14,7.1,1

18/11/2009,03:05:11,23.14,6.86,6.9,1

18/11/2009,03:07:40,20.86,9.14,9.1,2

18/11/2009,03:07:37,20.86,9.14,9.1,1

18/11/2009,03:16:11,22.82,7.18,7.2,1

18/11/2009,03:16:14,22.89,7.11,7.1,1

18/11/2009,03:21:28,17.36,12.64,12.6,1

18/11/2009,03:37:53,18.24,11.76,11.8,1

18/11/2009,03:44:39,18.45,11.55,11.6,1

18/11/2009,03:49:58,14.91,15.09,15.1,1

18/11/2009,03:54:18,17.33,12.67,12.7,1

18/11/2009,03:57:08,18.2,11.8,5.4,1

18/11/2009,03:56:16,24.61,5.39,11.8,1

18/11/2009,04:08:26,23.38,6.62,6.6,1

18/11/2009,04:08:30,23.52,6.48,6.5,1

18/11/2009,04:08:33,23.63,6.37,6.4,1

18/11/2009,04:23:40,18.97,11.03,11.0,1

18/11/2009,04:23:42,18.69,11.31,11.3,1

18/11/2009,04:24:20,19.81,10.19,10.2,1

18/11/2009,04:24:23,19.88,10.12,10.1,1

18/11/2009,04:24:51,17.4,12.6,12.6,1

18/11/2009,04:28:32,21.88,8.12,8.1,1

18/11/2009,04:28:42,22.12,7.88,7.9,1

18/11/2009,04:28:45,22.16,7.84,7.8,1

18/11/2009,04:36:39,23.42,6.58,20.7,1

18/11/2009,04:36:41,23.63,6.37,6.6,1

18/11/2009,04:38:09,17.4,12.6,6.4,1

18/11/2009,04:38:11,17.33,12.67,12.6,1

18/11/2009,04:35:45,9.27,20.73,12.7,1

18/11/2009,04:41:02,19.39,10.61,10.6,1

18/11/2009,04:41:01,19.39,10.61,10.6,1

18/11/2009,04:48:45,21.91,8.09,8.1,1

18/11/2009,04:49:29,23.87,6.13,6.1,1

18/11/2009,04:49:38,23.98,6.02,6.0,1

18/11/2009,04:56:16,21,9,9.0,1

18/11/2009,04:56:33,20.76,9.24,9.2,1

18/11/2009,04:56:34,20.55,9.45,9.5,1

18/11/2009,04:56:40,20.72,9.28,9.3,2

18/11/2009,04:56:42,20.34,9.66,9.7,1

18/11/2009,04:57:49,16.35,13.65,13.7,1

18/11/2009,04:57:51,16.45,13.55,13.6,1

18/11/2009,04:57:52,16.31,13.69,14.5,1

18/11/2009,04:57:51,15.47,14.53,13.7,1

18/11/2009,04:58:00,9.03,20.97,21.0,1

18/11/2009,04:58:01,8.75,21.25,21.3,1

18/11/2009,05:00:14,20.2,9.8,9.8,1

18/11/2009,05:00:16,19.95,10.05,10.1,2

18/11/2009,05:09:28,21.63,8.37,8.4,1

18/11/2009,05:13:47,16.28,13.72,13.7,1

18/11/2009,05:13:47,16.07,13.93,13.9,1

18/11/2009,05:13:48,16.1,13.9,13.9,1

18/11/2009,05:13:51,16.1,13.9,13.9,1

18/11/2009,05:19:05,11.55,18.45,18.5,1

18/11/2009,05:23:51,20.93,9.07,9.1,1

18/11/2009,05:23:51,20.62,9.38,9.4,1

18/11/2009,05:24:18,21,9,9.0,1

18/11/2009,05:28:40,23.07,6.93,6.9,1

18/11/2009,05:30:34,20.72,9.28,9.3,1

18/11/2009,05:30:44,22.23,7.77,7.8,1

18/11/2009,05:30:48,22.54,7.46,7.5,1

18/11/2009,05:30:49,22.4,7.6,7.6,1

18/11/2009,05:30:50,22.05,7.95,8.0,1

18/11/2009,05:30:57,21.74,8.26,8.3,1

18/11/2009,05:31:05,21.98,8.02,8.0,1

18/11/2009,05:31:08,21.77,8.23,8.2,1

18/11/2009,05:31:24,22.3,7.7,7.5,1

18/11/2009,05:31:24,22.54,7.46,7.7,1

18/11/2009,05:32:03,24.08,5.92,5.9,1

18/11/2009,05:32:15,24.82,5.18,5.2,1

18/11/2009,05:32:17,24.78,5.22,5.2,1

18/11/2009,05:33:34,23.66,6.34,6.3,1

18/11/2009,05:33:37,24.01,5.99,6.0,1

18/11/2009,05:33:40,24.19,5.81,5.8,1

18/11/2009,05:33:41,24.15,5.85,5.9,1

18/11/2009,05:34:55,24.01,5.99,6.0,1

18/11/2009,05:35:03,24.15,5.85,5.9,1

18/11/2009,05:35:17,20.44,9.56,9.6,1

18/11/2009,05:35:24,20.86,9.14,9.1,1

18/11/2009,05:38:35,25.03,4.97,5.0,1

18/11/2009,05:39:19,19.6,10.4,10.4,1

18/11/2009,05:39:38,19.6,10.4,10.4,1

18/11/2009,05:39:37,19.64,10.36,10.4,1

18/11/2009,05:39:35,19.64,10.36,10.4,1

18/11/2009,05:40:29,23.98,6.02,6.0,1

18/11/2009,05:41:18,10.78,19.22,18.9,1

18/11/2009,05:41:18,11.06,18.94,19.2,1

18/11/2009,05:44:24,22.37,7.63,7.6,1

18/11/2009,05:44:44,22.02,7.98,8.0,1

18/11/2009,05:44:50,20.93,9.07,9.1,1

18/11/2009,05:46:47,23.77,6.23,6.4,1

18/11/2009,05:46:45,23.63,6.37,6.2,1

18/11/2009,05:46:54,25.17,4.83,4.8,1

18/11/2009,05:48:39,22.16,7.84,7.8,1

18/11/2009,05:48:41,21.95,8.05,8.1,1

18/11/2009,05:48:41,21.74,8.26,8.3,1

18/11/2009,05:49:06,18.55,11.45,11.5,1

18/11/2009,05:49:23,16.38,13.62,11.4,1

18/11/2009,05:49:24,16.14,13.86,13.6,1

18/11/2009,05:49:07,18.59,11.41,13.9,2

18/11/2009,05:50:50,17.05,12.95,13.0,1

18/11/2009,05:50:55,16.94,13.06,13.1,1

18/11/2009,05:51:01,16.63,13.37,13.4,1

18/11/2009,05:51:09,17.15,12.85,12.9,1

18/11/2009,05:51:11,16.87,13.13,13.1,2

18/11/2009,05:51:42,24.26,5.74,5.7,1

18/11/2009,05:51:44,24.26,5.74,5.7,1

18/11/2009,05:51:45,24.26,5.74,5.7,1

18/11/2009,05:51:48,24.15,5.85,13.4,1

18/11/2009,05:51:49,21.21,8.79,5.9,1

18/11/2009,05:51:45,16.56,13.44,8.8,1

18/11/2009,05:52:35,22.61,7.39,8.0,2

18/11/2009,05:52:31,22.05,7.95,7.4,1

18/11/2009,05:53:17,22.86,7.14,7.1,1

18/11/2009,05:53:18,19.43,10.57,10.6,1

18/11/2009,05:53:22,19.85,10.15,10.2,1

18/11/2009,05:53:24,20.37,9.63,9.6,1

18/11/2009,05:53:56,18.34,11.66,11.7,1

18/11/2009,05:53:58,18.48,11.52,11.5,1

18/11/2009,05:53:58,18.13,11.87,11.9,1

18/11/2009,05:54:06,17.96,12.04,12.0,1

18/11/2009,05:54:08,17.99,12.01,12.0,1

18/11/2009,05:54:17,16.77,13.23,13.2,1

18/11/2009,05:54:29,14.7,15.3,15.3,1

18/11/2009,05:54:32,14.74,15.26,15.3,1

18/11/2009,05:55:21,17.05,12.95,13.0,1

18/11/2009,05:55:28,17.08,12.92,12.9,1

18/11/2009,05:55:32,18.41,11.59,11.6,1

18/11/2009,05:56:00,18.31,11.69,11.7,1

18/11/2009,05:56:05,18.62,11.38,11.4,1

18/11/2009,05:56:07,18.9,11.1,11.1,1

18/11/2009,05:56:45,19.92,10.08,10.1,1

18/11/2009,05:56:51,20.02,9.98,10.0,1

18/11/2009,05:56:54,20.27,9.73,9.7,1

18/11/2009,05:56:54,15.23,14.77,14.8,1

18/11/2009,05:56:57,14.74,15.26,15.3,1

18/11/2009,05:56:59,14.49,15.51,15.5,1

18/11/2009,05:57:06,18.1,11.9,11.9,1

18/11/2009,05:57:06,18.1,11.9,11.9,1

18/11/2009,05:57:14,18.48,11.52,11.5,1

18/11/2009,05:57:34,21.53,8.47,20.8,1

18/11/2009,05:57:40,21.49,8.51,8.5,1

18/11/2009,05:57:23,9.24,20.76,8.5,1

18/11/2009,05:57:55,20.06,9.94,9.9,1

18/11/2009,05:58:00,20.83,9.17,9.2,1

18/11/2009,05:58:10,14.84,15.16,15.2,1

18/11/2009,05:58:13,14.98,15.02,15.0,4

18/11/2009,05:58:25,15.65,14.35,14.4,1

18/11/2009,05:59:05,18.27,11.73,11.7,1

18/11/2009,05:59:14,19.15,10.85,10.9,1

18/11/2009,05:59:36,13.23,16.77,16.8,1

18/11/2009,05:59:40,13.27,16.73,16.7,1

18/11/2009,06:01:56,21.14,8.86,8.9,1

18/11/2009,06:02:04,21.67,8.33,8.3,1

18/11/2009,06:02:07,21.7,8.3,8.3,1

18/11/2009,06:02:07,21.6,8.4,8.4,2

18/11/2009,06:03:11,17.99,12.01,12.0,1

18/11/2009,06:03:19,19.78,10.22,10.2,1

18/11/2009,06:03:27,20.55,9.45,9.5,1

18/11/2009,06:03:28,20.27,9.73,9.7,1

18/11/2009,06:04:02,21.74,8.26,8.3,1

18/11/2009,06:04:05,21.91,8.09,4.7,1

18/11/2009,06:04:07,21.91,8.09,8.1,1

18/11/2009,06:04:03,25.31,4.69,8.1,1

18/11/2009,06:04:09,25.34,4.66,4.7,1

18/11/2009,06:04:12,25.31,4.69,4.7,1

18/11/2009,06:04:32,23.87,6.13,6.1,1

18/11/2009,06:04:32,23.8,6.2,6.2,1

18/11/2009,06:04:33,23.66,6.34,6.3,1

18/11/2009,06:04:36,23.52,6.48,6.5,1

18/11/2009,06:05:14,19.36,10.64,10.6,1

18/11/2009,06:05:22,19.95,10.05,10.1,1

18/11/2009,06:05:50,24.12,5.88,5.9,1

18/11/2009,06:06:01,21.91,8.09,8.1,1

18/11/2009,06:06:06,22.05,7.95,8.0,1

18/11/2009,06:06:10,22.05,7.95,8.0,1

18/11/2009,06:06:21,24.4,5.6,5.6,1

18/11/2009,06:06:52,24.12,5.88,7.3,1

18/11/2009,06:06:51,22.68,7.32,5.9,1

18/11/2009,06:07:16,23.98,6.02,6.0,1

18/11/2009,06:07:33,24.71,5.29,6.2,1

18/11/2009,06:07:16,23.8,6.2,5.3,1

18/11/2009,06:07:34,24.78,5.22,5.2,1

18/11/2009,06:07:58,21.39,8.61,8.6,1

18/11/2009,06:08:15,17.82,12.18,12.2,1

18/11/2009,06:08:17,18.03,11.97,12.0,1

18/11/2009,06:10:50,21.42,8.58,8.6,1

18/11/2009,06:10:54,21.21,8.79,8.8,1

18/11/2009,06:10:59,19.11,10.89,10.9,1

18/11/2009,06:12:37,21.14,8.86,8.9,1

18/11/2009,06:12:39,21.11,8.89,8.9,1

18/11/2009,06:12:45,21.56,8.44,8.4,1

18/11/2009,06:12:46,21.49,8.51,8.5,1

18/11/2009,06:12:57,23.49,6.51,6.5,1

18/11/2009,06:12:59,23.45,6.55,6.6,1

18/11/2009,06:16:34,17.99,12.01,12.0,1

18/11/2009,06:16:40,18.2,11.8,11.8,1

18/11/2009,06:16:42,18.24,11.76,11.8,1

18/11/2009,06:17:24,19.74,10.26,10.3,1

18/11/2009,06:17:29,20.44,9.56,9.6,1

18/11/2009,06:17:32,20.48,9.52,9.5,1

18/11/2009,06:17:35,20.58,9.42,9.4,1

18/11/2009,06:17:38,20.86,9.14,9.1,1

18/11/2009,06:18:00,21.81,8.19,8.2,1

18/11/2009,06:18:08,22.58,7.42,7.4,1

18/11/2009,06:18:10,22.72,7.28,7.3,1

18/11/2009,06:18:11,22.51,7.49,7.5,1

18/11/2009,06:21:39,20.23,9.77,9.8,1

18/11/2009,06:22:06,19.46,10.54,10.5,1

18/11/2009,06:22:08,19.53,10.47,10.5,1

18/11/2009,06:24:57,14.14,15.86,15.9,1

18/11/2009,06:26:13,20.48,9.52,9.5,1

18/11/2009,06:26:17,20.06,9.94,9.9,1

18/11/2009,06:26:42,22.61,7.39,7.4,1

18/11/2009,06:26:43,22.86,7.14,7.1,1

18/11/2009,06:26:44,22.75,7.25,7.3,1

18/11/2009,06:26:44,22.51,7.49,7.5,1

18/11/2009,06:26:58,21.91,8.09,8.1,1

18/11/2009,06:27:02,21.95,8.05,8.1,1

18/11/2009,06:27:17,19.04,10.96,11.0,1

18/11/2009,06:27:24,22.19,7.81,7.8,1

18/11/2009,06:28:19,24.75,5.25,5.3,2

18/11/2009,06:28:22,23.31,6.69,6.7,1

18/11/2009,06:28:37,22.58,7.42,7.4,1

18/11/2009,06:29:09,19.57,10.43,10.1,1

18/11/2009,06:29:08,19.92,10.08,9.8,1

18/11/2009,06:29:09,20.2,9.8,10.4,1

18/11/2009,06:30:15,22.02,7.98,8.0,1

18/11/2009,06:30:26,22.65,7.35,7.4,1

18/11/2009,06:30:25,22.58,7.42,7.4,1

18/11/2009,06:30:33,19.74,10.26,10.3,1

18/11/2009,06:30:35,19.78,10.22,10.2,1

18/11/2009,06:30:55,22.37,7.63,7.6,1

18/11/2009,06:31:05,19.6,10.4,10.4,1

18/11/2009,06:31:33,23.87,6.13,6.1,1

18/11/2009,06:31:41,21.81,8.19,8.2,1

18/11/2009,06:31:58,24.71,5.29,5.3,1

18/11/2009,06:32:36,22.79,7.21,7.2,1

18/11/2009,06:32:40,22.68,7.32,7.3,1

18/11/2009,06:32:42,22.72,7.28,7.3,1

18/11/2009,06:34:10,16.94,13.06,4.3,1

18/11/2009,06:34:09,16.94,13.06,4.3,1

18/11/2009,06:34:30,17.82,12.18,4.3,1

18/11/2009,06:34:33,17.92,12.08,13.1,1

18/11/2009,06:33:53,25.73,4.27,13.1,1

18/11/2009,06:33:45,25.73,4.27,4.3,1

18/11/2009,06:33:43,25.69,4.31,4.2,1

18/11/2009,06:34:12,25.73,4.27,12.2,1

18/11/2009,06:34:14,25.8,4.2,12.1,1

18/11/2009,06:35:49,22.33,7.67,7.6,1

18/11/2009,06:35:47,22.37,7.63,7.7,1

18/11/2009,06:36:38,24.19,5.81,5.8,1

18/11/2009,06:36:42,24.15,5.85,5.9,1

18/11/2009,06:36:44,24.29,5.71,5.7,1

18/11/2009,06:37:01,24.15,5.85,5.9,1

18/11/2009,06:37:41,21.25,8.75,8.8,1

18/11/2009,06:37:39,21.25,8.75,8.8,1

18/11/2009,06:39:44,24.33,5.67,5.7,1

20/11/2009,16:39:45,22.35,7.65,7.7,1

20/11/2009,16:39:46,22.33,7.67,7.7,1

20/11/2009,16:39:50,22.26,7.74,7.7,1

20/11/2009,16:39:53,22.79,7.21,7.2,1

20/11/2009,16:43:29,18.84,11.16,11.2,1

20/11/2009,16:43:31,18.84,11.16,11.2,1

20/11/2009,16:47:57,22.19,7.81,7.8,1

20/11/2009,16:47:59,22.33,7.67,7.7,1

20/11/2009,16:48:00,22.49,7.51,7.5,1

20/11/2009,16:48:01,22.56,7.44,7.4,1

20/11/2009,21:17:33,17.84,12.16,12.2,1

20/11/2009,21:17:40,17.82,12.18,12.2,1

20/11/2009,21:18:09,19.1,10.9,10.9,1

20/11/2009,21:22:11,18.53,11.47,11.4,1

20/11/2009,21:22:02,18.6,11.4,11.5,1

20/11/2009,21:22:53,17.77,12.23,12.2,1

20/11/2009,21:22:56,17.77,12.23,12.2,1

20/11/2009,21:27:46,19.43,10.57,10.6,1

20/11/2009,21:27:48,19.43,10.57,10.6,1

20/11/2009,21:27:53,19.4,10.6,10.6,1

20/11/2009,22:33:23,24.98,5.02,4.4,1

20/11/2009,22:33:23,25.57,4.43,5.0,1

20/11/2009,22:33:44,25.08,4.92,4.9,1

20/11/2009,22:40:37,16.04,13.96,14.0,1

20/11/2009,22:40:38,15.76,14.24,14.2,1

20/11/2009,22:50:26,24.39,5.61,5.6,1

20/11/2009,22:50:39,24.66,5.34,4.9,1

20/11/2009,22:50:42,24.66,5.34,5.3,1

20/11/2009,22:50:45,24.73,5.27,5.3,1

20/11/2009,22:50:48,24.91,5.09,5.3,1

20/11/2009,22:50:39,25.12,4.88,5.1,1

20/11/2009,22:53:34,22.1,7.9,7.9,1

20/11/2009,22:53:39,22.27,7.73,7.7,1

20/11/2009,22:54:04,22.24,7.76,6.7,1

20/11/2009,22:53:59,22.97,7.03,6.1,1

20/11/2009,22:53:58,23.28,6.72,7.0,1

20/11/2009,22:53:59,23.94,6.06,7.8,1

20/11/2009,23:35:47,23.66,6.34,6.3,1

20/11/2009,23:57:24,7.9,22.1,22.1,1

20/11/2009,23:57:26,8.38,21.62,21.6,1

21/11/2009,00:13:40,22.93,7.07,7.1,1

21/11/2009,00:16:48,16.66,13.34,13.3,1

21/11/2009,00:19:07,24.6,5.4,5.4,1

21/11/2009,00:19:11,24.56,5.44,5.4,1

21/11/2009,00:27:21,25.36,4.64,4.6,1

21/11/2009,00:27:24,25.67,4.33,4.3,2

21/11/2009,00:27:27,25.84,4.16,4.2,1

21/11/2009,00:28:16,24.7,5.3,5.3,1

21/11/2009,00:34:34,20.75,9.25,8.6,1

21/11/2009,00:34:31,21.37,8.63,9.3,1

21/11/2009,00:37:34,22.07,7.93,7.9,1

21/11/2009,00:37:37,22.31,7.69,7.7,1

21/11/2009,00:37:40,22.52,7.48,7.5,1

21/11/2009,00:49:38,23.31,6.69,6.7,1

21/11/2009,00:49:40,23.31,6.69,6.7,1

21/11/2009,00:50:17,22.52,7.48,7.5,1

21/11/2009,00:50:26,22.86,7.14,7.1,1

21/11/2009,00:53:51,16.66,13.34,14.1,1

21/11/2009,00:53:47,15.93,14.07,13.3,1

21/11/2009,00:56:35,25.98,4.02,4.0,1

21/11/2009,00:56:46,26.19,3.81,3.8,2

21/11/2009,00:56:50,26.47,3.53,3.5,1

21/11/2009,00:56:53,26.5,3.5,3.5,1

21/11/2009,01:04:13,24.49,5.51,5.5,1

21/11/2009,01:04:17,24.66,5.34,5.3,1

21/11/2009,01:10:52,17.32,12.68,12.7,1

21/11/2009,01:10:53,17.56,12.44,12.4,1

21/11/2009,01:11:10,21.06,8.94,9.3,1

21/11/2009,01:11:08,20.68,9.32,8.9,1

21/11/2009,01:11:14,21.44,8.56,8.6,1

21/11/2009,01:21:06,14.31,15.69,15.1,1

21/11/2009,01:21:06,14.55,15.45,15.2,1

21/11/2009,01:21:06,14.76,15.24,15.5,1

21/11/2009,01:21:07,14.96,15.04,15.7,1

21/11/2009,01:21:06,14.93,15.07,15.0,1

21/11/2009,01:21:38,19.12,10.88,11.4,2

21/11/2009,01:21:22,18.57,11.43,10.9,1

21/11/2009,01:22:27,22.17,7.83,7.8,1

21/11/2009,01:24:31,18.91,11.09,11.1,1

21/11/2009,01:34:33,24.91,5.09,14.7,1

21/11/2009,01:34:26,14.79,15.21,15.2,1

21/11/2009,01:34:24,15.31,14.69,15.1,4

21/11/2009,01:34:33,15.03,14.97,5.1,2

21/11/2009,01:34:39,16,14,15.0,1

21/11/2009,01:34:40,16,14,14.0,1

21/11/2009,01:34:41,15.8,14.2,14.0,1

21/11/2009,01:34:42,15.59,14.41,14.2,1

21/11/2009,01:34:43,15.31,14.69,14.4,1

21/11/2009,01:34:32,14.89,15.11,14.7,1

21/11/2009,01:38:35,19.02,10.98,11.0,1

21/11/2009,01:47:25,22.97,7.03,7.0,1

21/11/2009,01:47:30,22.93,7.07,7.1,1

21/11/2009,01:51:33,21.3,8.7,8.1,1

21/11/2009,01:51:31,21.03,8.97,9.0,1

21/11/2009,01:51:23,21.86,8.14,8.7,1

21/11/2009,02:10:31,21.82,8.18,8.3,1

21/11/2009,02:10:28,21.69,8.31,8.2,1

21/11/2009,02:10:44,22.24,7.76,7.8,1

21/11/2009,02:10:48,22.2,7.8,7.8,1

21/11/2009,02:15:51,20.51,9.49,9.5,2

21/11/2009,02:15:53,20.51,9.49,9.5,2

21/11/2009,02:15:57,20.82,9.18,9.2,1

21/11/2009,02:16:00,20.89,9.11,9.1,1

21/11/2009,02:18:21,15.66,14.34,14.3,1

21/11/2009,02:19:43,18.01,11.99,12.0,1

21/11/2009,02:19:47,18.15,11.85,11.9,1

21/11/2009,02:19:49,18.29,11.71,11.7,1

21/11/2009,02:22:27,18.08,11.92,11.8,1

21/11/2009,02:22:26,18.22,11.78,11.9,1

21/11/2009,02:23:24,16.07,13.93,13.9,1

21/11/2009,02:25:58,15.28,14.72,14.7,1

21/11/2009,02:29:25,18.78,11.22,11.2,2

21/11/2009,02:36:05,22.9,7.1,7.1,1

21/11/2009,02:38:18,22.59,7.41,7.4,1

21/11/2009,02:38:21,22.52,7.48,7.5,1

21/11/2009,02:39:01,23.73,6.27,6.3,1

21/11/2009,02:39:05,23.8,6.2,6.2,1

21/11/2009,02:42:25,23.42,6.58,6.8,1

21/11/2009,02:42:21,23.24,6.76,6.6,1

21/11/2009,02:44:35,11.19,18.81,18.8,1

21/11/2009,02:49:18,22.55,7.45,7.5,1

21/11/2009,02:58:05,27.16,2.84,2.8,1

21/11/2009,02:58:07,27.16,2.84,2.8,1

21/11/2009,02:58:10,27.19,2.81,2.8,1

21/11/2009,03:02:14,7.24,22.76,7.6,1

21/11/2009,02:59:31,24.7,5.3,5.3,1

21/11/2009,02:59:20,22.45,7.55,22.8,1

21/11/2009,03:03:34,15.52,14.48,14.3,1

21/11/2009,03:03:33,15.73,14.27,14.5,1

21/11/2009,03:04:12,14.79,15.21,15.2,1

21/11/2009,03:08:58,13.51,16.49,16.5,1

21/11/2009,03:09:02,13.68,16.32,16.3,1

21/11/2009,03:09:55,16.28,13.72,13.7,1

21/11/2009,03:09:58,16.35,13.65,13.7,1

21/11/2009,03:10:03,17.04,12.96,13.0,3

21/11/2009,03:10:08,18.05,11.95,12.0,1

21/11/2009,03:10:10,18.05,11.95,12.0,1

21/11/2009,03:10:17,19.43,10.57,10.1,1

21/11/2009,03:10:18,19.33,10.67,10.6,1

21/11/2009,03:10:19,19.19,10.81,10.7,2

21/11/2009,03:10:14,19.92,10.08,10.8,1

21/11/2009,03:10:29,18.15,11.85,11.9,1

21/11/2009,03:10:32,18.32,11.68,11.7,1

21/11/2009,03:10:36,18.74,11.26,11.3,1

21/11/2009,03:10:38,19.23,10.77,10.8,1

21/11/2009,03:10:39,19.23,10.77,10.8,1

21/11/2009,03:10:41,19.23,10.77,10.8,1

21/11/2009,03:20:21,18.12,11.88,11.8,1

21/11/2009,03:20:20,18.19,11.81,11.9,1

21/11/2009,03:20:37,19.02,10.98,9.8,1

21/11/2009,03:20:30,20.2,9.8,9.9,2

21/11/2009,03:20:30,20.09,9.91,6.9,1

21/11/2009,03:20:40,23.63,6.37,11.0,1

21/11/2009,03:20:39,23.8,6.2,6.2,2

21/11/2009,03:20:34,23.07,6.93,6.4,1

21/11/2009,03:23:51,12.57,17.43,17.4,1

21/11/2009,03:27:34,16.56,13.44,13.5,1

21/11/2009,03:27:32,16.52,13.48,13.4,1

21/11/2009,03:33:12,20.09,9.91,9.9,2

21/11/2009,03:33:39,10.98,19.02,19.0,1

21/11/2009,03:33:47,11.22,18.78,18.8,2

21/11/2009,03:33:48,11.36,18.64,18.6,1

21/11/2009,03:35:55,19.57,10.43,10.4,1

21/11/2009,03:38:41,19.23,10.77,10.1,1

21/11/2009,03:38:43,19.29,10.71,10.8,1

21/11/2009,03:38:24,19.95,10.05,10.7,1

21/11/2009,03:40:11,16.63,13.37,13.4,1

21/11/2009,03:40:25,16.73,13.27,13.3,1

21/11/2009,03:43:47,10.7,19.3,19.3,1

21/11/2009,03:48:45,14.13,15.87,15.8,1

21/11/2009,03:48:43,14.17,15.83,15.9,1

21/11/2009,03:55:21,23.97,6.03,6.0,2

21/11/2009,03:55:32,24.14,5.86,5.9,1

21/11/2009,03:56:11,17.53,12.47,12.5,1

21/11/2009,03:56:14,17.6,12.4,12.4,1

21/11/2009,03:58:22,25.84,4.16,4.2,1

21/11/2009,04:01:55,25.32,4.68,4.4,1

21/11/2009,04:01:54,25.57,4.43,4.7,2

21/11/2009,04:10:19,21.48,8.52,5.9,1

21/11/2009,04:09:28,24.08,5.92,6.2,1

21/11/2009,04:09:30,23.83,6.17,8.5,2

21/11/2009,04:18:20,23.69,6.31,11.6,1

21/11/2009,04:17:06,17.56,12.44,12.4,1

21/11/2009,04:17:04,18.43,11.57,6.3,1

21/11/2009,04:22:21,24.56,5.44,5.4,1

21/11/2009,04:24:55,17.15,12.85,13.0,1

21/11/2009,04:24:51,17.01,12.99,12.9,1

21/11/2009,04:27:22,23.07,6.93,4.9,1

21/11/2009,04:28:03,22.03,7.97,6.9,1

21/11/2009,04:25:43,25.15,4.85,8.0,1

21/11/2009,04:29:12,18.32,11.68,11.7,1

21/11/2009,04:29:06,18.26,11.74,11.7,2

21/11/2009,04:35:48,22.41,7.59,7.6,1

21/11/2009,04:36:58,21.03,8.97,9.0,1

21/11/2009,04:38:07,18.22,11.78,11.8,1

21/11/2009,04:41:52,13.37,16.63,11.9,1

21/11/2009,04:41:49,12.95,17.05,12.2,1

21/11/2009,04:41:48,13.44,16.56,12.0,1

21/11/2009,04:41:05,19.02,10.98,11.7,1

21/11/2009,04:41:03,18.84,11.16,11.2,3

21/11/2009,04:40:55,18.26,11.74,11.0,1

21/11/2009,04:40:52,17.98,12.02,16.6,1

21/11/2009,04:40:50,17.84,12.16,17.1,1

21/11/2009,04:40:49,18.12,11.88,16.6,1

21/11/2009,04:46:23,22.69,7.31,10.2,1

21/11/2009,04:45:15,19.78,10.22,10.2,1

21/11/2009,04:45:13,19.85,10.15,10.2,1

21/11/2009,04:45:12,19.81,10.19,7.3,1

21/11/2009,04:47:26,24.01,5.99,6.0,1

21/11/2009,04:52:44,19.29,10.71,5.2,1

21/11/2009,04:52:49,19.36,10.64,5.3,1

21/11/2009,04:52:50,19.33,10.67,10.7,1

21/11/2009,04:52:52,19.33,10.67,10.6,1

21/11/2009,04:53:11,14.2,15.8,10.7,1

21/11/2009,04:52:30,24.7,5.3,10.7,1

21/11/2009,04:52:26,24.77,5.23,15.8,1

21/11/2009,05:00:13,14.2,15.8,15.8,1

21/11/2009,05:01:32,14.34,15.66,15.7,2

21/11/2009,05:04:56,23.94,6.06,6.1,1

21/11/2009,05:05:56,25.7,4.3,4.3,1

21/11/2009,05:09:39,23.83,6.17,8.0,1

21/11/2009,05:09:33,22.03,7.97,6.2,1

21/11/2009,05:10:49,24.84,5.16,5.2,1

21/11/2009,05:10:58,26.12,3.88,3.9,1

21/11/2009,05:16:02,26.74,3.26,9.2,1

21/11/2009,05:15:57,20.85,9.15,15.6,1

21/11/2009,05:15:58,14.41,15.59,3.3,1

21/11/2009,05:21:33,17.91,12.09,12.1,1

21/11/2009,05:24:59,19.88,10.12,17.2,1

21/11/2009,05:25:03,20.54,9.46,10.2,1

21/11/2009,05:24:57,19.85,10.15,10.1,1

21/11/2009,05:25:10,18.57,11.43,9.5,1

21/11/2009,05:25:10,18.57,11.43,11.4,1

21/11/2009,05:24:53,12.78,17.22,11.4,1

21/11/2009,05:26:01,20.99,9.01,9.0,1

21/11/2009,05:26:54,19.81,10.19,10.5,1

21/11/2009,05:26:51,19.54,10.46,10.2,1

21/11/2009,05:28:31,24.91,5.09,16.8,1

21/11/2009,05:28:45,24.32,5.68,5.1,1

21/11/2009,05:28:47,23.94,6.06,14.9,1

21/11/2009,05:28:41,15.1,14.9,5.7,1

21/11/2009,05:28:11,13.2,16.8,6.1,1

21/11/2009,05:29:18,23.04,6.96,5.3,1

21/11/2009,05:29:21,23.04,6.96,7.0,1

21/11/2009,05:29:22,24.73,5.27,7.0,1

21/11/2009,05:29:59,12.26,17.74,5.3,1

21/11/2009,05:29:59,12.54,17.46,5.3,2

21/11/2009,05:29:25,24.7,5.3,5.3,1

21/11/2009,05:29:23,24.73,5.27,17.5,1

21/11/2009,05:29:13,24.73,5.27,17.7,1

21/11/2009,05:30:41,14.48,15.52,15.5,2

21/11/2009,05:35:03,20.96,9.04,11.9,1

21/11/2009,05:35:01,20.96,9.04,9.0,1

21/11/2009,05:34:45,18.12,11.88,9.0,1

21/11/2009,05:36:01,24.11,5.89,4.9,1

21/11/2009,05:36:04,24.11,5.89,4.7,1

21/11/2009,05:37:45,24.01,5.99,5.9,1

21/11/2009,05:37:46,23.73,6.27,5.9,1

21/11/2009,05:35:07,25.29,4.71,6.0,1

21/11/2009,05:35:06,25.12,4.88,6.3,1

21/11/2009,05:45:19,20.2,9.8,5.4,1

21/11/2009,05:45:19,20.02,9.98,5.4,1

21/11/2009,05:45:10,19.99,10.01,8.5,1

21/11/2009,05:44:44,21.48,8.52,10.0,1

21/11/2009,05:43:18,24.56,5.44,9.8,1

21/11/2009,05:43:16,24.56,5.44,10.0,1

21/11/2009,05:45:26,20.16,9.84,9.8,1

21/11/2009,05:49:42,21.86,8.14,6.1,1

21/11/2009,05:50:20,24.08,5.92,4.8,1

21/11/2009,05:50:19,24.08,5.92,8.1,1

21/11/2009,05:50:18,24.08,5.92,5.9,1

21/11/2009,05:50:20,15.62,14.38,5.9,1

21/11/2009,05:46:45,23.94,6.06,5.9,1

21/11/2009,05:47:01,25.22,4.78,5.9,1

21/11/2009,05:50:27,15.97,14.03,5.9,1

21/11/2009,05:50:22,15.86,14.14,5.9,2

21/11/2009,05:50:19,24.14,5.86,14.4,2

21/11/2009,05:50:19,24.14,5.86,14.1,3

21/11/2009,05:50:19,24.14,5.86,14.0,1

21/11/2009,05:52:32,17.67,12.33,4.1,1

21/11/2009,05:52:51,23.42,6.58,7.7,1

21/11/2009,05:52:29,22.27,7.73,12.3,1

21/11/2009,05:51:39,25.91,4.09,6.6,1

21/11/2009,05:53:45,25.46,4.54,4.6,1

21/11/2009,05:53:43,25.39,4.61,4.5,1

21/11/2009,05:54:11,20.44,9.56,9.6,2

21/11/2009,05:54:12,20.44,9.56,9.6,1

21/11/2009,05:54:14,20.54,9.46,9.5,1

21/11/2009,05:54:47,20.2,9.8,9.8,1

21/11/2009,05:54:49,20.2,9.8,9.8,1

21/11/2009,05:59:43,20.26,9.74,9.7,1

21/11/2009,05:59:45,19.99,10.01,10.0,2

21/11/2009,05:59:50,19.99,10.01,10.0,1

21/11/2009,06:03:04,12.43,17.57,17.5,1

21/11/2009,06:03:04,12.5,17.5,17.6,2

21/11/2009,06:03:24,23.83,6.17,17.3,1

21/11/2009,06:05:20,21.44,8.56,17.3,1

21/11/2009,06:05:21,21.55,8.45,6.2,1

21/11/2009,06:05:24,21.37,8.63,22.1,1

21/11/2009,06:05:13,7.9,22.1,8.6,1

21/11/2009,06:03:05,12.68,17.32,8.5,1

21/11/2009,06:03:05,12.71,17.29,8.6,1

21/11/2009,06:05:40,21.2,8.8,8.8,1

21/11/2009,06:05:47,15.86,14.14,14.1,1

21/11/2009,06:06:03,19.92,10.08,10.1,1

21/11/2009,06:06:15,20.02,9.98,10.0,1

21/11/2009,06:06:21,20.82,9.18,9.2,1

21/11/2009,06:06:45,21.34,8.66,6.8,1

21/11/2009,06:06:45,23.17,6.83,8.7,1

21/11/2009,06:07:09,21.3,8.7,8.7,1

21/11/2009,06:07:26,24.32,5.68,5.7,1

21/11/2009,06:07:27,24.32,5.68,5.7,1

21/11/2009,06:07:32,21.82,8.18,8.2,1

21/11/2009,06:07:36,21.72,8.28,8.3,1

21/11/2009,06:08:05,14.27,15.73,15.7,1

21/11/2009,06:10:13,23.94,6.06,6.1,1

21/11/2009,06:10:21,23.59,6.41,6.6,1

21/11/2009,06:10:19,23.45,6.55,9.3,1

21/11/2009,06:10:23,20.85,9.15,6.4,1

21/11/2009,06:10:20,20.75,9.25,9.2,1

21/11/2009,06:11:37,25.15,4.85,4.9,1

21/11/2009,06:11:38,25.01,4.99,5.0,1

21/11/2009,06:11:50,17.8,12.2,4.4,2

21/11/2009,06:11:52,17.8,12.2,12.2,2

21/11/2009,06:12:00,19.92,10.08,12.2,1

21/11/2009,06:11:59,19.99,10.01,10.0,1

21/11/2009,06:12:04,20.26,9.74,10.1,1

21/11/2009,06:12:08,20.33,9.67,9.7,1

21/11/2009,06:12:40,15.97,14.03,9.7,1

21/11/2009,06:12:35,21.2,8.8,8.8,1

21/11/2009,06:11:39,25.57,4.43,14.0,5

21/11/2009,06:14:59,20.33,9.67,4.5,1

21/11/2009,06:15:02,20.51,9.49,4.5,1

21/11/2009,06:13:31,25.32,4.68,4.7,2

21/11/2009,06:13:31,25.53,4.47,9.7,1

21/11/2009,06:13:31,25.53,4.47,9.5,1

21/11/2009,06:16:01,21.3,8.7,8.8,2

21/11/2009,06:15:57,21.17,8.83,11.8,2

21/11/2009,06:16:01,18.26,11.74,8.7,1

21/11/2009,06:15:59,18.19,11.81,11.7,1

21/11/2009,06:17:53,24.49,5.51,5.8,1

21/11/2009,06:17:51,24.49,5.51,5.7,1

21/11/2009,06:17:46,24.35,5.65,5.5,1

21/11/2009,06:18:08,23.31,6.69,5.5,1

21/11/2009,06:18:06,23.28,6.72,9.8,1

21/11/2009,06:17:55,20.23,9.77,6.7,1

21/11/2009,06:18:22,23.66,6.34,6.7,1

21/11/2009,06:18:18,23.69,6.31,6.3,1

21/11/2009,06:18:57,22,8,6.3,1

21/11/2009,06:18:54,22,8,8.7,1

21/11/2009,06:18:44,21.3,8.7,4.5,1

21/11/2009,06:18:54,25.53,4.47,8.0,1

21/11/2009,06:19:00,25.67,4.33,8.0,1

21/11/2009,06:19:10,26.02,3.98,4.3,2

21/11/2009,06:19:34,18.84,11.16,4.0,1

21/11/2009,06:19:32,19.05,10.95,4.0,1

21/11/2009,06:19:27,20.47,9.53,9.5,1

21/11/2009,06:20:02,23.11,6.89,11.0,1

21/11/2009,06:20:02,23.07,6.93,11.2,1

21/11/2009,06:19:57,24.18,5.82,5.8,1

21/11/2009,06:21:03,10.95,19.05,6.9,1

21/11/2009,06:21:02,11.01,18.99,6.9,1

21/11/2009,06:21:15,16.07,13.93,19.0,1

21/11/2009,06:19:10,25.98,4.02,19.1,1

21/11/2009,06:17:40,24.25,5.75,19.1,1

21/11/2009,06:21:03,10.95,19.05,19.1,1

21/11/2009,06:21:04,10.91,19.09,13.9,1

21/11/2009,06:22:38,23.73,6.27,6.3,1

21/11/2009,06:22:44,24.01,5.99,6.0,1

21/11/2009,06:22:47,23.87,6.13,6.1,1

21/11/2009,06:23:52,20.96,9.04,4.4,2

21/11/2009,06:24:32,24.39,5.61,4.1,1

21/11/2009,06:24:31,24.42,5.58,4.0,1

21/11/2009,06:24:30,24.35,5.65,9.0,1

21/11/2009,06:24:31,25.05,4.95,5.7,2

21/11/2009,06:24:37,25.5,4.5,5.0,2

21/11/2009,06:24:47,16.45,13.55,5.6,1

21/11/2009,06:24:50,17.25,12.75,5.6,1

21/11/2009,06:24:52,17.94,12.06,4.5,1

21/11/2009,06:24:52,17.39,12.61,13.6,1

21/11/2009,06:24:51,17.39,12.61,12.8,1

21/11/2009,06:25:22,23.94,6.06,12.6,1

21/11/2009,06:25:17,23.8,6.2,12.1,1

21/11/2009,06:25:15,23.66,6.34,12.6,1

21/11/2009,06:25:10,23.59,6.41,6.4,1

21/11/2009,06:23:47,25.95,4.05,6.3,1

21/11/2009,06:23:48,26.02,3.98,6.2,1

21/11/2009,06:23:46,25.63,4.37,6.2,1

21/11/2009,06:25:21,23.76,6.24,6.2,1

21/11/2009,06:25:17,23.83,6.17,6.1,1

21/11/2009,06:27:15,20.3,9.7,5.2,2

21/11/2009,06:27:18,20.26,9.74,9.7,1

21/11/2009,06:27:19,20.26,9.74,9.7,1

21/11/2009,06:27:45,18.91,11.09,9.7,1

21/11/2009,06:27:46,18.95,11.05,11.1,1

21/11/2009,06:26:26,24.77,5.23,11.1,1

21/11/2009,06:28:39,15.28,14.72,15.1,1

21/11/2009,06:28:37,14.93,15.07,14.7,1

21/11/2009,06:28:46,16.35,13.65,13.7,1

21/11/2009,06:28:49,16.45,13.55,20.4,1

21/11/2009,06:28:46,9.63,20.37,20.1,1

21/11/2009,06:28:48,9.91,20.09,13.6,1

21/11/2009,06:30:54,23.35,6.65,6.7,2

21/11/2009,06:30:55,23.35,6.65,6.7,1

21/11/2009,06:30:57,23.52,6.48,6.5,1

21/11/2009,06:31:00,23.8,6.2,6.2,1

21/11/2009,06:31:14,26.12,3.88,3.9,1

21/11/2009,06:31:28,25.53,4.47,5.3,1

21/11/2009,06:31:31,25.36,4.64,5.4,1

21/11/2009,06:31:18,24.49,5.51,5.5,1

21/11/2009,06:31:18,24.56,5.44,4.5,2

21/11/2009,06:31:18,24.7,5.3,4.6,3

21/11/2009,06:32:10,24.42,5.58,5.4,1

21/11/2009,06:32:09,24.6,5.4,5.6,1

21/11/2009,06:32:22,24.91,5.09,5.1,1

21/11/2009,06:32:23,24.66,5.34,5.3,1

21/11/2009,06:32:26,24.73,5.27,5.3,1

21/11/2009,06:32:28,24.73,5.27,5.3,1

21/11/2009,06:32:30,24.56,5.44,5.4,1

21/11/2009,06:32:48,25.98,4.02,4.0,1

21/11/2009,06:33:16,20.16,9.84,9.8,1

21/11/2009,06:33:47,24.25,5.75,5.8,1

21/11/2009,06:34:23,20.78,9.22,9.2,1

21/11/2009,06:34:45,23.45,6.55,9.7,1

21/11/2009,06:34:43,20.3,9.7,10.0,1

21/11/2009,06:34:44,19.99,10.01,6.6,2

21/11/2009,06:34:48,19.99,10.01,10.0,1

21/11/2009,06:34:49,19.81,10.19,10.2,1

21/11/2009,06:34:49,19.61,10.39,10.4,2

21/11/2009,06:35:06,23.83,6.17,6.2,1

21/11/2009,06:35:00,23.83,6.17,6.2,2

21/11/2009,06:36:05,21.82,8.18,8.3,2

21/11/2009,06:36:04,21.86,8.14,8.1,1

21/11/2009,06:36:03,21.75,8.25,8.2,1

21/11/2009,06:36:41,20.82,9.18,9.2,1

21/11/2009,06:36:44,21.06,8.94,8.9,1

21/11/2009,06:36:46,21.06,8.94,8.9,1

21/11/2009,15:38:51,22.03,7.97,8.0,1

21/11/2009,15:38:57,23.24,6.76,6.8,2

21/11/2009,15:39:08,22.41,7.59,7.6,1

21/11/2009,15:50:09,24.49,5.51,5.5,1

21/11/2009,15:50:11,24.66,5.34,5.3,1

21/11/2009,16:12:07,19.95,10.05,10.1,1

21/11/2009,16:15:31,24.01,5.99,6.0,1

21/11/2009,16:15:34,24.21,5.79,5.8,1

21/11/2009,16:15:38,24.28,5.72,5.7,1

21/11/2009,16:15:46,24.84,5.16,5.2,2

21/11/2009,16:15:49,25.01,4.99,5.0,1

21/11/2009,16:17:25,22.55,7.45,7.5,1

21/11/2009,16:17:24,22.48,7.52,7.5,1

21/11/2009,16:17:28,22.55,7.45,7.5,1

21/11/2009,16:21:52,21.1,8.9,8.9,2

21/11/2009,16:22:05,24.04,5.96,6.0,1

21/11/2009,16:22:22,26.33,3.67,3.7,1

21/11/2009,16:33:40,24.63,5.37,5.4,1

21/11/2009,16:33:41,24.56,5.44,5.4,1

21/11/2009,16:33:46,24.91,5.09,5.1,1

21/11/2009,16:35:23,25.77,4.23,3.9,1

21/11/2009,16:35:23,26.12,3.88,4.2,1

21/11/2009,16:35:46,25.32,4.68,4.7,1

21/11/2009,16:35:52,26.09,3.91,3.9,1

21/11/2009,16:54:17,25.39,4.61,4.6,1

21/11/2009,17:04:07,26.54,3.46,2.8,1

21/11/2009,17:04:07,27.23,2.77,3.5,1

21/11/2009,17:06:02,26.4,3.6,3.6,1

21/11/2009,17:06:00,26.4,3.6,3.6,1

21/11/2009,17:05:59,26.4,3.6,3.6,1

21/11/2009,17:05:58,26.4,3.6,3.6,1

21/11/2009,17:06:09,26.22,3.78,3.8,1

21/11/2009,17:06:15,27.47,2.53,2.5,1

21/11/2009,17:06:58,23.9,6.1,6.0,1

21/11/2009,17:06:56,24.01,5.99,6.1,1

21/11/2009,17:08:53,19.43,10.57,10.6,1

21/11/2009,17:08:55,19.57,10.43,10.4,1

21/11/2009,17:20:27,20.2,9.8,10.0,1

21/11/2009,17:20:21,19.99,10.01,10.0,1

21/11/2009,17:20:20,20.02,9.98,9.8,1

21/11/2009,17:21:13,26.5,3.5,3.5,1

21/11/2009,17:21:19,26.5,3.5,3.6,1

21/11/2009,17:21:21,26.67,3.33,3.5,1

21/11/2009,17:21:05,26.54,3.46,3.5,1

21/11/2009,17:21:07,26.43,3.57,3.3,1

21/11/2009,17:56:05,26.02,3.98,4.0,2

21/11/2009,17:56:09,26.09,3.91,3.9,1

21/11/2009,17:56:13,26.12,3.88,3.9,2

21/11/2009,17:56:24,25.46,4.54,4.5,1

21/11/2009,17:56:25,25.46,4.54,4.5,1

21/11/2009,18:33:21,21.62,8.38,8.4,1

21/11/2009,18:33:26,22.91,7.09,6.0,1

21/11/2009,18:33:28,23.52,6.48,6.1,1

21/11/2009,18:33:28,23.76,6.24,6.1,1

21/11/2009,18:33:28,23.96,6.04,7.1,2

21/11/2009,18:33:26,23.89,6.11,6.0,1

21/11/2009,18:33:25,23.93,6.07,6.2,1

21/11/2009,18:33:25,23.96,6.04,6.5,1

21/11/2009,18:55:05,12.46,17.54,17.5,1

21/11/2009,19:58:25,18.06,11.94,11.9,1

21/11/2009,19:58:27,18.23,11.77,11.8,1

21/11/2009,19:58:31,18.23,11.77,11.8,1

21/11/2009,19:58:34,18.26,11.74,11.7,1

21/11/2009,20:02:50,19.11,10.89,9.5,1

21/11/2009,20:02:48,19.79,10.21,7.8,1

21/11/2009,20:02:46,20.53,9.47,7.5,1

21/11/2009,20:02:47,22.2,7.8,10.2,1

21/11/2009,20:02:48,22.47,7.53,10.9,1

21/11/2009,20:02:56,20.87,9.13,9.1,1

21/11/2009,20:33:20,21.72,8.28,8.3,1

21/11/2009,20:33:22,21.79,8.21,8.2,1

21/11/2009,21:23:31,23.32,6.68,6.7,1

21/11/2009,21:23:46,23.66,6.34,6.3,1

21/11/2009,21:23:46,23.49,6.51,6.5,1

21/11/2009,21:51:39,27.22,2.78,2.8,1

21/11/2009,21:51:43,27.15,2.85,2.9,1

21/11/2009,21:51:46,27.8,2.2,2.2,1

21/11/2009,21:51:46,27.25,2.75,2.8,1

21/11/2009,22:12:29,15.14,14.86,14.9,1

21/11/2009,22:12:34,16.77,13.23,13.2,1

21/11/2009,22:23:13,18.46,11.54,11.5,1

21/11/2009,22:23:25,20.23,9.77,9.8,1

21/11/2009,22:23:29,18.8,11.2,11.2,1

21/11/2009,22:26:55,26.37,3.63,3.6,1

21/11/2009,22:26:54,26.37,3.63,3.6,1

21/11/2009,22:29:47,10.66,19.34,9.3,1

21/11/2009,22:29:01,20.7,9.3,8.2,1

21/11/2009,22:29:08,21.82,8.18,19.3,2

21/11/2009,22:33:25,26.47,3.53,3.4,1

21/11/2009,22:32:53,26.61,3.39,3.5,1

21/11/2009,22:48:22,14.49,15.51,6.8,1

21/11/2009,22:48:24,15.92,14.08,5.9,1

21/11/2009,22:48:37,16.09,13.91,4.8,2

21/11/2009,22:48:39,15.82,14.18,15.5,2

21/11/2009,22:48:42,15.78,14.22,14.1,2

21/11/2009,22:48:00,23.22,6.78,13.9,1

21/11/2009,22:48:09,24.1,5.9,14.2,1

21/11/2009,22:48:13,25.25,4.75,14.2,1

21/11/2009,22:58:29,24.37,5.63,5.6,1

21/11/2009,22:58:31,24.37,5.63,5.6,2

21/11/2009,23:00:50,23.59,6.41,6.4,1

21/11/2009,23:00:52,23.76,6.24,6.2,1

21/11/2009,23:27:17,17.72,12.28,12.3,1

21/11/2009,23:27:19,17.89,12.11,12.1,1

21/11/2009,23:27:50,20.47,9.53,7.6,1

21/11/2009,23:27:50,20.74,9.26,9.3,1

21/11/2009,23:27:56,21.99,8.01,9.5,1

21/11/2009,23:27:56,22.13,7.87,7.9,1

21/11/2009,23:27:57,22.64,7.36,8.0,1

21/11/2009,23:28:08,20.81,9.19,7.4,1

21/11/2009,23:27:43,22.43,7.57,9.2,1

21/11/2009,23:31:37,10.05,19.95,7.6,1

21/11/2009,23:31:37,10.28,19.72,7.6,1

21/11/2009,23:31:37,10.83,19.17,5.4,1

21/11/2009,23:31:44,14.53,15.47,5.6,1

21/11/2009,23:31:26,22.4,7.6,5.6,3

21/11/2009,23:31:23,22.43,7.57,6.1,1

21/11/2009,23:31:35,24.57,5.43,19.2,1

21/11/2009,23:31:36,24.44,5.56,19.7,1

21/11/2009,23:31:37,24.37,5.63,20.0,1

21/11/2009,23:31:37,23.89,6.11,15.5,1

21/11/2009,23:35:15,25.35,4.65,4.7,1

21/11/2009,23:43:05,24.23,5.77,5.6,1

21/11/2009,23:43:05,24.44,5.56,5.8,1

21/11/2009,23:43:09,24.17,5.83,5.8,2

21/11/2009,23:43:13,23.89,6.11,6.1,1

21/11/2009,23:43:33,23.96,6.04,5.9,1

21/11/2009,23:43:33,24.06,5.94,6.0,1

21/11/2009,23:57:49,21.31,8.69,8.4,1

21/11/2009,23:57:49,21.65,8.35,8.7,1

22/11/2009,00:00:35,23.93,6.07,5.9,1

22/11/2009,00:00:33,24.13,5.87,6.1,1

22/11/2009,00:03:50,22.6,7.4,7.5,1

22/11/2009,00:03:47,22.54,7.46,7.4,1

22/11/2009,00:06:51,20.87,9.13,9.5,1

22/11/2009,00:06:18,20.3,9.7,8.4,1

22/11/2009,00:06:14,20.47,9.53,7.1,1

22/11/2009,00:06:15,21.59,8.41,9.7,1

22/11/2009,00:06:17,22.88,7.12,9.1,1

22/11/2009,00:10:24,20.53,9.47,9.5,1

22/11/2009,00:10:27,20.16,9.84,9.8,1

22/11/2009,00:12:03,12.83,17.17,6.4,1

22/11/2009,00:12:00,12.73,17.27,6.6,1

22/11/2009,00:12:01,13.03,16.97,7.6,1

22/11/2009,00:12:04,14.83,15.17,7.8,1

22/11/2009,00:11:33,22.16,7.84,6.6,1

22/11/2009,00:11:31,22.4,7.6,6.8,1

22/11/2009,00:11:36,23.25,6.75,7.9,1

22/11/2009,00:11:36,23.38,6.62,17.3,1

22/11/2009,00:11:41,22.06,7.94,17.0,2

22/11/2009,00:11:16,23.45,6.55,17.2,1

22/11/2009,00:11:14,23.59,6.41,15.2,1

22/11/2009,00:13:08,19.04,10.96,11.0,1

22/11/2009,00:14:46,21.45,8.55,8.6,1

22/11/2009,00:15:31,15.68,14.32,13.7,1

22/11/2009,00:15:30,15.95,14.05,14.1,1

22/11/2009,00:15:29,16.29,13.71,14.3,1

22/11/2009,00:15:40,21.31,8.69,8.9,1

22/11/2009,00:15:38,21.14,8.86,8.7,2

22/11/2009,00:17:07,23.93,6.07,6.1,1

22/11/2009,00:19:53,13.41,16.59,16.6,1

22/11/2009,00:21:38,11.23,18.77,18.8,1

22/11/2009,00:21:51,14.15,15.85,15.9,1

22/11/2009,00:22:03,11.57,18.43,18.4,1

22/11/2009,00:22:03,11.64,18.36,18.4,1

22/11/2009,00:22:15,16.26,13.74,13.7,1

22/11/2009,00:27:22,18.94,11.06,11.1,1

22/11/2009,00:27:27,17.89,12.11,12.1,1

22/11/2009,00:35:00,19.72,10.28,10.0,1

22/11/2009,00:35:00,19.96,10.04,10.3,3

22/11/2009,00:35:03,19.75,10.25,10.3,1

22/11/2009,00:35:03,19.58,10.42,10.4,1

22/11/2009,00:35:11,21.42,8.58,8.2,1

22/11/2009,00:35:09,21.76,8.24,8.6,1

22/11/2009,00:39:57,16.83,13.17,13.4,1

22/11/2009,00:39:55,16.73,13.27,13.3,1

22/11/2009,00:39:52,16.6,13.4,13.2,1

22/11/2009,00:42:48,24.74,5.26,5.8,1

22/11/2009,00:42:50,24.88,5.12,5.3,1

22/11/2009,00:42:17,24.17,5.83,5.1,1

22/11/2009,00:49:17,25.35,4.65,4.7,1

22/11/2009,00:52:08,23.45,6.55,16.0,1

22/11/2009,00:51:32,18.97,11.03,11.0,1

22/11/2009,00:51:15,13.98,16.02,6.6,1

22/11/2009,00:52:15,25.39,4.61,4.6,1

22/11/2009,01:01:25,27.02,2.98,3.0,1

22/11/2009,01:02:30,26.2,3.8,9.1,1

22/11/2009,01:02:27,26.17,3.83,9.2,1

22/11/2009,01:02:18,20.91,9.09,9.1,1

22/11/2009,01:02:15,20.77,9.23,3.8,1

22/11/2009,01:02:15,20.87,9.13,3.8,1

22/11/2009,01:02:35,21.86,8.14,8.1,1

22/11/2009,01:02:45,23.35,6.65,6.7,1

22/11/2009,01:07:24,24.5,5.5,5.3,1

22/11/2009,01:07:23,24.74,5.26,5.5,1

22/11/2009,01:09:39,18.4,11.6,3.1,1

22/11/2009,01:09:40,18.8,11.2,3.0,1

22/11/2009,01:09:40,19.07,10.93,10.2,1

22/11/2009,01:09:35,19.79,10.21,11.6,1

22/11/2009,01:09:52,13.75,16.25,10.9,1

22/11/2009,01:09:53,13.07,16.93,11.2,1

22/11/2009,01:09:57,10.86,19.14,16.3,1

22/11/2009,01:09:10,27.02,2.98,16.9,1

22/11/2009,01:09:09,26.95,3.05,19.1,1

22/11/2009,01:11:27,26.81,3.19,3.2,1

22/11/2009,01:11:24,26.74,3.26,3.3,1

22/11/2009,01:11:22,26.78,3.22,3.2,1

22/11/2009,01:18:17,26.24,3.76,3.8,2

22/11/2009,01:28:23,11.34,18.66,5.6,1

22/11/2009,01:27:47,26.17,3.83,5.7,1

22/11/2009,01:27:45,26.24,3.76,6.5,1

22/11/2009,01:27:40,26.41,3.59,6.9,1

22/11/2009,01:27:38,26.27,3.73,7.2,1

22/11/2009,01:26:49,22.81,7.19,3.7,1

22/11/2009,01:26:49,23.11,6.89,3.6,1

22/11/2009,01:26:49,23.52,6.48,3.8,1

22/11/2009,01:26:45,24.27,5.73,3.8,1

22/11/2009,01:26:45,24.44,5.56,18.7,1

22/11/2009,01:31:34,20.84,9.16,9.1,1

22/11/2009,01:31:30,20.6,9.4,9.4,1

22/11/2009,01:31:27,20.87,9.13,9.2,1

22/11/2009,01:33:18,16.02,13.98,14.1,1

22/11/2009,01:33:16,15.92,14.08,14.0,1

22/11/2009,01:34:22,24.71,5.29,5.3,1

22/11/2009,01:34:24,24.81,5.19,5.2,1

22/11/2009,01:34:40,26.58,3.42,3.4,1

22/11/2009,01:39:06,21.38,8.62,7.0,2

22/11/2009,01:39:05,21.62,8.38,7.3,3

22/11/2009,01:39:03,22.74,7.26,8.4,1

22/11/2009,01:39:03,22.98,7.02,8.6,1

22/11/2009,01:43:22,24.3,5.7,5.4,1

22/11/2009,01:43:21,24.47,5.53,5.4,1

22/11/2009,01:43:19,24.54,5.46,5.2,1

22/11/2009,01:43:18,24.47,5.53,5.2,2

22/11/2009,01:43:16,24.37,5.63,5.3,1

22/11/2009,01:43:16,24.47,5.53,5.5,1

22/11/2009,01:43:15,24.67,5.33,5.6,1

22/11/2009,01:43:15,24.81,5.19,5.5,1

22/11/2009,01:43:14,24.78,5.22,5.5,1

22/11/2009,01:43:12,24.61,5.39,5.5,1

22/11/2009,01:43:12,24.57,5.43,5.7,2

22/11/2009,01:44:59,18.36,11.64,3.5,1

22/11/2009,01:44:11,26.54,3.46,11.6,1

22/11/2009,01:45:49,25.01,4.99,6.8,1

22/11/2009,01:45:54,26.98,3.02,7.0,1

22/11/2009,01:45:16,23.05,6.95,5.0,1

22/11/2009,01:45:16,23.22,6.78,3.0,1

22/11/2009,01:49:21,16.9,13.1,13.3,1

22/11/2009,01:49:19,16.97,13.03,13.0,1

22/11/2009,01:49:17,16.73,13.27,13.1,1

22/11/2009,01:50:03,15.51,14.49,14.5,1

22/11/2009,01:50:07,16.5,13.5,13.5,1

22/11/2009,01:57:07,23.38,6.62,6.4,1

22/11/2009,01:57:05,23.52,6.48,6.5,1

22/11/2009,01:56:54,23.49,6.51,6.5,1

22/11/2009,01:56:54,23.62,6.38,6.6,1

22/11/2009,01:58:10,21.62,8.38,8.4,1

22/11/2009,01:58:19,24.23,5.77,5.8,1

22/11/2009,02:04:04,23.89,6.11,6.3,1

22/11/2009,02:03:57,23.76,6.24,6.3,1

22/11/2009,02:03:53,23.76,6.24,4.1,1

22/11/2009,02:03:49,23.76,6.24,6.2,1

22/11/2009,02:03:44,23.69,6.31,6.2,1

22/11/2009,02:03:44,23.72,6.28,6.2,1

22/11/2009,02:03:46,25.93,4.07,6.1,1

22/11/2009,02:04:12,25.93,4.07,4.1,1

22/11/2009,02:09:11,27.02,2.98,3.1,1

22/11/2009,02:09:08,26.95,3.05,3.0,2

22/11/2009,02:09:22,27.22,2.78,2.8,1

22/11/2009,02:09:24,27.25,2.75,2.8,1

22/11/2009,02:12:05,17.48,12.52,12.5,2

22/11/2009,02:12:06,17.58,12.42,12.4,1

22/11/2009,02:12:57,23.32,6.68,6.7,1

22/11/2009,02:13:06,26.2,3.8,3.8,1

22/11/2009,02:15:04,26.34,3.66,3.7,1

22/11/2009,02:16:44,24.4,5.6,7.5,1

22/11/2009,02:16:28,22.54,7.46,6.6,2

22/11/2009,02:16:30,23.45,6.55,5.6,2

22/11/2009,02:17:33,23.72,6.28,6.3,4

22/11/2009,02:20:39,26.1,3.9,4.1,1

22/11/2009,02:20:36,25.93,4.07,3.9,3

22/11/2009,02:22:51,22.88,7.12,7.1,1

22/11/2009,02:23:48,23.62,6.38,6.4,1

22/11/2009,02:23:49,23.32,6.68,6.7,1

22/11/2009,02:32:20,9.23,20.77,20.8,1

22/11/2009,02:32:26,9.54,20.46,20.2,1

22/11/2009,02:32:21,9.78,20.22,20.5,2

22/11/2009,02:36:00,21.31,8.69,7.8,1

22/11/2009,02:35:59,21.65,8.35,8.5,1

22/11/2009,02:35:59,21.93,8.07,8.6,2

22/11/2009,02:34:04,22.16,7.84,8.1,1

22/11/2009,02:34:08,21.48,8.52,8.4,3

22/11/2009,02:34:10,21.42,8.58,8.7,1

22/11/2009,02:36:12,22.43,7.57,7.0,1

22/11/2009,02:36:12,22.98,7.02,7.6,1

22/11/2009,02:36:14,21.89,8.11,8.1,1

22/11/2009,02:37:40,22.84,7.16,7.1,1

22/11/2009,02:37:38,22.94,7.06,7.2,1

22/11/2009,02:38:54,24.47,5.53,5.5,1

22/11/2009,02:38:55,24.47,5.53,5.5,2

22/11/2009,02:41:43,23.69,6.31,7.2,2

22/11/2009,02:41:37,23.86,6.14,5.1,1

22/11/2009,02:40:56,22.81,7.19,5.7,2

22/11/2009,02:40:58,24.34,5.66,5.5,2

22/11/2009,02:41:00,24.47,5.53,5.0,1

22/11/2009,02:41:02,25.01,4.99,6.1,1

22/11/2009,02:40:58,24.88,5.12,6.3,2

22/11/2009,02:44:36,23.69,6.31,5.5,1

22/11/2009,02:44:35,24.23,5.77,5.8,1

22/11/2009,02:44:34,24.47,5.53,6.3,1

22/11/2009,02:45:00,24.67,5.33,5.3,1

22/11/2009,02:45:04,24.57,5.43,5.4,1

22/11/2009,02:45:21,25.22,4.78,4.8,1

22/11/2009,02:45:23,25.12,4.88,4.9,1

22/11/2009,02:47:06,25.52,4.48,4.5,1

22/11/2009,02:50:09,8.42,21.58,21.6,1

22/11/2009,02:50:16,8.18,21.82,21.8,1

22/11/2009,02:54:41,26.51,3.49,3.4,1

22/11/2009,02:54:38,26.51,3.49,3.5,1

22/11/2009,02:54:37,26.58,3.42,3.5,1

22/11/2009,02:59:05,25.46,4.54,4.5,1

22/11/2009,02:59:11,25.32,4.68,4.7,1

22/11/2009,02:59:36,26.3,3.7,3.7,1

22/11/2009,03:00:10,26.54,3.46,3.5,1

22/11/2009,03:00:15,26.47,3.53,3.5,1

22/11/2009,03:00:17,26.41,3.59,3.6,1

22/11/2009,03:03:29,25.52,4.48,3.2,1

22/11/2009,03:03:19,26.95,3.05,3.1,2

22/11/2009,03:03:12,26.85,3.15,4.5,2

22/11/2009,03:06:01,19.65,10.35,9.6,2

22/11/2009,03:05:56,20.4,9.6,10.4,1

22/11/2009,03:07:50,26.13,3.87,3.9,1

22/11/2009,03:07:51,25.76,4.24,4.2,1

22/11/2009,03:09:11,26.51,3.49,3.5,1

22/11/2009,03:09:18,26.68,3.32,3.3,1

22/11/2009,03:09:51,26.95,3.05,3.1,1

22/11/2009,03:09:51,26.58,3.42,3.4,1

22/11/2009,03:09:55,25.9,4.1,4.1,1

22/11/2009,03:09:57,25.73,4.27,4.3,1

22/11/2009,03:09:58,25.12,4.88,4.9,1

22/11/2009,03:10:01,25.79,4.21,4.2,1

22/11/2009,03:10:04,25.86,4.14,4.1,1

22/11/2009,03:10:04,25.76,4.24,4.2,1

22/11/2009,03:10:05,25.79,4.21,4.2,1

22/11/2009,03:10:11,26.24,3.76,4.9,1

22/11/2009,03:10:11,26.1,3.9,3.8,1

22/11/2009,03:10:05,25.12,4.88,3.9,1

22/11/2009,03:10:31,18.6,11.4,11.4,2

22/11/2009,03:10:43,19.62,10.38,10.4,1

22/11/2009,03:10:41,19.65,10.35,10.4,1

22/11/2009,03:10:44,19.14,10.86,10.9,1

22/11/2009,03:13:47,21.76,8.24,8.0,1

22/11/2009,03:13:47,21.99,8.01,8.2,1

22/11/2009,03:16:21,22.57,7.43,7.4,1

22/11/2009,03:16:18,22.64,7.36,7.4,1

22/11/2009,03:16:29,21.11,8.89,8.9,1

22/11/2009,03:16:35,22.06,7.94,7.9,1

22/11/2009,03:16:37,21.76,8.24,8.2,1

22/11/2009,03:16:42,21.72,8.28,8.3,1

22/11/2009,03:16:45,21.86,8.14,8.1,1

22/11/2009,03:16:59,23.52,6.48,6.5,1

22/11/2009,03:17:04,23.79,6.21,6.2,1

22/11/2009,03:17:39,25.59,4.41,4.4,2

22/11/2009,03:17:42,25.66,4.34,4.3,1

22/11/2009,03:18:23,25.73,4.27,4.3,1

22/11/2009,03:18:26,25.66,4.34,4.3,1

22/11/2009,03:21:50,25.59,4.41,4.4,1

22/11/2009,03:26:37,22.37,7.63,7.6,1

22/11/2009,03:26:42,22.74,7.26,7.3,1

22/11/2009,03:26:42,22.43,7.57,7.6,1

22/11/2009,03:26:42,21.62,8.38,8.4,2

22/11/2009,03:28:49,21.45,8.55,8.6,1

22/11/2009,03:28:47,21.38,8.62,8.6,1

22/11/2009,03:28:55,21.28,8.72,8.7,1

22/11/2009,03:29:59,19.48,10.52,10.5,1

22/11/2009,03:30:00,19.38,10.62,10.6,1

22/11/2009,03:30:33,19.21,10.79,10.8,1

22/11/2009,03:31:52,23.96,6.04,6.2,1

22/11/2009,03:31:50,24,6,6.0,1

22/11/2009,03:32:30,24.37,5.63,6.0,1

22/11/2009,03:32:32,24.34,5.66,6.2,1

22/11/2009,03:33:47,19.01,10.99,5.6,1

22/11/2009,03:31:52,23.79,6.21,5.7,1

22/11/2009,03:31:49,23.79,6.21,11.0,1

22/11/2009,03:37:38,19.07,10.93,10.9,1

22/11/2009,03:37:35,19.14,10.86,10.9,1

22/11/2009,03:38:22,25.01,4.99,5.0,1

22/11/2009,03:38:27,25.12,4.88,4.9,1

22/11/2009,03:38:29,25.18,4.82,4.8,1

22/11/2009,03:39:20,24.98,5.02,5.1,1

22/11/2009,03:39:18,24.95,5.05,5.0,1

22/11/2009,03:39:22,24.98,5.02,5.0,2

22/11/2009,03:39:23,24.95,5.05,5.1,1

22/11/2009,03:41:51,25.59,4.41,4.4,1

22/11/2009,03:47:18,26.47,3.53,3.5,1

22/11/2009,03:50:39,17.55,12.45,12.5,1

22/11/2009,03:50:45,17.78,12.22,12.2,1

22/11/2009,03:50:59,23.86,6.14,6.1,1

22/11/2009,03:51:02,24.2,5.8,5.8,1

22/11/2009,03:51:04,24.06,5.94,5.9,1

22/11/2009,03:51:42,25.52,4.48,4.5,1

22/11/2009,03:51:44,25.46,4.54,4.5,1

22/11/2009,03:52:23,14.9,15.1,15.1,1

22/11/2009,03:52:33,16.22,13.78,13.8,2

22/11/2009,03:52:34,16.09,13.91,13.9,1

22/11/2009,03:59:50,16.9,13.1,12.9,1

22/11/2009,03:59:50,17.07,12.93,13.1,1

22/11/2009,03:59:57,18.46,11.54,11.5,1

22/11/2009,04:02:30,26.41,3.59,3.0,1

22/11/2009,04:02:29,26.51,3.49,3.5,1

22/11/2009,04:02:36,24.4,5.6,3.6,1

22/11/2009,04:02:40,24.44,5.56,5.6,1

22/11/2009,04:02:50,23.86,6.14,5.6,1

22/11/2009,04:04:00,22.33,7.67,6.1,1

22/11/2009,04:01:41,26.98,3.02,7.7,1

22/11/2009,04:04:48,26.37,3.63,3.6,1

22/11/2009,04:05:39,23.45,6.55,3.7,1

22/11/2009,04:06:06,25.59,4.41,6.6,1

22/11/2009,04:06:16,25.79,4.21,4.4,1

22/11/2009,04:04:48,26.34,3.66,4.2,1

22/11/2009,04:08:51,25.49,4.51,4.5,1

22/11/2009,04:09:43,26.64,3.36,2.9,1

22/11/2009,04:09:41,27.08,2.92,3.4,1

22/11/2009,04:09:54,24.54,5.46,5.5,1

22/11/2009,04:11:27,26.64,3.36,3.4,1

22/11/2009,04:13:56,17.21,12.79,15.6,1

22/11/2009,04:13:55,17.24,12.76,12.8,1

22/11/2009,04:13:56,15.88,14.12,12.8,1

22/11/2009,04:13:57,15.92,14.08,14.1,2

22/11/2009,04:13:58,15.92,14.08,15.1,1

22/11/2009,04:14:00,15.99,14.01,14.1,1

22/11/2009,04:14:02,16.16,13.84,14.1,1

22/11/2009,04:13:56,14.87,15.13,14.0,1

22/11/2009,04:13:54,14.36,15.64,13.8,1

22/11/2009,04:14:17,26.34,3.66,3.7,1

22/11/2009,04:14:22,26.37,3.63,3.6,1

22/11/2009,04:14:23,26.44,3.56,3.6,1

22/11/2009,04:14:28,27.05,2.95,3.0,1

22/11/2009,04:18:38,26.44,3.56,3.6,1

22/11/2009,04:22:36,27.25,2.75,2.8,1

22/11/2009,04:22:38,27.25,2.75,2.8,1

22/11/2009,04:22:31,27.22,2.78,2.8,1

22/11/2009,04:25:06,25.62,4.38,4.4,1

22/11/2009,04:25:08,25.56,4.44,4.4,1

22/11/2009,04:25:31,27.22,2.78,2.8,1

22/11/2009,04:35:52,23.49,6.51,6.5,1

22/11/2009,04:40:00,20.74,9.26,8.9,1

22/11/2009,04:39:27,20.81,9.19,9.2,1

22/11/2009,04:39:25,21.08,8.92,9.3,1

22/11/2009,04:40:35,26.58,3.42,3.3,1

22/11/2009,04:40:32,26.68,3.32,3.4,1

22/11/2009,04:40:47,23.72,6.28,6.3,1

22/11/2009,04:40:54,23.66,6.34,6.3,1

22/11/2009,04:41:03,25.22,4.78,4.8,1

22/11/2009,04:41:06,25.12,4.88,4.9,1

22/11/2009,04:41:36,25.15,4.85,4.9,1

22/11/2009,04:41:38,24.98,5.02,5.0,1

22/11/2009,04:42:14,23.25,6.75,6.8,1

22/11/2009,04:43:43,23.01,6.99,3.7,1

22/11/2009,04:42:39,26.3,3.7,7.0,2

22/11/2009,04:43:57,22.81,7.19,7.2,1

22/11/2009,04:44:01,22.98,7.02,7.0,1

22/11/2009,04:44:45,21.35,8.65,8.7,1

22/11/2009,04:44:42,21.28,8.72,8.7,1

22/11/2009,04:47:10,25.49,4.51,4.5,1

22/11/2009,04:49:10,27.19,2.81,2.9,1

22/11/2009,04:49:07,27.15,2.85,2.8,1

22/11/2009,04:55:40,22.37,7.63,4.7,1

22/11/2009,04:55:18,13.95,16.05,16.1,1

22/11/2009,04:55:20,14.97,15.03,15.0,1

22/11/2009,04:55:51,23.42,6.58,12.2,2

22/11/2009,04:55:18,25.29,4.71,12.4,1

22/11/2009,04:55:32,24.88,5.12,5.1,1

22/11/2009,04:55:44,23.69,6.31,7.6,1

22/11/2009,04:55:31,17.65,12.35,6.3,1

22/11/2009,04:55:30,17.78,12.22,6.6,1

22/11/2009,04:56:32,25.35,4.65,4.7,1

22/11/2009,04:59:21,24.17,5.83,5.8,1

22/11/2009,04:59:28,24.3,5.7,5.7,1

22/11/2009,04:59:30,24.4,5.6,5.6,1

22/11/2009,04:59:46,26.37,3.63,3.6,1

22/11/2009,05:00:03,26.71,3.29,3.1,1

22/11/2009,05:00:01,26.88,3.12,3.3,1

22/11/2009,05:00:06,26.68,3.32,3.3,1

22/11/2009,05:11:49,16.02,13.98,14.0,1

22/11/2009,05:14:34,23.45,6.55,6.6,1

22/11/2009,05:14:40,24.3,5.7,5.7,1

22/11/2009,05:14:42,24.37,5.63,5.6,1

22/11/2009,05:14:42,24.34,5.66,5.7,1

22/11/2009,05:14:45,24.47,5.53,5.5,1

22/11/2009,05:14:45,24.37,5.63,5.6,1

22/11/2009,05:14:45,24.44,5.56,5.6,1

22/11/2009,05:14:46,24.4,5.6,5.6,1

22/11/2009,05:14:47,24.47,5.53,5.5,1

22/11/2009,05:15:35,25.56,4.44,4.4,1

22/11/2009,05:18:55,26.64,3.36,3.4,1

22/11/2009,05:20:05,19.18,10.82,10.8,1

22/11/2009,05:20:30,23.52,6.48,6.2,2

22/11/2009,05:20:30,23.79,6.21,6.5,2

22/11/2009,05:22:52,24.61,5.39,5.4,2

22/11/2009,05:22:55,26.61,3.39,3.4,1

22/11/2009,05:23:09,24.98,5.02,5.0,1

22/11/2009,05:28:16,23.79,6.21,6.2,1

22/11/2009,05:28:23,24.84,5.16,5.2,2

22/11/2009,05:28:25,24.98,5.02,5.0,1

22/11/2009,05:33:40,23.59,6.41,6.5,1

22/11/2009,05:33:35,23.42,6.58,6.6,2

22/11/2009,05:33:35,23.55,6.45,6.4,1

22/11/2009,05:38:16,22.67,7.33,7.3,2

22/11/2009,05:38:19,23.05,6.95,7.0,1

22/11/2009,05:41:09,12.76,17.24,17.2,1

22/11/2009,05:46:20,19.92,10.08,10.1,1

22/11/2009,05:46:22,19.92,10.08,10.1,1

22/11/2009,05:48:09,24.74,5.26,5.3,1

22/11/2009,05:51:29,25.52,4.48,3.1,1

22/11/2009,05:51:03,26.88,3.12,4.5,1

22/11/2009,05:51:33,25.59,4.41,4.4,1

22/11/2009,05:52:40,24.98,5.02,6.1,1

22/11/2009,05:52:42,24.95,5.05,5.0,1

22/11/2009,05:51:50,23.93,6.07,5.1,1

22/11/2009,05:54:45,26.37,3.63,3.6,1

22/11/2009,05:58:39,25.86,4.14,4.1,1

22/11/2009,06:03:23,25.49,4.51,4.5,1

22/11/2009,06:06:19,26.41,3.59,3.6,1

22/11/2009,06:11:19,25.86,4.14,4.1,1

22/11/2009,06:16:24,26.27,3.73,3.7,1

22/11/2009,06:19:25,24.88,5.12,5.1,1

22/11/2009,06:25:53,25.18,4.82,4.8,1

22/11/2009,06:25:46,25.12,4.88,4.9,1

22/11/2009,06:25:45,25.18,4.82,4.8,1

22/11/2009,06:29:54,24.5,5.5,5.5,1

22/11/2009,06:29:57,24.5,5.5,5.5,1

22/11/2009,06:30:10,25.93,4.07,4.1,1

22/11/2009,06:30:14,26.2,3.8,3.8,1

22/11/2009,06:30:15,26.41,3.59,4.0,1

22/11/2009,06:30:14,26,4,3.6,1

22/11/2009,14:58:35,24.2,5.8,5.8,1

22/11/2009,14:58:39,24.2,5.8,5.8,2

22/11/2009,14:58:39,23.59,6.41,6.4,1

22/11/2009,14:58:43,24.34,5.66,5.7,1

22/11/2009,14:58:48,22.74,7.26,7.3,1

22/11/2009,14:58:55,22.98,7.02,7.0,1

22/11/2009,14:58:57,23.08,6.92,6.9,1

22/11/2009,14:59:00,23.32,6.68,6.7,1

22/11/2009,14:59:01,23.05,6.95,7.0,1

22/11/2009,14:59:01,22.91,7.09,7.1,1

22/11/2009,14:59:02,22.84,7.16,7.2,1

22/11/2009,14:59:03,22.74,7.26,7.7,1

22/11/2009,14:59:03,22.64,7.36,7.3,1

22/11/2009,14:59:02,22.26,7.74,7.4,1

22/11/2009,14:59:03,22.1,7.9,7.9,1

22/11/2009,14:59:04,21.89,8.11,8.6,1

22/11/2009,14:59:03,21.45,8.55,8.1,1

22/11/2009,14:59:07,24.17,5.83,5.6,1

22/11/2009,14:59:07,24.27,5.73,5.7,1

22/11/2009,14:59:07,24.4,5.6,5.8,1

22/11/2009,14:59:10,24.84,5.16,4.7,1

22/11/2009,14:59:09,25.35,4.65,5.2,1

22/11/2009,15:07:05,24.2,5.8,6.7,1

22/11/2009,15:07:09,24,6,5.8,1

22/11/2009,15:07:12,24.03,5.97,6.0,1

22/11/2009,15:07:14,24.2,5.8,6.0,1

22/11/2009,15:07:16,24.3,5.7,6.0,1

22/11/2009,15:07:18,24.3,5.7,5.8,1

22/11/2009,15:07:18,24.03,5.97,5.7,1

22/11/2009,15:07:26,24.71,5.29,5.7,1

22/11/2009,15:07:29,25.39,4.61,6.0,1

22/11/2009,15:07:29,24.88,5.12,5.3,1

22/11/2009,15:07:29,24.17,5.83,4.6,1

22/11/2009,15:07:30,23.96,6.04,5.1,1

22/11/2009,15:07:33,23.83,6.17,5.8,1

22/11/2009,15:07:36,23.76,6.24,6.0,1

22/11/2009,15:07:39,23.69,6.31,6.2,1

22/11/2009,15:07:42,23.22,6.78,6.2,2

22/11/2009,15:07:41,23.22,6.78,6.3,1

22/11/2009,15:07:46,23.28,6.72,6.8,1

22/11/2009,15:07:45,23.11,6.89,6.8,1

22/11/2009,15:07:48,23.08,6.92,6.9,1

22/11/2009,15:07:47,22.98,7.02,4.4,1

22/11/2009,15:07:50,22.94,7.06,6.7,1

22/11/2009,15:07:50,22.77,7.23,4.5,1

22/11/2009,15:07:52,22.71,7.29,7.0,1

22/11/2009,15:07:52,22.43,7.57,6.9,1

22/11/2009,15:07:58,23.42,6.58,7.1,1

22/11/2009,15:08:08,25.12,4.88,7.2,1

22/11/2009,15:07:55,25.35,4.65,7.3,1

22/11/2009,15:07:47,25.52,4.48,7.6,1

22/11/2009,15:07:46,25.62,4.38,4.7,2

22/11/2009,15:07:12,23.96,6.04,6.6,1

22/11/2009,15:07:01,23.32,6.68,4.9,1

22/11/2009,15:08:08,22.71,7.29,7.3,1

22/11/2009,15:08:12,22.23,7.77,7.8,1

22/11/2009,15:13:13,25.49,4.51,4.1,1

22/11/2009,15:13:12,25.66,4.34,4.1,1

22/11/2009,15:13:11,25.86,4.14,4.1,1

22/11/2009,15:13:09,25.86,4.14,4.3,1

22/11/2009,15:13:27,25.66,4.34,4.5,1

22/11/2009,15:13:30,25.59,4.41,4.3,1

22/11/2009,15:13:06,25.9,4.1,4.4,1

22/11/2009,15:14:12,25.56,4.44,4.3,1

22/11/2009,15:14:11,25.69,4.31,4.4,1

22/11/2009,15:14:14,25.46,4.54,4.5,1

22/11/2009,15:14:15,25.46,4.54,4.5,1

22/11/2009,15:14:16,25.79,4.21,4.2,1

22/11/2009,15:14:23,25.76,4.24,4.2,1

22/11/2009,15:15:16,25.62,4.38,4.4,1

22/11/2009,15:15:18,25.49,4.51,4.5,1

22/11/2009,15:15:19,25.39,4.61,4.6,1

22/11/2009,15:16:18,25.29,4.71,4.7,1

22/11/2009,15:16:21,25.32,4.68,4.7,1

22/11/2009,15:16:24,25.32,4.68,4.7,1

22/11/2009,15:16:36,25.15,4.85,4.6,1

22/11/2009,15:16:36,25.39,4.61,4.9,2

22/11/2009,15:16:40,24.95,5.05,5.1,2

22/11/2009,15:16:45,25.15,4.85,5.6,2

22/11/2009,15:16:47,25.05,4.95,4.9,1

22/11/2009,15:16:40,24.4,5.6,5.9,1

22/11/2009,15:16:45,24.1,5.9,5.0,2

22/11/2009,15:27:51,26.37,3.63,3.6,2

22/11/2009,15:27:54,26.27,3.73,3.7,1

22/11/2009,15:27:59,26.41,3.59,3.6,1

22/11/2009,15:28:02,26.27,3.73,3.7,1

22/11/2009,15:31:20,24.27,5.73,5.7,1

22/11/2009,15:31:18,24.27,5.73,5.7,1

22/11/2009,15:48:02,23.62,6.38,12.4,1

22/11/2009,15:48:03,23.62,6.38,12.7,1

22/11/2009,15:47:49,17.58,12.42,12.6,1

22/11/2009,15:47:55,17.34,12.66,12.8,1

22/11/2009,15:47:58,17.38,12.62,6.4,1

22/11/2009,15:47:59,17.24,12.76,6.4,1

22/11/2009,16:03:06,24.2,5.8,5.3,1

22/11/2009,16:03:05,24.67,5.33,5.8,1

22/11/2009,16:03:26,24.94,5.06,5.1,1

22/11/2009,16:03:29,25.01,4.99,5.0,1

22/11/2009,16:06:39,24.54,5.46,5.3,1

22/11/2009,16:06:39,24.74,5.26,5.5,3

22/11/2009,16:06:45,24.47,5.53,5.5,1

22/11/2009,16:06:48,24.54,5.46,5.5,1

22/11/2009,16:11:31,24.91,5.09,5.1,1

22/11/2009,16:11:32,25.08,4.92,4.9,1

22/11/2009,16:20:11,19.99,10.01,10.0,2

22/11/2009,16:20:10,20.02,9.98,10.0,1

22/11/2009,16:23:15,22.53,7.47,7.5,1

22/11/2009,16:23:17,22.53,7.47,7.5,1

22/11/2009,16:24:11,24.71,5.29,5.3,1

22/11/2009,16:24:09,24.81,5.19,5.2,1

22/11/2009,16:24:08,24.71,5.29,5.3,1

22/11/2009,16:24:21,24.64,5.36,5.4,1

22/11/2009,16:24:22,24.77,5.23,5.2,1

22/11/2009,16:24:24,24.84,5.16,5.2,1

22/11/2009,16:52:12,25.3,4.7,4.6,2

22/11/2009,16:52:11,25.35,4.65,4.7,1

22/11/2009,16:52:11,25.43,4.57,4.7,1

22/11/2009,17:10:28,26.91,3.09,3.1,1

22/11/2009,17:10:35,26.89,3.11,3.1,1

22/11/2009,17:10:38,26.97,3.03,3.0,1

22/11/2009,17:14:04,20.64,9.36,9.6,1

22/11/2009,17:14:02,20.42,9.58,9.4,1

22/11/2009,17:14:11,20.85,9.15,9.3,1

22/11/2009,17:14:10,20.7,9.3,9.2,1

22/11/2009,17:14:11,20.77,9.23,9.2,1

22/11/2009,17:14:13,20.8,9.2,9.2,2

22/11/2009,17:14:14,20.75,9.25,9.3,1

22/11/2009,17:14:15,20.57,9.43,9.4,1

22/11/2009,17:31:55,22.47,7.53,7.2,1

22/11/2009,17:31:51,22.66,7.34,7.3,1

22/11/2009,17:31:50,22.76,7.24,7.5,1

22/11/2009,17:42:26,23.05,6.95,7.0,1

22/11/2009,18:05:33,23.85,6.15,6.4,1

22/11/2009,18:05:31,23.69,6.31,6.3,1

22/11/2009,18:05:30,23.62,6.38,6.2,1

22/11/2009,18:08:33,20.52,9.48,9.5,1

22/11/2009,18:17:37,23.91,6.09,6.1,1

22/11/2009,18:17:39,23.85,6.15,6.2,1

22/11/2009,18:28:18,23.11,6.89,7.1,2

22/11/2009,18:28:16,22.86,7.14,6.9,1

22/11/2009,18:29:13,24.07,5.93,5.9,1

22/11/2009,18:29:16,24.2,5.8,5.8,1

22/11/2009,19:12:45,18.28,11.72,10.2,2

22/11/2009,19:12:41,18.88,11.12,11.1,1

22/11/2009,19:12:40,19.84,10.16,11.7,1

22/11/2009,19:20:49,25.45,4.55,4.4,1

22/11/2009,19:20:48,25.53,4.47,4.5,1

22/11/2009,19:20:48,25.6,4.4,4.6,1

22/11/2009,19:33:42,19.06,10.94,10.9,1

22/11/2009,20:52:51,23.41,6.59,6.4,1

22/11/2009,20:52:44,23.59,6.41,6.6,2

22/11/2009,21:19:36,11.3,18.7,18.7,1

22/11/2009,21:19:35,11.26,18.74,18.7,1

22/11/2009,21:38:56,18.77,11.23,10.9,1

22/11/2009,21:38:50,18.99,11.01,10.3,1

22/11/2009,21:38:49,19.09,10.91,10.9,1

22/11/2009,21:38:51,11.94,18.06,11.0,1

22/11/2009,21:38:54,12.11,17.89,18.1,1

22/11/2009,21:38:54,11.97,18.03,17.9,1

22/11/2009,21:38:08,19.06,10.94,18.0,1

22/11/2009,21:38:09,19.66,10.34,11.2,1

22/11/2009,21:40:37,17.36,12.64,12.9,1

22/11/2009,21:40:30,17.07,12.93,12.6,1

22/11/2009,21:53:03,25.33,4.67,4.4,1

22/11/2009,21:53:01,25.5,4.5,4.5,1

22/11/2009,21:52:59,25.58,4.42,4.7,1

22/11/2009,21:54:39,19.77,10.23,10.3,1

22/11/2009,21:54:38,19.7,10.3,10.2,2

22/11/2009,22:02:04,23.66,6.34,6.4,1

22/11/2009,22:01:58,23.56,6.44,6.3,1

22/11/2009,22:07:36,20.05,9.95,9.7,1

22/11/2009,22:07:34,20.26,9.74,10.0,1

22/11/2009,22:10:46,11.51,18.49,18.5,1

22/11/2009,22:10:50,11.58,18.42,18.4,1

22/11/2009,22:20:31,18.92,11.08,11.1,1

22/11/2009,22:25:49,21.64,8.36,8.4,1

22/11/2009,22:35:32,25.26,4.74,4.7,1

22/11/2009,22:35:54,25.5,4.5,4.5,1

22/11/2009,22:35:55,25.54,4.46,4.5,1

22/11/2009,22:35:57,25.75,4.25,4.3,1

22/11/2009,22:35:59,25.79,4.21,4.2,1

22/11/2009,22:49:42,16.44,13.56,13.6,1

22/11/2009,23:16:26,18.88,11.12,11.1,2

22/11/2009,23:16:24,18.85,11.15,10.7,1

22/11/2009,23:16:52,22.67,7.33,10.4,1

22/11/2009,23:16:04,19.31,10.69,10.5,1

22/11/2009,23:16:05,19.52,10.48,11.2,1

22/11/2009,23:16:05,19.59,10.41,11.1,1

22/11/2009,23:15:58,18.88,11.12,7.3,1

22/11/2009,23:21:04,21.89,8.11,12.9,1

22/11/2009,23:21:06,21.57,8.43,12.1,1

22/11/2009,23:19:11,17.14,12.86,8.1,1

22/11/2009,23:19:17,17.89,12.11,8.4,1

22/11/2009,23:29:55,23.17,6.83,6.9,1

22/11/2009,23:29:53,23.13,6.87,6.9,1

22/11/2009,23:29:52,23.06,6.94,6.8,1

22/11/2009,23:31:01,18.92,11.08,11.0,1

22/11/2009,23:30:59,19.02,10.98,11.1,1

22/11/2009,23:35:36,20.93,9.07,9.1,1

22/11/2009,23:39:08,23.59,6.41,6.4,1

22/11/2009,23:39:09,23.56,6.44,6.4,1

22/11/2009,23:45:32,23.59,6.41,6.4,1

22/11/2009,23:46:11,24.44,5.56,5.6,1

22/11/2009,23:58:30,22.14,7.86,7.7,1

22/11/2009,23:58:28,22.25,7.75,7.8,1

22/11/2009,23:58:27,22.32,7.68,7.9,1

23/11/2009,00:00:23,24.41,5.59,5.6,1

23/11/2009,00:01:07,20.05,9.95,10.0,1

23/11/2009,00:01:09,19.73,10.27,10.3,1

23/11/2009,00:01:10,19.8,10.2,10.2,2

23/11/2009,00:02:09,23.77,6.23,7.8,1

23/11/2009,00:02:11,23.8,6.2,7.5,1

23/11/2009,00:02:12,23.49,6.51,6.2,1

23/11/2009,00:04:04,10.52,19.48,6.2,1

23/11/2009,00:04:03,10.34,19.66,6.5,1

23/11/2009,00:03:20,25.22,4.78,4.8,2

23/11/2009,00:02:07,22.46,7.54,19.6,1

23/11/2009,00:02:04,22.17,7.83,19.7,1

23/11/2009,00:04:06,25.93,4.07,19.5,1

23/11/2009,00:04:05,10.52,19.48,19.5,1

23/11/2009,00:04:03,10.45,19.55,4.1,1

23/11/2009,00:04:07,26.18,3.82,3.8,1

23/11/2009,00:04:43,27.2,2.8,2.8,1

23/11/2009,00:12:37,19.7,10.3,10.3,1

23/11/2009,00:12:43,19.77,10.23,10.2,1

23/11/2009,00:12:46,19.87,10.13,10.1,1

23/11/2009,00:20:20,20.65,9.35,9.4,1

23/11/2009,00:24:23,13.28,16.72,16.8,1

23/11/2009,00:24:23,13.6,16.4,16.4,1

23/11/2009,00:24:20,13.25,16.75,16.7,1

23/11/2009,00:43:25,25.22,4.78,5.0,1

23/11/2009,00:43:22,25.08,4.92,4.9,1

23/11/2009,00:43:20,25.01,4.99,4.8,1

23/11/2009,00:54:11,22.28,7.72,7.6,1

23/11/2009,00:54:09,22.42,7.58,7.7,1

23/11/2009,00:59:03,24.05,5.95,5.9,1

23/11/2009,00:58:55,24.09,5.91,6.0,1

23/11/2009,00:59:08,25.01,4.99,5.0,1

23/11/2009,00:59:44,26.04,3.96,4.1,1

23/11/2009,00:59:38,25.86,4.14,4.1,1

23/11/2009,00:59:36,25.89,4.11,4.0,1

23/11/2009,01:06:05,22.99,7.01,7.2,1

23/11/2009,01:06:03,22.92,7.08,7.1,1

23/11/2009,01:06:01,22.81,7.19,7.0,1

23/11/2009,01:10:49,25.15,4.85,5.0,1

23/11/2009,01:10:46,24.97,5.03,4.9,1

23/11/2009,01:11:23,26.28,3.72,3.8,1

23/11/2009,01:11:19,26.11,3.89,3.9,1

23/11/2009,01:11:17,26.18,3.82,3.9,2

23/11/2009,01:11:16,26.11,3.89,3.8,1

23/11/2009,01:11:15,26.11,3.89,3.9,1

23/11/2009,01:11:14,26.18,3.82,3.7,1

23/11/2009,01:16:54,25.75,4.25,4.3,1

23/11/2009,01:26:35,7.01,22.99,11.2,1

23/11/2009,01:26:41,6.87,23.13,23.0,1

23/11/2009,01:26:15,18.77,11.23,23.1,1

23/11/2009,01:27:16,21.71,8.29,8.3,1

23/11/2009,01:27:19,22,8,11.4,1

23/11/2009,01:27:16,18.56,11.44,8.0,1

23/11/2009,01:27:28,22.03,7.97,11.4,1

23/11/2009,01:27:19,18.6,11.4,8.0,1

23/11/2009,01:27:43,21.96,8.04,10.5,1

23/11/2009,01:27:45,22.25,7.75,9.7,1

23/11/2009,01:27:28,19.48,10.52,8.0,1

23/11/2009,01:28:10,23.13,6.87,7.8,2

23/11/2009,01:28:00,23.02,6.98,6.7,1

23/11/2009,01:28:00,23.31,6.69,7.0,1

23/11/2009,01:27:39,20.26,9.74,6.9,1

23/11/2009,01:31:08,19.8,10.2,10.2,1

23/11/2009,01:31:15,19.8,10.2,10.0,1

23/11/2009,01:31:15,20.01,9.99,10.2,1

23/11/2009,01:31:18,19.94,10.06,10.1,1

23/11/2009,01:31:20,19.8,10.2,10.2,1

23/11/2009,01:31:20,19.09,10.91,10.9,1

23/11/2009,01:31:24,20.01,9.99,10.0,2

23/11/2009,01:33:51,24.73,5.27,5.3,1

23/11/2009,01:34:03,24.83,5.17,5.2,1

23/11/2009,01:34:05,25.08,4.92,4.9,1

23/11/2009,01:34:12,25.86,4.14,4.1,1

23/11/2009,01:34:13,25.86,4.14,4.1,1

23/11/2009,01:42:37,27.2,2.8,2.8,1

23/11/2009,01:45:31,22.67,7.33,6.8,1

23/11/2009,01:45:30,23.2,6.8,7.3,1

23/11/2009,01:46:06,9.92,20.08,20.1,1

23/11/2009,01:46:20,14.91,15.09,15.1,1

23/11/2009,01:50:38,25.08,4.92,4.7,1

23/11/2009,01:50:37,25.36,4.64,4.6,1

23/11/2009,01:50:36,25.33,4.67,4.9,1

23/11/2009,01:52:23,25.29,4.71,4.7,1

23/11/2009,01:52:22,25.29,4.71,4.7,1

23/11/2009,01:52:29,25.54,4.46,4.5,1

23/11/2009,01:52:32,25.65,4.35,4.4,1

23/11/2009,01:53:41,27.06,2.94,5.2,1

23/11/2009,01:53:31,26.82,3.18,5.1,1

23/11/2009,01:53:18,24.87,5.13,3.2,1

23/11/2009,01:53:16,24.83,5.17,2.9,1

23/11/2009,01:54:55,22.42,7.58,7.6,1

23/11/2009,01:55:19,21.29,8.71,8.7,1

23/11/2009,01:56:04,19.23,10.77,10.8,1

23/11/2009,02:02:13,20.07,9.93,9.9,1

23/11/2009,02:02:15,21.02,8.98,9.0,1

23/11/2009,02:02:19,22.24,7.76,7.8,1

23/11/2009,02:03:10,23.71,6.29,6.3,1

23/11/2009,02:03:08,23.46,6.54,6.4,1

23/11/2009,02:03:06,23.64,6.36,6.5,1

23/11/2009,02:03:06,23.74,6.26,6.3,1

23/11/2009,02:05:00,20.18,9.82,9.8,1

23/11/2009,02:05:08,21.37,8.63,8.8,1

23/11/2009,02:05:09,21.68,8.32,8.6,1

23/11/2009,02:05:01,21.19,8.81,8.3,1

23/11/2009,02:09:43,24.86,5.14,5.1,1

23/11/2009,02:13:02,15.77,14.23,14.1,3

23/11/2009,02:13:01,15.88,14.12,14.2,1

23/11/2009,02:24:57,20.91,9.09,6.9,1

23/11/2009,02:25:15,23.32,6.68,7.2,1

23/11/2009,02:23:28,24.79,5.21,5.2,1

23/11/2009,02:23:19,24.76,5.24,5.2,1

23/11/2009,02:22:29,22.83,7.17,9.1,1

23/11/2009,02:22:21,23.11,6.89,6.7,1

23/11/2009,02:25:44,8.18,21.82,21.8,1

23/11/2009,02:27:33,8.78,21.22,21.2,1

23/11/2009,02:27:40,10.6,19.4,19.4,1

23/11/2009,02:29:14,18.64,11.36,11.4,1

23/11/2009,02:29:19,19.16,10.84,10.8,1

23/11/2009,02:32:06,18.6,11.4,5.5,1

23/11/2009,02:32:02,18.15,11.85,5.6,1

23/11/2009,02:31:46,24.41,5.59,11.9,1

23/11/2009,02:31:44,24.48,5.52,11.4,1

23/11/2009,02:35:10,19.55,10.45,10.5,1

23/11/2009,02:35:15,21.33,8.67,8.7,1

23/11/2009,02:35:16,21.96,8.04,8.0,1

23/11/2009,02:37:06,23.01,6.99,7.0,1

23/11/2009,02:37:08,23.32,6.68,6.7,1

23/11/2009,02:38:54,24.69,5.31,5.4,1

23/11/2009,02:38:53,24.62,5.38,5.3,1

23/11/2009,02:41:46,23.64,6.36,6.4,1

23/11/2009,02:41:49,23.53,6.47,6.5,1

23/11/2009,02:44:08,24.23,5.77,5.7,1

23/11/2009,02:44:06,24.34,5.66,5.8,1

23/11/2009,02:46:53,25.56,4.44,4.4,1

23/11/2009,02:46:53,25.49,4.51,4.5,2

23/11/2009,02:50:48,23.99,6.01,14.5,1

23/11/2009,02:50:47,24.51,5.49,5.5,1

23/11/2009,02:50:58,24.34,5.66,6.0,1

23/11/2009,02:50:14,15.53,14.47,5.7,1

23/11/2009,02:52:10,17.03,12.97,13.0,1

23/11/2009,02:55:58,22.76,7.24,10.0,1

23/11/2009,02:55:59,22.83,7.17,7.2,1

23/11/2009,02:56:20,25.35,4.65,7.2,1

23/11/2009,02:55:16,20,10,4.7,1

23/11/2009,03:04:52,22.48,7.52,7.6,2

23/11/2009,03:04:51,22.38,7.62,7.5,1

23/11/2009,03:06:55,24.69,5.31,5.3,1

23/11/2009,03:15:00,23.88,6.12,6.1,1

23/11/2009,03:17:22,23.6,6.4,6.0,1

23/11/2009,03:17:22,24.02,5.98,6.4,1

23/11/2009,03:17:32,24.09,5.91,5.9,1

23/11/2009,03:17:37,24.02,5.98,6.0,2

23/11/2009,03:19:02,19.72,10.28,3.7,1

23/11/2009,03:21:19,23.81,6.19,10.3,1

23/11/2009,03:21:22,23.81,6.19,6.2,1

23/11/2009,03:22:27,24.55,5.45,6.2,1

23/11/2009,03:21:55,24.65,5.35,5.4,1

23/11/2009,03:22:00,25.39,4.61,4.6,1

23/11/2009,03:18:57,26.26,3.74,5.5,1

23/11/2009,03:22:40,25.18,4.82,4.8,1

23/11/2009,03:22:38,25.18,4.82,4.8,2

23/11/2009,03:27:59,15.63,14.37,14.4,1

23/11/2009,03:28:13,16.54,13.46,13.5,1

23/11/2009,03:28:15,16.58,13.42,13.4,1

23/11/2009,03:28:19,16.65,13.35,13.4,1

23/11/2009,03:28:20,16.75,13.25,13.3,1

23/11/2009,03:28:25,17.48,12.52,12.5,1

23/11/2009,03:28:27,17.52,12.48,12.5,1

23/11/2009,03:42:02,18.57,11.43,11.4,1

23/11/2009,03:52:28,20.7,9.3,9.4,1

23/11/2009,03:52:26,20.6,9.4,9.3,1

23/11/2009,03:58:18,20.63,9.37,9.4,1

23/11/2009,03:59:56,23.15,6.85,6.9,1

23/11/2009,04:00:08,23.25,6.75,8.8,1

23/11/2009,04:00:08,23.32,6.68,6.7,1

23/11/2009,04:02:54,24.48,5.52,6.8,1

23/11/2009,04:02:57,24.48,5.52,4.8,1

23/11/2009,04:02:50,25.21,4.79,4.8,1

23/11/2009,04:00:02,21.23,8.77,5.5,1

23/11/2009,04:02:56,24.34,5.66,5.5,1

23/11/2009,04:02:54,24.48,5.52,5.7,1

23/11/2009,04:02:50,25.21,4.79,5.5,1

23/11/2009,04:06:23,8.78,21.22,21.1,1

23/11/2009,04:06:23,8.92,21.08,21.2,2

23/11/2009,04:06:32,18.53,11.47,5.0,1

23/11/2009,04:06:34,24.86,5.14,11.5,1

23/11/2009,04:06:32,24.97,5.03,5.1,1

23/11/2009,04:07:57,15.25,14.75,14.7,1

23/11/2009,04:07:56,15.39,14.61,14.6,1

23/11/2009,04:07:54,15.35,14.65,14.8,1

23/11/2009,04:08:53,21.16,8.84,8.8,1

23/11/2009,04:10:41,22.69,7.31,18.8,1

23/11/2009,04:10:39,22.87,7.13,7.1,1

23/11/2009,04:09:31,11.16,18.84,7.3,1

23/11/2009,04:14:07,22.48,7.52,7.5,1

23/11/2009,04:14:22,18.6,11.4,11.4,1

23/11/2009,04:14:23,18.5,11.5,11.5,1

23/11/2009,04:19:37,17.83,12.17,10.1,1

23/11/2009,04:19:40,17.97,12.03,12.2,1

23/11/2009,04:19:43,17.83,12.17,12.0,1

23/11/2009,04:19:48,17.87,12.13,12.2,1

23/11/2009,04:19:48,17.76,12.24,12.5,1

23/11/2009,04:19:48,17.69,12.31,12.1,1

23/11/2009,04:19:47,17.48,12.52,12.2,1

23/11/2009,04:20:06,18.74,11.26,12.3,1

23/11/2009,04:18:26,19.86,10.14,11.3,1

23/11/2009,04:20:29,20.67,9.33,9.3,1

23/11/2009,04:21:50,22.69,7.31,7.3,1

23/11/2009,04:21:50,22.76,7.24,7.2,1

23/11/2009,04:21:47,22.76,7.24,7.2,1

23/11/2009,04:21:45,22.66,7.34,7.3,1

23/11/2009,04:21:53,20.25,9.75,9.8,2

23/11/2009,04:21:52,20.25,9.75,9.8,1

23/11/2009,04:22:24,22.73,7.27,7.2,1

23/11/2009,04:22:23,22.76,7.24,7.3,1

23/11/2009,04:22:27,22.76,7.24,7.2,1

23/11/2009,04:27:04,18.71,11.29,11.6,1

23/11/2009,04:27:11,21.02,8.98,11.9,1

23/11/2009,04:27:11,21.12,8.88,11.2,1

23/11/2009,04:27:12,21.26,8.74,11.3,1

23/11/2009,04:27:49,24.23,5.77,8.9,2

23/11/2009,04:26:29,18.36,11.64,9.0,1

23/11/2009,04:26:40,18.08,11.92,8.7,1

23/11/2009,04:26:46,18.81,11.19,5.8,1

23/11/2009,04:30:08,15.39,14.61,14.4,1

23/11/2009,04:30:05,15.25,14.75,14.8,1

23/11/2009,04:30:02,15.6,14.4,14.6,1

23/11/2009,04:31:04,18.74,11.26,11.3,1

23/11/2009,04:31:09,18.88,11.12,11.1,1

23/11/2009,04:31:11,18.81,11.19,11.2,1

23/11/2009,04:31:12,18.78,11.22,11.2,1

23/11/2009,04:31:14,18.78,11.22,11.2,2

23/11/2009,04:31:53,10.14,19.86,19.9,2

23/11/2009,04:31:53,9.97,20.03,20.0,1

23/11/2009,04:31:54,9.79,20.21,20.2,4

23/11/2009,04:37:15,22.76,7.24,12.0,1

23/11/2009,04:37:10,22.76,7.24,12.2,1

23/11/2009,04:36:52,17.8,12.2,7.2,1

23/11/2009,04:36:51,17.97,12.03,7.2,1

23/11/2009,04:38:22,13.25,16.75,16.8,1

23/11/2009,04:38:35,17.2,12.8,16.5,1

23/11/2009,04:38:32,13.5,16.5,16.5,1

23/11/2009,04:38:28,13.5,16.5,12.8,2

23/11/2009,04:39:50,21.72,8.28,8.4,1

23/11/2009,04:39:39,21.61,8.39,8.4,2

23/11/2009,04:39:37,21.61,8.39,8.3,1

23/11/2009,04:40:20,22.76,7.24,7.2,1

23/11/2009,04:41:44,18.25,11.75,7.2,1

23/11/2009,04:42:55,17.24,12.76,7.3,1

23/11/2009,04:42:53,17.38,12.62,11.8,1

23/11/2009,04:42:51,17.52,12.48,12.5,1

23/11/2009,04:42:52,20.7,9.3,9.2,1

23/11/2009,04:42:52,20.84,9.16,9.3,1

23/11/2009,04:43:27,21.61,8.39,12.6,1

23/11/2009,04:43:22,18.32,11.68,12.8,1

23/11/2009,04:43:20,18.32,11.68,11.7,1

23/11/2009,04:43:50,22.24,7.76,11.7,2

23/11/2009,04:43:47,22.14,7.86,8.4,1

23/11/2009,04:44:12,22.17,7.83,7.9,4

23/11/2009,04:40:22,22.73,7.27,7.8,1

23/11/2009,04:40:20,22.76,7.24,7.8,1

23/11/2009,04:44:12,22.21,7.79,7.8,3

23/11/2009,04:44:23,22.52,7.48,7.5,1

23/11/2009,04:44:24,22.59,7.41,7.4,1

23/11/2009,04:44:51,22.35,7.65,7.7,1

23/11/2009,04:46:20,22.35,7.65,7.7,1

23/11/2009,04:47:48,23.67,6.33,6.6,1

23/11/2009,04:47:41,23.43,6.57,6.3,1

23/11/2009,04:47:53,24.41,5.59,5.6,1

23/11/2009,04:48:58,20.07,9.93,14.1,1

23/11/2009,04:48:47,16.02,13.98,14.0,1

23/11/2009,04:48:44,15.91,14.09,9.9,1

23/11/2009,04:49:16,20.74,9.26,9.3,1

23/11/2009,04:49:25,12.59,17.41,17.4,1

23/11/2009,04:49:58,13.99,16.01,16.0,1

23/11/2009,04:50:28,15.6,14.4,11.2,1

23/11/2009,04:50:38,20.53,9.47,14.4,1

23/11/2009,04:50:38,20.67,9.33,3.9,1

23/11/2009,04:50:26,18.81,11.19,9.3,2

23/11/2009,04:50:33,26.09,3.91,9.5,2

23/11/2009,04:50:56,23.95,6.05,6.0,2

23/11/2009,04:50:55,24.02,5.98,6.1,1

23/11/2009,04:51:25,21.33,8.67,8.7,2

23/11/2009,04:51:29,21.61,8.39,8.4,2

23/11/2009,04:51:30,19.97,10.03,10.0,1

23/11/2009,04:51:31,20.11,9.89,9.9,1

23/11/2009,04:52:08,22.62,7.38,7.4,1

23/11/2009,04:52:11,23.39,6.61,15.2,1

23/11/2009,04:52:12,15.81,14.19,6.6,1

23/11/2009,04:52:16,16.23,13.77,14.2,1

23/11/2009,04:52:09,14.83,15.17,14.1,1

23/11/2009,04:52:15,15.91,14.09,13.8,1

23/11/2009,04:52:24,17.17,12.83,12.8,1

23/11/2009,04:52:25,17.8,12.2,12.2,1

23/11/2009,04:53:26,22.76,7.24,7.2,1

23/11/2009,04:53:27,22.9,7.1,19.3,1

23/11/2009,04:53:28,22.97,7.03,7.1,1

23/11/2009,04:53:39,23.39,6.61,7.0,1

23/11/2009,04:53:43,23.53,6.47,6.6,1

23/11/2009,04:53:58,24.51,5.49,6.5,1

23/11/2009,04:53:56,24.55,5.45,5.4,1

23/11/2009,04:53:55,24.58,5.42,5.5,1

23/11/2009,04:53:26,10.67,19.33,5.5,2

23/11/2009,04:54:14,12.55,17.45,17.5,1

23/11/2009,04:56:26,24.41,5.59,5.6,1

23/11/2009,04:56:24,24.44,5.56,5.6,1

23/11/2009,05:02:15,17.55,12.45,12.5,1

23/11/2009,05:02:29,21.23,8.77,8.8,1

23/11/2009,05:02:31,19.23,10.77,10.8,1

23/11/2009,05:05:15,21.79,8.21,8.2,1

23/11/2009,05:07:22,13.25,16.75,16.8,1

23/11/2009,05:07:24,13.36,16.64,16.6,1

23/11/2009,05:09:18,10.25,19.75,19.8,1

23/11/2009,05:12:42,19.69,10.31,10.3,1

23/11/2009,05:14:12,21.72,8.28,8.3,2

23/11/2009,05:14:46,18.25,11.75,11.8,1

23/11/2009,05:14:49,18.29,11.71,11.7,1

23/11/2009,05:20:50,23.15,6.85,6.9,1

23/11/2009,05:24:49,22.76,7.24,7.4,1

23/11/2009,05:24:52,22.8,7.2,7.2,2

23/11/2009,05:24:39,22.62,7.38,7.2,1

23/11/2009,05:27:55,23.46,6.54,6.5,1

23/11/2009,05:32:23,23.01,6.99,6.2,1

23/11/2009,05:32:23,23.78,6.22,7.0,1

23/11/2009,05:35:49,23.08,6.92,6.9,1

23/11/2009,05:37:57,17.94,12.06,12.2,1

23/11/2009,05:37:55,17.76,12.24,12.1,1

23/11/2009,05:38:05,22,8,8.0,1

23/11/2009,05:38:07,22.07,7.93,7.9,1

23/11/2009,05:40:50,20.53,9.47,7.7,1

23/11/2009,05:40:47,20.46,9.54,7.8,1

23/11/2009,05:40:49,22.73,7.27,7.7,1

23/11/2009,05:40:37,22.35,7.65,9.5,1

23/11/2009,05:40:36,22.28,7.72,7.3,1

23/11/2009,05:40:36,22.24,7.76,9.5,1

23/11/2009,05:42:26,19.16,10.84,10.8,1

23/11/2009,05:43:45,15.42,14.58,14.9,2

23/11/2009,05:43:38,15.18,14.82,14.9,1

23/11/2009,05:43:37,15.07,14.93,14.8,1

23/11/2009,05:44:18,20.98,9.02,14.6,1

23/11/2009,05:44:16,20.91,9.09,9.1,1

23/11/2009,05:43:32,15.11,14.89,9.0,1

23/11/2009,05:46:22,18.74,11.26,11.3,1

23/11/2009,05:47:42,20.39,9.61,9.7,1

23/11/2009,05:47:40,20.35,9.65,9.6,1

23/11/2009,05:48:24,22.87,7.13,7.1,1

23/11/2009,05:48:32,23.08,6.92,6.9,1

23/11/2009,05:54:48,19.2,10.8,11.1,1

23/11/2009,05:54:45,18.92,11.08,10.8,1

23/11/2009,05:57:14,18.01,11.99,12.0,1

23/11/2009,05:57:45,15.07,14.93,14.9,1

23/11/2009,05:57:48,15.11,14.89,14.9,1

23/11/2009,05:58:23,16.4,13.6,13.6,1

23/11/2009,05:58:53,21.61,8.39,10.8,1

23/11/2009,05:58:54,21.51,8.49,8.4,1

23/11/2009,05:58:51,19.16,10.84,8.5,1

23/11/2009,05:58:56,18.99,11.01,11.0,1

23/11/2009,05:59:11,23.71,6.29,6.3,1

23/11/2009,05:59:18,22.03,7.97,10.5,1

23/11/2009,05:59:14,19.3,10.7,10.7,1

23/11/2009,05:59:12,19.51,10.49,8.0,1

23/11/2009,06:00:08,16.23,13.77,13.8,1

23/11/2009,06:00:09,16.12,13.88,13.9,1

23/11/2009,06:00:41,18.29,11.71,11.4,2

23/11/2009,06:00:40,18.43,11.57,11.6,1

23/11/2009,06:00:40,18.57,11.43,11.7,1

23/11/2009,06:02:42,20.91,9.09,9.1,1

23/11/2009,06:08:53,17.1,12.9,12.9,1

23/11/2009,06:08:59,17.48,12.52,12.5,1

23/11/2009,06:09:08,18.15,11.85,11.9,1

23/11/2009,06:09:11,18.11,11.89,11.9,1

23/11/2009,06:09:44,19.41,10.59,10.6,1

23/11/2009,06:09:52,19.62,10.38,10.4,1

23/11/2009,06:11:17,22.97,7.03,7.0,1

23/11/2009,06:12:48,19.72,10.28,10.3,1

23/11/2009,06:21:10,18.15,11.85,11.9,1

23/11/2009,06:21:19,22.24,7.76,7.8,2

23/11/2009,06:21:17,22.17,7.83,7.8,2

23/11/2009,06:22:08,24.65,5.35,6.8,1

23/11/2009,06:22:14,24.23,5.77,5.4,1

23/11/2009,06:22:20,24.27,5.73,5.7,1

23/11/2009,06:22:03,23.18,6.82,5.8,1

23/11/2009,06:22:14,24.34,5.66,5.7,1

23/11/2009,06:24:28,24.51,5.49,5.5,1

23/11/2009,06:24:27,24.51,5.49,5.5,1

23/11/2009,06:37:38,22.45,7.55,7.6,4

23/11/2009,06:37:36,22.38,7.62,7.6,3

23/11/2009,15:41:35,20.53,9.47,9.5,1

23/11/2009,15:41:31,20.53,9.47,9.5,1

23/11/2009,16:02:01,24.41,5.59,5.6,1

23/11/2009,16:52:32,23.67,6.33,6.3,1

23/11/2009,16:52:34,24.3,5.7,5.6,1

23/11/2009,16:52:34,24.41,5.59,5.7,1

23/11/2009,17:23:52,24.06,5.94,6.1,1

23/11/2009,17:23:49,23.99,6.01,6.0,2

23/11/2009,17:23:47,23.95,6.05,5.9,1

23/11/2009,17:40:56,21.79,8.21,8.2,1

23/11/2009,17:41:25,22.9,7.1,7.1,1

23/11/2009,17:41:33,22.97,7.03,7.0,1

23/11/2009,18:00:48,20,10,9.7,1

23/11/2009,18:00:47,20.28,9.72,10.0,1

23/11/2009,20:07:51,23.52,6.48,6.5,1

23/11/2009,20:07:54,24.01,5.99,6.0,1

23/11/2009,20:07:56,24.3,5.7,5.7,1

23/11/2009,20:07:57,24.27,5.73,5.7,1

23/11/2009,20:38:12,24.82,5.18,5.2,1

23/11/2009,21:13:01,21.66,8.34,8.3,1

23/11/2009,21:13:02,21.32,8.68,8.7,1

23/11/2009,21:13:13,23.21,6.79,6.8,1

23/11/2009,21:15:54,13.63,16.37,16.4,1

23/11/2009,21:48:18,22.41,7.59,7.6,1

23/11/2009,21:56:00,17.16,12.84,13.4,1

23/11/2009,21:55:59,16.64,13.36,12.8,1

23/11/2009,21:56:16,22.49,7.51,7.5,2

23/11/2009,22:40:24,24.61,5.39,5.4,1

23/11/2009,22:40:26,24.5,5.5,5.5,1

23/11/2009,22:42:57,24.04,5.96,18.3,1

23/11/2009,22:42:59,24.13,5.87,17.6,1

23/11/2009,22:42:09,11.74,18.26,6.0,1

23/11/2009,22:42:13,12.43,17.57,5.9,1

23/11/2009,22:45:02,17.19,12.81,12.8,1

23/11/2009,22:44:48,17.22,12.78,12.8,1

23/11/2009,22:54:01,26.36,3.64,3.6,1

23/11/2009,22:59:17,18.48,11.52,11.5,1

23/11/2009,22:59:19,18.36,11.64,11.6,1

23/11/2009,23:00:00,20.94,9.06,9.1,1

23/11/2009,23:00:03,20.97,9.03,9.0,1

23/11/2009,23:16:51,23.04,6.96,6.9,1

23/11/2009,23:16:47,23.09,6.91,7.0,1

23/11/2009,23:17:13,24.07,5.93,5.7,2

23/11/2009,23:17:13,24.16,5.84,5.8,1

23/11/2009,23:17:13,24.33,5.67,5.9,1

23/11/2009,23:40:34,18.65,11.35,11.4,1

23/11/2009,23:53:58,21.52,8.48,8.5,1

23/11/2009,23:54:17,22.41,7.59,7.6,1

23/11/2009,23:54:18,22.64,7.36,7.4,1

24/11/2009,00:29:47,22.35,7.65,7.6,1

24/11/2009,00:29:44,22.38,7.62,7.6,1

24/11/2009,00:29:42,22.41,7.59,7.7,1

24/11/2009,00:30:02,22.95,7.05,7.1,1

24/11/2009,00:30:05,23.07,6.93,6.9,1

24/11/2009,00:46:20,26.42,3.58,2.7,1

24/11/2009,00:46:19,26.48,3.52,3.5,1

24/11/2009,00:46:32,26.28,3.72,3.6,1

24/11/2009,00:46:38,26.31,3.69,3.7,1

24/11/2009,00:46:09,27.34,2.66,3.7,1

24/11/2009,01:03:18,16.16,13.84,13.8,1

24/11/2009,01:03:35,17.62,12.38,12.4,1

24/11/2009,01:03:37,18.05,11.95,12.0,1

24/11/2009,01:03:41,17.99,12.01,12.0,1

24/11/2009,01:03:42,17.99,12.01,12.0,1

24/11/2009,01:03:45,18.48,11.52,11.5,1

24/11/2009,01:08:22,22.89,7.11,7.1,1

24/11/2009,01:08:24,23.15,6.85,6.9,1

24/11/2009,01:08:25,23.07,6.93,6.9,1

24/11/2009,01:08:26,23.12,6.88,6.9,1

24/11/2009,01:08:33,23.24,6.76,6.8,1

24/11/2009,01:08:35,23.5,6.5,6.5,1

24/11/2009,01:09:45,24.47,5.53,5.5,1

24/11/2009,01:09:47,24.47,5.53,5.5,2

24/11/2009,01:11:32,15.3,14.7,14.2,1

24/11/2009,01:11:30,15.44,14.56,14.4,1

24/11/2009,01:11:24,15.07,14.93,14.9,1

24/11/2009,01:11:23,15.58,14.42,14.6,3

24/11/2009,01:11:23,15.78,14.22,14.7,2

24/11/2009,01:23:43,24.53,5.47,5.5,1

24/11/2009,01:27:20,17.93,12.07,12.1,1

24/11/2009,01:27:29,18.82,11.18,11.2,1

24/11/2009,01:27:32,19.08,10.92,10.9,2

24/11/2009,01:27:35,18.97,11.03,11.0,2

24/11/2009,01:27:41,20.06,9.94,9.9,1

24/11/2009,01:27:43,19.8,10.2,10.0,1

24/11/2009,01:27:43,20,10,10.2,1

24/11/2009,01:27:44,20.11,9.89,9.9,1

24/11/2009,01:28:48,27.17,2.83,2.8,1

24/11/2009,01:28:53,27.31,2.69,2.7,1

24/11/2009,01:29:35,24.96,5.04,5.0,2

24/11/2009,01:29:38,25.13,4.87,4.9,1

24/11/2009,01:31:57,27.51,2.49,2.6,1

24/11/2009,01:31:56,27.51,2.49,2.5,1

24/11/2009,01:31:56,27.4,2.6,2.6,1

24/11/2009,01:31:55,27.37,2.63,2.5,1

24/11/2009,01:36:08,24.9,5.1,5.1,1

24/11/2009,01:36:12,24.99,5.01,5.0,1

24/11/2009,01:36:13,25.27,4.73,4.7,1

24/11/2009,01:36:14,25.13,4.87,4.9,1

24/11/2009,01:39:50,20.71,9.29,9.2,1

24/11/2009,01:39:49,20.8,9.2,9.3,1

24/11/2009,01:40:02,21.63,8.37,8.4,1

24/11/2009,01:40:04,21.63,8.37,8.2,1

24/11/2009,01:40:04,21.78,8.22,8.4,1

24/11/2009,01:40:23,24.33,5.67,5.7,1

24/11/2009,01:42:57,20.8,9.2,9.2,1

24/11/2009,01:47:04,21.63,8.37,8.4,1

24/11/2009,01:49:38,27.31,2.69,2.8,1

24/11/2009,01:49:37,27.22,2.78,2.7,1

24/11/2009,01:50:20,23.58,6.42,6.4,1

24/11/2009,01:50:28,24.73,5.27,5.3,1

24/11/2009,01:50:40,8.36,21.64,22.0,1

24/11/2009,01:50:37,8.27,21.73,21.7,1

24/11/2009,01:50:31,8.04,21.96,21.6,1

24/11/2009,01:55:32,24.16,5.84,10.5,1

24/11/2009,01:55:38,24.27,5.73,6.3,1

24/11/2009,01:54:51,23.7,6.3,5.8,1

24/11/2009,01:54:38,19.51,10.49,5.7,1

24/11/2009,01:56:32,24.41,5.59,5.8,1

24/11/2009,01:56:29,24.21,5.79,5.8,1

24/11/2009,01:56:28,24.24,5.76,5.6,1

24/11/2009,01:57:25,16.67,13.33,13.3,1

24/11/2009,02:08:53,20.03,9.97,10.0,1

24/11/2009,02:08:58,20.11,9.89,9.9,1

24/11/2009,02:09:27,22.06,7.94,7.9,1

24/11/2009,02:09:30,22.03,7.97,8.0,2

24/11/2009,02:11:40,25.02,4.98,5.0,1

24/11/2009,02:11:40,25.02,4.98,5.0,1

24/11/2009,02:11:46,25.36,4.64,4.6,1

24/11/2009,02:12:14,25.25,4.75,4.8,1

24/11/2009,02:12:16,25.07,4.93,4.9,2

24/11/2009,02:12:52,25.02,4.98,5.0,1

24/11/2009,02:14:04,25.59,4.41,4.4,2

24/11/2009,02:14:20,25.85,4.15,4.2,1

24/11/2009,02:14:25,25.76,4.24,4.2,1

24/11/2009,02:15:29,22.41,7.59,7.6,1

24/11/2009,02:15:30,22.58,7.42,7.4,1

24/11/2009,02:15:31,22.78,7.22,7.2,1

24/11/2009,02:15:37,24.33,5.67,5.7,1

24/11/2009,02:16:18,19.02,10.98,11.0,1

24/11/2009,02:16:25,20.06,9.94,9.9,1

24/11/2009,02:16:42,20.49,9.51,9.5,1

24/11/2009,02:22:01,24.7,5.3,5.3,1

24/11/2009,02:22:03,24.44,5.56,5.6,1

24/11/2009,02:22:06,24.61,5.39,5.4,1

24/11/2009,02:28:27,21.09,8.91,5.8,1

24/11/2009,02:28:25,21.17,8.83,8.8,1

24/11/2009,02:28:30,23.44,6.56,8.9,1

24/11/2009,02:28:29,23.47,6.53,6.5,1

24/11/2009,02:28:49,22.15,7.85,6.6,1

24/11/2009,02:28:52,22.23,7.77,7.9,1

24/11/2009,02:28:23,24.24,5.76,7.8,1

24/11/2009,02:29:58,26.34,3.66,3.6,1

24/11/2009,02:29:57,26.45,3.55,3.7,1

24/11/2009,02:31:19,24.79,5.21,5.2,1

24/11/2009,02:32:06,18.74,11.26,11.0,1

24/11/2009,02:32:06,19.02,10.98,11.3,1

24/11/2009,02:32:07,19.97,10.03,10.0,1

24/11/2009,02:32:15,21.89,8.11,8.1,1

24/11/2009,02:32:16,22.01,7.99,8.0,1

24/11/2009,02:33:21,13.66,16.34,17.6,1

24/11/2009,02:33:20,13.6,16.4,17.0,1

24/11/2009,02:33:17,13,17,16.4,1

24/11/2009,02:34:03,13.86,16.14,16.3,1

24/11/2009,02:34:04,14.21,15.79,15.9,1

24/11/2009,02:34:02,14.06,15.94,15.5,1

24/11/2009,02:32:29,12.43,17.57,16.1,1

24/11/2009,02:34:05,14.72,15.28,15.8,1

24/11/2009,02:34:03,14.55,15.45,15.3,1

24/11/2009,02:34:17,20.74,9.26,9.3,1

24/11/2009,02:37:47,24.82,5.18,5.2,2

24/11/2009,02:37:55,24.93,5.07,5.1,1

24/11/2009,02:37:58,24.82,5.18,5.2,1

24/11/2009,02:38:11,25.13,4.87,4.9,1

24/11/2009,02:41:05,26.82,3.18,3.2,1

24/11/2009,02:41:02,26.82,3.18,3.2,1

24/11/2009,02:41:31,25.19,4.81,4.8,1

24/11/2009,02:41:31,25.45,4.55,4.6,2

24/11/2009,02:41:30,25.16,4.84,4.8,1

24/11/2009,02:46:04,25.07,4.93,4.9,1

24/11/2009,02:46:04,24.56,5.44,5.4,1

24/11/2009,02:50:36,24.9,5.1,4.3,1

24/11/2009,02:50:36,25.07,4.93,4.6,1

24/11/2009,02:50:36,25.22,4.78,4.8,2

24/11/2009,02:50:36,25.42,4.58,4.9,1

24/11/2009,02:50:35,25.68,4.32,5.1,1

24/11/2009,02:52:11,17.47,12.53,12.5,1

24/11/2009,02:52:14,17.76,12.24,12.2,1

24/11/2009,02:52:18,17.68,12.32,12.3,1

24/11/2009,02:52:20,17.68,12.32,12.3,1

24/11/2009,02:52:23,17.88,12.12,12.1,1

24/11/2009,02:52:33,18.48,11.52,11.5,1

24/11/2009,02:52:54,19.6,10.4,10.4,1

24/11/2009,02:53:00,23.78,6.22,5.2,1

24/11/2009,02:52:59,24.47,5.53,5.5,1

24/11/2009,02:52:59,24.82,5.18,6.2,1

24/11/2009,02:53:12,24.9,5.1,9.9,2

24/11/2009,02:53:14,24.82,5.18,10.2,1

24/11/2009,02:53:01,19.83,10.17,5.1,1

24/11/2009,02:53:01,20.06,9.94,5.2,1

24/11/2009,02:53:21,16.01,13.99,14.0,1

24/11/2009,02:53:23,15.93,14.07,14.1,1

24/11/2009,02:54:38,7.98,22.02,4.1,1

24/11/2009,02:53:57,25.93,4.07,22.0,1

24/11/2009,02:55:40,23.61,6.39,5.0,1

24/11/2009,02:55:39,23.7,6.3,6.3,1

24/11/2009,02:55:33,23.67,6.33,6.3,1

24/11/2009,02:55:32,25.04,4.96,6.4,1

24/11/2009,02:55:56,25.73,4.27,4.3,1

24/11/2009,02:56:48,22.64,7.36,4.5,1

24/11/2009,02:56:50,22.58,7.42,7.4,1

24/11/2009,02:57:17,17.02,12.98,7.4,1

24/11/2009,02:57:17,17.27,12.73,12.2,1

24/11/2009,02:57:17,17.65,12.35,12.4,2

24/11/2009,02:57:17,17.85,12.15,12.7,1

24/11/2009,02:56:48,25.5,4.5,13.0,1

24/11/2009,02:57:21,17.33,12.67,12.7,1

24/11/2009,02:57:18,17.3,12.7,12.7,1

24/11/2009,02:58:21,16.76,13.24,7.2,1

24/11/2009,02:58:19,16.76,13.24,7.6,1

24/11/2009,02:57:36,14.55,15.45,9.3,1

24/11/2009,02:57:35,14.55,15.45,12.1,1

24/11/2009,02:57:38,15.32,14.68,12.2,1

24/11/2009,02:57:41,18.25,11.75,15.5,1

24/11/2009,02:57:39,18.13,11.87,15.5,1

24/11/2009,02:57:29,17.79,12.21,14.7,1

24/11/2009,02:57:28,17.9,12.1,11.9,1

24/11/2009,02:57:24,20.71,9.29,11.8,1

24/11/2009,02:57:24,22.38,7.62,13.2,1

24/11/2009,02:57:24,22.78,7.22,13.2,1

24/11/2009,02:58:44,16.3,13.7,13.8,1

24/11/2009,02:58:42,16.24,13.76,13.7,2

24/11/2009,03:00:29,23.93,6.07,9.2,1

24/11/2009,03:00:25,20.83,9.17,6.1,1

24/11/2009,03:00:50,21.32,8.68,8.7,1

24/11/2009,03:00:52,21.29,8.71,8.7,1

24/11/2009,03:01:07,21.92,8.08,8.1,1

24/11/2009,03:01:11,22.78,7.22,7.2,1

24/11/2009,03:01:11,21.95,8.05,8.1,1

24/11/2009,03:01:48,21.43,8.57,8.5,1

24/11/2009,03:01:48,21.55,8.45,8.6,1

24/11/2009,03:01:53,21.98,8.02,7.5,1

24/11/2009,03:01:51,22.29,7.71,7.7,1

24/11/2009,03:01:51,22.52,7.48,8.0,1

24/11/2009,03:01:59,25.3,4.7,4.4,1

24/11/2009,03:01:59,25.59,4.41,4.7,1

24/11/2009,03:02:04,23.52,6.48,6.5,1

24/11/2009,03:02:04,23.32,6.68,6.7,1

24/11/2009,03:02:05,23.01,6.99,7.0,1

24/11/2009,03:02:30,25.19,4.81,3.8,2

24/11/2009,03:02:29,25.36,4.64,4.6,2

24/11/2009,03:02:23,26.16,3.84,4.8,1

24/11/2009,03:04:33,26.68,3.32,4.5,1

24/11/2009,03:04:10,25.47,4.53,3.3,1

24/11/2009,03:08:50,25.45,4.55,11.6,1

24/11/2009,03:08:47,25.42,4.58,11.3,1

24/11/2009,03:08:12,18.65,11.35,11.3,1

24/11/2009,03:08:10,18.68,11.32,11.4,1

24/11/2009,03:08:04,18.42,11.58,4.6,1

24/11/2009,03:08:05,18.74,11.26,4.6,1

24/11/2009,03:11:46,25.27,4.73,4.7,2

24/11/2009,03:12:37,17.96,12.04,12.0,1

24/11/2009,03:14:11,9.62,20.38,20.4,1

24/11/2009,03:17:22,18.54,11.46,11.7,1

24/11/2009,03:17:21,18.33,11.67,11.5,1

24/11/2009,03:17:53,24.18,5.82,5.8,1

24/11/2009,03:17:52,24.21,5.79,5.7,1

24/11/2009,03:17:53,24.27,5.73,5.8,2

24/11/2009,03:26:07,24.61,5.39,5.5,1

24/11/2009,03:26:02,24.47,5.53,5.4,1

24/11/2009,03:28:43,25.47,4.53,4.4,1

24/11/2009,03:28:42,25.56,4.44,4.5,1

24/11/2009,03:28:44,25.73,4.27,4.3,1

24/11/2009,03:29:30,19.14,10.86,11.0,1

24/11/2009,03:29:27,18.97,11.03,10.2,1

24/11/2009,03:29:45,20.11,9.89,10.9,1

24/11/2009,03:29:48,20.28,9.72,10.4,1

24/11/2009,03:29:32,19.57,10.43,9.9,1

24/11/2009,03:29:29,19.85,10.15,9.9,1

24/11/2009,03:29:44,20.06,9.94,9.7,1

24/11/2009,03:29:48,20.31,9.69,9.7,1

24/11/2009,03:30:33,22.75,7.25,7.3,1

24/11/2009,03:31:12,24.36,5.64,4.6,1

24/11/2009,03:31:10,24.61,5.39,5.4,1

24/11/2009,03:31:07,25.36,4.64,5.6,2

24/11/2009,03:31:20,21.43,8.57,8.5,1

24/11/2009,03:31:20,21.52,8.48,8.6,1

24/11/2009,03:36:32,13.78,16.22,8.9,2

24/11/2009,03:36:28,13.52,16.48,16.5,1

24/11/2009,03:36:01,21.12,8.88,16.2,1

24/11/2009,03:36:41,25.36,4.64,4.6,1

24/11/2009,03:39:07,10.19,19.81,19.8,1

24/11/2009,03:39:17,10.48,19.52,19.5,5

24/11/2009,03:40:48,19.77,10.23,6.3,1

24/11/2009,03:40:44,19.51,10.49,10.5,1

24/11/2009,03:40:02,23.7,6.3,10.2,1

24/11/2009,03:43:45,16.21,13.79,13.8,1

24/11/2009,03:43:43,16.18,13.82,13.8,1

24/11/2009,03:44:31,26.11,3.89,3.9,1

24/11/2009,03:44:28,26.13,3.87,3.9,1

24/11/2009,03:44:54,25.19,4.81,4.8,1

24/11/2009,03:45:01,25.73,4.27,4.3,1

24/11/2009,03:51:24,25.5,4.5,4.5,1

24/11/2009,03:53:32,22.29,7.71,7.5,1

24/11/2009,03:53:32,22.49,7.51,7.7,1

24/11/2009,03:54:52,25.21,4.79,4.8,1

24/11/2009,03:55:25,25.28,4.72,4.7,1

24/11/2009,03:55:26,25.25,4.75,4.8,1

24/11/2009,03:56:44,24.65,5.35,5.2,1

24/11/2009,03:56:42,24.79,5.21,5.4,1

24/11/2009,03:58:12,22.76,7.24,7.2,1

24/11/2009,03:58:22,22.97,7.03,7.0,1

24/11/2009,03:59:37,21.96,8.04,7.8,2

24/11/2009,03:59:39,21.86,8.14,8.0,1

24/11/2009,03:59:47,22.41,7.59,8.1,1

24/11/2009,03:59:49,22.35,7.65,7.6,1

24/11/2009,03:59:55,22.28,7.72,7.7,1

24/11/2009,03:59:33,22.17,7.83,7.7,2

24/11/2009,04:10:28,26.65,3.35,2.8,1

24/11/2009,04:10:26,26.61,3.39,3.4,1

24/11/2009,04:10:11,27.17,2.83,3.4,1

24/11/2009,04:12:52,25.42,4.58,4.5,2

24/11/2009,04:12:48,25.46,4.54,4.5,1

24/11/2009,04:12:46,25.49,4.51,4.6,1

24/11/2009,04:15:32,23.95,6.05,3.0,1

24/11/2009,04:15:28,24.09,5.91,5.9,1

24/11/2009,04:15:08,27.03,2.97,6.1,1

24/11/2009,04:20:11,18.01,11.99,2.8,1

24/11/2009,04:20:29,20.35,9.65,5.2,1

24/11/2009,04:20:27,19.97,10.03,3.3,1

24/11/2009,04:20:25,19.97,10.03,12.0,2

24/11/2009,04:20:25,19.97,10.03,10.0,1

24/11/2009,04:22:01,18.57,11.43,10.0,1

24/11/2009,04:19:58,24.83,5.17,10.0,1

24/11/2009,04:19:34,27.24,2.76,9.7,1

24/11/2009,04:20:10,26.68,3.32,11.4,1

24/11/2009,04:23:12,24.65,5.35,5.4,1

24/11/2009,04:24:02,22.8,7.2,7.2,2

24/11/2009,04:24:03,23.08,6.92,6.9,1

24/11/2009,04:24:05,23.01,6.99,7.0,1

24/11/2009,04:24:08,23.22,6.78,6.8,1

24/11/2009,04:24:11,23.25,6.75,6.8,1

24/11/2009,04:24:20,26.12,3.88,3.9,1

24/11/2009,04:27:31,25.07,4.93,5.2,1

24/11/2009,04:27:28,24.83,5.17,4.9,1

24/11/2009,04:33:13,20.46,9.54,8.8,1

24/11/2009,04:33:13,21.16,8.84,9.5,1

24/11/2009,04:37:12,14.02,15.98,15.7,2

24/11/2009,04:37:12,14.3,15.7,16.0,1

24/11/2009,04:39:44,17.55,12.45,12.2,1

24/11/2009,04:39:39,17.76,12.24,12.5,1

24/11/2009,04:40:10,24.9,5.1,5.1,1

24/11/2009,04:43:50,24.72,5.28,7.4,1

24/11/2009,04:43:49,24.9,5.1,7.5,1

24/11/2009,04:42:01,22.52,7.48,5.1,1

24/11/2009,04:41:59,22.59,7.41,5.3,2

24/11/2009,04:43:59,5.7,24.3,23.8,1

24/11/2009,04:43:58,5.84,24.16,23.9,1

24/11/2009,04:43:59,5.63,24.37,24.0,2

24/11/2009,04:43:59,5.81,24.19,24.2,1

24/11/2009,04:43:58,5.98,24.02,24.2,1

24/11/2009,04:43:58,6.12,23.88,24.3,1

24/11/2009,04:43:58,6.22,23.78,24.4,1

24/11/2009,04:47:04,18.57,11.43,11.4,1

24/11/2009,04:49:51,22.97,7.03,3.1,1

24/11/2009,04:49:48,22.87,7.13,3.3,1

24/11/2009,04:49:42,22.69,7.31,7.3,1

24/11/2009,04:49:28,26.75,3.25,7.1,1

24/11/2009,04:49:25,26.89,3.11,7.0,1

24/11/2009,04:50:56,15.53,14.47,15.1,1

24/11/2009,04:50:52,15.28,14.72,14.7,2

24/11/2009,04:50:52,15.35,14.65,14.7,1

24/11/2009,04:50:45,14.93,15.07,14.5,1

24/11/2009,04:52:07,24.3,5.7,5.7,1

24/11/2009,04:52:11,24.41,5.59,5.6,1

24/11/2009,04:53:50,24.3,5.7,5.7,1

24/11/2009,04:53:56,24.44,5.56,5.6,1

24/11/2009,04:53:58,24.51,5.49,5.5,1

24/11/2009,04:53:59,24.65,5.35,5.4,1

24/11/2009,04:56:02,27.17,2.83,2.8,1

24/11/2009,04:59:05,24.2,5.8,5.8,1

24/11/2009,04:59:29,26.05,3.95,4.0,2

24/11/2009,04:59:41,18.85,11.15,11.2,1

24/11/2009,05:02:25,16.16,13.84,13.8,1

24/11/2009,05:04:52,25.56,4.44,4.1,1

24/11/2009,05:04:51,25.91,4.09,4.4,1

24/11/2009,05:06:22,27.35,2.65,2.7,1

24/11/2009,05:11:39,24.55,5.45,5.6,1

24/11/2009,05:11:36,24.37,5.63,5.5,1

24/11/2009,05:16:27,12.8,17.2,17.4,1

24/11/2009,05:16:27,12.9,17.1,17.1,1

24/11/2009,05:16:26,12.59,17.41,17.2,2

24/11/2009,05:22:15,20.39,9.61,9.6,1

24/11/2009,05:22:13,20.42,9.58,9.6,1

24/11/2009,05:24:49,24.09,5.91,5.9,1

24/11/2009,05:26:05,20.49,9.51,9.4,1

24/11/2009,05:26:02,20.49,9.51,9.5,1

24/11/2009,05:26:00,20.56,9.44,9.5,1

24/11/2009,05:26:22,21.19,8.81,8.8,1

24/11/2009,05:28:32,19.62,10.38,6.2,1

24/11/2009,05:28:30,20,10,10.0,1

24/11/2009,05:26:39,23.81,6.19,10.4,1

24/11/2009,05:29:45,15.91,14.09,14.1,1

24/11/2009,05:29:43,15.88,14.12,14.1,1

24/11/2009,05:30:04,16.09,13.91,13.9,2

24/11/2009,05:34:11,22.69,7.31,7.3,1

24/11/2009,05:34:13,22.8,7.2,7.2,2

24/11/2009,05:35:00,24.23,5.77,5.8,1

24/11/2009,05:35:00,24.16,5.84,5.8,1

24/11/2009,05:35:01,24.06,5.94,5.9,1

24/11/2009,05:42:29,21.58,8.42,8.8,1

24/11/2009,05:42:18,21.16,8.84,8.4,1

24/11/2009,05:43:59,23.04,6.96,6.9,1

24/11/2009,05:43:58,23.08,6.92,7.0,1

24/11/2009,05:44:04,22.9,7.1,7.1,1

24/11/2009,05:44:07,22.76,7.24,7.2,1

24/11/2009,05:48:16,24.02,5.98,6.0,1

24/11/2009,05:48:17,23.57,6.43,6.4,1

24/11/2009,05:48:52,24.72,5.28,5.3,1

24/11/2009,05:50:23,25.53,4.47,4.5,1

24/11/2009,05:50:29,25.53,4.47,4.5,1

24/11/2009,05:56:16,20.7,9.3,9.8,1

24/11/2009,05:56:12,20.6,9.4,10.1,1

24/11/2009,05:56:03,19.9,10.1,9.4,1

24/11/2009,05:56:02,20.21,9.79,9.3,2

24/11/2009,05:56:49,24.3,5.7,5.7,1

24/11/2009,05:56:51,24.13,5.87,5.9,2

24/11/2009,05:57:02,24.44,5.56,5.6,2

24/11/2009,05:57:05,24.72,5.28,5.3,2

24/11/2009,05:57:08,25.04,4.96,5.0,1

24/11/2009,06:04:57,22.28,7.72,8.8,1

24/11/2009,06:04:53,22.14,7.86,7.9,2

24/11/2009,06:04:43,21.19,8.81,7.7,1

24/11/2009,06:08:22,25.46,4.54,4.5,1

24/11/2009,06:08:33,26.4,3.6,3.6,1

24/11/2009,06:11:08,21.89,8.11,8.1,2

24/11/2009,06:13:41,24.97,5.03,5.5,1

24/11/2009,06:13:41,24.69,5.31,5.3,1

24/11/2009,06:13:43,24.79,5.21,5.0,1

24/11/2009,06:11:51,24.69,5.31,5.3,1

24/11/2009,06:11:49,24.55,5.45,5.2,1

24/11/2009,06:17:03,25.39,4.61,4.6,1

24/11/2009,06:17:09,27.31,2.69,2.7,1

24/11/2009,06:23:00,19.44,10.56,10.2,1

24/11/2009,06:23:00,19.62,10.38,10.4,1

24/11/2009,06:23:00,19.79,10.21,10.6,2

24/11/2009,06:28:04,24.34,5.66,5.5,1

24/11/2009,06:28:04,24.48,5.52,5.7,2

24/11/2009,06:30:46,17.52,12.48,12.7,1

24/11/2009,06:30:44,17.34,12.66,12.5,1

24/11/2009,06:32:49,27.1,2.9,2.9,1

24/11/2009,06:35:49,26.33,3.67,3.6,1

24/11/2009,06:35:49,26.4,3.6,3.7,1

24/11/2009,06:38:56,26.86,3.14,3.1,1

24/11/2009,06:38:56,26.68,3.32,3.3,1

24/11/2009,06:42:53,23.18,6.82,6.8,1

24/11/2009,06:43:08,23.99,6.01,5.9,1

24/11/2009,06:43:06,24.58,5.42,4.9,1

24/11/2009,06:42:58,24.09,5.91,5.4,1

24/11/2009,06:42:59,25.14,4.86,6.0,1

24/11/2009,12:13:16,21.51,8.49,10.1,1

24/11/2009,12:13:15,21.4,8.6,8.5,1

24/11/2009,12:13:14,21.3,8.7,8.7,1

24/11/2009,12:13:14,21.47,8.53,8.6,1

24/11/2009,12:13:10,19.9,10.1,8.5,1

24/11/2009,14:57:20,20.88,9.12,9.1,1

24/11/2009,14:57:32,21.23,8.77,8.8,1

24/11/2009,14:57:41,20.88,9.12,9.1,1

24/11/2009,15:57:00,23.92,6.08,6.3,3

24/11/2009,15:56:56,23.74,6.26,6.1,1

24/11/2009,16:00:09,25.56,4.44,4.4,1

24/11/2009,16:00:13,25.67,4.33,4.3,1

24/11/2009,16:02:37,24.72,5.28,5.3,1

24/11/2009,16:02:44,24.97,5.03,5.0,1

24/11/2009,16:02:45,24.79,5.21,5.2,1

24/11/2009,16:14:48,25.67,4.33,4.3,1

24/11/2009,16:23:21,23.43,6.57,6.6,1

24/11/2009,16:23:29,25.11,4.89,4.9,1

24/11/2009,16:50:51,26.47,3.53,3.5,1

24/11/2009,17:27:51,25.03,4.97,5.0,1

24/11/2009,17:27:52,25.62,4.38,4.4,1

24/11/2009,17:46:47,23.15,6.85,6.9,1

24/11/2009,19:26:33,26.07,3.93,3.9,1

24/11/2009,19:26:34,25.87,4.13,4.1,1

24/11/2009,19:26:35,25.69,4.31,4.3,1

24/11/2009,19:26:36,25.69,4.31,4.3,1

24/11/2009,19:26:37,25.73,4.27,4.3,1

24/11/2009,19:52:07,23.4,6.6,6.6,1

24/11/2009,19:52:09,23.57,6.43,6.4,1

24/11/2009,20:12:54,23.68,6.32,6.3,1

24/11/2009,20:13:00,24.75,5.25,5.3,1

24/11/2009,20:13:01,25,5,5.0,1

24/11/2009,20:13:03,24.86,5.14,5.1,1

24/11/2009,20:13:04,24.93,5.07,5.1,1

24/11/2009,20:13:51,25.69,4.31,4.3,1

24/11/2009,20:13:53,25.83,4.17,4.2,1

24/11/2009,20:40:22,25.66,4.34,4.3,2

24/11/2009,20:40:25,25.76,4.24,4.2,1

24/11/2009,20:54:51,21.87,8.13,8.0,1

24/11/2009,20:54:50,22.04,7.96,8.0,2

24/11/2009,20:54:50,22.04,7.96,8.1,1

24/11/2009,20:54:56,22.32,7.68,7.7,1

24/11/2009,20:54:58,22.32,7.68,7.7,1

24/11/2009,20:54:59,22.39,7.61,7.6,2

24/11/2009,20:55:01,22.42,7.58,7.5,1

24/11/2009,20:55:01,22.46,7.54,7.6,1

24/11/2009,20:55:02,22.39,7.61,7.6,1

24/11/2009,20:55:19,23.61,6.39,6.4,1

24/11/2009,20:55:19,23.64,6.36,6.4,1

24/11/2009,20:55:21,23.75,6.25,6.3,1

24/11/2009,20:55:22,23.81,6.19,6.2,1

24/11/2009,20:55:23,23.92,6.08,6.1,1

24/11/2009,20:55:24,23.95,6.05,6.1,1

24/11/2009,20:55:26,24.06,5.94,5.9,1

24/11/2009,20:55:33,24.89,5.11,5.1,1

24/11/2009,21:43:41,21.62,8.38,8.4,1

24/11/2009,21:43:47,21.76,8.24,8.2,1

24/11/2009,21:43:49,21.94,8.06,8.1,1

24/11/2009,21:44:50,23.61,6.39,6.4,1

24/11/2009,22:21:40,23.36,6.64,6.7,1

24/11/2009,22:21:38,23.33,6.67,6.6,1

24/11/2009,22:21:57,24.34,5.66,5.7,1

24/11/2009,22:42:43,10.53,19.47,19.1,1

24/11/2009,22:42:42,10.92,19.08,19.5,1

24/11/2009,22:47:20,15.33,14.67,14.6,1

24/11/2009,22:47:15,15.4,14.6,14.7,1

24/11/2009,22:55:18,24.61,5.39,5.4,1

24/11/2009,22:55:27,24.68,5.32,5.3,1

24/11/2009,23:00:06,17.52,12.48,12.5,1

24/11/2009,23:00:08,17.52,12.48,12.5,1

24/11/2009,23:06:03,15.47,14.53,14.5,1

24/11/2009,23:06:06,15.64,14.36,14.4,1

24/11/2009,23:06:08,15.75,14.25,14.3,1

24/11/2009,23:06:10,15.71,14.29,14.3,1

24/11/2009,23:06:17,17.28,12.72,12.7,1

24/11/2009,23:06:20,17.52,12.48,12.5,1

24/11/2009,23:06:23,17.73,12.27,12.3,1

24/11/2009,23:14:09,20.3,9.7,9.7,1

24/11/2009,23:14:14,23.05,6.95,6.9,1

24/11/2009,23:14:12,23.15,6.85,7.0,2

24/11/2009,23:14:27,22.98,7.02,7.0,1

24/11/2009,23:14:32,23.19,6.81,6.8,1

24/11/2009,23:38:54,27.4,2.6,2.6,1

24/11/2009,23:38:55,27.36,2.64,2.6,1

25/11/2009,00:13:30,18.6,11.4,11.6,1

25/11/2009,00:13:27,18.36,11.64,11.4,1

25/11/2009,00:17:41,23.43,6.57,5.6,2

25/11/2009,00:17:41,24.37,5.63,6.6,1

25/11/2009,00:17:45,26.32,3.68,3.7,1

25/11/2009,00:31:03,23.19,6.81,4.7,1

25/11/2009,00:30:59,23.4,6.6,4.7,1

25/11/2009,00:30:57,24.27,5.73,5.7,1

25/11/2009,00:30:14,25.31,4.69,6.6,1

25/11/2009,00:30:12,25.31,4.69,6.8,1

25/11/2009,00:32:04,23.5,6.5,6.4,1

25/11/2009,00:32:01,23.36,6.64,6.6,1

25/11/2009,00:32:01,23.64,6.36,6.5,1

25/11/2009,00:38:16,21.24,8.76,8.6,1

25/11/2009,00:38:15,21.45,8.55,8.8,2

25/11/2009,00:38:29,22.01,7.99,8.0,1

25/11/2009,00:47:58,23.26,6.74,13.1,1

25/11/2009,00:48:00,23.78,6.22,10.6,1

25/11/2009,00:47:26,19.19,10.81,10.7,1

25/11/2009,00:47:23,19.3,10.7,10.8,1

25/11/2009,00:47:23,19.43,10.57,6.7,1

25/11/2009,00:47:14,16.86,13.14,6.2,1

25/11/2009,00:49:46,-1.98,31.98,32.0,1

25/11/2009,00:56:28,24.16,5.84,5.8,1

25/11/2009,00:59:10,20.82,9.18,9.2,1

25/11/2009,00:59:11,21.03,8.97,9.0,3

25/11/2009,00:59:20,26.46,3.54,3.4,1

25/11/2009,00:59:19,26.6,3.4,3.5,1

25/11/2009,01:05:00,19.36,10.64,14.3,1

25/11/2009,01:04:50,19.09,10.91,12.3,1

25/11/2009,01:04:50,19.02,10.98,11.6,1

25/11/2009,01:05:01,14.36,15.64,10.9,1

25/11/2009,01:04:59,14.15,15.85,11.0,1

25/11/2009,01:03:20,17.66,12.34,15.9,2

25/11/2009,01:03:21,18.43,11.57,10.6,1

25/11/2009,01:01:32,15.75,14.25,15.6,1

25/11/2009,01:06:31,24.54,5.46,5.5,1

25/11/2009,01:06:32,24.61,5.39,5.4,1

25/11/2009,01:06:36,24.48,5.52,5.5,1

25/11/2009,01:06:39,24.93,5.07,5.1,1

25/11/2009,01:06:41,24.96,5.04,4.8,1

25/11/2009,01:06:40,25.24,4.76,5.0,1

25/11/2009,01:08:46,6.88,23.12,23.4,1

25/11/2009,01:08:41,6.64,23.36,23.4,2

25/11/2009,01:08:37,6.61,23.39,23.1,1

25/11/2009,01:11:29,26.28,3.72,3.7,1

25/11/2009,01:11:31,26.49,3.51,3.5,1

25/11/2009,01:17:26,19.61,10.39,10.0,1

25/11/2009,01:17:25,20.03,9.97,10.4,1

25/11/2009,01:24:35,23.43,6.57,6.5,1

25/11/2009,01:24:35,23.5,6.5,6.6,1

25/11/2009,01:26:38,23.36,6.64,6.6,1

25/11/2009,01:26:40,23.4,6.6,6.6,1

25/11/2009,01:28:31,26.91,3.09,3.1,1

25/11/2009,01:28:33,26.98,3.02,3.0,1

25/11/2009,01:28:38,27.19,2.81,2.8,1

25/11/2009,01:32:46,24.09,5.91,5.9,1

25/11/2009,01:32:55,26.91,3.09,3.1,1

25/11/2009,01:55:44,22.67,7.33,7.3,1

25/11/2009,01:55:40,23.12,6.88,7.0,1

25/11/2009,01:55:39,22.98,7.02,6.9,1

25/11/2009,01:55:37,22.74,7.26,7.3,1

25/11/2009,01:56:18,25.21,4.79,4.9,1

25/11/2009,01:56:16,25.14,4.86,4.8,1

25/11/2009,02:12:06,21.24,8.76,9.0,1

25/11/2009,02:12:01,21,9,8.8,1

25/11/2009,02:14:58,21.87,8.13,8.4,1

25/11/2009,02:14:58,21.9,8.1,8.2,1

25/11/2009,02:14:53,21.76,8.24,8.1,1

25/11/2009,02:14:48,21.62,8.38,8.1,1

25/11/2009,02:17:20,20.93,9.07,9.2,1

25/11/2009,02:17:18,20.79,9.21,9.1,1

25/11/2009,02:18:51,25.24,4.76,4.9,1

25/11/2009,02:18:46,25.27,4.73,4.7,1

25/11/2009,02:18:42,25.1,4.9,4.8,1

25/11/2009,02:20:33,25.76,4.24,4.1,1

25/11/2009,02:20:32,25.87,4.13,4.2,2

25/11/2009,02:20:54,26.63,3.37,3.4,1

25/11/2009,02:20:51,26.63,3.37,3.4,1

25/11/2009,02:30:25,20.16,9.84,9.6,1

25/11/2009,02:30:22,20.44,9.56,9.8,2

25/11/2009,02:37:08,16.1,13.9,14.4,1

25/11/2009,02:37:04,15.64,14.36,13.9,1

25/11/2009,02:50:49,18.43,11.57,11.6,1

25/11/2009,02:51:00,23.47,6.53,6.5,1

25/11/2009,02:53:02,26.63,3.37,3.1,1

25/11/2009,02:53:00,26.87,3.13,4.5,1

25/11/2009,02:52:58,26.87,3.13,3.1,1

25/11/2009,02:53:01,25.59,4.41,4.5,1

25/11/2009,02:53:01,25.55,4.45,4.4,1

25/11/2009,02:53:00,25.52,4.48,4.5,1

25/11/2009,02:52:59,25.52,4.48,3.4,1

25/11/2009,03:07:09,24.37,5.63,5.6,1

25/11/2009,03:07:15,24.37,5.63,5.6,1

25/11/2009,03:07:17,24.37,5.63,5.6,1

25/11/2009,03:07:19,24.3,5.7,5.7,1

25/11/2009,03:09:22,17.9,12.1,12.1,1

25/11/2009,03:22:29,24.54,5.46,5.4,1

25/11/2009,03:22:29,24.58,5.42,5.4,2

25/11/2009,03:22:28,24.51,5.49,5.5,1

25/11/2009,03:21:56,24.65,5.35,5.4,1

25/11/2009,03:21:54,24.61,5.39,5.5,1

25/11/2009,03:22:39,25.14,4.86,4.9,1

25/11/2009,03:22:37,25.14,4.86,4.9,1

25/11/2009,03:54:34,22.29,7.71,7.7,1

25/11/2009,03:55:18,22.39,7.61,7.6,1

25/11/2009,04:14:25,26.84,3.16,3.2,1

25/11/2009,04:14:34,26.84,3.16,3.2,1

25/11/2009,04:29:27,26.84,3.16,2.5,1

25/11/2009,04:29:25,26.91,3.09,2.6,1

25/11/2009,04:29:24,27.05,2.95,2.9,1

25/11/2009,04:29:23,27.12,2.88,3.0,4

25/11/2009,04:29:22,27.43,2.57,3.1,2

25/11/2009,04:29:22,27.5,2.5,3.2,1

25/11/2009,04:30:57,27.12,2.88,2.9,1

25/11/2009,04:31:00,27.26,2.74,2.7,1

25/11/2009,04:31:02,27.29,2.71,2.7,1

25/11/2009,05:21:33,23.08,6.92,7.0,1

25/11/2009,05:21:28,23.05,6.95,6.9,1

25/11/2009,05:36:53,25.59,4.41,4.6,1

25/11/2009,05:36:51,25.38,4.62,4.4,1

25/11/2009,05:48:30,23.75,6.25,6.3,1

25/11/2009,05:50:36,25.45,4.55,4.6,1

25/11/2009,05:50:44,26.11,3.89,3.9,1

25/11/2009,06:39:18,23.15,6.85,7.0,1

25/11/2009,06:39:13,23.05,6.95,6.9,1

25/11/2009,06:56:59,27.67,2.33,2.2,1

25/11/2009,06:56:59,27.81,2.19,2.3,1

25/11/2009,06:57:05,27.99,2.01,2.0,1

25/11/2009,06:57:06,27.92,2.08,2.1,1

25/11/2009,15:12:02,23.82,6.18,5.8,1

25/11/2009,15:12:02,24.04,5.96,6.1,3

25/11/2009,15:12:02,24.39,5.61,5.6,2

25/11/2009,15:12:02,24.61,5.39,5.5,1

25/11/2009,15:12:02,24.71,5.29,5.4,1

25/11/2009,15:12:00,24.71,5.29,5.3,1

25/11/2009,15:11:59,24.64,5.36,5.3,1

25/11/2009,15:11:58,24.54,5.46,5.4,1

25/11/2009,15:11:56,24.39,5.61,5.6,1

25/11/2009,15:11:55,24.25,5.75,6.0,1

25/11/2009,15:11:55,23.93,6.07,6.2,1

25/11/2009,15:12:05,25.25,4.75,4.6,1

25/11/2009,15:12:04,25.42,4.58,4.8,1

25/11/2009,15:44:23,21.94,8.06,8.1,1

25/11/2009,15:44:26,21.87,8.13,8.1,1

25/11/2009,16:16:22,23.81,6.19,6.2,1

25/11/2009,16:16:23,23.81,6.19,6.2,1

25/11/2009,17:41:23,25.55,4.45,4.6,1

25/11/2009,17:41:21,25.47,4.53,4.5,1

25/11/2009,17:41:20,25.45,4.55,4.5,1

25/11/2009,17:41:30,25.5,4.5,4.5,1

25/11/2009,17:41:31,25.57,4.43,4.4,1

25/11/2009,17:41:33,25.52,4.48,4.5,1

25/11/2009,19:09:13,22.86,7.14,7.2,1

25/11/2009,19:09:12,22.88,7.12,7.3,1

25/11/2009,19:09:09,22.74,7.26,7.1,1

25/11/2009,19:09:03,22.79,7.21,7.1,1

25/11/2009,19:21:23,20.75,9.25,9.4,1

25/11/2009,19:21:22,20.87,9.13,9.1,1

25/11/2009,19:21:19,20.58,9.42,9.3,1

25/11/2009,20:16:31,20.46,9.54,9.8,1

25/11/2009,20:16:28,20.15,9.85,10.3,1

25/11/2009,20:16:16,19.83,10.17,10.2,1

25/11/2009,20:16:12,19.71,10.29,9.9,1

25/11/2009,20:16:12,20.24,9.76,9.5,1

25/11/2009,20:25:50,19.08,10.92,9.6,1

25/11/2009,20:25:48,18.91,11.09,8.6,1

25/11/2009,20:25:47,20.37,9.63,11.1,1

25/11/2009,20:25:48,21.38,8.62,10.9,1

25/11/2009,21:14:48,10.9,19.1,19.3,1

25/11/2009,21:14:46,10.66,19.34,19.1,1

25/11/2009,21:40:33,22.54,7.46,8.1,1

25/11/2009,21:40:29,22.16,7.84,8.3,1

25/11/2009,21:38:57,21.72,8.28,8.1,1

25/11/2009,21:38:57,21.89,8.11,7.8,1

25/11/2009,21:40:31,22.13,7.87,7.9,1

25/11/2009,21:40:28,21.92,8.08,7.5,1

25/11/2009,22:00:26,14.17,15.83,15.7,1

25/11/2009,22:00:24,14.34,15.66,15.8,1

25/11/2009,22:21:09,21.53,8.47,8.5,1

25/11/2009,22:21:11,21.55,8.45,8.5,1

25/11/2009,22:25:46,16.69,13.31,13.3,1

25/11/2009,22:25:46,16.71,13.29,13.3,1

25/11/2009,22:58:15,18.7,11.3,11.3,1

25/11/2009,22:58:12,18.66,11.34,11.3,1

25/11/2009,23:14:28,17.69,12.31,12.6,1

25/11/2009,23:14:24,17.45,12.55,12.3,1

25/11/2009,23:33:44,18.83,11.17,11.0,1

25/11/2009,23:33:43,18.97,11.03,11.2,1

25/11/2009,23:33:45,19.34,10.66,10.5,2

25/11/2009,23:33:45,19.47,10.53,10.7,1

25/11/2009,23:34:58,19.44,10.56,9.9,1

25/11/2009,23:34:57,19.74,10.26,10.3,1

25/11/2009,23:34:56,20.08,9.92,10.6,1

25/11/2009,23:35:26,22.3,7.7,7.7,1

26/11/2009,00:11:51,18.87,11.13,11.4,1

26/11/2009,00:11:50,18.63,11.37,11.1,3

26/11/2009,00:11:57,18.76,11.24,11.2,1

26/11/2009,00:33:38,16.24,13.76,13.9,1

26/11/2009,00:33:36,16.1,13.9,13.8,1

26/11/2009,00:33:47,23.18,6.82,7.2,1

26/11/2009,00:33:42,22.84,7.16,7.2,1

26/11/2009,00:33:42,22.81,7.19,6.8,1

26/11/2009,00:40:31,18.29,11.71,11.8,2

26/11/2009,00:40:29,18.19,11.81,11.7,1

26/11/2009,00:46:47,24.52,5.48,5.3,1

26/11/2009,00:46:42,24.66,5.34,5.5,1

26/11/2009,00:53:45,19.74,10.26,11.1,1

26/11/2009,00:53:42,18.93,11.07,10.3,1

26/11/2009,01:14:59,22.74,7.26,7.3,1

26/11/2009,01:14:59,22.57,7.43,7.4,1

26/11/2009,01:29:42,22.57,7.43,7.7,1

26/11/2009,01:29:38,22.33,7.67,7.4,1

26/11/2009,01:37:44,9.16,20.84,20.7,1

26/11/2009,01:37:44,9.27,20.73,20.8,1

26/11/2009,01:43:35,18.46,11.54,6.6,1

26/11/2009,01:43:02,23.48,6.52,6.7,1

26/11/2009,01:43:09,24.05,5.95,6.5,2

26/11/2009,01:42:58,23.31,6.69,6.0,1

26/11/2009,01:42:55,23.38,6.62,11.5,1

26/11/2009,01:52:46,18.6,11.4,11.4,1

26/11/2009,01:52:43,18.6,11.4,11.4,1

26/11/2009,02:15:13,17.05,12.95,12.9,1

26/11/2009,02:15:12,17.08,12.92,13.0,1

26/11/2009,02:16:38,19.17,10.83,10.8,1

26/11/2009,02:16:43,19.94,10.06,10.1,1

26/11/2009,02:29:30,13.85,16.15,15.9,1

26/11/2009,02:29:28,13.85,16.15,16.3,1

26/11/2009,02:29:26,13.75,16.25,16.2,1

26/11/2009,02:29:26,14.12,15.88,16.2,1

26/11/2009,02:32:54,19.74,10.26,10.8,1

26/11/2009,02:32:52,19.17,10.83,10.3,1

26/11/2009,02:33:08,20.48,9.52,9.5,3

26/11/2009,02:36:17,20.28,9.72,10.0,1

26/11/2009,02:36:11,20.01,9.99,9.7,1

26/11/2009,02:36:26,17.42,12.58,12.6,1

26/11/2009,02:36:27,17.59,12.41,12.4,1

26/11/2009,02:43:24,12.87,17.13,17.3,2

26/11/2009,02:43:21,13.27,16.73,16.7,1

26/11/2009,02:43:21,12.3,17.7,17.7,1

26/11/2009,02:43:19,12.73,17.27,17.1,1

26/11/2009,02:46:26,20.72,9.28,10.3,1

26/11/2009,02:46:23,20.08,9.92,9.9,1

26/11/2009,02:46:20,19.67,10.33,10.4,1

26/11/2009,02:46:25,19.57,10.43,9.3,1

26/11/2009,02:46:30,19.54,10.46,10.5,1

26/11/2009,03:00:20,19.44,10.56,10.1,1

26/11/2009,03:00:16,19.34,10.66,10.7,1

26/11/2009,03:00:04,19.94,10.06,10.6,1

26/11/2009,03:24:18,23.58,6.42,7.2,1

26/11/2009,03:24:16,23.48,6.52,7.2,1

26/11/2009,03:24:14,23.38,6.62,7.2,1

26/11/2009,03:24:12,23.21,6.79,7.2,1

26/11/2009,03:24:09,23.21,6.79,7.0,1

26/11/2009,03:24:08,23.04,6.96,6.8,1

26/11/2009,03:24:07,22.84,7.16,6.8,1

26/11/2009,03:24:04,22.84,7.16,6.6,1

26/11/2009,03:24:03,22.84,7.16,6.5,1

26/11/2009,03:24:03,22.81,7.19,6.4,1

26/11/2009,04:03:21,19.74,10.26,10.3,1

26/11/2009,04:03:23,19.71,10.29,10.3,1

26/11/2009,04:03:38,23.41,6.59,6.3,1

26/11/2009,04:03:36,23.68,6.32,6.6,2

26/11/2009,04:14:59,19.88,10.12,10.1,1

26/11/2009,04:14:59,19.81,10.19,10.2,1

26/11/2009,04:17:12,19.88,10.12,10.1,1

26/11/2009,04:25:41,17.08,12.92,12.5,1

26/11/2009,04:25:41,17.55,12.45,12.9,2

26/11/2009,04:26:18,18.46,11.54,11.5,1

26/11/2009,04:41:00,25.37,4.63,4.6,1

26/11/2009,05:09:16,20.82,9.18,9.2,2

26/11/2009,05:09:17,21.59,8.41,8.4,1

26/11/2009,05:12:46,25.03,4.97,5.0,1

26/11/2009,05:35:18,21.59,8.41,8.5,1

26/11/2009,05:35:17,21.49,8.51,8.4,1

26/11/2009,05:38:32,18.02,11.98,12.5,1

26/11/2009,05:38:28,17.55,12.45,12.0,1

26/11/2009,05:41:34,22.94,7.06,7.4,2

26/11/2009,05:41:32,22.87,7.13,7.1,1

26/11/2009,05:41:31,22.64,7.36,7.1,1

26/11/2009,05:45:35,17.59,12.41,12.4,1

26/11/2009,06:00:47,23.21,6.79,6.7,1

26/11/2009,06:00:46,23.31,6.69,6.8,1

26/11/2009,06:36:13,19,11,11.3,1

26/11/2009,06:36:09,18.7,11.3,12.1,1

26/11/2009,06:36:13,17.92,12.08,11.0,1

26/11/2009,06:36:11,17.89,12.11,12.1,1

26/11/2009,11:24:39,19.4,10.6,8.8,1

26/11/2009,11:24:33,20.68,9.32,9.2,2

26/11/2009,11:24:32,20.75,9.25,9.3,1

26/11/2009,11:24:32,20.89,9.11,9.1,1

26/11/2009,11:24:31,20.72,9.28,9.3,1

26/11/2009,11:24:31,20.85,9.15,7.9,1

26/11/2009,11:24:30,21.22,8.78,9.3,1

26/11/2009,11:24:33,22.1,7.9,10.6,1

26/11/2009,11:57:48,22,8,8.8,1

26/11/2009,11:57:48,22.07,7.93,8.4,1

26/11/2009,11:57:47,22,8,8.4,1

26/11/2009,11:57:45,21.8,8.2,8.3,1

26/11/2009,11:57:44,21.66,8.34,8.2,2

26/11/2009,11:57:42,21.59,8.41,8.0,1

26/11/2009,11:57:42,21.59,8.41,7.9,1

26/11/2009,11:57:40,21.22,8.78,8.0,2

26/11/2009,15:59:00,23.92,6.08,6.1,1

26/11/2009,15:59:03,24.22,5.78,5.8,1

26/11/2009,16:15:30,24.02,5.98,5.8,1

26/11/2009,16:15:29,24.19,5.81,5.8,1

26/11/2009,16:15:26,24.22,5.78,6.0,1

26/11/2009,16:16:23,22.84,7.16,7.3,1

26/11/2009,16:16:21,22.81,7.19,7.2,1

26/11/2009,16:16:20,22.74,7.26,7.2,1

26/11/2009,21:21:07,15.16,14.84,14.5,1

26/11/2009,21:21:07,15.27,14.73,14.7,1

26/11/2009,21:21:06,15.48,14.52,14.8,1

26/11/2009,21:21:16,18.32,11.68,11.7,1

26/11/2009,21:54:00,24.22,5.78,5.5,1

26/11/2009,21:53:58,24.46,5.54,5.6,2

26/11/2009,21:53:58,24.36,5.64,5.8,1

26/11/2009,22:11:46,19.71,10.29,10.1,1

26/11/2009,22:11:45,19.92,10.08,10.3,1

26/11/2009,22:49:01,6.18,23.82,23.8,1

26/11/2009,22:56:23,24.07,5.93,5.9,1

26/11/2009,23:22:22,17.72,12.28,12.3,1

26/11/2009,23:30:36,6.82,23.18,5.7,1

26/11/2009,23:30:33,6.57,23.43,23.4,1

26/11/2009,23:31:13,12.18,17.82,23.2,1

26/11/2009,23:31:12,12.18,17.82,17.9,1

26/11/2009,23:31:11,12.21,17.79,17.8,2

26/11/2009,23:31:09,12.11,17.89,17.8,1

26/11/2009,23:34:32,15.98,14.02,17.8,1

26/11/2009,23:34:23,15.84,14.16,14.2,1

26/11/2009,23:29:49,24.29,5.71,14.0,1

26/11/2009,23:38:31,14.7,15.3,15.3,1

26/11/2009,23:56:59,21.38,8.62,7.8,2

26/11/2009,23:56:59,21.66,8.34,7.8,1

26/11/2009,23:56:59,21.94,8.06,7.7,1

26/11/2009,23:56:58,22.12,7.88,7.9,1

26/11/2009,23:56:56,22.3,7.7,8.1,1

26/11/2009,23:56:53,22.23,7.77,8.3,1

26/11/2009,23:56:51,22.16,7.84,8.6,1

27/11/2009,00:43:30,8.95,21.05,21.1,1

27/11/2009,00:48:21,24.61,5.39,5.0,1

27/11/2009,00:48:19,24.39,5.61,5.0,1

27/11/2009,00:48:01,25.14,4.86,5.0,1

27/11/2009,00:47:57,25,5,4.9,2

27/11/2009,00:47:52,24.96,5.04,5.6,2

27/11/2009,00:47:51,25,5,5.4,1

27/11/2009,01:03:33,22.97,7.03,7.0,1

27/11/2009,01:06:26,25.25,4.75,4.8,1

27/11/2009,01:06:29,25.25,4.75,4.8,1

27/11/2009,01:28:01,21.2,8.8,8.7,1

27/11/2009,01:27:59,21.3,8.7,8.8,1

27/11/2009,01:33:44,23.86,6.14,6.1,1

27/11/2009,01:36:24,20.91,9.09,9.0,1

27/11/2009,01:36:19,20.7,9.3,9.3,1

27/11/2009,01:36:18,20.98,9.02,9.1,1

27/11/2009,01:46:38,15.37,14.63,14.6,1

27/11/2009,01:56:02,16.33,13.67,13.6,1

27/11/2009,01:55:51,16.44,13.56,13.6,1

27/11/2009,01:55:50,16.44,13.56,13.7,1

27/11/2009,02:01:46,20.06,9.94,5.9,1

27/11/2009,02:01:40,19.71,10.29,10.3,1

27/11/2009,02:00:31,24.07,5.93,9.9,2

27/11/2009,02:05:13,24.46,5.54,15.3,1

27/11/2009,02:05:15,24.46,5.54,15.1,1

27/11/2009,02:05:17,24.5,5.5,5.5,1

27/11/2009,02:05:17,23.4,6.6,5.5,1

27/11/2009,02:04:25,14.91,15.09,5.5,1

27/11/2009,02:04:24,14.7,15.3,6.6,1

27/11/2009,02:13:41,20.13,9.87,9.7,1

27/11/2009,02:13:39,20.31,9.69,9.9,2

27/11/2009,02:16:56,22.26,7.74,7.7,1

27/11/2009,02:16:55,22.3,7.7,7.7,1

27/11/2009,02:16:55,22.26,7.74,7.7,1

27/11/2009,02:19:46,23.68,6.32,20.1,1

27/11/2009,02:19:43,23.68,6.32,19.9,1

27/11/2009,02:19:47,24,6,12.5,1

27/11/2009,02:19:48,24.43,5.57,12.7,2

27/11/2009,02:22:09,23.26,6.74,20.7,1

27/11/2009,02:19:42,9.41,20.59,20.6,1

27/11/2009,02:19:41,9.27,20.73,6.3,1

27/11/2009,02:17:48,9.91,20.09,6.3,1

27/11/2009,02:17:53,10.08,19.92,6.0,1

27/11/2009,02:18:32,17.26,12.74,5.6,1

27/11/2009,02:18:32,17.5,12.5,6.7,1

27/11/2009,02:25:51,23.86,6.14,6.3,1

27/11/2009,02:25:49,23.75,6.25,6.1,3

27/11/2009,02:32:41,24.39,5.61,5.6,1

27/11/2009,02:32:44,24.43,5.57,5.6,1

27/11/2009,02:32:46,24.68,5.32,5.3,1

27/11/2009,02:34:49,15.13,14.87,15.0,1

27/11/2009,02:34:47,15.05,14.95,14.9,1

27/11/2009,02:35:18,23.43,6.57,6.6,1

27/11/2009,02:40:45,20.49,9.51,9.7,2

27/11/2009,02:40:43,20.31,9.69,9.5,1

27/11/2009,02:41:46,18.04,11.96,12.0,1

27/11/2009,02:44:18,23.68,6.32,6.3,1

27/11/2009,02:44:31,23.75,6.25,6.6,2

27/11/2009,02:44:30,23.43,6.57,6.3,1

27/11/2009,02:50:26,15.34,14.66,14.7,1

27/11/2009,02:50:28,15.34,14.66,14.7,1

27/11/2009,02:53:05,20.38,9.62,9.6,1

27/11/2009,02:54:19,17.43,12.57,12.6,1

27/11/2009,03:01:46,23.15,6.85,6.9,2

27/11/2009,03:02:43,23.33,6.67,6.7,1

27/11/2009,03:02:47,23.19,6.81,6.8,1

27/11/2009,03:14:08,22.05,7.95,8.0,1

27/11/2009,03:14:36,23.61,6.39,6.4,1

27/11/2009,03:14:38,23.68,6.32,6.3,1

27/11/2009,03:26:22,18.14,11.86,11.9,1

27/11/2009,03:29:16,23.22,6.78,6.8,1

27/11/2009,03:31:00,24.07,5.93,5.9,1

27/11/2009,03:31:01,23.86,6.14,6.1,1

27/11/2009,03:31:14,25,5,5.0,1

27/11/2009,03:31:14,24.82,5.18,5.2,1

27/11/2009,03:32:34,19.43,10.57,10.8,1

27/11/2009,03:32:32,19.22,10.78,10.6,1

27/11/2009,03:33:42,23.71,6.29,6.3,3

27/11/2009,03:43:48,16.76,13.24,13.5,1

27/11/2009,03:43:45,16.52,13.48,13.2,1

27/11/2009,04:02:22,23.88,6.12,6.1,1

27/11/2009,04:02:35,21.29,8.71,8.7,1

27/11/2009,04:02:35,21.29,8.71,8.7,1

27/11/2009,04:11:39,12.41,17.59,17.6,1

27/11/2009,04:11:56,12.37,17.63,17.6,1

27/11/2009,04:20:49,21.85,8.15,8.3,2

27/11/2009,04:20:47,21.74,8.26,8.2,1

27/11/2009,04:21:05,18.22,11.78,11.8,1

27/11/2009,04:21:08,18.22,11.78,12.0,1

27/11/2009,04:21:07,17.97,12.03,11.8,1

27/11/2009,04:24:54,22.5,7.5,7.5,2

27/11/2009,04:39:27,24.06,5.94,5.9,1

27/11/2009,04:39:28,24.06,5.94,5.9,1

27/11/2009,04:39:31,24.2,5.8,5.8,3

27/11/2009,04:58:39,23.95,6.05,6.1,1

27/11/2009,04:58:39,23.85,6.15,6.2,1

27/11/2009,05:03:35,23.57,6.43,6.4,1

27/11/2009,05:03:38,23.95,6.05,6.1,1

27/11/2009,05:10:04,22.16,7.84,7.8,1

27/11/2009,05:22:37,22.81,7.19,6.9,1

27/11/2009,05:22:36,23.06,6.94,7.2,1

27/11/2009,05:23:10,22.81,7.19,7.2,1

27/11/2009,05:23:11,22.71,7.29,7.3,1

27/11/2009,05:27:59,23.02,6.98,6.7,1

27/11/2009,05:27:59,23.12,6.88,6.8,1

27/11/2009,05:27:59,23.23,6.77,6.4,1

27/11/2009,05:27:58,23.57,6.43,6.8,1

27/11/2009,05:27:52,23.23,6.77,6.9,1

27/11/2009,05:27:46,23.3,6.7,7.0,2

27/11/2009,05:29:29,21.91,8.09,8.1,1

27/11/2009,05:29:38,21.15,8.85,8.9,1

27/11/2009,05:29:39,21.19,8.81,8.8,1

27/11/2009,05:29:51,23.37,6.63,6.6,1

27/11/2009,05:29:52,23.57,6.43,6.4,1

27/11/2009,05:29:57,23.4,6.6,6.6,1

27/11/2009,05:29:59,23.47,6.53,6.5,1

27/11/2009,05:30:01,23.44,6.56,6.6,1

27/11/2009,05:30:01,23.26,6.74,6.7,1

27/11/2009,05:35:57,22.12,7.88,8.3,2

27/11/2009,05:35:54,21.95,8.05,8.3,1

27/11/2009,05:35:47,21.71,8.29,8.1,1

27/11/2009,05:35:46,21.71,8.29,7.9,1

27/11/2009,05:36:26,21.91,8.09,8.3,1

27/11/2009,05:36:24,21.74,8.26,8.1,1

27/11/2009,06:03:14,22.78,7.22,7.4,1

27/11/2009,06:03:13,22.57,7.43,7.2,1

27/11/2009,06:15:29,25.54,4.46,5.1,1

27/11/2009,06:15:24,25.23,4.77,4.8,1

27/11/2009,06:15:24,25.13,4.87,4.9,1

27/11/2009,06:15:23,24.89,5.11,4.5,1

27/11/2009,06:33:31,24.2,5.8,5.8,1

27/11/2009,06:37:48,24.23,5.77,5.8,1

27/11/2009,06:37:46,24.06,5.94,5.8,1

27/11/2009,06:37:43,24.16,5.84,5.9,1

27/11/2009,06:37:42,24.16,5.84,5.8,1

27/11/2009,06:42:11,21.29,8.71,8.9,1

27/11/2009,06:42:09,21.22,8.78,8.8,1

27/11/2009,06:42:08,21.09,8.91,8.7,1

27/11/2009,06:42:29,22.29,7.71,7.7,1

27/11/2009,06:42:31,22.26,7.74,7.7,1

27/11/2009,06:42:32,22.26,7.74,7.7,1

27/11/2009,06:42:34,22.29,7.71,7.7,1

27/11/2009,06:42:37,22.4,7.6,7.6,1

27/11/2009,06:42:39,22.47,7.53,7.5,1

27/11/2009,06:42:40,22.68,7.32,7.3,2

27/11/2009,07:14:16,21.12,8.88,9.2,1

27/11/2009,07:14:14,21.02,8.98,9.0,1

27/11/2009,07:14:11,20.77,9.23,8.9,1

27/11/2009,07:14:28,21.78,8.22,8.2,1

27/11/2009,07:14:27,21.78,8.22,8.2,1

27/11/2009,15:17:22,23.37,6.63,6.6,1

27/11/2009,15:38:04,22.54,7.46,7.5,1

27/11/2009,15:38:06,22.85,7.15,7.2,1

27/11/2009,15:38:13,22.68,7.32,7.3,1

27/11/2009,15:38:14,22.81,7.19,7.2,1

27/11/2009,16:22:15,22.99,7.01,6.7,1

27/11/2009,16:22:12,22.74,7.26,6.8,1

27/11/2009,16:22:10,22.5,7.5,7.5,1

27/11/2009,16:22:00,23.16,6.84,7.3,1

27/11/2009,16:21:59,23.33,6.67,7.0,1

27/11/2009,16:27:28,25.68,4.32,6.9,1

27/11/2009,16:27:28,25.68,4.32,4.3,1

27/11/2009,16:27:22,23.12,6.88,4.3,2

27/11/2009,16:30:00,25.58,4.42,4.4,2

27/11/2009,16:36:00,24.68,5.32,5.4,1

27/11/2009,16:35:58,24.65,5.35,5.3,1

27/11/2009,16:37:09,20.95,9.05,9.1,1

27/11/2009,16:37:09,20.91,9.09,9.1,1

27/11/2009,16:43:05,24.96,5.04,5.0,1

27/11/2009,18:06:07,24.75,5.25,5.3,1

27/11/2009,18:44:07,23.44,6.56,6.5,1

27/11/2009,18:44:02,23.61,6.39,6.4,1

27/11/2009,18:44:01,23.47,6.53,6.6,1

27/11/2009,19:04:52,16.76,13.24,13.2,1

27/11/2009,19:04:49,16.8,13.2,12.7,1

27/11/2009,19:04:50,17.32,12.68,13.2,1

27/11/2009,19:09:53,24.96,5.04,5.0,1

27/11/2009,19:18:18,23.92,6.08,5.9,1

27/11/2009,19:18:17,24.13,5.87,6.1,1

27/11/2009,19:31:55,26.2,3.8,4.0,3

27/11/2009,19:31:52,25.96,4.04,3.8,1

27/11/2009,19:52:27,19.15,10.85,10.8,1

27/11/2009,19:52:23,19.18,10.82,10.9,2

27/11/2009,19:53:34,25.06,4.94,5.1,3

27/11/2009,19:53:32,24.92,5.08,4.9,1

27/11/2009,21:47:00,11.82,18.18,18.2,1

27/11/2009,21:47:00,11.65,18.35,18.4,1

27/11/2009,21:47:01,11.3,18.7,18.7,1

27/11/2009,21:48:59,16.38,13.62,13.6,1

27/11/2009,21:49:03,16.63,13.37,13.4,1

27/11/2009,21:50:06,25.68,4.32,4.3,1

27/11/2009,21:51:49,17.59,12.41,12.4,1

27/11/2009,21:51:50,17.73,12.27,12.3,1

27/11/2009,22:20:35,26.54,3.46,3.5,1

27/11/2009,22:28:34,23.99,6.01,6.2,1

27/11/2009,22:28:32,23.85,6.15,6.0,1

27/11/2009,22:33:37,26.06,3.94,3.9,1

27/11/2009,22:50:45,17.94,12.06,11.8,1

27/11/2009,22:50:45,18.04,11.96,11.0,1

27/11/2009,22:50:45,18.18,11.82,11.7,1

27/11/2009,22:50:45,18.35,11.65,11.8,1

27/11/2009,22:50:40,19.04,10.96,12.0,1

27/11/2009,22:50:37,18.25,11.75,12.1,1

27/11/2009,22:55:11,24.95,5.05,4.9,3

27/11/2009,22:55:11,25.13,4.87,5.1,1

27/11/2009,22:55:14,25.33,4.67,4.7,1

27/11/2009,23:11:12,26.85,3.15,3.0,2

27/11/2009,23:11:08,26.99,3.01,3.2,1

27/11/2009,23:51:44,9.09,20.91,20.7,1

27/11/2009,23:51:43,9.26,20.74,20.9,1

27/11/2009,23:58:09,20.08,9.92,9.9,1

27/11/2009,23:58:13,19.08,10.92,10.9,1

28/11/2009,00:15:54,26.34,3.66,3.8,1

28/11/2009,00:15:52,26.27,3.73,3.7,1

28/11/2009,00:15:49,26.16,3.84,3.7,2

28/11/2009,00:22:27,25.58,4.42,5.2,1

28/11/2009,00:22:24,25.44,4.56,5.0,1

28/11/2009,00:22:23,25.51,4.49,6.5,1

28/11/2009,00:22:22,25.54,4.46,4.5,1

28/11/2009,00:22:20,25.47,4.53,4.5,1

28/11/2009,00:22:10,25.02,4.98,4.5,1

28/11/2009,00:22:06,24.78,5.22,4.6,1

28/11/2009,00:22:42,26.06,3.94,4.4,1

28/11/2009,00:22:41,26.3,3.7,3.6,1

28/11/2009,00:22:41,26.41,3.59,3.6,1

28/11/2009,00:22:41,26.44,3.56,3.7,1

28/11/2009,00:22:42,25.85,4.15,3.9,1

28/11/2009,00:22:44,25.44,4.56,4.2,2

28/11/2009,00:22:15,23.5,6.5,4.6,1

28/11/2009,00:23:07,23.85,6.15,6.2,1

28/11/2009,00:23:10,24.16,5.84,5.8,1

28/11/2009,00:23:14,24.3,5.7,5.7,1

28/11/2009,00:23:22,24.75,5.25,5.3,1

28/11/2009,00:23:24,24.82,5.18,5.2,1

28/11/2009,00:23:46,25.68,4.32,4.3,1

28/11/2009,00:23:47,25.54,4.46,4.5,1

28/11/2009,00:23:49,25.64,4.36,4.4,1

28/11/2009,00:24:56,26.27,3.73,3.7,1

28/11/2009,00:24:58,26.47,3.53,3.5,1

28/11/2009,00:36:37,26.2,3.8,3.8,1

28/11/2009,00:36:46,26.13,3.87,3.9,1

28/11/2009,00:44:04,25.37,4.63,4.6,1

28/11/2009,00:44:01,25.4,4.6,4.6,1

28/11/2009,00:44:44,26.27,3.73,3.8,1

28/11/2009,00:44:39,26.16,3.84,3.7,1

28/11/2009,00:44:47,22.74,7.26,7.2,1

28/11/2009,00:44:46,22.81,7.19,7.3,1

28/11/2009,00:47:20,26.47,3.53,4.1,1

28/11/2009,00:47:18,26.27,3.73,3.7,1

28/11/2009,00:47:09,25.92,4.08,3.5,1

28/11/2009,00:48:22,25.71,4.29,4.3,1

28/11/2009,00:48:33,26.09,3.91,3.9,1

28/11/2009,00:58:59,24.61,5.39,12.1,1

28/11/2009,00:56:35,17.9,12.1,11.2,1

28/11/2009,00:56:37,18.77,11.23,5.4,1

28/11/2009,01:11:48,18.08,11.92,11.5,1

28/11/2009,01:11:44,18.49,11.51,11.9,1

28/11/2009,01:28:49,21.08,8.92,10.1,1

28/11/2009,01:28:44,20.94,9.06,9.8,1

28/11/2009,01:28:38,20.25,9.75,9.1,1

28/11/2009,01:28:37,19.91,10.09,8.9,1

28/11/2009,01:48:39,25.75,4.25,4.2,1

28/11/2009,01:48:38,25.85,4.15,4.3,1

28/11/2009,01:53:36,24.85,5.15,5.1,1

28/11/2009,01:53:34,24.92,5.08,5.2,1

28/11/2009,01:57:24,24.75,5.25,5.2,1

28/11/2009,01:57:20,24.57,5.43,5.1,1

28/11/2009,01:57:15,24.88,5.12,5.4,1

28/11/2009,01:57:14,24.85,5.15,5.3,1

28/11/2009,02:03:20,25.58,4.42,4.6,1

28/11/2009,02:03:18,25.4,4.6,4.4,1

28/11/2009,02:11:49,23.5,6.5,6.9,1

28/11/2009,02:11:45,23.09,6.91,6.5,1

28/11/2009,02:16:10,21.08,8.92,9.0,1

28/11/2009,02:16:08,20.98,9.02,8.9,1

28/11/2009,02:16:34,22.12,7.88,7.9,1

28/11/2009,02:16:37,22.43,7.57,7.6,1

28/11/2009,02:16:49,23.99,6.01,6.0,1

28/11/2009,02:16:53,24.19,5.81,5.8,1

28/11/2009,02:18:05,24.71,5.29,5.3,2

28/11/2009,02:20:59,15.45,14.55,14.6,1

28/11/2009,02:27:22,18.97,11.03,11.1,1

28/11/2009,02:27:14,18.87,11.13,11.0,1

28/11/2009,02:27:41,24.37,5.63,5.6,1

28/11/2009,02:32:57,25.58,4.42,4.6,1

28/11/2009,02:32:55,25.4,4.6,4.7,1

28/11/2009,02:32:52,25.44,4.56,4.6,2

28/11/2009,02:32:49,25.33,4.67,4.6,1

28/11/2009,02:32:49,25.4,4.6,4.4,3

28/11/2009,02:41:39,23.71,6.29,6.4,1

28/11/2009,02:41:37,23.64,6.36,6.3,1

28/11/2009,02:46:10,24.71,5.29,4.6,1

28/11/2009,02:46:11,24.92,5.08,4.8,1

28/11/2009,02:46:10,25.13,4.87,4.9,2

28/11/2009,02:46:10,25.23,4.77,5.3,1

28/11/2009,02:46:09,25.4,4.6,5.1,1

28/11/2009,03:04:26,25.09,4.91,5.0,1

28/11/2009,03:04:25,25.33,4.67,5.2,1

28/11/2009,03:04:17,24.85,5.15,4.7,1

28/11/2009,03:04:16,24.99,5.01,4.9,1

28/11/2009,03:10:56,24.37,5.63,2.5,1

28/11/2009,03:10:55,24.26,5.74,2.4,3

28/11/2009,03:10:53,24.23,5.77,2.5,2

28/11/2009,03:10:32,27.51,2.49,2.5,1

28/11/2009,03:10:30,27.62,2.38,5.8,1

28/11/2009,03:10:30,27.55,2.45,5.7,1

28/11/2009,03:10:27,27.48,2.52,5.6,1

28/11/2009,03:14:26,21.6,8.4,3.0,1

28/11/2009,03:14:24,21.39,8.61,10.1,1

28/11/2009,03:14:22,21.12,8.88,9.8,1

28/11/2009,03:14:15,20.77,9.23,9.8,1

28/11/2009,03:14:15,20.7,9.3,9.4,1

28/11/2009,03:14:12,20.29,9.71,8.9,2

28/11/2009,03:14:01,21.19,8.81,8.6,1

28/11/2009,03:14:00,21.36,8.64,8.8,4

28/11/2009,03:13:57,21.12,8.88,9.7,1

28/11/2009,03:13:56,20.63,9.37,9.2,1

28/11/2009,03:13:53,20.18,9.82,9.3,1

28/11/2009,03:13:51,20.22,9.78,8.9,1

28/11/2009,03:13:49,19.87,10.13,8.6,1

28/11/2009,03:12:40,26.99,3.01,8.4,1

28/11/2009,03:18:50,25.41,4.59,4.6,1

28/11/2009,03:27:43,19.84,10.16,10.2,2

28/11/2009,03:38:42,11.48,18.52,18.5,1

28/11/2009,03:52:38,26.93,3.07,3.4,1

28/11/2009,03:52:35,27.03,2.97,3.4,1

28/11/2009,03:52:36,26.89,3.11,3.0,1

28/11/2009,03:52:18,26.65,3.35,3.1,1

28/11/2009,03:52:16,26.58,3.42,3.1,1

28/11/2009,03:55:13,24.41,5.59,6.2,1

28/11/2009,03:55:13,25.01,4.99,5.6,1

28/11/2009,03:55:08,24.86,5.14,5.5,1

28/11/2009,03:55:05,24.65,5.35,5.5,1

28/11/2009,03:55:04,24.65,5.35,5.7,1

28/11/2009,03:55:00,24.23,5.77,5.9,1

28/11/2009,03:54:55,24.09,5.91,5.8,1

28/11/2009,03:54:55,24.27,5.73,5.4,1

28/11/2009,03:54:55,24.48,5.52,5.4,1

28/11/2009,03:54:53,24.48,5.52,5.1,1

28/11/2009,03:54:49,24.41,5.59,5.0,1

28/11/2009,03:54:47,23.77,6.23,5.6,1

28/11/2009,03:58:40,17.16,12.84,13.1,1

28/11/2009,03:58:38,16.88,13.12,12.8,1

28/11/2009,04:04:43,25.18,4.82,5.0,1

28/11/2009,04:04:40,24.97,5.03,5.0,1

28/11/2009,04:04:38,25.04,4.96,4.8,1

28/11/2009,04:09:07,17.06,12.94,17.1,1

28/11/2009,04:09:02,16.85,13.15,13.2,3

28/11/2009,04:08:49,12.94,17.06,12.9,1

28/11/2009,04:14:37,23.77,6.23,6.5,1

28/11/2009,04:14:33,23.6,6.4,6.4,1

28/11/2009,04:14:29,23.49,6.51,6.2,3

28/11/2009,04:18:43,22.68,7.32,21.7,2

28/11/2009,04:17:13,8.33,21.67,21.3,1

28/11/2009,04:17:15,8.72,21.28,21.9,1

28/11/2009,04:17:17,8.09,21.91,7.3,1

28/11/2009,04:20:09,16.04,13.96,13.8,1

28/11/2009,04:20:05,15.47,14.53,14.0,1

28/11/2009,04:20:02,16,14,14.5,1

28/11/2009,04:20:01,16.18,13.82,14.0,1

28/11/2009,04:23:20,23.35,6.65,6.7,1

28/11/2009,04:24:58,24.44,5.56,5.6,1

28/11/2009,04:31:18,18.81,11.19,11.2,1

28/11/2009,04:31:15,18.81,11.19,11.2,5

28/11/2009,04:45:45,25.53,4.47,5.1,1

28/11/2009,04:45:45,25.81,4.19,4.2,1

28/11/2009,04:45:12,24.86,5.14,4.5,1

28/11/2009,04:50:52,26.38,3.62,8.8,1

28/11/2009,04:50:49,26.48,3.52,3.4,1

28/11/2009,04:50:47,26.59,3.41,3.5,1

28/11/2009,04:48:31,21.21,8.79,3.6,1

28/11/2009,04:56:35,14,16,4.5,1

28/11/2009,04:56:32,13.82,16.18,4.9,1

28/11/2009,04:56:30,13.93,16.07,16.4,1

28/11/2009,04:56:26,13.89,16.11,16.1,1

28/11/2009,04:56:26,13.89,16.11,16.1,1

28/11/2009,04:56:24,13.89,16.11,16.1,1

28/11/2009,04:56:21,13.61,16.39,16.1,1

28/11/2009,04:56:19,25.15,4.85,16.2,1

28/11/2009,04:56:19,25.5,4.5,16.0,1

28/11/2009,04:58:46,21.1,8.9,8.9,2

28/11/2009,04:58:44,21.17,8.83,8.8,1

28/11/2009,04:58:42,21.07,8.93,8.9,1

28/11/2009,04:59:22,20.15,9.85,9.9,1

28/11/2009,04:59:22,19.76,10.24,10.2,1

28/11/2009,05:06:39,22.12,7.88,7.5,1

28/11/2009,05:06:37,22.51,7.49,7.9,1

28/11/2009,05:07:50,21.38,8.62,8.6,1

28/11/2009,05:09:03,24.94,5.06,5.1,1

28/11/2009,05:09:06,25.04,4.96,5.0,1

28/11/2009,05:09:59,26.66,3.34,13.9,1

28/11/2009,05:10:01,26.59,3.41,3.3,1

28/11/2009,05:09:41,16.11,13.89,3.4,1

28/11/2009,05:16:06,25.92,4.08,4.1,1

28/11/2009,05:17:24,22.82,7.18,7.2,2

28/11/2009,05:24:17,19.69,10.31,9.9,1

28/11/2009,05:24:14,19.69,10.31,10.3,3

28/11/2009,05:24:14,20.08,9.92,10.3,1

28/11/2009,05:28:18,23.7,6.3,6.3,1

28/11/2009,05:28:22,23.99,6.01,6.0,1

28/11/2009,05:28:27,24.16,5.84,5.8,1

28/11/2009,05:28:29,24.09,5.91,5.9,1

28/11/2009,05:28:30,23.56,6.44,6.4,1

28/11/2009,05:33:11,26.69,3.31,3.3,1

28/11/2009,05:49:32,20.57,9.43,9.4,1

28/11/2009,05:49:34,21.24,8.76,8.8,1

28/11/2009,05:51:26,21.73,8.27,8.2,1

28/11/2009,05:51:25,21.88,8.12,7.9,2

28/11/2009,05:51:24,22.09,7.91,8.2,1

28/11/2009,05:51:24,21.8,8.2,8.1,4

28/11/2009,05:51:21,21.77,8.23,8.3,1

28/11/2009,05:51:45,19.24,10.76,10.5,2

28/11/2009,05:51:45,19.52,10.48,10.8,1

28/11/2009,05:51:49,18.53,11.47,11.5,1

28/11/2009,05:51:52,18.53,11.47,11.5,1

28/11/2009,06:08:50,22.16,7.84,8.1,1

28/11/2009,06:08:46,21.95,8.05,7.8,1

28/11/2009,06:08:57,22.61,7.39,7.4,1

28/11/2009,06:12:57,21.07,8.93,8.9,1

28/11/2009,06:13:02,21.03,8.97,9.0,1

28/11/2009,06:13:30,22.65,7.35,7.4,1

28/11/2009,06:13:33,22.72,7.28,7.3,1

28/11/2009,06:17:12,23.46,6.54,6.3,1

28/11/2009,06:17:11,23.63,6.37,6.4,2

28/11/2009,06:17:09,23.67,6.33,6.5,1

28/11/2009,06:17:14,22.61,7.39,7.4,1

28/11/2009,06:22:04,24.65,5.35,5.4,1

28/11/2009,06:22:04,24.58,5.42,5.4,1

28/11/2009,06:22:07,24.65,5.35,5.4,1

28/11/2009,06:22:29,23.6,6.4,6.4,1

28/11/2009,06:22:30,23.67,6.33,6.3,1

28/11/2009,06:24:40,21.49,8.51,8.5,2

28/11/2009,06:30:20,23.39,6.61,6.6,1

28/11/2009,06:30:24,23.35,6.65,6.7,1

28/11/2009,06:31:35,26.03,3.97,4.0,1

28/11/2009,06:51:02,24.06,5.94,5.9,1

28/11/2009,06:51:32,25.08,4.92,4.7,1

28/11/2009,06:51:29,25.32,4.68,4.9,1

28/11/2009,15:16:32,21.17,8.83,8.8,1

28/11/2009,15:16:36,22.19,7.81,10.6,1

28/11/2009,15:16:32,19.41,10.59,7.8,1

28/11/2009,15:16:37,19.8,10.2,10.2,1

28/11/2009,15:17:04,22.82,7.18,7.2,1

28/11/2009,15:28:57,24.41,5.59,5.5,1

28/11/2009,15:28:55,24.51,5.49,5.6,1

28/11/2009,15:30:03,25.74,4.26,4.2,1

28/11/2009,15:30:01,25.85,4.15,4.2,2

28/11/2009,15:30:02,25.6,4.4,4.4,1

28/11/2009,15:30:01,25.85,4.15,4.3,2

28/11/2009,15:30:51,25.99,4.01,3.6,1

28/11/2009,15:30:48,26.45,3.55,4.0,1

28/11/2009,15:32:53,25.64,4.36,4.4,1

28/11/2009,15:33:50,25.85,4.15,4.2,1

28/11/2009,15:36:19,26.94,3.06,3.1,1

28/11/2009,15:36:26,27.01,2.99,3.0,1

28/11/2009,15:40:48,25.04,4.96,5.0,1

28/11/2009,15:40:50,25.92,4.08,4.1,1

28/11/2009,15:43:40,26.55,3.45,3.5,1

28/11/2009,15:43:40,26.45,3.55,3.6,1

28/11/2009,15:52:03,23.77,6.23,6.2,1

28/11/2009,15:52:03,23.67,6.33,6.3,1

28/11/2009,15:52:07,23.74,6.26,6.3,1

28/11/2009,16:09:19,23.67,6.33,6.3,1

28/11/2009,16:16:17,23.63,6.37,6.4,1

28/11/2009,16:16:31,27.96,2.04,2.0,1

28/11/2009,16:16:30,27.96,2.04,2.0,1

28/11/2009,16:16:26,27.96,2.04,2.0,1

28/11/2009,16:16:35,27.68,2.32,2.3,1

28/11/2009,16:16:38,27.82,2.18,2.2,1

28/11/2009,16:16:39,27.78,2.22,2.2,1

28/11/2009,16:26:10,27.82,2.18,2.4,1

28/11/2009,16:26:09,27.78,2.22,2.2,1

28/11/2009,16:22:21,27.64,2.36,4.2,1

28/11/2009,16:22:26,27.85,2.15,2.2,1

28/11/2009,16:23:59,25.81,4.19,2.2,1

28/11/2009,16:37:59,25.68,4.32,4.7,1

28/11/2009,16:37:47,25.35,4.65,4.3,1

28/11/2009,16:38:46,24.98,5.02,4.5,1

28/11/2009,16:38:46,25.48,4.52,5.0,2

28/11/2009,16:43:13,27.68,2.32,2.3,1

28/11/2009,16:52:54,25.05,4.95,5.0,1

28/11/2009,16:53:00,23.45,6.55,6.6,1

28/11/2009,16:53:25,24.42,5.58,5.6,1

28/11/2009,17:07:12,26.62,3.38,3.2,2

28/11/2009,17:07:10,26.76,3.24,3.4,2

28/11/2009,17:08:24,26.45,3.55,3.2,1

28/11/2009,17:08:24,26.55,3.45,3.2,1

28/11/2009,17:08:22,26.76,3.24,3.2,1

28/11/2009,17:08:16,26.72,3.28,3.2,1

28/11/2009,17:08:11,26.69,3.31,3.3,1

28/11/2009,17:08:10,26.79,3.21,3.2,1

28/11/2009,17:08:07,26.72,3.28,3.3,1

28/11/2009,17:08:06,26.79,3.21,3.3,2

28/11/2009,17:08:05,26.76,3.24,3.2,1

28/11/2009,17:08:05,26.76,3.24,3.5,3

28/11/2009,17:08:04,26.76,3.24,3.6,1

28/11/2009,17:12:50,14.14,15.86,16.0,1

28/11/2009,17:12:47,13.97,16.03,15.9,1

28/11/2009,17:19:28,24.39,5.61,5.6,1

28/11/2009,17:19:30,25.19,4.81,4.8,1

28/11/2009,17:32:11,25.12,4.88,4.9,1

28/11/2009,17:33:50,25.54,4.46,4.5,1

28/11/2009,17:51:41,26.48,3.52,3.3,1

28/11/2009,17:51:38,26.69,3.31,3.5,1

28/11/2009,17:51:44,26.69,3.31,3.2,1

28/11/2009,17:51:44,26.79,3.21,3.3,1

28/11/2009,17:51:44,26.69,3.31,3.3,1

28/11/2009,17:52:25,25.89,4.11,4.1,1

28/11/2009,17:52:30,26.62,3.38,3.4,1

28/11/2009,17:53:46,17.63,12.37,12.8,1

28/11/2009,17:53:42,17.25,12.75,12.4,2

28/11/2009,17:54:16,20.94,9.06,10.4,1

28/11/2009,17:54:11,19.65,10.35,9.1,1

28/11/2009,17:54:18,20.84,9.16,3.3,1

28/11/2009,17:54:20,20.59,9.41,9.2,1

28/11/2009,17:54:22,20.1,9.9,9.4,1

28/11/2009,17:54:23,19.89,10.11,9.9,2

28/11/2009,17:54:25,20.07,9.93,10.1,1

28/11/2009,17:54:25,20.24,9.76,9.8,1

28/11/2009,17:54:18,26.72,3.28,9.9,1

28/11/2009,18:10:57,26.13,3.87,3.8,1

28/11/2009,18:10:54,26.2,3.8,3.8,1

28/11/2009,18:10:55,26.2,3.8,3.9,1

28/11/2009,18:15:56,25.33,4.67,4.7,1

28/11/2009,18:23:22,26.86,3.14,3.8,1

28/11/2009,18:23:21,26.52,3.48,3.6,1

28/11/2009,18:23:19,26.41,3.59,3.6,1

28/11/2009,18:23:17,26.41,3.59,3.6,1

28/11/2009,18:23:17,26.38,3.62,3.5,1

28/11/2009,18:23:16,26.24,3.76,3.1,1

28/11/2009,18:23:35,26.24,3.76,3.8,1

28/11/2009,18:23:36,26.34,3.66,3.7,1

28/11/2009,18:23:38,26.27,3.73,3.7,1

28/11/2009,18:34:11,24.46,5.54,5.5,1

28/11/2009,18:53:07,25.33,4.67,4.7,1

28/11/2009,18:53:16,25.4,4.6,5.8,1

28/11/2009,18:53:10,24.22,5.78,4.6,1

28/11/2009,18:53:22,24.11,5.89,6.0,1

28/11/2009,18:53:21,24.04,5.96,5.9,1

28/11/2009,19:27:48,24.91,5.09,5.1,1

28/11/2009,19:33:06,25.68,4.32,4.3,1

28/11/2009,19:33:06,25.68,4.32,4.3,1

28/11/2009,19:33:08,25.5,4.5,4.6,1

28/11/2009,19:33:10,25.71,4.29,4.5,1

28/11/2009,19:33:06,25.4,4.6,4.3,2

28/11/2009,19:38:13,22.51,7.49,4.7,1

28/11/2009,19:38:10,22.26,7.74,8.0,1

28/11/2009,19:38:08,22.02,7.98,7.7,1

28/11/2009,19:33:11,25.3,4.7,7.5,1

28/11/2009,19:43:10,26.97,3.03,3.2,1

28/11/2009,19:43:09,26.83,3.17,3.2,1

28/11/2009,19:43:09,26.83,3.17,3.2,1

28/11/2009,19:43:07,26.76,3.24,3.0,1

28/11/2009,19:46:48,24.91,5.09,5.1,1

28/11/2009,19:47:20,24.77,5.23,5.2,1

28/11/2009,19:47:24,24.88,5.12,5.1,1

28/11/2009,19:47:26,24.74,5.26,5.3,3

28/11/2009,19:47:26,24.7,5.3,5.3,1

28/11/2009,19:47:29,24.74,5.26,5.3,1

28/11/2009,19:47:32,24.77,5.23,5.9,1

28/11/2009,19:47:32,24.6,5.4,5.2,1

28/11/2009,19:47:30,24.15,5.85,5.4,1

28/11/2009,19:47:32,24.22,5.78,5.8,1

28/11/2009,19:50:53,24.04,5.96,6.0,1

28/11/2009,19:57:02,25.85,4.15,4.2,1

28/11/2009,19:57:45,17,13,12.5,1

28/11/2009,19:57:42,17.14,12.86,11.3,1

28/11/2009,19:57:35,18.57,11.43,11.4,1

28/11/2009,19:57:33,18.67,11.33,12.9,1

28/11/2009,19:57:22,17.53,12.47,13.0,3

28/11/2009,20:24:50,24.81,5.19,5.1,1

28/11/2009,20:24:44,24.88,5.12,2.7,2

28/11/2009,20:24:42,24.95,5.05,5.1,1

28/11/2009,20:24:43,27.3,2.7,3.1,1

28/11/2009,20:24:45,26.91,3.09,5.2,1

28/11/2009,20:49:24,25.05,4.95,5.0,1

28/11/2009,20:49:26,25.71,4.29,4.3,1

28/11/2009,20:49:30,25.75,4.25,4.2,1

28/11/2009,20:49:30,25.78,4.22,4.3,1

28/11/2009,20:49:32,25.68,4.32,4.3,1

28/11/2009,21:01:10,18.01,11.99,12.0,1

28/11/2009,21:01:20,18.88,11.12,11.1,1

28/11/2009,21:16:45,26.62,3.38,3.4,1

28/11/2009,21:16:48,26.17,3.83,3.8,1

28/11/2009,21:35:35,22.4,7.6,7.6,1

28/11/2009,21:39:16,21.36,8.64,8.5,1

28/11/2009,21:39:15,21.46,8.54,8.6,1

28/11/2009,21:39:22,21.64,8.36,8.4,1

28/11/2009,21:53:07,17.66,12.34,11.9,1

28/11/2009,21:53:07,18.08,11.92,12.3,1

28/11/2009,21:53:16,25.85,4.15,4.2,2

28/11/2009,21:53:15,25.78,4.22,4.2,3

28/11/2009,21:53:14,25.82,4.18,4.2,2

28/11/2009,21:55:53,23.66,6.34,6.5,1

28/11/2009,21:55:56,23.52,6.48,6.2,1

28/11/2009,21:55:52,23.76,6.24,6.3,1

28/11/2009,21:55:46,23.52,6.48,6.5,1

28/11/2009,22:01:23,25.16,4.84,4.7,1

28/11/2009,22:01:19,24.91,5.09,4.9,1

28/11/2009,22:01:18,25.09,4.91,5.1,1

28/11/2009,22:01:12,25.26,4.74,4.8,1

28/11/2009,22:10:09,25.05,4.95,4.7,1

28/11/2009,22:10:07,24.42,5.58,5.6,1

28/11/2009,22:09:48,24.42,5.58,5.6,1

28/11/2009,22:05:44,25.33,4.67,5.0,1

28/11/2009,22:12:25,17.98,12.02,12.0,1

28/11/2009,22:12:33,18.74,11.26,11.3,1

28/11/2009,22:27:10,15.71,14.29,14.3,2

28/11/2009,22:27:08,15.68,14.32,14.3,1

28/11/2009,22:36:37,17.25,12.75,8.3,1

28/11/2009,22:36:36,17.28,12.72,8.2,1

28/11/2009,22:35:04,21.85,8.15,12.7,1

28/11/2009,22:35:00,21.71,8.29,12.8,1

28/11/2009,22:40:04,26.17,3.83,3.8,2

28/11/2009,22:40:08,26.27,3.73,3.7,1

28/11/2009,22:42:59,19.3,10.7,9.4,1

28/11/2009,22:42:59,20.59,9.41,10.7,1

28/11/2009,22:47:55,26.06,3.94,3.9,1

28/11/2009,23:02:44,25.68,4.32,4.0,1

28/11/2009,23:02:41,25.96,4.04,4.3,1

28/11/2009,23:06:59,26.17,3.83,3.8,1

28/11/2009,23:07:02,26.24,3.76,3.8,1

28/11/2009,23:13:07,25.12,4.88,4.9,1

28/11/2009,23:18:24,24.01,5.99,6.0,1

28/11/2009,23:18:23,23.97,6.03,6.0,1

28/11/2009,23:24:31,14.32,15.68,15.7,1

28/11/2009,23:24:30,14.35,15.65,15.7,1

28/11/2009,23:26:54,23,7,7.0,1

28/11/2009,23:27:00,23.31,6.69,6.7,1

28/11/2009,23:27:02,23.17,6.83,6.8,1

28/11/2009,23:27:34,24.18,5.82,5.8,1

28/11/2009,23:27:36,24.18,5.82,5.8,1

28/11/2009,23:27:45,25.47,4.53,4.5,1

28/11/2009,23:27:47,25.61,4.39,9.3,1

28/11/2009,23:27:47,25.5,4.5,4.4,1

28/11/2009,23:27:46,20.66,9.34,4.5,1

28/11/2009,23:27:48,20.77,9.23,9.2,1

28/11/2009,23:27:48,20.49,9.51,9.5,1

28/11/2009,23:34:45,26.24,3.76,3.8,1

28/11/2009,23:34:47,26.03,3.97,4.0,1

28/11/2009,23:39:33,21.99,8.01,6.1,2

28/11/2009,23:39:37,23.62,6.38,5.8,1

28/11/2009,23:40:06,22.3,7.7,5.6,1

28/11/2009,23:40:13,23.97,6.03,8.0,1

28/11/2009,23:40:57,25.78,4.22,6.4,1

28/11/2009,23:41:06,25.57,4.43,7.7,1

28/11/2009,23:38:26,24.42,5.58,6.0,1

28/11/2009,23:38:25,24.25,5.75,4.2,1

28/11/2009,23:38:24,23.94,6.06,4.4,1

29/11/2009,00:04:43,23.03,6.97,6.3,1

29/11/2009,00:04:43,23.31,6.69,6.0,1

29/11/2009,00:04:42,23.62,6.38,5.8,1

29/11/2009,00:04:42,23.73,6.27,7.0,1

29/11/2009,00:04:43,23.8,6.2,5.8,1

29/11/2009,00:04:36,23.94,6.06,6.0,1

29/11/2009,00:04:31,22.96,7.04,6.1,1

29/11/2009,00:04:33,23.76,6.24,6.2,1

29/11/2009,00:04:32,23.87,6.13,6.1,1

29/11/2009,00:04:32,24.01,5.99,6.3,1

29/11/2009,00:04:32,24.18,5.82,6.4,1

29/11/2009,00:04:30,24.22,5.78,6.2,2

29/11/2009,00:04:29,24.04,5.96,6.7,1

29/11/2009,00:04:25,23.69,6.31,7.0,1

29/11/2009,00:06:13,15.82,14.18,14.3,2

29/11/2009,00:06:10,15.71,14.29,14.2,1

29/11/2009,00:06:28,21.99,8.01,8.0,1

29/11/2009,00:06:29,22.26,7.74,7.7,1

29/11/2009,00:08:05,25.85,4.15,4.2,1

29/11/2009,00:17:02,25.61,4.39,4.4,1

29/11/2009,00:17:04,25.68,4.32,4.3,1

29/11/2009,00:19:01,24.95,5.05,5.0,1

29/11/2009,00:18:59,25.02,4.98,5.1,1

29/11/2009,00:27:36,22.12,7.88,4.6,1

29/11/2009,00:27:33,22.09,7.91,2.7,1

29/11/2009,00:27:25,21.64,8.36,2.8,1

29/11/2009,00:27:23,21.6,8.4,8.4,1

29/11/2009,00:25:24,25.37,4.63,8.4,1

29/11/2009,00:25:37,27.28,2.72,7.9,1

29/11/2009,00:25:40,27.21,2.79,7.9,1

29/11/2009,00:32:35,25.37,4.63,10.5,2

29/11/2009,00:32:35,25.78,4.22,9.9,1

29/11/2009,00:32:33,25.99,4.01,9.9,1

29/11/2009,00:32:27,25.33,4.67,6.5,1

29/11/2009,00:32:24,25.23,4.77,7.0,1

29/11/2009,00:32:21,25.02,4.98,7.2,1

29/11/2009,00:31:56,22.82,7.18,7.8,1

29/11/2009,00:31:56,22.82,7.18,7.4,1

29/11/2009,00:31:55,22.61,7.39,7.2,1

29/11/2009,00:31:53,22.79,7.21,7.2,1

29/11/2009,00:31:53,23.03,6.97,18.0,1

29/11/2009,00:31:53,23.55,6.45,15.5,1

29/11/2009,00:31:16,20.1,9.9,5.0,1

29/11/2009,00:31:14,20.14,9.86,4.8,1

29/11/2009,00:31:10,19.48,10.52,13.2,1

29/11/2009,00:32:18,12.02,17.98,4.7,1

29/11/2009,00:32:19,14.46,15.54,4.0,1

29/11/2009,00:32:25,16.76,13.24,4.2,1

29/11/2009,00:31:54,22.23,7.77,4.6,1

29/11/2009,00:38:51,10.87,19.13,4.8,1

29/11/2009,00:38:49,10.87,19.13,4.9,1

29/11/2009,00:37:15,25.16,4.84,4.7,1

29/11/2009,00:37:12,25.26,4.74,4.8,1

29/11/2009,00:37:07,25.09,4.91,19.1,1

29/11/2009,00:37:06,25.16,4.84,19.1,1

29/11/2009,00:39:21,22.72,7.28,7.3,2

29/11/2009,00:39:22,23.2,6.8,6.8,1

29/11/2009,00:39:26,23.14,6.86,6.9,1

29/11/2009,00:39:35,24.22,5.78,5.8,1

29/11/2009,00:39:36,24.11,5.89,5.9,1

29/11/2009,00:39:45,25.05,4.95,5.0,1

29/11/2009,00:39:48,25.05,4.95,5.0,1

29/11/2009,00:39:48,24.95,5.05,5.1,1

29/11/2009,00:39:50,25.02,4.98,5.0,2

29/11/2009,00:39:51,24.95,5.05,5.1,1

29/11/2009,00:39:55,24.98,5.02,5.0,1

29/11/2009,00:39:56,24.98,5.02,5.0,1

29/11/2009,00:39:58,25.02,4.98,5.0,1

29/11/2009,00:40:00,25.09,4.91,4.9,1

29/11/2009,00:43:25,20.73,9.27,9.3,1

29/11/2009,00:44:11,23.69,6.31,6.3,1

29/11/2009,00:46:35,25.16,4.84,4.8,1

29/11/2009,00:46:33,25.19,4.81,4.8,1

29/11/2009,00:57:03,18.74,11.26,11.3,1

29/11/2009,01:01:52,25.44,4.56,4.5,1

29/11/2009,01:01:49,25.19,4.81,4.8,1

29/11/2009,01:01:47,25.5,4.5,4.6,1

29/11/2009,01:04:58,25.09,4.91,4.9,1

29/11/2009,01:05:56,26.31,3.69,3.7,1

29/11/2009,01:15:44,20.17,9.83,10.5,1

29/11/2009,01:15:41,19.51,10.49,9.8,1

29/11/2009,01:15:49,26.1,3.9,3.9,1

29/11/2009,01:23:54,25.75,4.25,9.6,1

29/11/2009,01:23:57,25.92,4.08,9.6,1

29/11/2009,01:23:51,25.89,4.11,4.2,1

29/11/2009,01:21:55,25.99,4.01,3.9,1

29/11/2009,01:21:53,26.06,3.94,4.0,1

29/11/2009,01:20:51,25.82,4.18,4.1,1

29/11/2009,01:20:17,20.45,9.55,4.3,1

29/11/2009,01:20:14,20.42,9.58,4.1,1

29/11/2009,01:31:01,26.06,3.94,4.2,1

29/11/2009,01:30:55,25.85,4.15,4.4,1

29/11/2009,01:30:53,25.61,4.39,4.4,1

29/11/2009,01:30:52,25.64,4.36,4.2,1

29/11/2009,01:30:49,25.78,4.22,3.9,1

29/11/2009,01:33:31,23.59,6.41,6.5,1

29/11/2009,01:33:26,23.41,6.59,6.6,1

29/11/2009,01:33:23,23.41,6.59,6.6,1

29/11/2009,01:33:21,23.48,6.52,6.4,1

29/11/2009,01:42:52,25.78,4.22,4.4,1

29/11/2009,01:42:48,25.64,4.36,4.4,1

29/11/2009,01:42:46,25.64,4.36,4.4,1

29/11/2009,01:42:43,25.64,4.36,4.2,1

29/11/2009,01:47:20,21.64,8.36,8.1,1

29/11/2009,01:47:19,21.88,8.12,8.4,1

29/11/2009,01:50:15,19.16,10.84,11.1,1

29/11/2009,01:50:10,19.16,10.84,10.8,3

29/11/2009,01:50:07,18.92,11.08,10.8,1

29/11/2009,01:52:09,10.17,19.83,19.9,1

29/11/2009,01:52:06,10.07,19.93,19.8,1

29/11/2009,02:02:47,18.36,11.64,2.7,1

29/11/2009,02:02:52,19.09,10.91,2.7,1

29/11/2009,02:03:04,22.44,7.56,11.6,1

29/11/2009,02:03:04,22.33,7.67,10.9,1

29/11/2009,02:03:06,22.16,7.84,7.6,1

29/11/2009,02:03:47,21.67,8.33,7.7,1

29/11/2009,02:03:55,21.71,8.29,7.8,1

29/11/2009,02:04:16,22.72,7.28,8.3,1

29/11/2009,02:04:18,22.68,7.32,8.3,1

29/11/2009,01:56:42,27.35,2.65,7.3,1

29/11/2009,01:56:39,27.28,2.72,7.3,1

29/11/2009,02:04:59,24.88,5.12,5.4,1

29/11/2009,02:04:56,24.63,5.37,13.8,1

29/11/2009,02:04:48,24.56,5.44,13.9,1

29/11/2009,02:04:53,17.21,12.79,12.8,1

29/11/2009,02:04:51,16.24,13.76,14.2,1

29/11/2009,02:04:51,16.13,13.87,14.4,1

29/11/2009,02:04:55,15.85,14.15,14.5,1

29/11/2009,02:04:55,15.64,14.36,5.4,1

29/11/2009,02:04:55,15.54,14.46,5.1,1

29/11/2009,02:08:51,11.6,18.4,18.4,1

29/11/2009,02:08:49,11.57,18.43,18.4,1

29/11/2009,02:10:11,24.67,5.33,5.1,1

29/11/2009,02:10:11,24.91,5.09,5.3,2

29/11/2009,02:10:25,20.94,9.06,9.4,1

29/11/2009,02:10:20,20.63,9.37,9.1,1

29/11/2009,02:15:21,22.33,7.67,7.7,2

29/11/2009,02:20:07,23.24,6.76,6.7,1

29/11/2009,02:20:04,23.34,6.66,6.8,1

29/11/2009,02:23:40,23.73,6.27,6.3,1

29/11/2009,02:23:42,23.55,6.45,6.5,1

29/11/2009,02:24:36,24.01,5.99,6.0,1

29/11/2009,02:24:37,23.94,6.06,7.1,1

29/11/2009,02:24:36,22.86,7.14,7.2,1

29/11/2009,02:24:36,22.82,7.18,6.1,1

29/11/2009,02:24:37,22.68,7.32,7.3,1

29/11/2009,02:25:15,27.18,2.82,2.8,1

29/11/2009,02:25:13,27.21,2.79,2.8,1

29/11/2009,02:31:41,24.25,5.75,5.7,1

29/11/2009,02:31:57,22.26,7.74,5.8,1

29/11/2009,02:31:55,22.09,7.91,7.9,1

29/11/2009,02:31:39,24.29,5.71,7.7,1

29/11/2009,02:34:01,19.86,10.14,10.1,1

29/11/2009,02:33:58,19.93,10.07,10.1,1

29/11/2009,02:34:16,22.54,7.46,7.5,1

29/11/2009,02:34:21,22.89,7.11,6.9,1

29/11/2009,02:34:21,23.07,6.93,7.1,1

29/11/2009,02:34:24,23.07,6.93,7.6,1

29/11/2009,02:34:24,22.86,7.14,6.9,1

29/11/2009,02:34:24,22.54,7.46,7.1,2

29/11/2009,02:34:23,22.4,7.6,7.5,2

29/11/2009,02:34:25,22.19,7.81,8.3,1

29/11/2009,02:34:24,21.71,8.29,7.8,1

29/11/2009,02:40:46,17.98,12.02,12.4,1

29/11/2009,02:40:40,17.56,12.44,12.0,1

29/11/2009,02:41:46,23.27,6.73,6.5,2

29/11/2009,02:41:41,23.17,6.83,6.8,1

29/11/2009,02:41:40,23.48,6.52,6.7,1

29/11/2009,02:43:05,21.81,8.19,8.2,1

29/11/2009,02:46:50,27.32,2.68,2.8,1

29/11/2009,02:46:44,27.25,2.75,2.8,1

29/11/2009,02:46:44,27.25,2.75,2.7,1

29/11/2009,02:47:16,25.68,4.32,4.3,1

29/11/2009,02:47:14,25.68,4.32,4.3,1

29/11/2009,02:47:50,27.18,2.82,2.8,1

29/11/2009,02:48:19,15.82,14.18,13.9,1

29/11/2009,02:48:18,16.06,13.94,14.2,1

29/11/2009,02:49:54,22.02,7.98,8.0,1

29/11/2009,02:52:24,26.9,3.1,3.1,1

29/11/2009,02:52:43,25.19,4.81,4.9,1

29/11/2009,02:52:41,25.12,4.88,4.8,2

29/11/2009,02:53:27,27.14,2.86,2.9,1

29/11/2009,02:55:23,27.07,2.93,3.8,1

29/11/2009,02:55:19,27.11,2.89,2.8,1

29/11/2009,02:55:14,27.21,2.79,2.8,1

29/11/2009,02:55:06,27.18,2.82,2.9,1

29/11/2009,02:54:57,26.24,3.76,2.9,1

29/11/2009,03:06:16,23.24,6.76,6.2,2

29/11/2009,03:06:14,23.2,6.8,6.8,1

29/11/2009,03:05:59,23.8,6.2,6.8,2

29/11/2009,03:10:55,16.83,13.17,2.6,1

29/11/2009,03:10:23,27.04,2.96,2.7,1

29/11/2009,03:10:21,26.86,3.14,3.1,1

29/11/2009,03:09:49,27.32,2.68,3.0,1

29/11/2009,03:09:47,27.42,2.58,13.2,1

29/11/2009,03:11:05,26.59,3.41,3.2,1

29/11/2009,03:11:05,26.65,3.35,3.4,1

29/11/2009,03:11:05,26.79,3.21,3.4,1

29/11/2009,03:12:31,25.47,4.53,4.5,1

29/11/2009,03:21:14,22.72,7.28,3.2,1

29/11/2009,03:21:00,19.44,10.56,3.2,1

29/11/2009,03:21:00,19.62,10.38,3.1,1

29/11/2009,03:21:01,26.65,3.35,10.4,1

29/11/2009,03:21:01,26.83,3.17,10.6,1

29/11/2009,03:21:00,26.9,3.1,3.2,1

29/11/2009,03:20:59,26.79,3.21,3.4,1

29/11/2009,03:20:58,26.83,3.17,7.3,1

29/11/2009,03:26:04,18.57,11.43,7.5,1

29/11/2009,03:26:04,18.74,11.26,7.9,1

29/11/2009,03:26:04,18.85,11.15,7.3,1

29/11/2009,03:26:04,18.92,11.08,7.5,1

29/11/2009,03:26:04,19.2,10.8,7.6,1

29/11/2009,03:25:41,18.43,11.57,7.0,1

29/11/2009,03:23:33,22.12,7.88,3.2,1

29/11/2009,03:23:32,22.47,7.53,2.7,1

29/11/2009,03:24:08,23,7,11.6,1

29/11/2009,03:24:05,22.72,7.28,10.8,1

29/11/2009,03:24:06,22.51,7.49,11.1,1

29/11/2009,03:24:07,22.37,7.63,11.2,1

29/11/2009,03:24:15,26.76,3.24,11.3,1

29/11/2009,03:24:48,27.35,2.65,11.4,1

29/11/2009,03:26:29,26.9,3.1,3.3,1

29/11/2009,03:26:27,26.72,3.28,3.1,1

29/11/2009,03:30:52,23.52,6.48,6.9,1

29/11/2009,03:30:49,23.07,6.93,6.5,1

29/11/2009,03:32:38,25.23,4.77,4.8,1

29/11/2009,03:35:00,26.9,3.1,3.1,1

29/11/2009,03:35:09,27.11,2.89,2.9,1

29/11/2009,03:35:13,26.9,3.1,3.1,1

29/11/2009,03:35:18,26.86,3.14,3.1,1

29/11/2009,03:35:26,26.93,3.07,3.1,1

29/11/2009,03:35:59,23.52,6.48,6.9,1

29/11/2009,03:35:55,23.24,6.76,6.8,1

29/11/2009,03:35:48,23.1,6.9,6.8,1

29/11/2009,03:35:54,23.17,6.83,6.5,1

29/11/2009,03:43:59,15.23,14.77,14.8,1

29/11/2009,03:43:57,15.23,14.77,14.8,1

29/11/2009,03:44:47,21.67,8.33,8.1,1

29/11/2009,03:44:46,21.85,8.15,8.2,1

29/11/2009,03:44:46,21.95,8.05,8.3,1

29/11/2009,03:47:53,18.92,11.08,10.7,1

29/11/2009,03:47:51,19.3,10.7,11.1,1

29/11/2009,03:49:28,25.09,4.91,4.9,1

29/11/2009,03:49:30,24.91,5.09,5.1,1

29/11/2009,03:53:08,18.92,11.08,11.2,1

29/11/2009,03:53:05,18.85,11.15,11.1,1

29/11/2009,03:54:04,20.1,9.9,9.6,1

29/11/2009,03:54:03,20.38,9.62,9.9,1

29/11/2009,03:54:28,21.32,8.68,8.7,1

29/11/2009,03:54:31,21.46,8.54,8.5,1

29/11/2009,03:54:34,21.64,8.36,8.4,1

29/11/2009,03:54:38,26.2,3.8,3.8,1

29/11/2009,03:55:18,22.02,7.98,7.7,1

29/11/2009,03:55:18,22.3,7.7,8.0,1

29/11/2009,03:55:20,22.3,7.7,7.7,1

29/11/2009,03:58:06,25.02,4.98,4.8,1

29/11/2009,03:58:06,25.12,4.88,4.9,1

29/11/2009,03:58:39,20.84,9.16,5.0,1

29/11/2009,03:58:42,20.73,9.27,5.0,1

29/11/2009,03:59:18,23.62,6.38,9.2,1

29/11/2009,03:59:22,23.9,6.1,9.3,1

29/11/2009,03:59:02,26.13,3.87,3.9,1

29/11/2009,03:58:07,25.05,4.95,6.4,1

29/11/2009,03:58:06,25.19,4.81,6.1,1

29/11/2009,04:01:24,15.05,14.95,15.0,1

29/11/2009,04:01:31,17.28,12.72,12.7,1

29/11/2009,04:01:35,20.17,9.83,9.8,1

29/11/2009,04:01:51,22.06,7.94,7.6,1

29/11/2009,04:01:51,22.16,7.84,7.8,1

29/11/2009,04:01:50,22.4,7.6,7.9,1

29/11/2009,04:01:53,21.85,8.15,8.2,1

29/11/2009,04:01:59,20.94,9.06,8.4,1

29/11/2009,04:01:58,21.18,8.82,8.5,1

29/11/2009,04:01:58,21.32,8.68,8.7,1

29/11/2009,04:01:58,21.46,8.54,8.8,1

29/11/2009,04:01:58,21.64,8.36,9.1,1

29/11/2009,04:02:45,24.91,5.09,4.8,1

29/11/2009,04:02:45,25.16,4.84,5.1,1

29/11/2009,04:08:23,24.18,5.82,5.8,1

29/11/2009,04:08:22,24.22,5.78,5.8,1

29/11/2009,04:10:37,19.82,10.18,8.4,2

29/11/2009,04:10:36,19.89,10.11,8.9,1

29/11/2009,04:10:28,19.82,10.18,10.2,1

29/11/2009,04:10:25,21.15,8.85,10.1,1

29/11/2009,04:10:24,21.6,8.4,10.2,1

29/11/2009,04:10:55,23.14,6.86,6.9,1

29/11/2009,04:11:21,24.22,5.78,5.8,1

29/11/2009,04:12:01,24.39,5.61,5.6,2

29/11/2009,04:12:03,24.67,5.33,5.3,1

29/11/2009,04:12:07,24.6,5.4,5.2,2

29/11/2009,04:12:07,24.81,5.19,5.4,1

29/11/2009,04:14:17,22.06,7.94,7.6,1

29/11/2009,04:14:15,22.12,7.88,7.7,3

29/11/2009,04:14:10,22.54,7.46,7.5,1

29/11/2009,04:14:07,22.33,7.67,7.9,1

29/11/2009,04:14:06,22.4,7.6,7.9,2

29/11/2009,04:14:38,22.65,7.35,7.4,1

29/11/2009,04:14:40,22.82,7.18,7.2,1

29/11/2009,04:15:56,24.56,5.44,5.2,1

29/11/2009,04:15:56,24.77,5.23,5.4,1

29/11/2009,04:19:22,20.38,9.62,4.3,1

29/11/2009,04:19:21,20.73,9.27,9.3,1

29/11/2009,04:17:59,25.68,4.32,9.6,1

29/11/2009,04:20:57,16.86,13.14,3.8,1

29/11/2009,04:20:56,17.14,12.86,3.9,1

29/11/2009,04:20:47,26.06,3.94,12.9,1

29/11/2009,04:20:46,26.24,3.76,13.1,1

29/11/2009,04:21:42,22.54,7.46,7.5,1

29/11/2009,04:23:08,25.61,4.39,4.4,1

29/11/2009,04:23:14,24.95,5.05,5.3,1

29/11/2009,04:23:11,24.74,5.26,5.1,1

29/11/2009,04:24:41,23.83,6.17,6.2,1

29/11/2009,04:24:38,23.8,6.2,6.2,1

29/11/2009,04:24:36,23.83,6.17,6.2,1

29/11/2009,04:27:22,25.09,4.91,5.0,1

29/11/2009,04:27:19,24.74,5.26,5.3,1

29/11/2009,04:27:19,24.98,5.02,4.9,2

29/11/2009,04:31:34,20.42,9.58,8.7,1

29/11/2009,04:31:34,21.32,8.68,9.6,1

29/11/2009,04:31:41,20.91,9.09,9.1,1

29/11/2009,04:31:43,20.91,9.09,9.1,1

29/11/2009,04:31:44,21.25,8.75,8.8,1

29/11/2009,04:31:45,21.01,8.99,9.0,1

29/11/2009,04:31:47,20.63,9.37,9.4,1

29/11/2009,04:31:53,20.45,9.55,9.6,1

29/11/2009,04:31:57,20.84,9.16,9.2,1

29/11/2009,04:31:59,21.39,8.61,8.6,1

29/11/2009,04:32:28,23,7,6.7,1

29/11/2009,04:32:27,23.27,6.73,7.0,1

29/11/2009,04:34:31,26.06,3.94,3.9,1

29/11/2009,04:35:02,26.2,3.8,3.8,1

29/11/2009,04:35:04,26.2,3.8,3.8,1

29/11/2009,04:36:51,20.52,9.48,9.5,1

29/11/2009,04:36:53,20.91,9.09,9.1,1

29/11/2009,04:36:57,20.91,9.09,9.1,2

29/11/2009,04:36:59,21.08,8.92,8.9,1

29/11/2009,04:37:08,22.23,7.77,7.8,1

29/11/2009,04:37:14,23.24,6.76,6.8,1

29/11/2009,04:37:16,23.24,6.76,6.8,1

29/11/2009,04:37:17,23.14,6.86,6.9,2

29/11/2009,04:37:27,23,7,7.0,1

29/11/2009,04:37:30,23.59,6.41,6.4,1

29/11/2009,04:37:31,23.41,6.59,6.6,1

29/11/2009,04:37:34,23.45,6.55,6.6,1

29/11/2009,04:38:01,23.9,6.1,6.1,1

29/11/2009,04:38:02,24.01,5.99,6.0,1

29/11/2009,04:38:29,25.16,4.84,4.8,1

29/11/2009,04:38:31,25.23,4.77,4.8,1

29/11/2009,04:43:08,23.62,6.38,6.2,1

29/11/2009,04:43:08,23.76,6.24,6.4,1

29/11/2009,04:45:48,24.39,5.61,5.6,1

29/11/2009,04:45:46,24.39,5.61,5.6,1

29/11/2009,04:47:55,26.1,3.9,3.9,1

29/11/2009,04:48:36,25.99,4.01,4.0,1

29/11/2009,04:58:40,23.94,6.06,6.2,1

29/11/2009,04:58:37,23.8,6.2,6.2,1

29/11/2009,04:58:40,24.11,5.89,5.9,1

29/11/2009,04:58:37,23.83,6.17,6.1,1

29/11/2009,04:59:12,25.61,4.39,4.5,1

29/11/2009,04:59:09,25.47,4.53,4.4,1

29/11/2009,05:02:38,19.48,10.52,10.5,1

29/11/2009,05:02:36,19.55,10.45,10.5,1

29/11/2009,05:02:58,20.14,9.86,9.9,1

29/11/2009,05:03:11,22.68,7.32,7.3,1

29/11/2009,05:03:14,22.37,7.63,8.6,1

29/11/2009,05:03:17,22.47,7.53,7.6,1

29/11/2009,05:03:17,22.26,7.74,8.8,1

29/11/2009,05:03:17,22.16,7.84,8.1,1

29/11/2009,05:03:16,21.95,8.05,7.5,1

29/11/2009,05:03:13,21.36,8.64,7.7,1

29/11/2009,05:03:14,21.18,8.82,7.8,1

29/11/2009,05:04:35,25.78,4.22,4.2,1

29/11/2009,05:04:33,25.85,4.15,4.2,1

29/11/2009,05:04:40,27.21,2.79,2.8,1

29/11/2009,05:05:52,25.99,4.01,4.0,1

29/11/2009,05:05:56,25.64,4.36,4.4,1

29/11/2009,05:06:30,25.61,4.39,4.4,1

29/11/2009,05:06:33,25.68,4.32,4.3,1

29/11/2009,05:07:01,25.99,4.01,4.0,1

29/11/2009,05:07:03,25.89,4.11,4.1,1

29/11/2009,05:18:23,22.33,7.67,7.7,1

29/11/2009,05:18:27,22.26,7.74,7.7,2

29/11/2009,05:18:28,22.12,7.88,7.9,1

29/11/2009,05:18:31,22.3,7.7,7.7,1

29/11/2009,05:18:33,22.4,7.6,7.6,1

29/11/2009,05:18:59,24.81,5.19,5.2,1

29/11/2009,05:19:08,25.47,4.53,4.5,1

29/11/2009,05:19:08,25.44,4.56,4.6,1

29/11/2009,05:19:10,25.26,4.74,4.7,1

29/11/2009,05:19:36,25.85,4.15,4.2,1

29/11/2009,05:29:57,27.28,2.72,2.7,1

29/11/2009,05:29:55,27.28,2.72,2.6,1

29/11/2009,05:29:55,27.39,2.61,2.5,1

29/11/2009,05:29:54,27.49,2.51,2.6,1

29/11/2009,05:29:53,27.42,2.58,2.7,1

29/11/2009,05:29:52,27.35,2.65,2.7,1

29/11/2009,05:33:39,9.51,20.49,20.5,1

29/11/2009,05:35:32,23.62,6.38,6.7,2

29/11/2009,05:35:29,23.31,6.69,6.4,3

29/11/2009,05:36:26,25.44,4.56,4.3,1

29/11/2009,05:36:25,25.71,4.29,4.6,1

29/11/2009,05:41:06,25.89,4.11,4.1,1

29/11/2009,05:54:13,27,3,2.9,1

29/11/2009,05:54:12,26.9,3.1,2.9,1

29/11/2009,05:54:11,27.07,2.93,2.9,1

29/11/2009,05:54:06,27.07,2.93,3.1,1

29/11/2009,05:54:05,27.07,2.93,3.0,1

29/11/2009,06:03:15,25.68,4.32,4.3,2

29/11/2009,06:03:11,25.71,4.29,3.4,1

29/11/2009,06:03:13,26.59,3.41,4.3,1

29/11/2009,06:08:00,25.33,4.67,4.7,1

29/11/2009,06:19:24,26.76,3.24,3.2,1

29/11/2009,06:19:26,26.59,3.41,3.4,1

29/11/2009,06:19:30,26.69,3.31,3.3,1

29/11/2009,06:19:37,26.55,3.45,3.5,1

29/11/2009,06:19:39,25.47,4.53,4.5,1

29/11/2009,06:19:42,25.61,4.39,4.4,1

29/11/2009,06:19:47,26.62,3.38,3.4,1

29/11/2009,06:19:50,26.76,3.24,3.2,1

29/11/2009,06:35:33,24.98,5.02,16.5,1

29/11/2009,06:35:36,25.23,4.77,16.3,1

29/11/2009,06:35:36,25.47,4.53,5.0,1

29/11/2009,06:35:22,13.69,16.31,4.5,1

29/11/2009,06:35:20,13.48,16.52,4.8,1

29/11/2009,06:44:54,21.78,8.22,8.2,1

29/11/2009,06:53:21,24.18,5.82,8.9,1

29/11/2009,06:53:13,21.15,8.85,5.8,1

29/11/2009,07:14:16,26.93,3.07,2.4,1

29/11/2009,07:14:15,26.9,3.1,2.7,1

29/11/2009,07:14:06,26.38,3.62,3.1,1

29/11/2009,07:14:04,26.76,3.24,3.3,2

29/11/2009,07:14:02,26.69,3.31,3.2,1

29/11/2009,07:14:01,26.9,3.1,3.6,1

29/11/2009,07:13:58,27.32,2.68,3.1,1

29/11/2009,07:13:57,27.56,2.44,3.1,1

29/11/2009,15:11:49,25.12,4.88,4.2,1

29/11/2009,15:11:49,25.44,4.56,4.6,1

29/11/2009,15:11:49,25.78,4.22,4.9,1

29/11/2009,15:22:55,21.53,8.47,3.9,1

29/11/2009,15:22:54,21.71,8.29,6.9,1

29/11/2009,15:20:17,21.99,8.01,7.9,1

29/11/2009,15:20:16,22.06,7.94,8.0,1

29/11/2009,15:20:05,23.14,6.86,8.3,1

29/11/2009,15:19:03,26.13,3.87,8.5,1

29/11/2009,15:32:15,26.45,3.55,3.4,1

29/11/2009,15:32:14,26.59,3.41,3.6,1

29/11/2009,15:33:06,26.59,3.41,2.9,1

29/11/2009,15:33:04,27.14,2.86,3.4,2

29/11/2009,15:36:45,27.04,2.96,3.0,1

29/11/2009,15:37:00,22.06,7.94,7.9,1

29/11/2009,15:37:10,23.45,6.55,5.9,1

29/11/2009,15:37:06,23.45,6.55,6.4,1

29/11/2009,15:37:06,23.52,6.48,6.6,1

29/11/2009,15:37:05,23.66,6.34,6.3,1

29/11/2009,15:37:03,23.45,6.55,6.5,2

29/11/2009,15:37:03,23.59,6.41,6.6,1

29/11/2009,15:37:03,24.11,5.89,6.6,1

29/11/2009,15:38:02,26.9,3.1,3.4,1

29/11/2009,15:37:59,26.59,3.41,3.4,1

29/11/2009,15:37:58,26.65,3.35,7.0,1

29/11/2009,15:38:00,22.96,7.04,3.1,1

29/11/2009,15:38:17,26.45,3.55,3.6,1

29/11/2009,15:39:17,23.52,6.48,6.5,1

29/11/2009,15:44:17,27.49,2.51,2.5,2

29/11/2009,15:44:15,27.46,2.54,2.5,2

29/11/2009,15:47:39,27.49,2.51,2.5,1

29/11/2009,15:48:37,26.31,3.69,3.6,1

29/11/2009,15:48:35,26.45,3.55,3.7,1

29/11/2009,15:49:05,23.97,6.03,6.0,1

29/11/2009,15:49:11,25.85,4.15,6.0,1

29/11/2009,15:49:13,23.87,6.13,4.2,1

29/11/2009,15:49:10,24.04,5.96,6.1,1

29/11/2009,15:49:34,26.76,3.24,7.2,1

29/11/2009,15:49:34,27.04,2.96,7.0,1

29/11/2009,15:49:34,27.11,2.89,3.1,1

29/11/2009,15:49:29,26.9,3.1,3.1,1

29/11/2009,15:49:29,26.93,3.07,2.9,1

29/11/2009,15:49:21,23,7,3.0,1

29/11/2009,15:49:19,22.82,7.18,3.2,1

29/11/2009,15:51:09,26.45,3.55,2.6,1

29/11/2009,15:51:09,27.42,2.58,3.6,1

29/11/2009,15:52:45,27.42,2.58,2.5,1

29/11/2009,15:52:34,27.46,2.54,2.6,1

29/11/2009,15:53:28,23.48,6.52,6.5,1

29/11/2009,15:53:32,24.91,5.09,5.1,2

29/11/2009,15:54:19,27.21,2.79,2.8,1

29/11/2009,15:55:18,27.25,2.75,2.7,1

29/11/2009,15:55:15,27.28,2.72,2.8,1

29/11/2009,15:55:29,27.49,2.51,2.5,1

29/11/2009,15:56:21,23.73,6.27,6.3,1

29/11/2009,15:56:19,23.66,6.34,6.3,1

29/11/2009,15:57:31,27.39,2.61,2.4,2

29/11/2009,15:57:28,27.6,2.4,2.4,1

29/11/2009,15:57:25,27.63,2.37,2.6,1

29/11/2009,15:59:05,27.63,2.37,2.3,1

29/11/2009,15:59:05,27.7,2.3,2.3,1

29/11/2009,15:59:03,27.73,2.27,2.4,2

29/11/2009,16:00:08,27.7,2.3,2.2,1

29/11/2009,16:00:07,27.77,2.23,2.3,1

29/11/2009,16:00:12,27.6,2.4,2.4,1

29/11/2009,16:01:02,27.77,2.23,2.2,1

29/11/2009,16:00:59,27.84,2.16,2.2,1

29/11/2009,16:01:58,27.63,2.37,2.2,1

29/11/2009,16:01:57,27.7,2.3,2.2,1

29/11/2009,16:01:56,27.84,2.16,2.3,1

29/11/2009,16:01:56,27.84,2.16,2.4,2

29/11/2009,16:02:02,27.84,2.16,2.2,1

29/11/2009,16:02:05,27.98,2.02,2.0,2

29/11/2009,16:02:12,27.84,2.16,2.2,1

29/11/2009,16:02:13,27.7,2.3,2.3,2

29/11/2009,16:02:17,27.63,2.37,2.4,1

29/11/2009,16:02:21,27.91,2.09,2.1,2

29/11/2009,16:02:24,27.91,2.09,2.1,3

29/11/2009,16:02:25,27.87,2.13,2.1,1

29/11/2009,16:02:28,27.77,2.23,2.2,1

29/11/2009,16:02:36,27.87,2.13,2.1,1

29/11/2009,16:02:40,27.77,2.23,2.2,1

29/11/2009,16:03:21,27.87,2.13,2.1,1

29/11/2009,16:03:16,27.87,2.13,2.1,1

29/11/2009,16:04:21,27.87,2.13,2.1,1

29/11/2009,16:04:35,27.77,2.23,2.2,1

29/11/2009,16:04:59,25.37,4.63,4.7,2

29/11/2009,16:04:55,25.3,4.7,4.6,1

29/11/2009,16:07:24,27.91,2.09,2.1,1

29/11/2009,16:08:00,27.46,2.54,2.5,1

29/11/2009,16:09:27,27.67,2.33,2.3,1

29/11/2009,16:10:28,25.57,4.43,4.6,1

29/11/2009,16:10:23,25.4,4.6,4.6,1

29/11/2009,16:10:22,25.4,4.6,4.4,1

29/11/2009,16:11:29,27.67,2.33,2.2,1

29/11/2009,16:11:26,27.6,2.4,2.1,1

29/11/2009,16:11:25,27.7,2.3,2.3,1

29/11/2009,16:11:25,27.87,2.13,2.4,1

29/11/2009,16:11:22,27.8,2.2,2.3,1

29/11/2009,16:12:03,27.6,2.4,2.0,1

29/11/2009,16:12:02,28.01,1.99,2.4,1

29/11/2009,16:12:18,27.94,2.06,2.1,1

29/11/2009,16:12:19,27.98,2.02,2.0,1

29/11/2009,16:12:21,27.98,2.02,2.0,1

29/11/2009,16:12:23,27.98,2.02,2.0,1

29/11/2009,16:12:24,27.98,2.02,2.0,1

29/11/2009,16:12:56,27.91,2.09,2.2,1

29/11/2009,16:12:51,27.84,2.16,2.2,2

29/11/2009,16:12:49,27.84,2.16,2.1,1

29/11/2009,16:13:47,27.73,2.27,2.1,1

29/11/2009,16:13:46,27.94,2.06,2.3,1

29/11/2009,16:13:48,27.8,2.2,2.2,2

29/11/2009,16:14:21,27.8,2.2,2.2,1

29/11/2009,16:14:23,27.73,2.27,2.3,1

29/11/2009,16:15:35,27.56,2.44,2.4,1

29/11/2009,16:15:46,27.6,2.4,2.4,1

29/11/2009,16:15:50,27.77,2.23,2.2,1

29/11/2009,16:15:50,27.77,2.23,2.2,1

29/11/2009,16:15:51,27.67,2.33,2.3,1

29/11/2009,16:15:53,27.8,2.2,2.2,1

29/11/2009,16:17:27,27.73,2.27,2.3,1

29/11/2009,16:17:27,27.73,2.27,2.3,1

29/11/2009,16:17:43,27.91,2.09,2.1,1

29/11/2009,16:18:08,27.77,2.23,2.2,1

29/11/2009,16:18:10,27.63,2.37,2.4,1

29/11/2009,16:18:22,27.7,2.3,2.3,1

29/11/2009,16:18:32,27.6,2.4,2.4,2

29/11/2009,16:18:42,27.63,2.37,2.4,1

29/11/2009,16:18:49,27.7,2.3,2.3,1

29/11/2009,16:18:52,27.73,2.27,2.3,1

29/11/2009,16:18:53,27.8,2.2,2.2,1

29/11/2009,16:18:56,27.8,2.2,2.2,1

29/11/2009,16:18:57,27.67,2.33,2.3,1

29/11/2009,16:19:00,27.77,2.23,2.2,1

29/11/2009,16:19:02,27.7,2.3,2.3,1

29/11/2009,16:19:23,27.42,2.58,2.6,1

29/11/2009,16:19:30,27.56,2.44,2.4,1

29/11/2009,16:19:32,27.6,2.4,2.4,1

29/11/2009,16:19:32,27.63,2.37,2.4,1

29/11/2009,16:19:35,27.56,2.44,2.4,1

29/11/2009,16:19:52,27.63,2.37,2.4,1

29/11/2009,16:19:56,27.77,2.23,2.2,1

29/11/2009,16:20:17,27.84,2.16,2.2,1

29/11/2009,16:20:24,27.8,2.2,2.1,1

29/11/2009,16:20:23,27.73,2.27,2.3,1

29/11/2009,16:20:22,27.91,2.09,2.2,1

29/11/2009,16:20:28,27.8,2.2,2.2,1

29/11/2009,16:20:40,27.8,2.2,2.2,1

29/11/2009,16:20:50,27.53,2.47,2.5,2

29/11/2009,16:20:48,27.53,2.47,2.5,1

29/11/2009,16:21:14,27.77,2.23,2.2,1

29/11/2009,16:21:12,27.77,2.23,2.2,1

29/11/2009,16:21:36,27.46,2.54,2.5,2

29/11/2009,16:21:56,27.42,2.58,2.4,1

29/11/2009,16:21:54,27.6,2.4,2.6,1

29/11/2009,16:22:21,27.63,2.37,2.4,1

29/11/2009,16:22:31,27.46,2.54,2.5,1

29/11/2009,16:22:37,27.77,2.23,2.2,1

29/11/2009,16:23:20,27.63,2.37,2.4,1

29/11/2009,16:25:39,27.7,2.3,2.2,1

29/11/2009,16:25:34,27.77,2.23,2.3,1

29/11/2009,16:25:50,27.42,2.58,2.4,1

29/11/2009,16:25:49,27.63,2.37,2.4,1

29/11/2009,16:25:50,27.7,2.3,2.3,1

29/11/2009,16:25:45,27.56,2.44,2.6,2

29/11/2009,16:26:00,27.56,2.44,2.4,1

29/11/2009,16:26:18,27.6,2.4,2.4,1

29/11/2009,16:29:15,27.39,2.61,2.6,1

29/11/2009,16:30:07,27.73,2.27,2.3,1

29/11/2009,16:30:12,27.67,2.33,2.3,1

29/11/2009,16:30:21,27.77,2.23,2.2,1

29/11/2009,16:30:28,27.46,2.54,2.5,1

29/11/2009,16:30:43,27.7,2.3,2.2,1

29/11/2009,16:30:41,27.73,2.27,2.3,1

29/11/2009,16:30:41,27.8,2.2,2.3,1

29/11/2009,16:30:46,27.6,2.4,2.4,1

29/11/2009,16:30:48,27.73,2.27,2.3,1

29/11/2009,16:30:50,27.77,2.23,2.2,1

29/11/2009,16:30:54,27.67,2.33,2.3,1

29/11/2009,16:30:57,27.7,2.3,2.3,1

29/11/2009,16:30:58,27.73,2.27,2.3,1

29/11/2009,16:31:02,27.56,2.44,2.4,1

29/11/2009,16:31:24,27.42,2.58,2.6,1
